# Fighting Antimicrobial Resistance: Innovative Drugs in Antibacterial Research

**DOI:** 10.1002/anie.202414325

**Published:** 2025-02-10

**Authors:** Roderich D. Süssmuth, Marcel Kulike‐Koczula, Peng Gao, Simone Kosol

**Affiliations:** ^1^ Institut für Chemie Technische Universität Berlin Strasse des 17. Juni 124, TC2 10629 Berlin Germany; ^2^ Medical School Berlin Department Human Medicine Rüdesheimer Strasse 50 14195 Berlin Germany

**Keywords:** Antibacterials, Drug Discovery, Antimicrobial resistance, Innovative Mode of Action

## Abstract

In the fight against bacterial infections, particularly those caused by multi‐resistant pathogens known as “superbugs”, the need for new antibacterials is undoubted in scientific communities and is by now also widely perceived by the general population. However, the antibacterial research landscape has changed considerably over the past years. With few exceptions, the majority of big pharma companies has left the field and thus, the decline in R&D on antibacterials severely impacts the drug pipeline. In recent years, antibacterial research has increasingly relied on smaller companies or academic research institutions, which mostly have only limited financial resources, to carry a drug discovery and development process from the beginning and through to the beginning of clinical phases. This review formulates the requirements for an antibacterial in regard of targeted pathogens, resistance mechanisms and drug discovery. Strategies are shown for the discovery of new antibacterial structures originating from natural sources, by chemical synthesis and more recently from artificial intelligence approaches. This is complemented by principles for the computer‐aided design of antibacterials and the refinement of a lead structure. The second part of the article comprises a compilation of antibacterial molecules classified according to bacterial target structures, e.g. cell wall synthesis, protein synthesis, as well as more recently emerging target classes, e.g. fatty acid synthesis, proteases and membrane proteins. Aspects of the origin, the antibacterial spectrum, resistance and the current development status of the presented drug molecules are highlighted.

## Graphical abstract

This article provides an overview over antibacterial drugs investigated in the past three decades. Aspects of the compound's origins, their cellular targets, the antibacterial spectrum and proof‐of‐concept in animal studies are covered and current trends are highlighted.

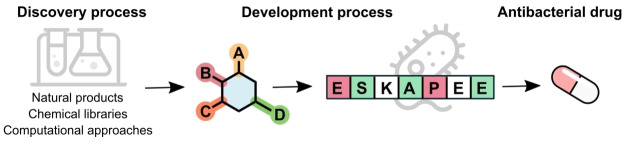

## 1 Bacterial Pathogens, Molecular Targets, Resistances and the Drug Development Process

In the fight against bacterial infections, particularly those caused by multi‐resistant pathogens known as “superbugs”, the need for new antibacterials is undoubted in scientific communities and is by now also widely perceived in the general public. However, the antibacterial research landscape has changed considerably over the past years. With few exceptions, the majority of big pharma companies has left the field and thus, the decline in R&D on antibacterials severely impacts the drug pipeline.^[1]^ In recent years, antibacterial research has increasingly relied on smaller companies or academic research institutions, which mostly have only limited financial resources, to carry a drug discovery and development process from the beginning and through to the beginning of clinical phases. This review highlights strategies for the discovery and design of new antibacterial molecules, the requirements for the profiling and drug development, and gives an overview of notable molecules from previous and ongoing antibacterial drug investigations from various sources.

### 1.1 Critical Bacterial Pathogens and Features of Physiology and Infection

The antibacterial spectrum and the targeted bacterial pathogens are of fundamental importance for drug development and the treatment of patients. In their 2019 report, the Centers for Disease Control and Prevention (CDC) lists prioritized pathogens with different degrees of severity of threat (urgent, serious, concerning and a watch list).^[2]^ Similarly, the WHO has published a list of bacterial pathogens according to critical, high and medium priority to be addressed by new antibacterials.^[3]^ In addition, the ESKAPE[^4^, ^5^]/ESKAPEE^[6]^ panel (*Enterococcus faecium* (*Ef*), *Staphylococcus aureus* (*Sa*), *Klebsiella pneumoniae* (*Kp*), *Acinetobacter baumannii* (*Ab*), *Pseudomonas aeruginosa* (*Pa*), *Enterobacter* species (*Eb*), *Escherichia coli* (*Ec*)) of nosocomial infections provides a good mnemonic of highly relevant targeted bacterial pathogens. It is important to note that most of these pathogens have developed severe levels of (multidrug‐)resistance against existing antibiotics. From these compilations, it can be easily seen, that infections with gram‐negative (G−) bacteria carrying an outer membrane as an additional barrier, pose the predominant threat to human health. Some bacteria on the watch lists are healthcare‐associated infections (HAIs), others are responsible for foodborne diseases, like Campylobacter (G−; gastrointestinal infection from food) or Shigella (G−; food and water).^[2]^ Sexually transmitted diseases (STDs) like those caused by *Neisseria gonorrhoeae* (*Ng*, G− cocci) or cell wall lacking *Mycoplasma genitalium*, have more recently gained attention due to the development of multi‐resistant strains.[^2^, ^7^] These and many other bacterial pathogens, despite their cause or the severity of infection, fall under the treatment regimen using existing antibiotics. Further examples are Lyme disease caused by *Borrelia burgdorferi* (G− spirochete), gastric ulcers caused by *Helicobacter pylori*, or *Bordetella pertussis* (G−) infections of the respiratory tract, mostly in children. More special applications of antibiotics are infections with anaerobe, toxin‐producing *Clostridioides difficile* (*Cd*, G+) causing symptoms of diarrhea, commonly treated with, but not exclusively, oral antibacterial therapy.^[2]^ Finally, *Mycobacterium tuberculosis* (*Mt*) causing tuberculosis requires a particular therapy adapted to the bacterial lifecycle.^[8]^ This is driven by a difficult to penetrate mycobacterial cell wall and the invasion of the bacterium in the host cell followed by intracellular persistence. Metabolism and cell division is slow to dormant, demanding therapy times exceeding those of classical antibacterial therapies.

The infection and subsequent interactions with host cells or tissues is quite complex and depends on the bacterial strain. For the bacteria mentioned above, this process is commonly accompanied by production of **virulence factors** (cytosolic, secretory or membrane‐bound),[^9^, ^10^] which can be toxins, hemolysins, adhesins, or siderophores, and with regard to their genetics are chromosomally‐encoded or derived from mobile genetic elements. In that context, **quorum sensing** (**QS**)^[11]^ is part of the regulation and synthesis of virulence factors. One area of intensive research addresses the interference of QS and the synthesis and sequestration of virulence factors as a potential therapeutic concept.^[9]^ As virulence factors are commonly not essential for survival, this approach only attenuates virulence. Examples are found in many G− bacteria which possess secretion systems (type III and IV) that have the function of injecting virulence factors into the host cell.^[12]^ Mutations in these systems cause avirulence. Natural products such as guadinomines target the type III secretion system (TTSS).^[13]^ They are highly potent inhibitors of hemolysis of enterohemorrhagic *E. coli* (EHEC), but however show no bacterial killing.

Many bacterial pathogens, including some from the ESKAPE(E) panel, also form **biofilms** embedded (Figure 2) in an extracellular matrix. Biofilms come with lower susceptibility to the antibiotic and are associated with chronic infections. Hence, there have been approaches to interfere with various stages of biofilm formation, e.g. attachment, quorum sensing and detachment/dispersal.[^14^, ^15^, ^16^] However, observations of resistance development against quorum quenchers (QQ)[^17^, ^18^] have been made and the therapeutic concept of QS interference is under debate.[^19^, ^20^, ^21^] Despite the identification of suited inhibitory molecules, the lack of an immediate reduction in growth or clearance of viable cells is, from a therapeutic perspective, an argument for staying with conventional antibacterial therapy, particularly in the case of immunocompromised patients.

### 1.2 Antibacterial Chemotherapy – Established and New Target Classes

The basic requirements for an **ideal antibacterial target** are 1) essentiality for bacterial growth or survival, 2) a low tendency of evolving mutations or bypassing, leading to resistance and 3) a good target accessibility. **Bactericidal** effects, i.e. a killing action, are sometimes preferred over **bacteriostasis** but are case‐dependent and may also cause a challenge to the immune system, e.g. due to induction of toxic shock or rapid lysis of bacteria.^[22]^ More importantly, the antibacterial drug should not cause the rapid development of mutations and the target should not be easily altered by mutations either, assessed by measuring the frequency of resistance FoR (synonymously used: frequency of mutation, FoM).[^23^, ^24^] Unwanted off‐target effects can be avoided if the target does not exist in humans or is only distantly related to human proteins or cellular structures.[^1^, ^25^]

**Figure 1:**
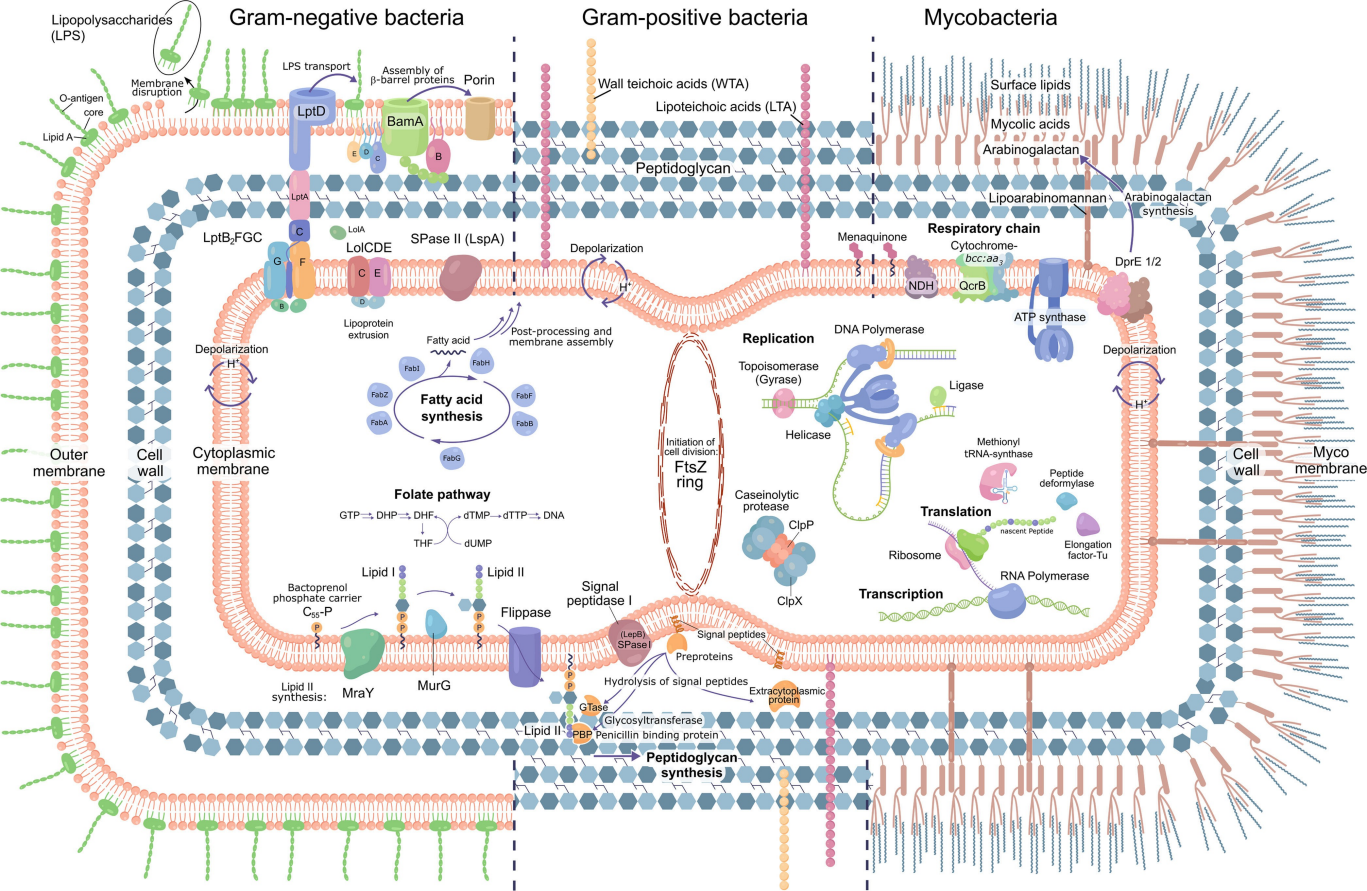
Schematic of established and new antibacterial target structures in gram‐negative (G−) & gram‐positive bacteria (G+), as well as mycobacteria. The outer membrane of G− bacteria represents an additional barrier for drugs required to reach the cytoplasm and molecular target structures. Mycobacteria have a particular cell wall architecture and a specialized metabolism.

The **classical and established antibacterial targets** are cell wall synthesis (peptidoglycan), ribosomal protein synthesis, gyrase and DNA synthesis, RNA polymerase, the folate pathway and the cell membrane (Figure 1).^[26]^ This is complemented for anti‐mycobacterial drugs by mycolic acid synthesis.^[27]^ Many of these drugs are used as monotherapy, targeting macrobiomolecule biosynthesis pathways.[^1^, ^28^] Deregulation of central processes functioning as junctions or hubs in many directions of various cellular functions, is highly effective. In this context, the opinion clearly prevails that antibacterials addressing multiple targets or causing multiple effects are more potent and avoid rapid resistance development, rather than those addressing a single target structure.[^1^, ^28^] Therefore, single‐gene targets bear the risk of rapid emergence of high level resistance and might require combination therapy with other antibacterials, as practiced, e.g. for trimethoprim/sulfonamides. In an attempt to categorize antibacterial effects of drugs,^[28]^ **multi‐target** compounds either inhibit multiple targets in one pathway or different targets in different pathways, whereas **multi‐effective** drugs with preferably one target hit several finely tuned processes, ultimately leading to a breakdown of important cellular functions. As a definition for the **novelty of a target structure** we suggest an autonomous cellular (sub)structure or protein domain for which no inhibitor is known thus far. An antibacterial drug which addresses a different binding site at a known target is still valuable, as it lowers the risk of cross‐resistance.

In the search for new inhibitory compounds, sticking to established target structures bears the risk of cross‐resistance. Nevertheless, **established targets** are targets which work, and this aspect should be carefully considered before disposing of a potential candidate molecule. Turning to **new targets** is basically considered to be more innovative and indeed the exploration of new and essential gene functions promises new molecular scaffold structures with a lower risk of the development of cross‐resistance.^[1]^ Even if already researched for quite some time, more recent targeted pathways include **fatty acid biosynthesis**,[^29^, ^30^] **cell division** with FtsZ as a major player,^[31]^ **protein processing and degradation by proteases** ^[32]^ and **membrane biogenesis** and **membrane integrity** (Figure 1).

In order to reach **intracellular** targets in the cytoplasm, a drug has to cross bacterial membranes and cell wall structures. A particular challenge is the outer membrane (OM) of G− bacteria (Figure 1) which represents a major barrier that is difficult to overcome. Therefore, target structures in proximity of **membranes** or the **cell wall** have the advantage of being better exposed to drug interactions. This has been addressed by recent research efforts on inhibitors of the lipopolysaccharide transport machinery (Lpt) involved in outer membrane biogenesis^[33]^ as well as the β‐barrel assembly machinery (BAM) of G− bacteria.[^34^, ^35^]

Not further outlined in this article is research on inhibitors of **virulence** and their potential use as therapeutics aiming at disarming bacteria, rather than killing and thus reducing selection pressure for development of resistance.^[36]^ A counterargument to anti‐virulence strategies is that severe infections require rapid intervention and the outcome of virulence inhibition is unsure.

Likewise and not further discussed is **vaccination**, which successfully can confer immunity against bacterial toxins produced during infection, e.g. diphtheria toxin (*Corynebacterium diphtheriae*), pertussis toxin (*Bordatella pertussis*) or also Shiga toxin (enterohemorrhagic *E. coli* (EHEC)), and in such cases the application of antibiotics may even foster toxin production.^[37]^ For decades the vaccination approach has been applied to protect populations from diphtheria and tetanus (*Clostridium tetani*) infections.^[38]^ Another vaccine‐based strategy is to generally reduce the burden of infection by a pathogen. A number of vaccine candidates against various bacteria are currently in clinical development.^[39]^ Further therapeutic options comprise the administration of **monoclonal antibodies** (**mABs**). Indeed, this is a field of high activity targeting secretory toxins, surface proteins and polysaccharides of specific pathogens.[^40^, ^41^] Advantages are seen in direct toxin neutralization, or the involvement of phagocytosis/complement‐activation of the innate immune response. Challenges are the variability of polysaccharides and the accessibility of surface proteins. The first FDA‐approved anti‐virulence therapeutic bezlotoxumab is a monoclonal antibody designed to bind the *Clostrioides difficile* toxin TcdB.^[42]^ Clinical trials are underway for mABs against *S. aureus*, *P. aeruginosa*, *Salmonella* Typhimurium and *E. coli*.[^40^, ^43^]

### 1.3 Drug Resistance Mechanisms and Inhibitors

**Figure 2:**
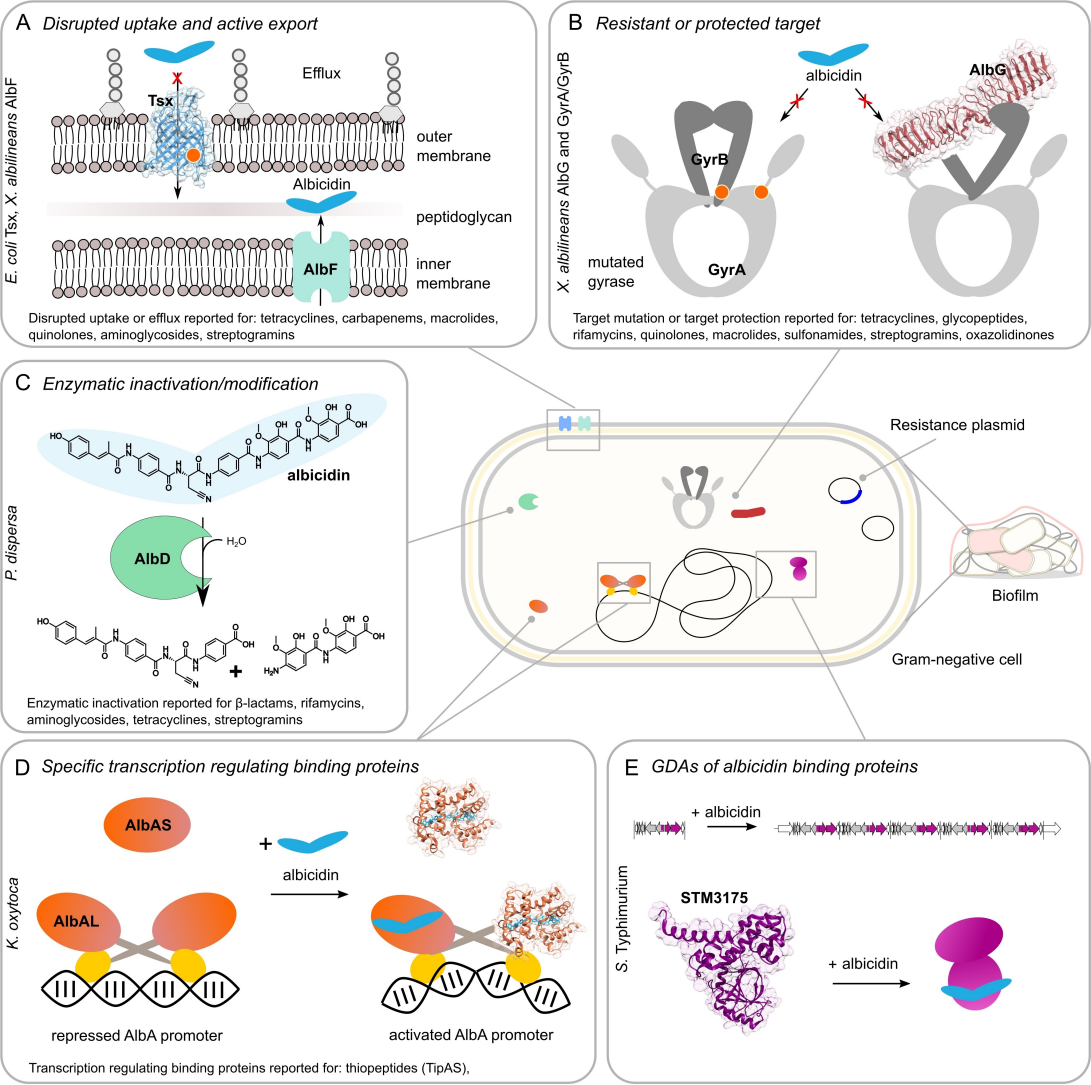
Major forms of resistance mechanisms against antibacterial drugs explained with the example of albicidin in a schematic Gram‐negative cell. A) *E. coli* Tsx transporter of the outer membrane (blue, PDB‐ID: 1TLY) with mutations (orange dots) and the *X. albilineans* efflux pump AlbF (mint green, no structure available). B) Gyrase may have mutated (orange dots). In *X. albilineans* the pentapeptide repeat protein AlbG (dark red, PDB‐ID: 2XT4) protects gyrase. C) In *Pantoea dispersa* the specific peptidase AlbD (green, no structure available) cleaves albicidin. D) The albicidin binding protein AlbA (orange, PDB‐ID: 6ET8) from *Klebsiella oxytoca* acts in its di‐domain form (AlbAL, orange and yellow) as an auto‐regulating transcription factor and its shorter form (AlbAS, orange) without a DNA‐binding domain sequesters albicidin. E) STM3175 (purple, PDB‐ID: 7R3W) from *Salmonella* Typhimurium binds albicidin and its gene is subjected to gene duplication amplifications (GDAs) in presence of albicidin.

Within the next few decades, antibiotic resistance, already one of the major threats in health care, is expected to lead to a drastic increase in deaths due to bacterial infections.^[44]^ The existence and evolution of resistance mechanisms against all currently available compound classes presents a difficult challenge for the development and application of antibacterials. And although many bacteria have shown resistances, six pathogens have emerged as particularly problematic: *Enterococcus faecium*, *Staphylococcus aureus*, *Klebsiella pneumoniae*, *Acinetobacter baumannii*, *Pseudomonas aeruginosa* and *Enterobacter* species – the ESKAPE organisms.^[4]^ These pathogens cause the majority of nosocomial infections and are, together with *E. coli* and *Streptococcus pneumoniae* also the leading cause for deaths associated with resistance.^[45]^ Since the introduction of penicillin, medicine finds itself in an arms race against bacterial pathogens that become resistant to new antibiotics with increasing speed. The **microbial defense strategies** can be summarized in the following four main categories: 1) reduced uptake of the drug, 2) active efflux of the drug 3) (enzymatic) modification of the drug, and 4) modification of the target. These mechanisms are employed by G+ and G− bacteria, with an additional level of protection granted by their outer cell membrane in case of the latter (Figure 2). Resistance factors are acquired by horizontal gene transfer (e.g. by plasmids) as is the case for β‐lactamase, the well‐known enzymatic defense against penicillin and related compounds.^[46]^ Resistance can also be obtained by mutations such as deletions, gene duplications and point mutations rendering the cellular target immune as is the case for ciprofloxacin resistance where several mutations in gyrase and topoisomerase IV lead to a high level of quinolone resistance.^[47]^ The above mentioned types of mechanisms enable bacteria to become resistant against multiple antibiotics and to show **multidrug resistance** (**MDR**), i.e. resistance at least against one representative from each of three or more antimicrobial drug categories.^[48]^ MDR is a major concern for healthcare because of the limited number of available treatment options. In addition to resistances arising from genetic diversity, the phenotypic heterogeneity of a population results in transiently tolerant **persister cells** that survive antibiotic exposure owing to lowered metabolic activity.^[49]^ Persistent bacteria might require prolonged antibiotic treatment or cause therapeutic failure.

Several review articles provide detailed overviews of resistance mechanisms against various antibiotics.[^50^, ^51^, ^52^] The peptide antibiotic **albicidin** serves as an excellent example to illustrate the large arsenal of defense mechanisms available to bacteria against antibiotics (Figure 2). Striving to understand and overcome resistance mechanisms against albicidin, we and other groups have studied the various mechanisms in detail. Albicidin inhibits DNA synthesis by targeting DNA gyrase, and both gyrase subunits in its producer *Xanthomonas albilineans* have multiple insertions and changes of amino acid residues that make the enzyme less susceptible to albicidin.^[53]^ But *X. albilineans* has additional mechanisms at its disposal to protect itself from the antibiotic: The albicidin‐biosynthetic gene cluster encodes the efflux pump AlbF which actively removes albicidin from the cytoplasm^[54]^ and the pentapeptide‐repeat protein AlbG, which binds to, and protects DNA gyrase.^[55]^

In albicidin non‐producer organisms, several markedly different resistance mechanisms have been found. *E. coli* usually gains resistance against albicidin through mutations in the gene of the nucleoside transporter Tsx, which has been shown to be responsible for the cellular uptake of albicidin.^[56]^ The G− *Pantoea dispersa* produces the endopeptidase AlbD,^[57]^ which cleaves albicidin into two inactive fragments.^[58]^ In *Klebsiella oxytoca* and *Alcaligenes denitrificans*, the highly specific drug‐binding proteins AlbA^[59]^ and AlbB^[60]^ have been identified. AlbA and AlbB sequester albicidin and act in an auto‐regulatory fashion to increase their own expression levels when albicidin is bound.[^61^, ^62^] Recently, we discovered a gene‐amplification‐based resistance mechanism in *Salmonella* Typhimurium and *E. coli* that causes high‐level albicidin resistance by increasing the cellular levels of the albicidin‐binding transcription regulator STM3175.^[63]^ This broad variety of resistance mechanisms highlights the difficulty in developing drugs that effectively target a bacterial infection.

**Figure 3:**
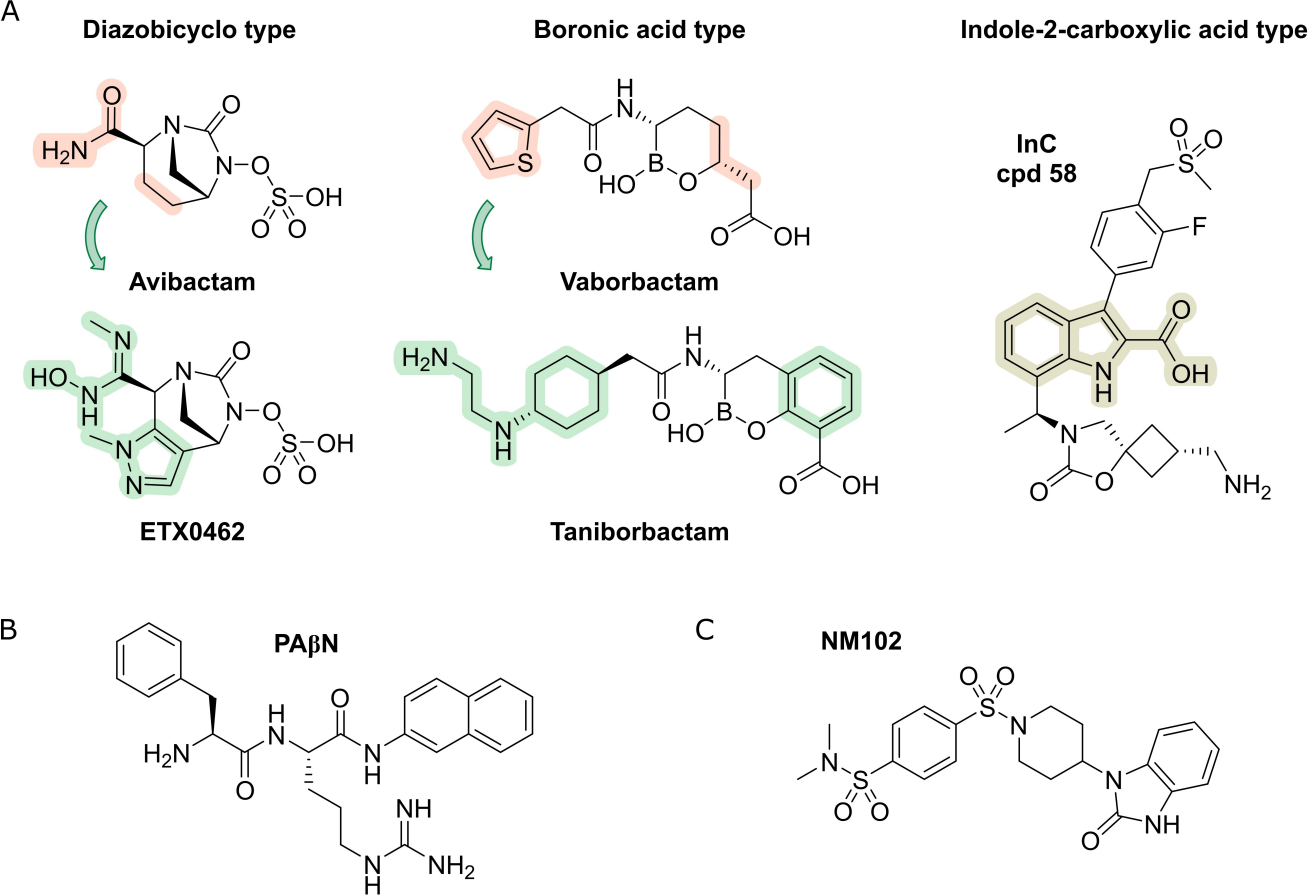
Inhibitors of bacterial resistance factors: A) Structures of β‐lactamase inhibitors binding penicillin‐binding proteins, B) of the efflux pump inhibitor PaβN and C) of the Mutation Frequency Decline (Mfd) protein binding molecule NM102.

One strategy is to directly counter resistance factors with drugs and thus to restore the activity of antibacterials: A highly successful concept is the use of β‐lactams in combination with **β‐lactamase inhibitors** (BLIs, Figure 3A) to overcome β‐lactam resistance particularly of G− bacteria.^[64]^ In this research field many pharmaceutical companies are still active. The BLIs, which are all small molecules, mostly without intrinsic antibacterial activity, address a wide range of serine β‐lactamases (SBLs), while metallo‐β‐lactamases (MBLs) remain challenging.^[64]^ In recent years, several non‐β‐lactam BLIs have reached preclinical to clinical phases, e.g. the intrinsic antibacterially active **ETX0462** (diazabicyclooctane, DBO; Figure 3A and Table 1),^[65]^ or have been approved like **avibactam** (DBO, weakly intrinsic antibacterial) and **vaborbactam** (boronic acid monoester).^[66]^ The search for inhibitors of MBLs is being performed with great efforts and has yielded candidates such as **taniborbactam** (clinical phase III)^[67]^ or **indole‐2‐carboxylates** (InCs, ongoing research) (Figure 3A).^[68]^

**Drug efflux** accounts for one of the most important resistance mechanisms. **Efflux pump inhibitors** (**EPIs**) disable efflux pumps, which however are structurally and mechanistically diverse.^[69]^ As a consequence, the discovery of a universal EPI is unlikely and rather an inhibitor has to be adapted to the targeted bacterium and the targeted efflux pump, respectively. Probably, the best‐known efflux inhibitor is Phe‐Arg‐β‐naphthylamide (**PAβN**, MC‐207–110, Figure 3B) which could reverse resistance of *P. aeruginosa* when the bacterium overexpresses multidrug‐efflux pumps.^[69]^ The search for EPIs however has not led to a marketed drug.^[6]^ Likewise, other mechanisms of bacterial drug resistance are highly diverse and often are tailor‐made in order to efficiently eliminate the targeted antibiotic. Hence, escape from a resistance mechanism is commonly achieved synthetically by structural variations of the drug. Recent research approaches aim at targets reducing evolving resistance, for example molecules like **NM102** (Figure 3C), which is an inhibitor of the Mutation Frequency Decline (Mfd) protein that uses ATP to translocate RNA polymerase (RNAP) along DNA and to dissociate it from the mRNA transcript.^[70]^

### 1.4 Antibacterial Profiling and the Drug Ddevelopment Process

**Figure 4:**
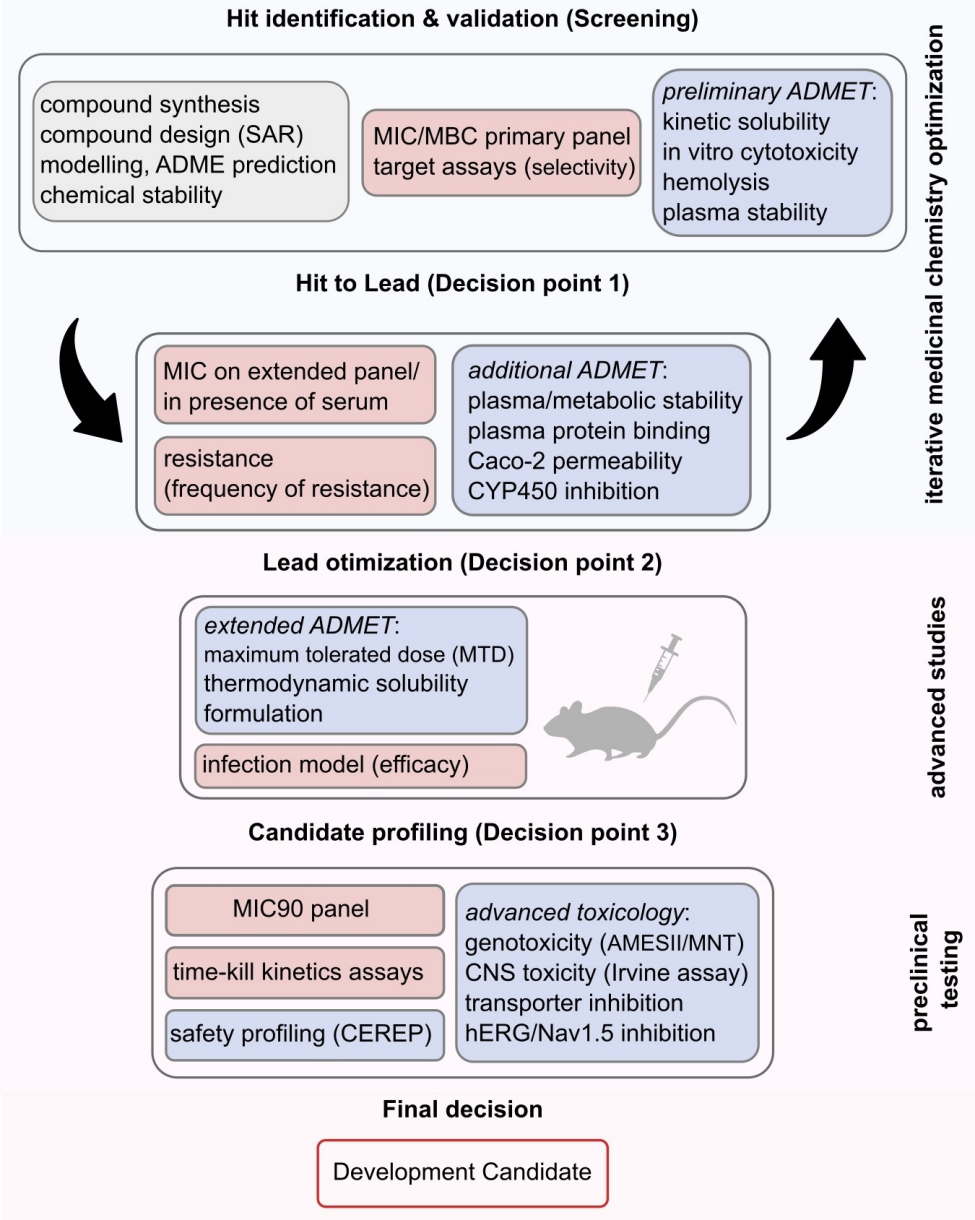
Schematic workflow for the progression of antibacterial hits (here from a phenotypic screening) to the identification of a development candidate (filing of patents and later development stages are not considered; red boxes: microbiology and infection model, blue boxes: pharmacokinetics (PK), safety & toxicity (cell biology, animal models, etc.)). ADME(T)=absorption, distribution, metabolism, excretion, (toxicity). hERG/Nav1.5 inhibition assays serve to determine cardiotoxicity. CEREP refers to binding assays to identify potentially problematic binding to receptors, ion channels and transporters.

The development of an antibacterial drug starting from a screening hit proceeds in stages schematically shown in Figure 4. It is a complex process where the candidate molecule can fail at any moment if certain criteria are not met. In the first level of a phenotypic screening (hit finding), which still is the main screening approach, compounds from different sources (e.g. natural products, synthetic molecules) are screened on an initial panel of bacterial pathogens. Hit compounds from other screenings (e.g. target‐based, structure‐based, etc.) have to be evaluated for their antibacterial activity. The whole process is accompanied by DMTA cycles (design–make–test–analyze). A quantitative evaluation by **minimal inhibitory concentration** (MIC) assays is conducted and ideally also the performance in a biochemical target assay. Of high importance are **haemolysis** and **cytotoxicity** assays in order to reduce the chance of toxic side‐effects in subsequent animal experiments. The assessment of bactericidal activity (MBC) is a parameter to understand, whether the candidate molecule has a killing (bactericidal) or rather a growth inhibitory (bacteriostatic) effect. In an extended assessment round (subsequent to decision point 1), the activity spectrum is characterized as well as the type and **frequency of resistance** (FoR). If the latter is too high, further development may be discontinued. Aspects of chemical/plasma and metabolic stability need to be considered as well. The subsequent **maximum tolerated dose** (**MTD**) study in animals, provides an impression on the tolerability of the dosing from which the first pharmacokinetic parameters (plasma half‐life, maximum serum concentration) can be derived. The in vivo efficacy assay using an **animal infection model** (thigh, sepsis, lung infection, etc.) provides the proof‐of‐concept for the action of the antibiotic in an animal species. Depending on the anticipated application area/Target Product Profile (TPP), data from different animal infection models (commonly in mice, for *C. difficile* in a hamster model) are assessed. Subsequent to decision point 3, the candidate will be profiled against hundreds of clinically relevant bacterial isolates (MIC50, MIC90) and also tested in a wide range of assays addressing aspects of toxicity and safety. Following these studies, a developmental candidate for clinical phase I may result.

Phase I clinical trials are designed to collect data on the **safety** and **side effects** of a new treatment. While approved antibiotics are generally considered as comparatively safe, occasionally occurring side effects can be quite serious. The adverse effects can vary between mild and severe, depending on the cellular targets and physico‐chemical properties of the antibiotic compound. For example, ototoxicity and nephrotoxicity are well‐documented detrimental effects of aminoglycosides and tendinopathy and tendon ruptures have been reported for quinolones.[^71^, ^72^] Antibiotics targeting the bacterial translation machinery frequently induce mitochondrial dysfunction since the organelles are bacteria‐derived and share similarities in their proteins.^[73]^ Mitochondrial dysfunction, in turn, has been linked to neurological disorders, abnormal behavior conditions and mental disorders such as depression or psychosis.^[74]^ On top of that, it has been shown that most bactericidal antibiotics such as quinolones, aminoglycosides and β‐lactams act as stressors that lead to overproduction of reactive oxygen species (ROS) thus causing oxidative damage and the disruption of the electron transport chain.^[75]^ Mitochondria are similarly affected and release ROS that can cause oxidative damage in mammalian tissues.^[76]^ For example, a number of ciprofloxacin‐treated patients have been reported to develop psychosis,^[77]^ which has been attributed to an increased release of ROS and the interference of ciprofloxacin with the binding of gamma‐aminobutyric acid (GABA) to its receptor, leading to stimulation of the central nervous system.[^77^, ^78^]

Additionally, the effects of antibacterials on the **host microbiota** have to be considered. Broad spectrum antibiotics, for example, can have an impact on the microorganisms that live in and on humans, causing not only changes in the composition of the microbiota but also in its resistome,[^79^, ^80^] i.e. the resistance genes present in the commensal bacterial community. Several studies have linked imbalances in the gut microbiota after antibiotic treatment to increased prevalence of obesity and asthma.[^81^, ^82^, ^83^] Changes in the microbiota composition can result in long‐term effects which are often most severe in children, arguing for more pathogen‐specific antibiotic therapies.[^81^, ^82^] To reduce damage to the host microbiota as well as to lower the selective pressure and therefore emergence of resistances, bacterial anti‐virulence therapy has been suggested to circumvent both.[^84^, ^85^] In this approach, functions that are essential for infection, such as virulence factors, are targeted. Most strategies focus on toxins, adhesion, two‐component systems or bacterial communication with the goal of disarming the pathogens (see section 1.1.). An alternative approach is the use of narrow‐spectrum antibiotics, also termed “microbiome sparing” which avoids the eradication of entire microbial communities.^[86]^

Finally, it needs to be mentioned particularly for academic groups that the assessment of drug molecules requires verification of the novelty of interesting molecules throughout the process, accompanied by the **filing of patents** and the development of a strong patent portfolio prior to publication. Otherwise, exploitation for development as a drug may become compromised. Further actions include, the establishment of a target product profile (TPP), the development of a business plan or partnering with industry.

## 2 Sources of New Antibacterial Drugs and Search Strategies

**Figure 5:**
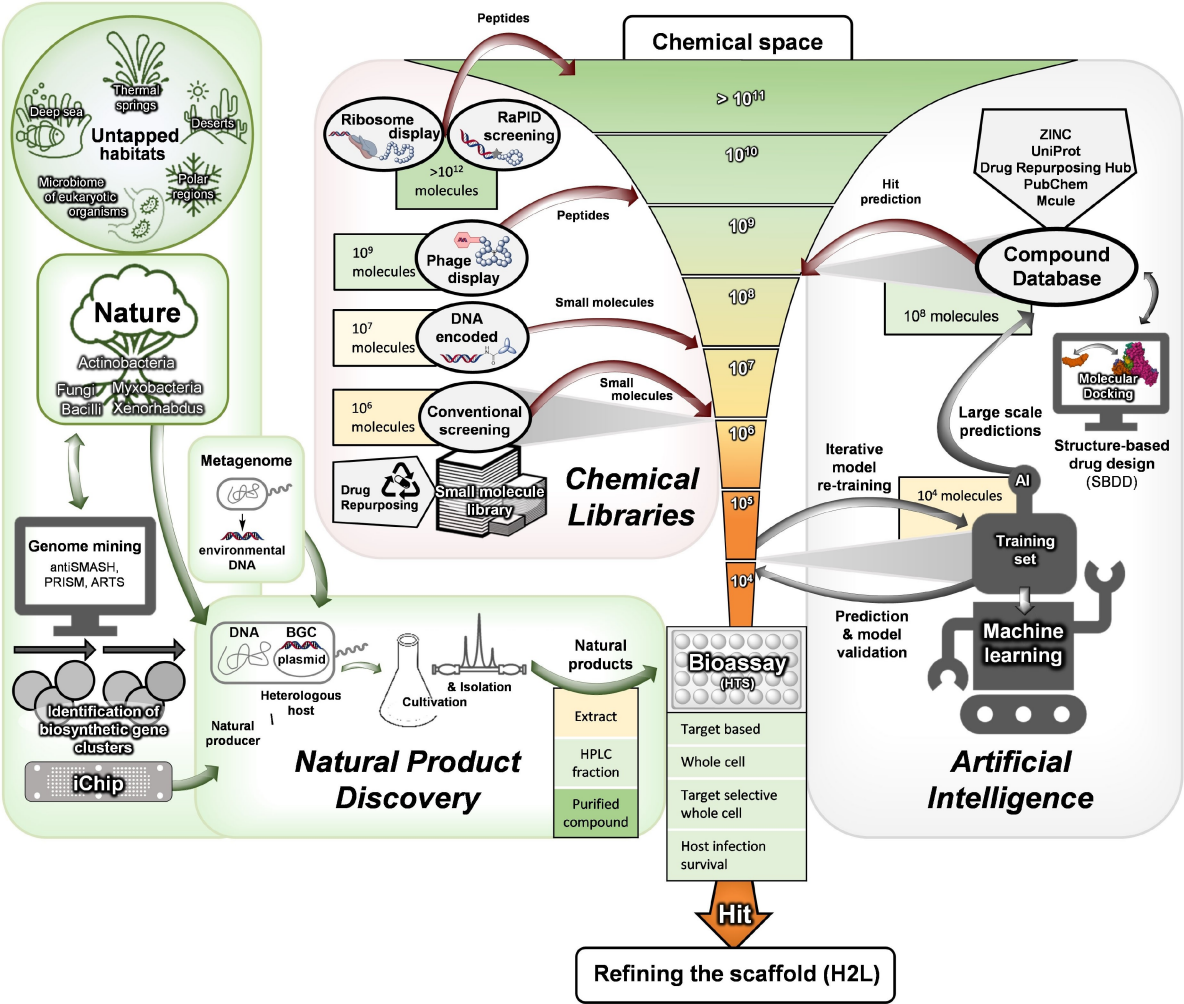
Strategies for the discovery of antibacterial drugs from natural sources, by molecular biology, biochemistry and synthesis‐based methods as well as AI/ML‐based methods.

In an ideal world, searching for a new antibacterial drug would succeed by uncovering an unprecedented chemical entity targeting a novel and essential biosynthetic pathway.[^1^, ^25^] In subsequent refinement rounds this new chemotype would be optimized to improve properties, e.g. broadened or narrowed antibacterial spectrum, increased potency as well as enhanced pharmacokinetics and pharmacodynamics (efficacy). Generally speaking, new structural scaffolds either come from nature's biosynthetic depository or are provided by chemical synthesis. Various search strategies can be employed to find suitable hits of interest. Unbiased or focused phenotypic screenings[^87^, ^88^] are contrasted by rational, target‐based drug discovery (TBDD)[^89^, ^90^] or structure‐based drug design (SBDD) strategies (Figure 5).^[91]^ Nowadays, high‐throughput screening (HTS) methods enable testing of hundreds of thousands to millions of compounds in a small amount of time (from days to weeks). More recently, cell‐based high‐content screening (HCS)[^88^, ^92^, ^93^] and computational methods,[^94^, ^95^, ^96^] employing virtual screening, e.g. HT‐docking or artificial intelligence (AI), have gained momentum (Figure 5). Phenotypic screenings have the advantage of directly monitoring the effect on bacteria.^[87]^ Target identification (target ID) and structure‐based drug design are supported by an arsenal of available molecular biology‐, biochemistry‐ and omics‐based techniques which can comparatively straightforwardly reveal the impacted pathway(s).^[97]^ In many cases molecular biology tools, e.g. generation of gene deletions, antisense technologies,^[98]^ etc. to manipulate the pathogen and to understand the significance and essentiality of the target, are already at hand. An additional approach for target ID consists in eliciting resistance against the drug of interest under laboratory conditions. The genomes of most bacterial pathogens are known^[99]^ and resistances arising can be quickly spotted.

### 2.1 De Novo Scaffold Identification from Nature

A view on the currently available antibacterial defense line (Figure 10) reveals that thus far nature has provided the majority of scaffolds clinically used as antibiotics. In addition, it has to be realized, that structures and complexity of mode‐of‐actions (MOAs) often lie beyond chemist's intuition and design principles. Previously, the **“classical” screening** (Figure 5) of fungal and predominantly bacterial strain collections by research teams from academia and industry was the workhorse for finding many antibacterials. The most prolific sources were actinobacteria (including the genus *Streptomyces*) and bacilli, complemented some time ago by myxobacteria.^[100]^ The majority of the **natural products** (**NPs**) comprises non‐ribosomal peptides (NRPs),^[101]^ polyketides (PKs),^[102]^ mixed PK‐NRP‐type, and ribosomally synthesized and post‐translationally modified peptides (RiPPs).^[103]^

Natural product screening requires well‐curated strain collections which are successively submitted to cultivation and screening. There are two main screening approaches:[^104^, ^105^, ^106^] 1) structure‐guided screening, in which compounds featuring novel structural attributes are submitted to the bioassay and 2) bioactivity‐guided screening which selects bioactive extracts, fractions or compounds followed by structural identification of the hit and dereplication^[107]^ to differentiate between known and unknown compounds. The bioassay either monitors antibacterial activity (whole‐cell/phenotypic screening), directly addresses a specific molecular target (target‐based screening) or both (including specialized assays, e.g. organisms with down‐regulated target). A sophisticated approach is the screening for efficacy of compounds in a miniaturized infection model of hosts like zebrafish, nematodes, insects, e.g. the greater wax moth (*Galleria mellonella*) or the fruit fly (*Drosophila*) and to additionally assess safety aspects of a hit compound.^[108]^ In order to increase the microbial and also chemical diversity, search strategies^[109]^ for microbial strains have been expanded to include previously understudied species, e.g. bacterial symbionts of the genera *Photorhabdus* and *Xenorhabdus*,[^110^, ^111^] untapped habitats (deep sea,^[112]^ deserts,^[113]^ human microbiota^[114]^ etc.) or to variation of cultivation conditions, and induction of silent genes by reverse genetics (interference with biosynthetic gene cluster (BGC) regulation).^[115]^ These screening approaches have turned out to be robust and have delivered compounds beyond the previously established antibacterial target structures. A remarkable achievement has been the expansion of the screening approach towards difficult‐to‐cultivate bacteria by development of the iChip technology (“Great Plate Count Anomaly”^[116]^) (Figure 5).^[117]^ Finally, the observation of **physiological effects**, which is the action as toxins, protectants or pathogenicity factors, has occasionally contributed new molecules.^[118]^ Noteworthy examples comprise albicidin which causes chlorosis in sugar cane plants,^[119]^ and lugdunin which lowers the chance for nasal colonization by *S. aureus* in patients. Nowadays, many natural products have been compiled and classified in various databases, which is of help in compound identification, dereplication and characterization, respectively.^[120]^

Gene‐technological progress in past decades has provided significant advancement in **heterologous expression** of sizeable biosynthetic gene clusters as potential sources of new natural products, particularly of bacterial secondary metabolite producers.^[121]^ This enables biosynthetic engineering in the heterologous host, but also the detection and activation of rare or non‐expressed genes and gene clusters.^[115]^ Ultimately, the developed technologies are key for approaches making use of environmental DNA (eDNA)^[122]^ or metagenomic DNA,^[123]^ but efforts have moved away from unbiased expressions to rather directed approaches e.g. with knowledge about the targeted gene sequences.

A promising application in compound discovery, which has emerged during recent decades, was introduced by **genome‐mining** tools (Figure 5) used in bioinformatic searches.^[124]^ High‐throughput DNA sequencing capabilities have resulted in a continuously increasing volume of genetic data from biosynthetic genes, gene clusters and microbial genomes which continues to fill databases. This in turn has facilitated rational searches for new biosynthetic functions and thus the identification of new natural products. Corresponding **bioinformatic tools** like antiSMASH,^[125]^ PRISM^[126]^ or ARTS,^[127]^ to name a few, are of great help in the identification and prediction of biosynthetic functions, gene clusters, metabolite structures and their likely biological activity. The popular genome‐mining tool antiSMASH enables the identification and annotation of secondary metabolite gene clusters from different organisms including bacteria, archaea and fungi with its accompanying database currently containing >230,000 biosynthetic gene clusters.^[128]^ More specialized on procaryotic sequencing data, the PRISM platform predicts the chemical structures of genomically encoded antibiotics with even higher accuracy.^[126]^ By exploiting the presence of auto‐resistance genes in antibiotic producing bacteria, ARTS specifically mines genome data for antibiotic‐encoding gene clusters with novel targets.^[127]^ However, predictions on biosynthetic product structures depend on the training set of existing structures and are not in all cases successful. This is particularly true if a compound displays unprecedented structural features. Nevertheless, tools are further improving and the identification of biosynthetic novelty with genome mining directly enables work with the strain of interest and the focus on a corresponding gene cluster.

### 2.2 Chemistry and Chemical Biology‐Based Identification of New Chemical Matter

Previously, chemical synthesis approaches have added sulfonamides, fluoroquinolones and oxazolidinones to the defense line of antibacterials used in human healthcare (Figure 10).^[26]^ With the improvement of **synthesis methods** and the emergence of **combinatorial & multi‐parallel chemistry** as well as automated synthesis methodology beginning in the 1990s, increasing numbers of small molecule compounds, particularly in the pharmaceutical industry, has been funneled into screening campaigns (Figure 5), e.g. target‐based screenings employing high‐throughput (HT) formats. The assembly of **small molecules** is commonly based on short synthesis sequences, which allows for a straightforward structural diversification. In **target‐based screenings**, hit compounds are identified, and subsequently, by optimization (the H2L process), lead structures are derived. Rational approaches in target selection were spurred by genomics in combination with the application of additional filters (e.g., abundance across species, cellular toxicity). In this context, two reports on target‐based HTS screening campaigns for antibacterials need to be mentioned: one by GlaxoSmithKline (GSK, 70 campaigns, 1995–2001)^[89]^ and the other by AstraZeneca (65 campaigns, 2001–2010).^[90]^ Both of which provide insights into the massive efforts put into the search. While these medicinal chemistry efforts could provide high affinity binders of a target, whole‐cell activity was more difficult to achieve because of difficulties for these hit compounds to cross bacterial membranes or to avoid being expelled by efflux pumps. Whole‐cell antibacterial testing against *E. coli* and *S. aureus* produced no hits or leads,^[89]^ and generally speaking success rates were rather moderate.[^89^, ^90^] Nevertheless, successes of small molecules are also known: one such example is the antimycobacterial diarylquinoline **bedaquiline** (approval 2012 (U.S. Food and Drug Administration, FDA)/2013 (European Medicines Agency, EMA); Figure 10).^[129]^ The compound class was identified from bioactivity screening in a whole cell (phenotypic) assay, and only subsequently was ATP synthase determined as the molecular target.

The screening of libraries of known drugs is the basis of **drug repurposing** (Figure 5).^[130]^ Examples are kinase inhibitors against *S. aureus*,^[131]^ which shows that these anticancer compounds had ClpP activity with activity against G+ bacteria.^[132]^ Another example is lansoprazole which interferes with the respiratory chain (anti‐*Mt*).^[133]^ Several of these drugs indeed show noteworthy antibacterial activities, which, however, then require further optimization.

**Library concepts** (Figure 5) exert their strength by the rapid generation of large compound numbers from a starting structure, and are used particularly in target‐based screenings. One noteworthy example is the β‐hairpin‐type 14‐mer peptide murepavadin (Figure 8C), which was a rationally designed **protein epitope mimetic** (**PEM**) from protegrin I as a starting structure. In several synthetic rounds amino acid positions were permutated in order to find the most active compound.^[33]^ The development of further library concepts facilitated miniaturization as well as even larger compound numbers (>10^6^–10^7^ compounds): This includes **DNA‐encoded libraries** (**DECLs**)^[134]^ and very powerful **display technologies** (phage‐/ribosome‐/RNA display, e.g. PeptiDream technology),[^135^, ^136^] which is based on affinity binding to the target followed by dereplication of the binding molecule. One example of a DECL is photocleavable amino acid‐based trimers on 85,000 beads, from which 97 beads showed growth inhibition (*E. coli*/*B. subtilis*). While a number of hits unsurprisingly contained a terminal fluoroquinolone, this proof‐of‐concept study nevertheless outlines the potential for identification of new hits. **Phage display** has been previously used^[137]^ to raise cyclopeptide binders against lipid II, which upon bicyclization and lipidation showed activity against various G+ bacteria. In addition to phage display, **RNA display** is a leading technology that selects hits from an enormous number of peptides (library size ~10^12^–10^14^ compounds).[^138^, ^139^] The library size can be further expanded by including non‐proteinogenic amino acids.^[140]^ Cell‐free expression and flexizyme‐based codon reprogramming has already been applied to a thiopeptide antibiotic scaffold,^[141]^ an approach that could potentially lead to antibacterial thiopeptide analogs.

Finally, there are reports that suggest **metal‐organic complexes** could be potential sources of antibacterials.^[142]^ However, from a current medicinal chemistry viewpoint, complexes of cobalt, ruthenium and of other metals would not be attractive options for consideration as systemically applied drugs in human.

### 2.3 Molecular Docking, Artificial Intelligence and Machine Learning

**Figure 6:**
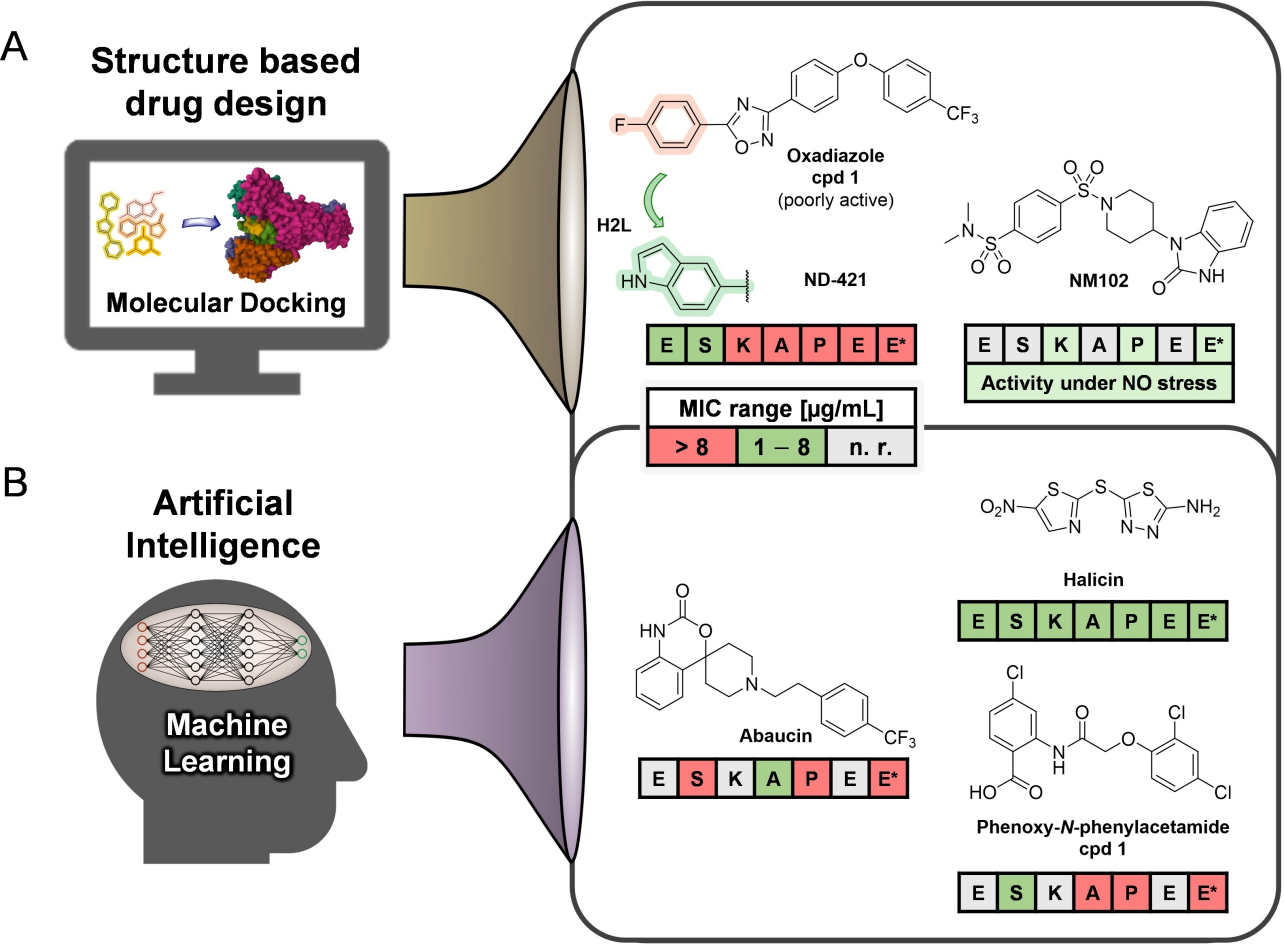
A): Structures of oxadiazole cpd 1, ND‐421 (binder of penicillin‐binding protein PBP2a) and of NM102 (Mfd protein) identified from virtual screening/molecular docking. B): Structures of abaucin (targets periplasmic lipoprotein transport), as well as halicin and phenoxy‐*N*‐phenylacetamide cpd 1 (both membrane depolarizers) from AI‐based approaches. Antibacterial spectrum against the ESKAPEE* panel= *Enterococcus faecium*/*Staphylococcus aureus*/*Klebsiella pneumoniae*/*Acinetobacter baumannii*/*Pseudomonas aeruginosa*/*Enterobacter* sp./Escherichia coli*. MIC=minimal inhibitory concentration. n. r.=not reported.

**Molecular docking** (Figures 5 and 6) can nowadays be used to perform virtual screening and scoring of compound libraries to identify hits, which are then further validated and optimized. Virtual screening of the ZINC database, which contains several millions of compounds, against the penicillin‐binding protein PBP2a of Methicillin‐resistant *Staphylococcus aureus* (MRSA) serves as an example where this approach has been successfully applied.[^94^, ^143^] From the list of virtual hits, **oxadiazole‐derivative cpd 1** (Figure 6)^[94]^ was identified, and due to poor bioactivity subsequently optimized. The resulting candidate compound (**ND‐421**, Figure 6 and Table 1)^[143]^ shows good activity against various *S. aureus/E. faecium* strains and shows encouraging oral bioavailability in a murine peritonitis model (*S. aureus*). Similarly, 4.8 million compounds were assessed in a virtual screening against the ATP‐binding site of a 3D model of *E. coli* Mfd protein. In total 95 molecules were identified of which **NM102** (Figure 6) was the best hit.^[70]^

Advancements in **machine and deep learning** (**ML**) as well as **artificial intelligence** (**AI**) (Figures 5 and 6), also have led to an increase in contributions to identify antibacterial compounds. In one approach,^[96]^ a neural network was trained with a set of 2,335 molecules of which 120 displayed growth inhibition of *E. coli* in order to identify relevant molecular features. The model derived from this approach was then applied to a prediction set from the Drug Repurposing Hub (size ~6,111 molecules) from which 99 molecules were identified of which 51 showed growth inhibition of *E. coli*. After prioritization of hits, **halicin** (MIC=2 μg/ml *E. coli*; Figure 6) was identified, a known c‐Jun N‐terminal kinase inhibitor, which mechanistically acts as a membrane depolarizer. Efficacy was shown in a topical *A. baumannii* wound model of the mouse. Another study of a deep learning approach^[96]^ used ~7,500 molecules as a training set, of which 480 were identified as “active” against *A. baumannii*. From these data, a predictive model was derived and applied to search 6,680 molecules of the Drug Repurposing Hub. A set of 240 molecules met the search criteria, and of these, 9 molecules showed activity against *A. baumannii*. The most potent among these was RS102895 (renamed **abaucin**; Figure 6), a CCR2‐chemokine receptor antagonist with a narrow spectrum activity against *A. baumannii* (MIC=2 μg/ml). The bacterial target of abaucin was suggested to be the inner membrane protein LolE, involved in lipoprotein trafficking, and proof of concept was accomplished in a topical wound infection model.^[144]^ Another deep learning approach used a training set of 39,312 compounds (anti‐staphylococcal activity/cell toxicity) to apply graph neural networks (GNN) to >12 million compounds. After a filtering process, **phenoxy‐*N*‐phenylacetamide cpd 1** (Figure 6) was identified which had anti‐staphylococcal activity (murine infection model) and, like halicin, dissipation of the proton motif force as a MOA.^[145]^

### 2.4 Design Approaches

**Figure 7:**
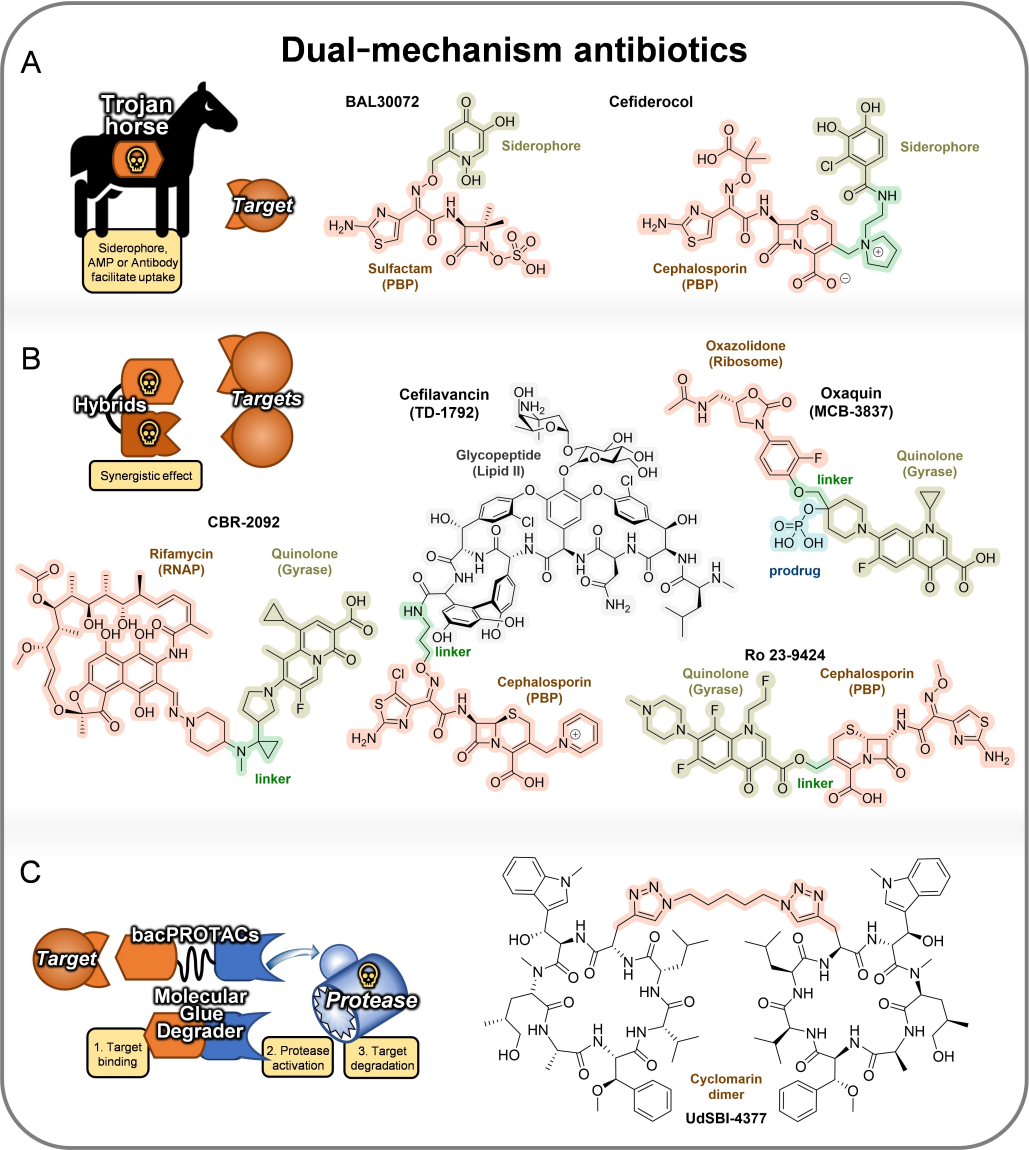
Rational design approaches toward dual‐mechanism antibiotics: A) Trojan‐horse strategy embodied by BAL30072 and cefiderocol, B) hybrid‐type antibacterials embodied by cefilavancin, oxaquin, CBR‐2092 and Ro 23–9424, C) bacPROTAC/molecular glue‐degrader approach embodied by the cyclomarin dimer UdSBI‐4377.

Making use of past experiences and observations, a number of **rational design approaches** have been established (Figure 7). One precondition for many antibacterials is, that they need to reach their target, located in the cytoplasm. An old concept for shuttling molecules is the **trojan‐horse strategy** (Figure 7A), which works by tethering antibacterials to transport molecules. This strategy has been mainly directed against G− bacteria and has been widely explored by the synthesis of conjugate molecules, the so‐called sideromycins.[^146^, ^147^] In this case, the transport molecule is a siderophore, an iron carrier molecule or a partial structure thereof. In fact, nature has already exploited this concept with albomycin (grisein),[^148^, ^149^] which is an aminoacyl‐tRNA synthetase (AaRS) inhibitor.[^150^, ^151^] This MOA has been the template for various synthetic siderophore‐conjugates, permutating the iron‐chelate part together with antibacterials, e.g., fluoroquinolones, glycopeptide antibiotics, oxazolidinones, and daptomycin.[^152^, ^153^, ^154^] The most successful example of this conjugate‐type is a combination of a hydroxypyridone or catecholate in combination with a β‐lactam. A favorable feature is that for G− bacteria the latter only has to reach the periplasm to exert its activity. One example for a hydroxypyridone conjugate is **BAL30072** (Basilea, Switzerland) (Figure 7A) which has been assessed against β‐lactam resistant G− bacteria^[155]^ and for synergistic effects with carbapenems.^[156]^ BAL30072 progressed to clinical phase I (complicated urinary tract infections, (cUTIs), 2014),^[157]^ but hepatotoxicity^[158]^ ultimately stopped further development. A sideromycin, which received drug approval (FDA/EMA), is the parenterally applied **cefiderocol** (cephalosporin/chlorocatecholate‐conjugate, Shionogi, Japan, Figure 7A),^[159]^ for treatment of infections with multi‐resistant (MDR) G− bacteria.[^160^, ^161^]

The coupling of two antibacterials (“**hybrid antibiotics**”, Figure 7B), resembling the above approach also has been known for a long time.^[162]^ Advantages are seen in synergistic effects against resistant organisms and reduced resistance development. However, molecular size increase and an unfavorable match of the partnering antibacterials can significantly alter the antibacterial and also pharmacokinetic profile. **Ro 23–9424** (β‐lactam‐fluoroquinolone), **oxaquin** (MCB‐3837, linezolid‐fluoroquinolone), **CBR‐2092** (TNP‐2092, rifamycin‐fluoroquinolone), and **TD‐1792** (vancomycin‐cephalosporin) are a number of examples (Figure 7B), which have reached clinical phases.^[163]^ Despite some continued research, interest in this approach appears to have declined.

A most recent antibacterial design strategy makes use of **bacterial PROTACs** (Figure 7C),^[164]^ programming target proteins for degradation by the ClpCP unfoldase/protease complex. The peptide cyclomarin A (CymA) is a known mycobacterial ClpC unfoldase binder. In an exploratory approach, protein constructs containing domains of the human protein BRDT in *Mycobacterium smegmatis* were depleted by synthetic bacPROTAC Cym‐JQ1, with JQ1 being a binder of BRTD. In a subsequent study the homo‐BacPROTAC **UdSBI‐4377** (HBPs, cyclomarin dimer, Figure 7C) were used to degrade ClpC1 and ClpC2 and thus to kill *M. tuberculosis*.[^165^, ^166^]

### 2.5 Refining the Scaffold

**Figure 8:**
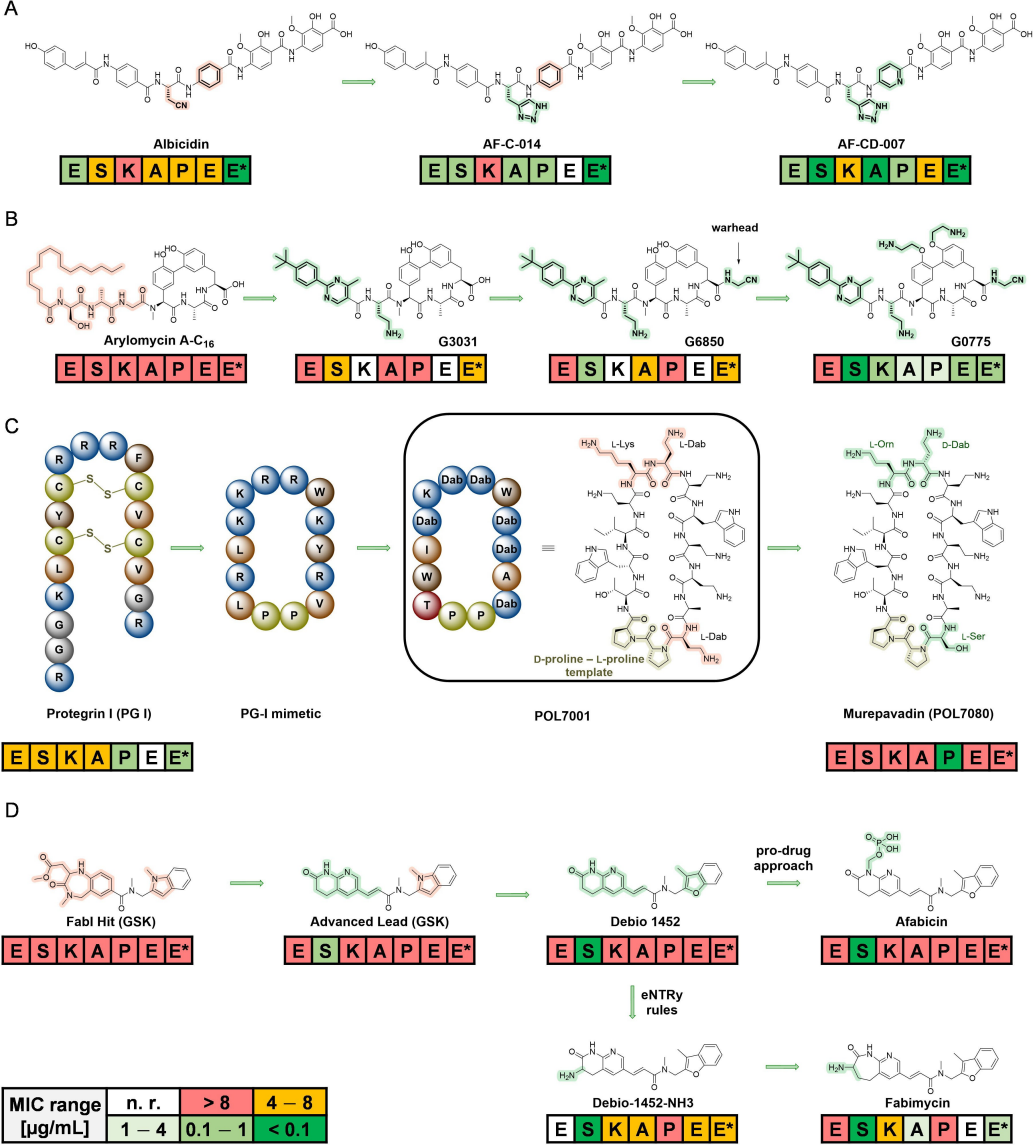
Studies for optimization of antibacterial activity (ESKAPEE* strains): A) Natural product Albicidin→AF‐CD‐007.^[177]^ Enhancement of the antibacterial spectrum and activity by heterocyclic substitutions. B) Natural product Arylomycin→G0775.^[179]^ Engineering of the structure with weak anti‐G+ activity into a compound with anti‐G+ and anti‐G− activity by integrating a C‐terminal warhead and reducing the peptidic character of the molecule. C) Peptide protegrin I (PG‐1)→murepavadin.^[33]^ Evolution of a broad‐spectrum antibacterial PG‐1 peptide via a β‐hairpin‐Pro‐D‐Pro‐template to a head‐to‐tail cyclopeptide with strong and specific anti‐*Pseudomonas* activity. The hemolytic and cytotoxic properties of PG‐1 have been reduced during optimization. D) Small molecule Afabicin→Fabimycin.[^171^, ^195^] Optimization of a weak FabI inhibitor (origin: target‐based screening) via a *S. aureus*‐specific inhibitor (Debio‐1452) to a molecule with anti‐G− activity (Debio‐1452‐NH3 and Fabimycin). ESKAPEE*=*Enterococcus faecium*/*Staphylococcus aureus*/*Klebsiella pneumoniae*/*Acinetobacter baumannii*/*Pseudomonas aeruginosa*/*Enterobacter* sp./Escherichia coli*. MIC=minimal inhibitory concentration. n. r.=not reported.

Once a scaffold structure of interest has been identified, the refining steps aim at understanding structure activity relationships (SAR) and ameliorating the antibacterial spectrum, the pharmacokinetic properties driving pharmacodynamic properties (exposure and efficacy) and the safety profile. A key aspect is the accessibility of the scaffold structure for generating structural diversity of a set of compounds, often comprising hundreds to thousands of analogs. Figure 8 shows examples of refining approaches on natural product, peptide and small molecule scaffolds, leading to either narrowed or broadened antibacterial compound profiles.

Scaffold refinement of **small molecules** lacking a natural product template, e.g., from target‐based screenings, commonly implies short synthesis sequences and ideally a straightforward modular assembly of building blocks. The involvement of state‐of‐the‐art medicinal chemistry design principles, like property‐based fingerprints (e.g. Tanimoto coefficient, Bayesian methods)^[167]^ are part of this process. Ideally, a structure model of the target‐inhibitor complex would be available to aid the process with structure‐based design. An archetypical example is the optimization towards the fatty acid synthesis inhibitor **afabicin** and related structures (Figure 8D).

**Figure 9:**
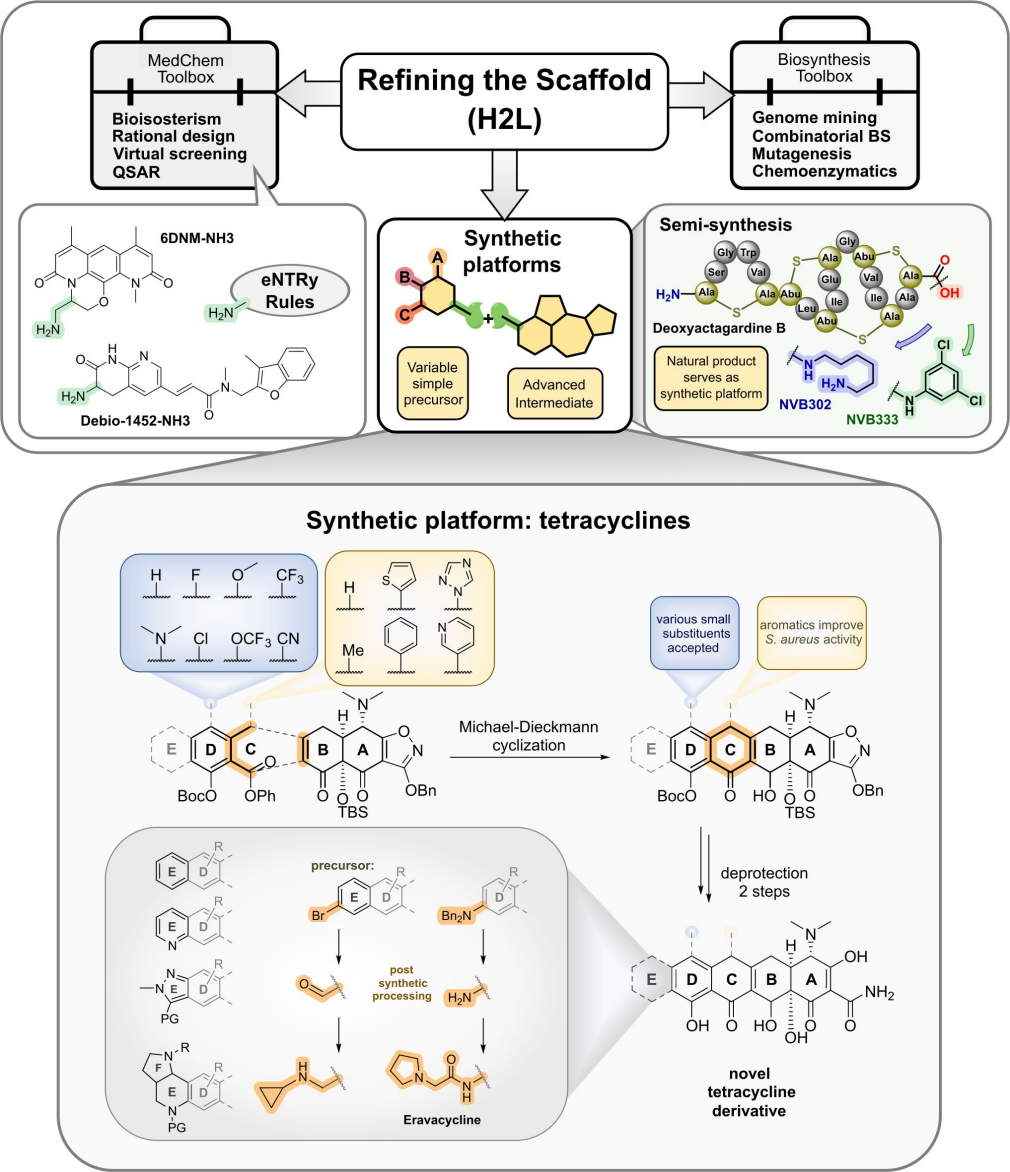
Toolboxes and methods for refining the scaffold applied to small molecules (medchem) and natural products (biosynthesis and medchem). Examples of synthetic platforms for variations and refinement of an antibacterial scaffold structures: semi‐synthesis starting from naturally produced deoxyactagardine B yielding the preclinical candidates NVB302 and NVB333 and total synthesis of tetracycline analogs via a synthetic platform approach.[^183^, ^184^]

From a medicinal chemistry viewpoint, molecular parameters are an integral part to a better understanding and tuning of target accessibility. A comprehensive analysis of anti‐G+ and anti‐G− compounds mapped out basic differences^[168]^ in molecular weight (Ø 813 Da vs. 414 Da respectively), polarity (clogD_7.4_ −0.2 vs. −2.8 respectively), and other parameters pointing to different requirements for the profiles of both types of antibacterials, as well as differences when compared to common drugs from other drug indications. The significance of molecular parameters, such as charge, was known for aminoglycosides and has also been formulated by others.^[169]^ A careful analysis of >180 compounds accumulating in the G− bacterium *E. coli* led to a set of rules termed the “**eNTRy rules**” (Figure 9).^[170]^ These rules are a guide for the transformation of anti‐G+ compounds into anti‐G−. Criteria encompass the placement of an amine functional group on the drug molecule (1°>2°>3° amine), which additionally requires a certain amount of globularity and rigidity. Knowledge of SAR and structural biology data on a drug‐target complex are additional factors guiding the molecular design process. Furthermore, proof‐of‐concept has been provided for a number of compounds, e.g., **Debio‐1452‐NH3**,^[171]^ **6DNM‐NH3** (Figure 9),^[172]^ N‐alkyl guanidinium and pyridinium derivatives.^[173]^ MD simulations point to a lower permeation barrier for molecules bearing a positive charge, which is strongly supported by a favorable alignment of the dipole in the electric field of the transporting porin.^[174]^

The value of a natural product scaffold has spurred the application of organic synthesis to increasingly complex structures and to ultimately establish a **synthetic platform** (Figure 9) for a lead structure. One challenge is to achieve synthetic control over various features, e.g., tuning the antibacterial spectrum and pharmacokinetics, which are not provided by the initial natural product. For structurally simpler peptides (linear/cyclic), e.g. **murepavadin** (derived from **protegrin‐1**; Figure 8C),^[33]^ solid‐phase‐peptide synthesis (SPPS) rapidly provides a considerable number of library members. Examples of peptide‐based structures of higher structural complexity for which **de novo syntheses** have been established include polymyxin‐type compounds (**F365** (QPX9003); anti‐G−) (Figure 23A),^[175]^ **albicidin** (anti‐G+/anti‐G−) (Figure 8A),[^176^, ^177^] **arylomycin**/**G0775** (anti‐G+/anti‐G−) (Figure 8B)[^178^, ^179^] and the maxamycins (anti‐G+) as vancomycin analogs.^[180]^ Further noteworthy examples of synthetic diversification of highly challenging, albeit already established antibacterial structures, are streptogramin,^[181]^ clindamycin (>500 derivatives→iboxamycin),^[182]^ and the polyketide antibiotics tetracycline (>30 derivatives→**eravacycline**) (Figure 9)[^183^, ^184^] and erythromycin (>300 derivatives).^[185]^

For many natural product antibiotics, semi‐synthetic derivatization (partial synthesis; Figure 9) has been the dominating approach in the past. Depending on the complexity of the scaffold, **semi‐synthesis** can range from a basic set of simple modification reactions to elaborate synthesis sequences. However, the semi‐synthesis approach is inherently limited by natural product supply and the sensitivity of the scaffold to side‐reactions by the applied chemistry. Previously, the semi‐synthesis approach has been successfully applied to many established antibacterial drugs, e.g., β‐lactams, and still is used for the modification of synthetically challenging antibiotic scaffolds, e.g., vancomycin‐type glycopeptide antibiotics.^[186]^ For example starting from the lantibiotic **deoxyactagardine B** (Figure 9), which is obtained from large scale fermentation, the preclinical candidates **NVB302** ^[187]^ and **NVB333** ^[188]^ have been synthesized.

Among biology‐based approaches, combinatorial biosynthesis and genome mining have made major contributions. **Combinatorial biosynthesis/synthetic biology** harnesses the modular architecture of mostly non‐ribosomal peptides (NRPs), polyketides (PKs) and ribosomally synthesized and post‐translationally modified peptides (RiPPs) as well as of products of some other pathways. Established heterologous host organisms, e.g., *Streptomyces lividans* for Actinomycetes BGCs, or *E. coli* for many RiPP BGCs, but also many other heterologous host systems, provide a set of molecular biology tools beyond those from the original producer organism.^[189]^ However, in respect of only few examples, e.g., the non‐ribosomal peptide daptomycin,^[190]^ combinatorial biosynthesis requires significant experimental efforts, rendering comparatively low numbers of mutants. For RiPP pathways, e.g., lantibiotics like actagardine,^[187]^ site‐directed mutagenesis of precursor peptides provides a better experimental outcome. Generally, it must be considered that, according to the current state‐of‐the‐art, for some scaffold structures, chemical synthesis is a competitive, if not superior, alternative. **Chemoenzymatics** is feasible even at an experimentally very high level, as impressively demonstrated for fluorinated erythromycins,^[191]^ but in order to maintain compound supplies, scale‐up by alternative methods is required, e.g., chemical synthesis.

**Genome mining** (Figures 5 and 9) is highly valuable for the rapid identification of additional members of a natural product family,^[192]^ which may display better or extended properties. This has been shown by a phylogenetic analysis of metagenomic data for daptomycin‐type Calcium‐dependent antibiotics.^[105]^ The predicted distinct non‐ribosomal BGC was heterologously expressed, yielding the lipopeptide‐type malacidins (structure not shown), which were further characterized. In this line, **retro‐biosynthetic approaches** try to reconstruct biosynthetic pathways from fragmented biosynthesis genes or biosynthetic gene clusters which are not expressed. This approach on non‐ribosomal BGCs of hypothetical cyclopeptide candidate molecules is currently a major focus. Examples are the polymyxin/colistin‐related compounds cilagicin^[193]^ and macolacin (structures not shown),^[194]^ which were chemically synthesized according to bioinformatic predictions and characterized for their antibacterial profile.

## 3 Target Classes of Antibiotic Research

**Figure 10:**
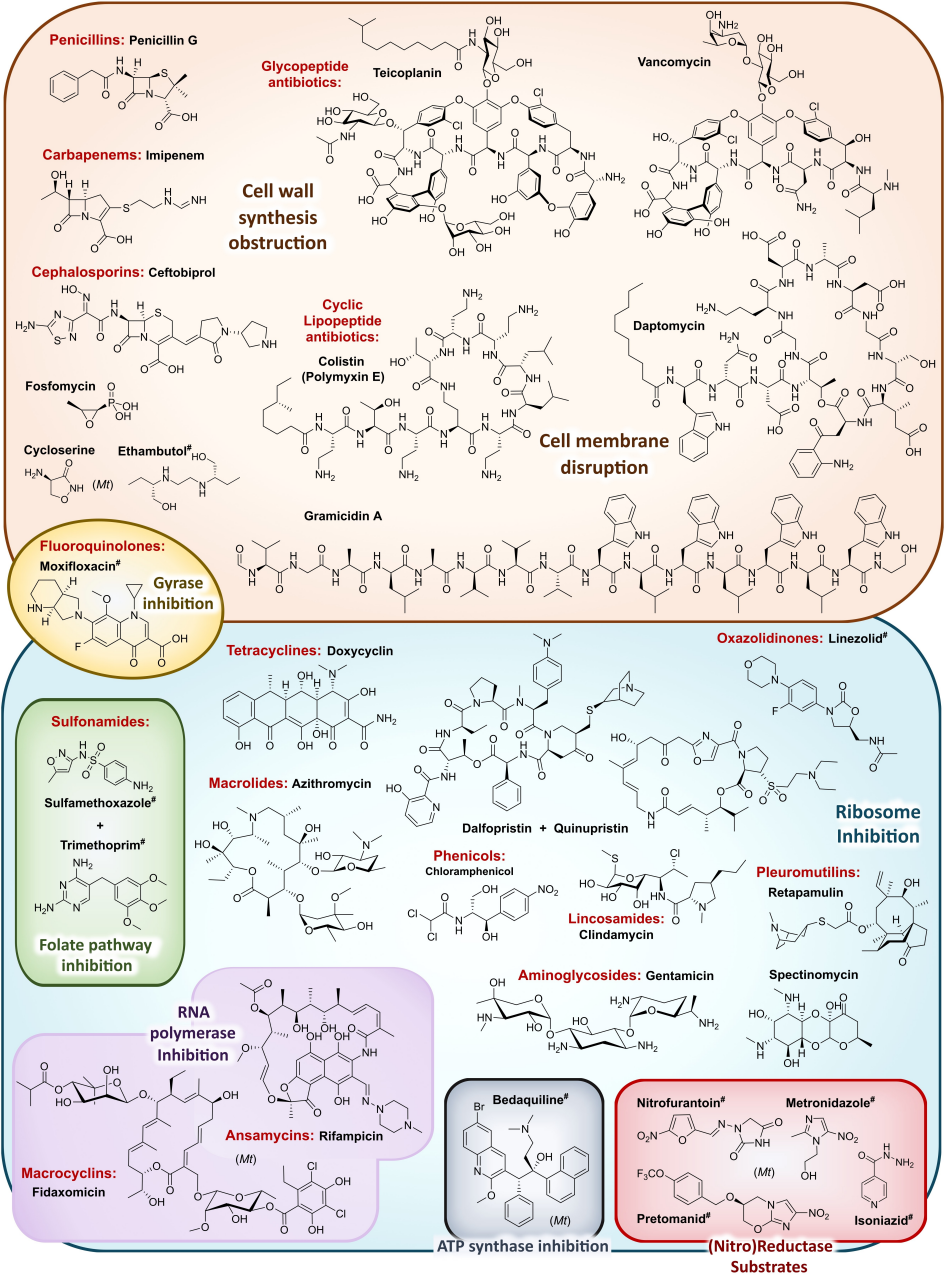
The current defense line of antibacterials in human healthcare. Structures of marketed drugs are shown with one exemplary compound. Among the large drug classes (penicillins, cephalosporins, carbapenems, fluoroquinolones, tetracyclines, macrolides, aminoglycosides, sulfonamides+trimethoprim) antibacterials targeting the cell envelope and protein biosynthesis dominate. More specialized targets are RNA‐polymerase (fidaxomicin/*C. difficile*, rifampicin/mycobacteria) and ATP synthase (bedaquiline/mycobacteria). Nitrofurantoin (broad spectrum), metronidazole (anaerobes, Helicobacter), pretomanid and isoniazid (mycobacteria) are prodrugs activated by nitroreductases and have different mode‐of‐actions. Topical antibacterials have not been considered. Antibiotics originating from synthetic origin are indicated ^#^. The listing makes no claim to completeness.

**Figure 11:**
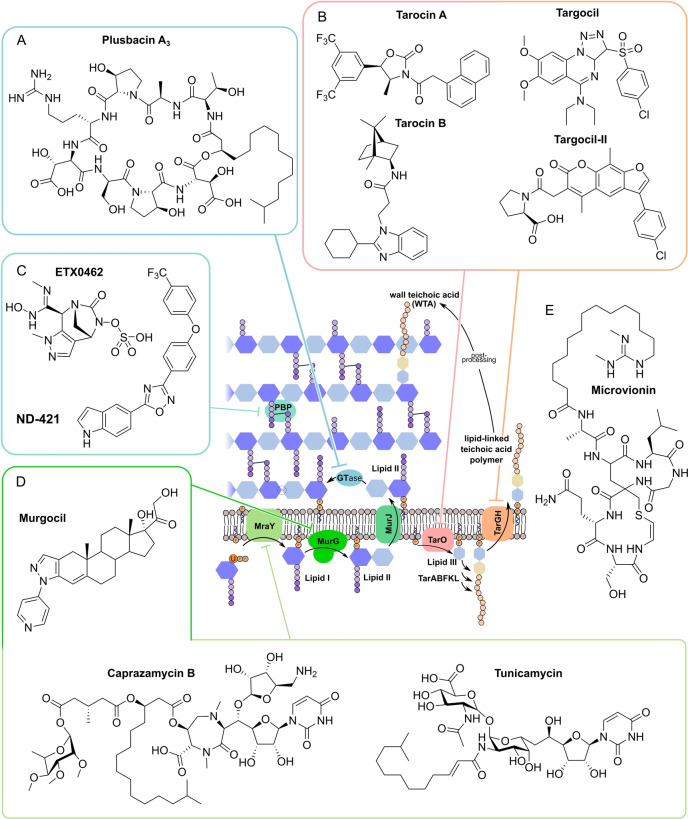
Inhibitors of the bacterial cell wall synthesis. Structures of A) transglycosylation inhibitor plusbacin A_3_ (GTase=glycosyltransferase), B) the TarO and TarGH (wall teichoic acid biosynthesis) inhibitors tarocin A, tarocin B, targocil and targocil‐II, C) the inhibitors of the penicillin binding protein (PBP) ETX‐0462 and ND‐421. D) MraY and MurG (Lipid II biosynthesis) inhibitors murgocil, caprazamycin B and tunicamycin, E) putative Lipid II binder microvionin.

**Figure 12:**
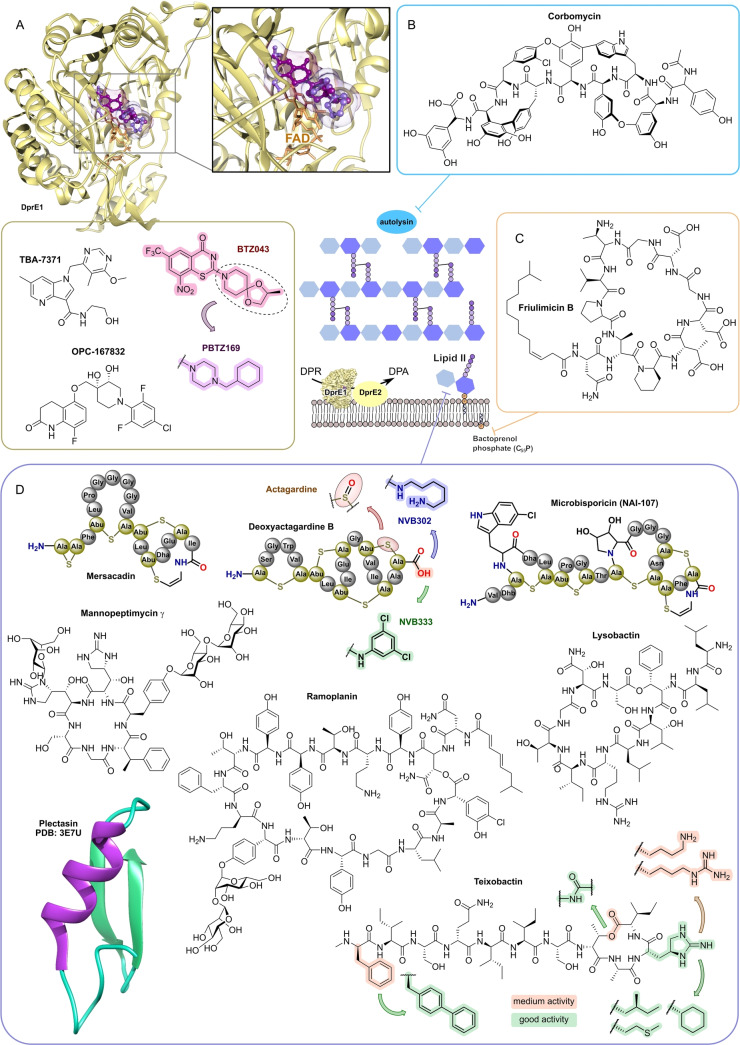
Inhibitors of the bacterial cell wall synthesis. Structures of A) the DprE1 (yellow, mycobacterial arabinan biosynthesis) inhibitors TBA‐7371, OPC‐167832, BTZ043 and PBTZ169, the latter in complex with DprE1 (pink/purple, PDB: 6HEZ, FAD=flavin‐adenine‐dinucleotide), B) the autolysin inhibitor corbomycin, C) the bactoprenol binder friulimicin, and D) various lipid II binders.

**Figure 13:**
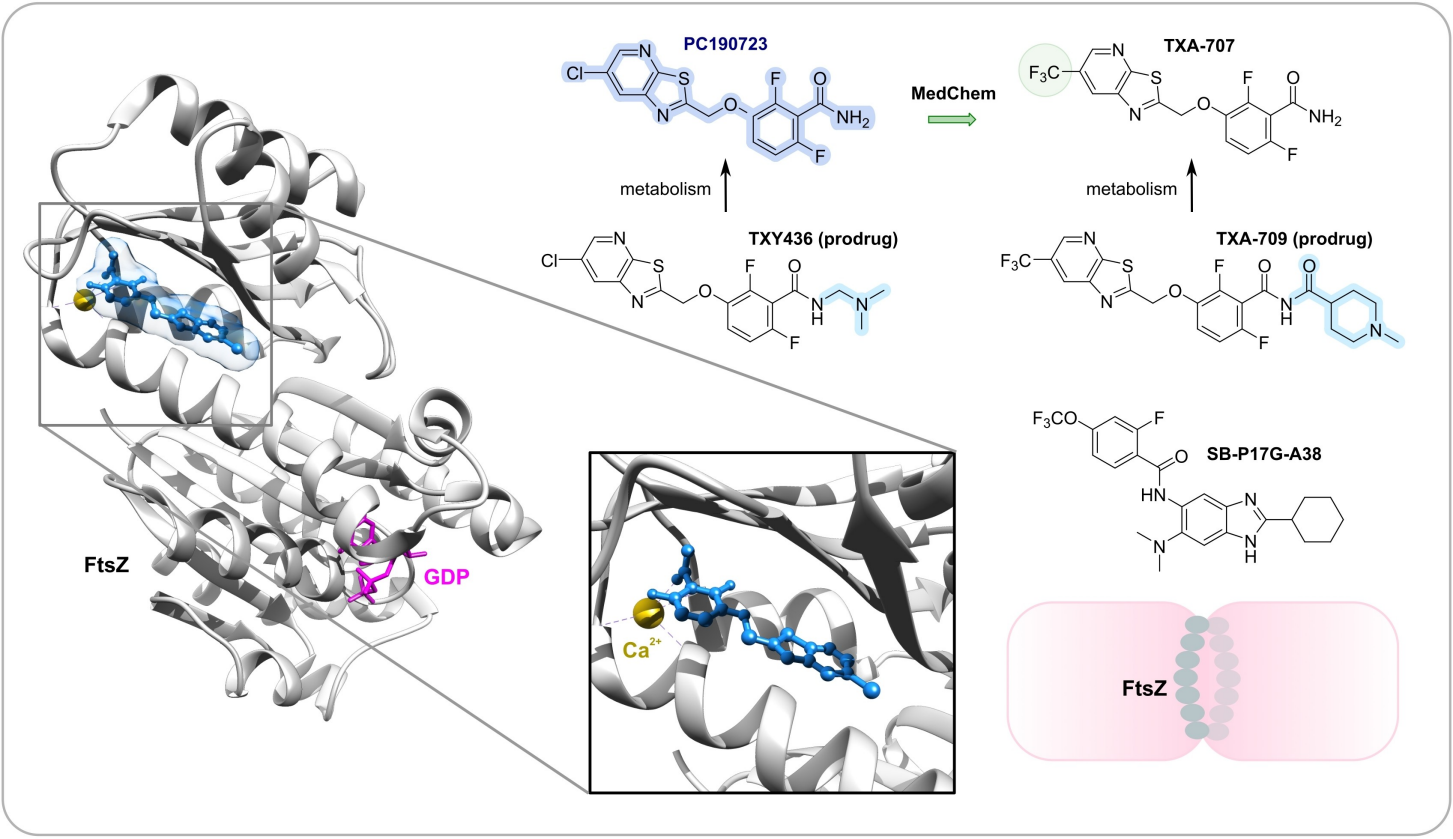
The FtsZ (cell division protein) inhibitors PC190723 (crystal structure PDB: 3VOB, GDP=guanosine diphosphate), with its prodrug TXY436, TXA‐707 with its prodrug TXA‐709 and the antimycobacterial compound SB‐P17G‐A38.

**Figure 14:**
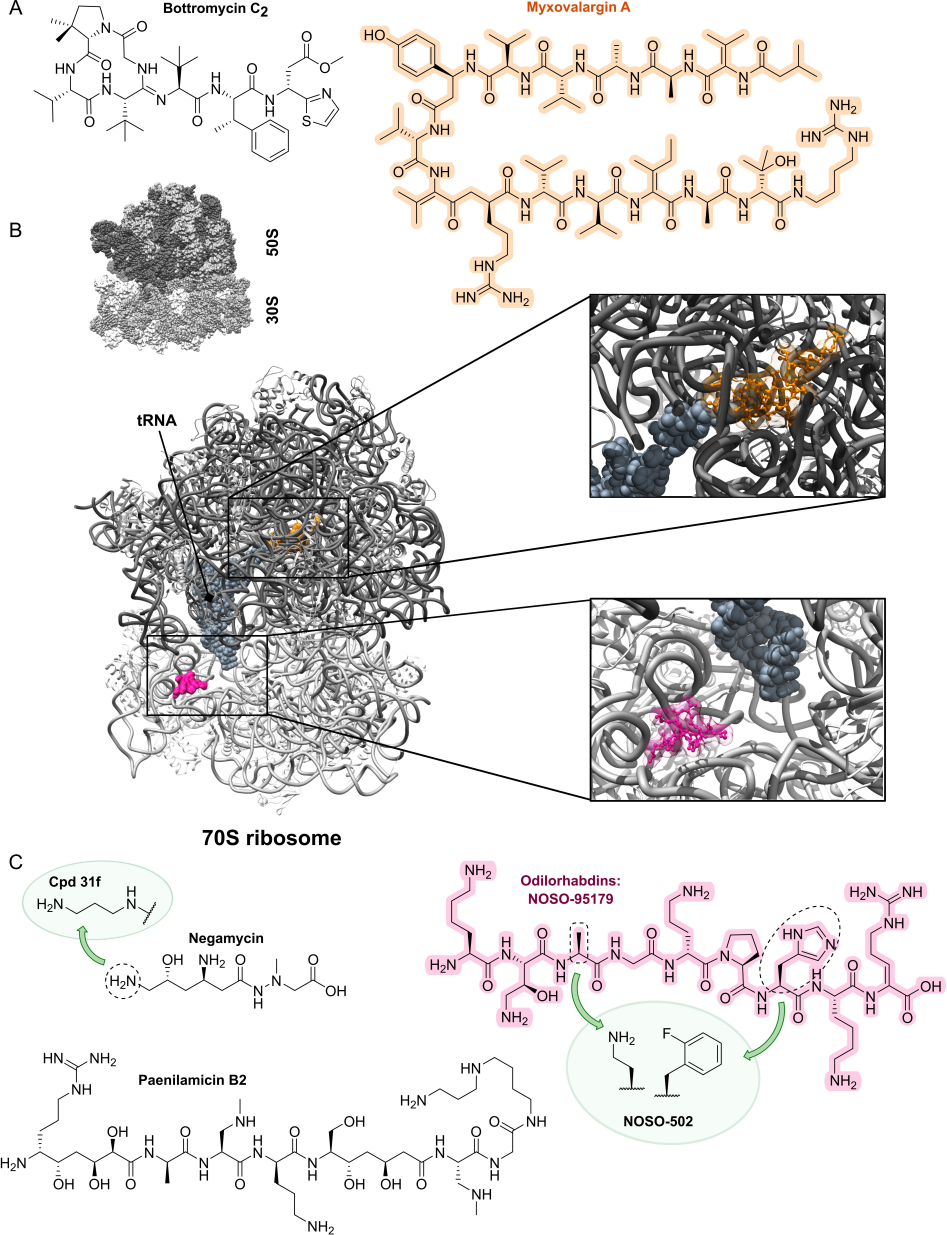
Compounds interfering with ribosomal protein synthesis. A) Structures of bottromycin C_2_ and myxovalargin, B) the 70S ribosome (50S in dark grey and 30S in bright grey, PDB: 8B7Y) in complex with myxovalargin (orange, PDB: 8B7Y) and NOSO‐95179 (pink, PDB: 6CAE) and C) structures of negamycin, negamycin cpd 31f, paenilamicin B2 and odilorhabdins NOSO‐95179 and NOSO‐502.

**Figure 15:**
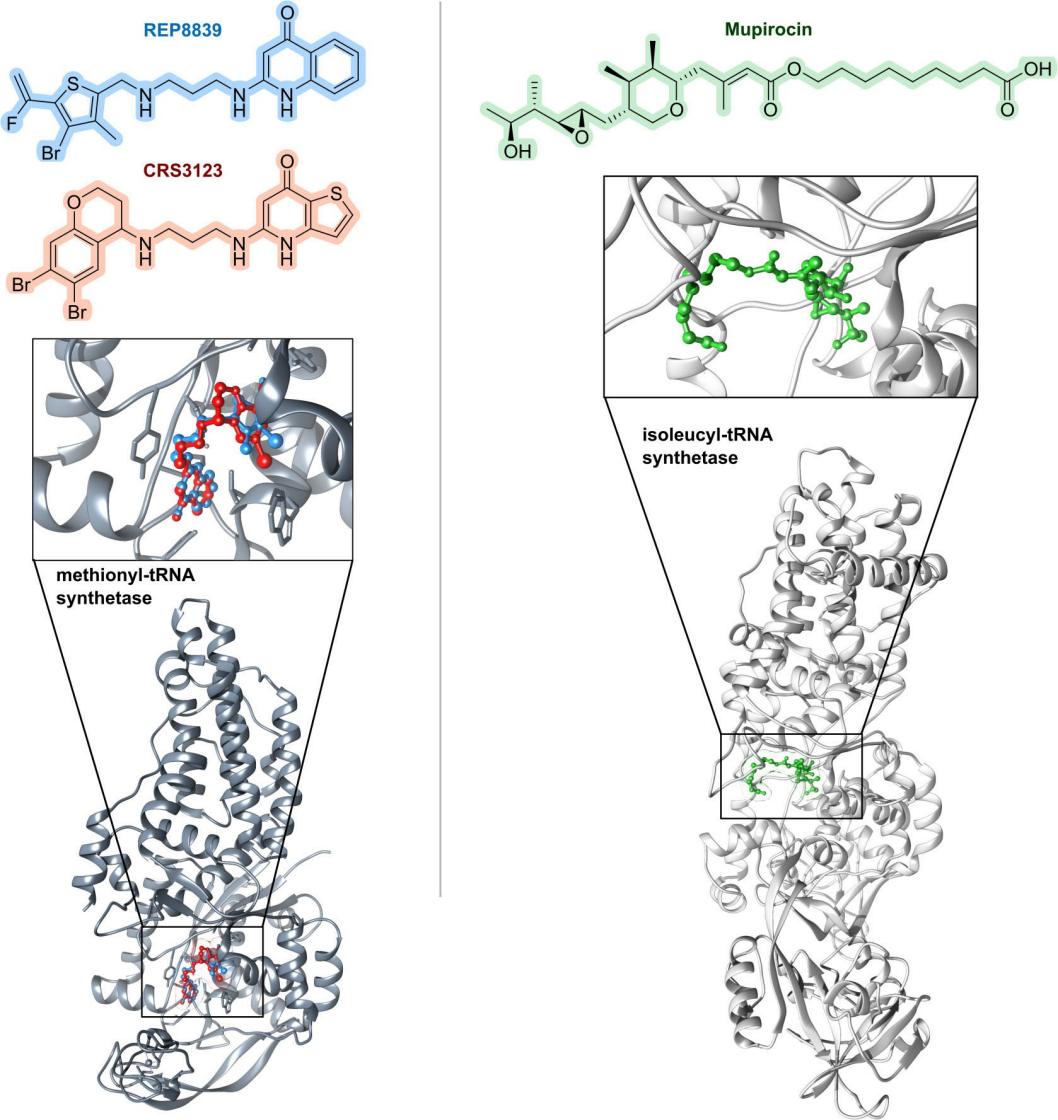
Structures of aminoacyl tRNA synthetase inhibitors REP8839 (blue, PDB: 6WQS) and CRS3123 (salmon, PDB: 6WQT) in complex with methionyl‐tRNA synthetase and mupirocin (green, PDB: 1JZS) in complex with isoleucyl‐tRNA synthetase.

**Figure 16:**
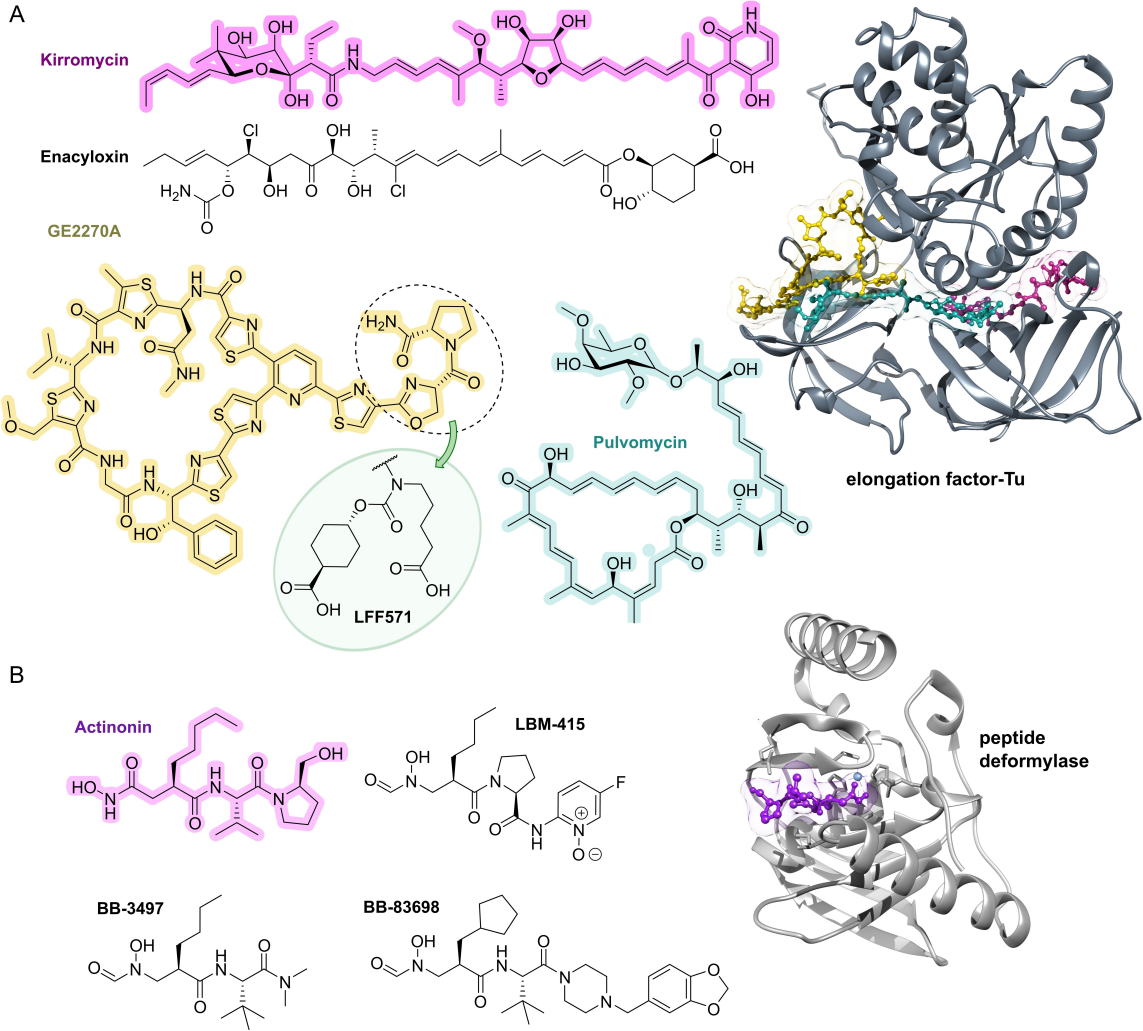
Structures of A) kirromycin (pink, PDB: 1OB2), enacyloxin, GE2270A (yellow, PDB: 2C77), LFF571 and pulvomycin (turquoise, PDB: 2C78) in complex with elongation factor EF‐Tu. B) Structures of actinonin, LBM‐415, BB‐3497, BB‐83698 as well as of actinonin (purple, PDB: 1G2A) in complex with peptide deformylase.

**Figure 17:**
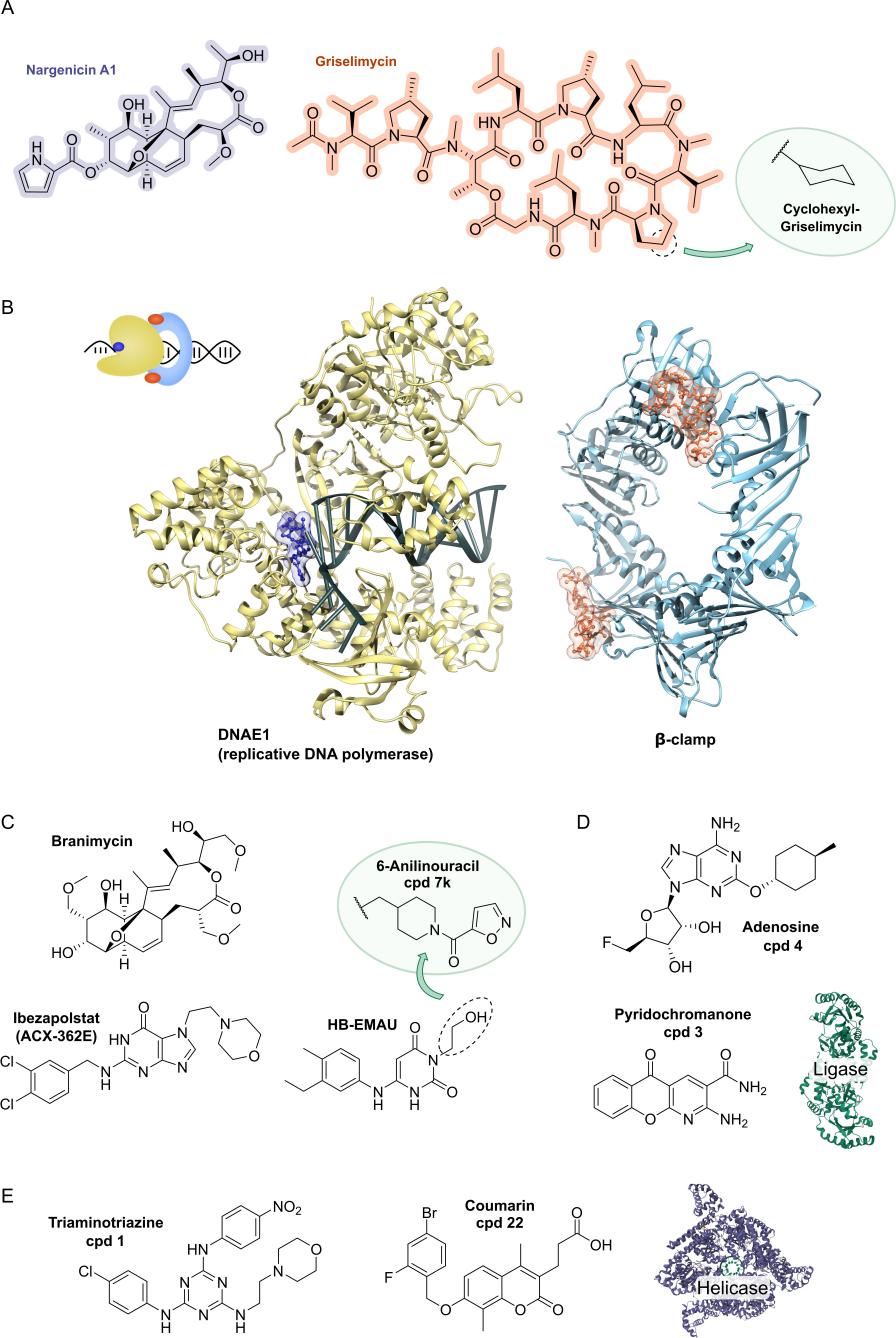
Inhibitors involved in DNA replication. A) Chemical structures of nargenicin A1, griselimycin and cyclohexyl‐griselimycin. B) *M. tuberculosis* DNA polymerase (DnaE1, yellow) in complex with nargenicin (blue, PDB: 7PU7) and *M. smegmatis* sliding clamp (light blue) in complex with griselimycin (pink, PDB: 5AH2). Structures of DNA polymerase inhibitors branimycin, ibezapolstat, HB‐EMAU, anilinouracil cpd 7k, D) of the ligase inhibitors adenosine cpd 4, pyridochromanone cpd 3, and E) of the helicase inhibitors coumarin cpd 22 and triaminotriazine cpd 1.

**Figure 18:**
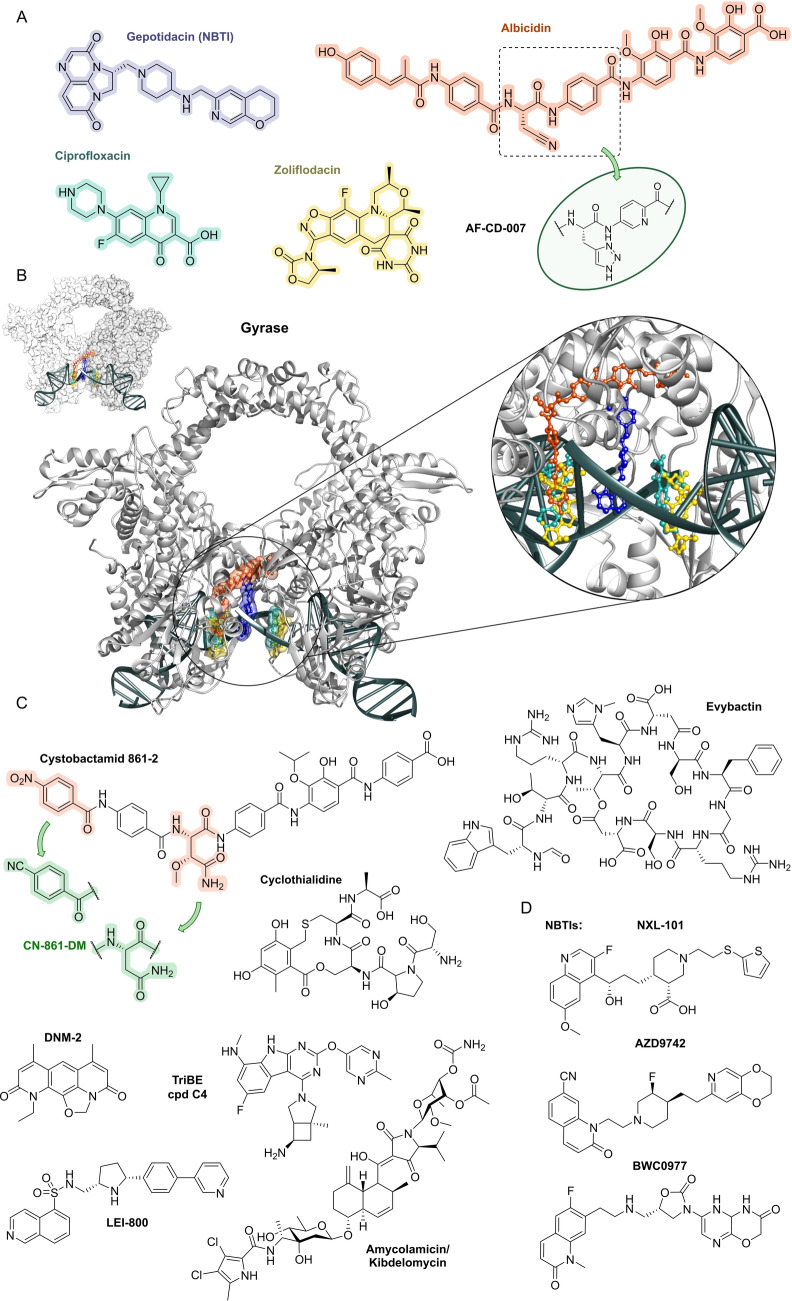
Gyrase and topoisomerase IV inhibitors. A) Chemical structures of gepotidacin, ciprofloxacin, zoliflodacin and albicidin. B) *E. coli* gyrase DNA cleavage complex with albicidin (red, PDB: 7z9c) and superimposed structures of gepotidacin (blue, PDB:6QTP), zoliflodacin (yellow, PDB: 8bp2) and ciprofloxacin (cyan, PDB: 2xct) in complex with *S. aureus* gyrase. C) Structures of further gyrase inhibitors and D) novel bacterial topoisomerase inhibitors (NBTIs) NXL‐101, AZD9742, and BWC0977.

**Figure 19:**
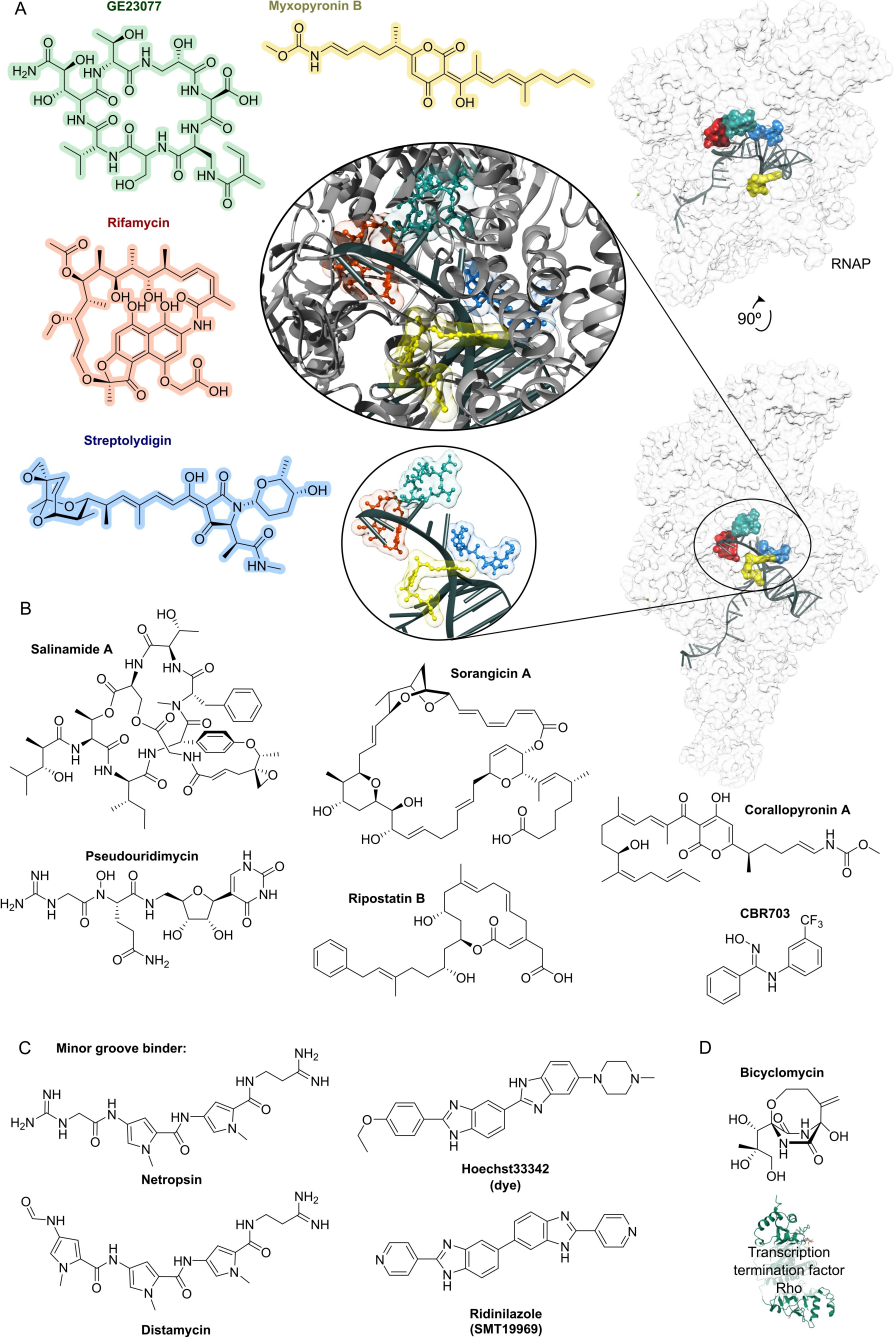
RNA polymerase inhibitors. A) Structures of GE23077 (green, PDB: 4OIR) and rifamycin (red, PDB: 4OIR) in complex with *Thermus thermophilus* RNA polymerase. Streptolydigin (blue, PDB: 1ZYR), myxopyronin (yellow, PDB: 3DXJ) are superimposed at their respective binding sites. B) Structures of further RNA polymerase inhibitors. Structures of C) minor groove binders as predecessors of ridinilazole, and of D) the termination factor Rho binder bicyclomycin.

**Figure 20:**
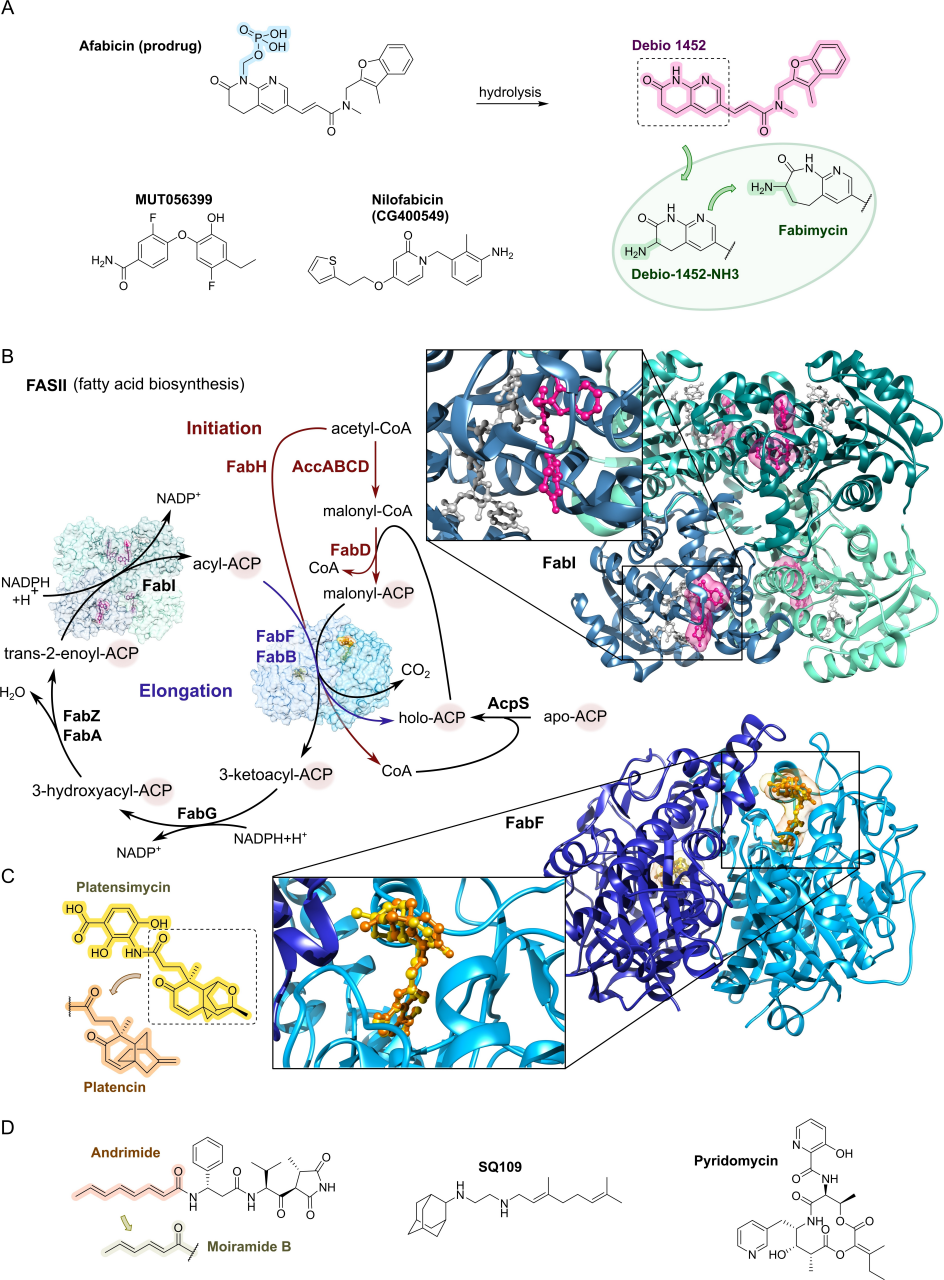
Fatty acid synthesis inhibition. Structures of A) FabI inhibitors, B) schematic FAS II fatty acid synthesis and Debio‐1452 (pink, PDB: 4FS3) in complex with FabI, C) platensimycin (yellow, PDB: 3HNZ) and platencin (orange, PDB: 3HO2) in complex with FabF and D) inhibitors of acetyl‐CoA carboxylase (andrimide/moiramide B), mycobacterial mycolic acid synthesis (SQ109) and fatty acid synthesis (pyridomycin).

**Figure 21:**
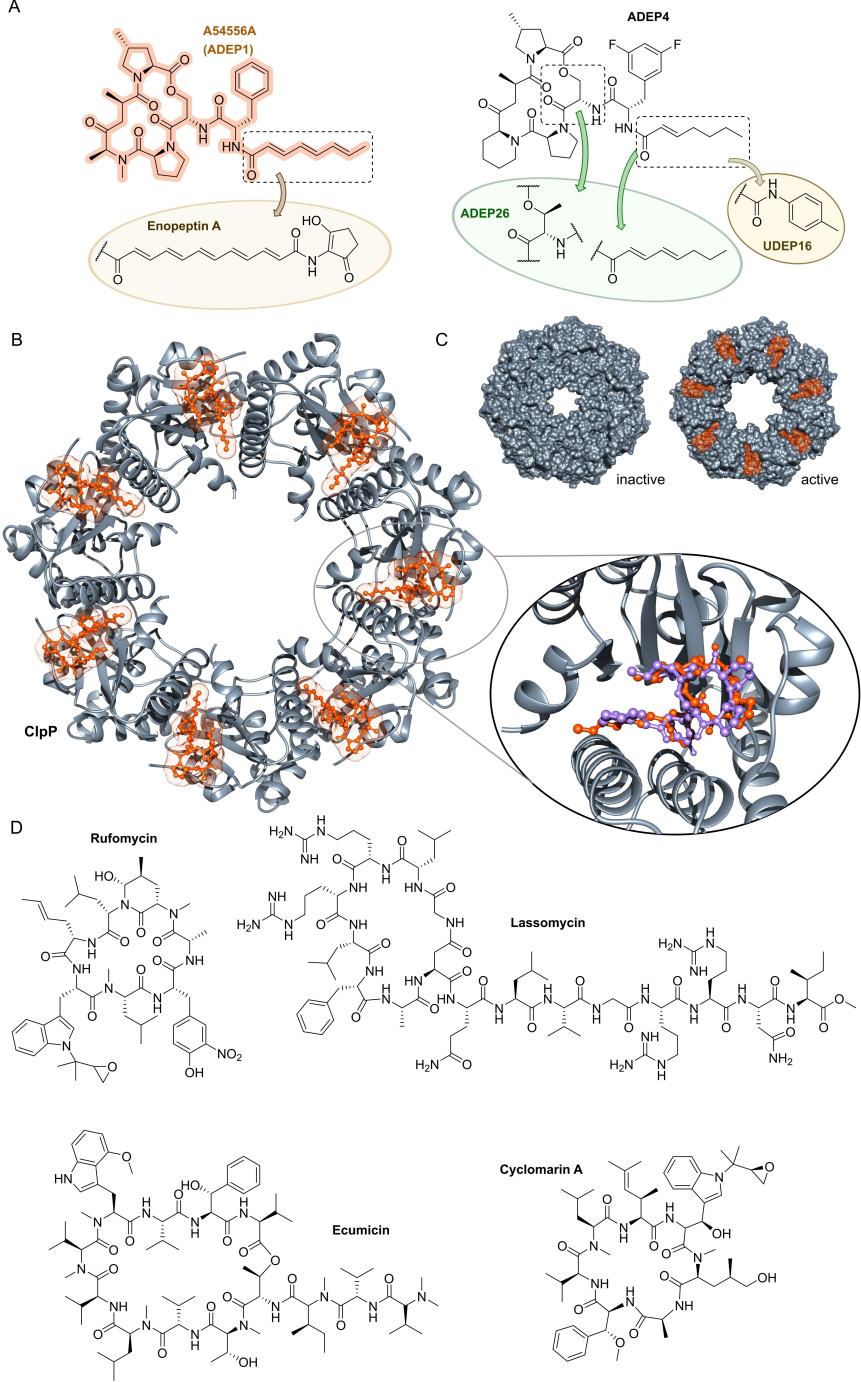
Inhibitors of cytoplasmic and membrane‐bound proteases. Structures of A) cyclic acyldepsipeptides (A54556A, Enopeptin A, ADEP4, ADEP26 and UDEP16), B) of heptameric ClpP in complex with A54556A (orange, PDB: 3KTI). C) Top and side view of inactive/activated ClpP. D) Structures of antimycobacterial ClpC1/ClpX inhibitors.

**Figure 22:**
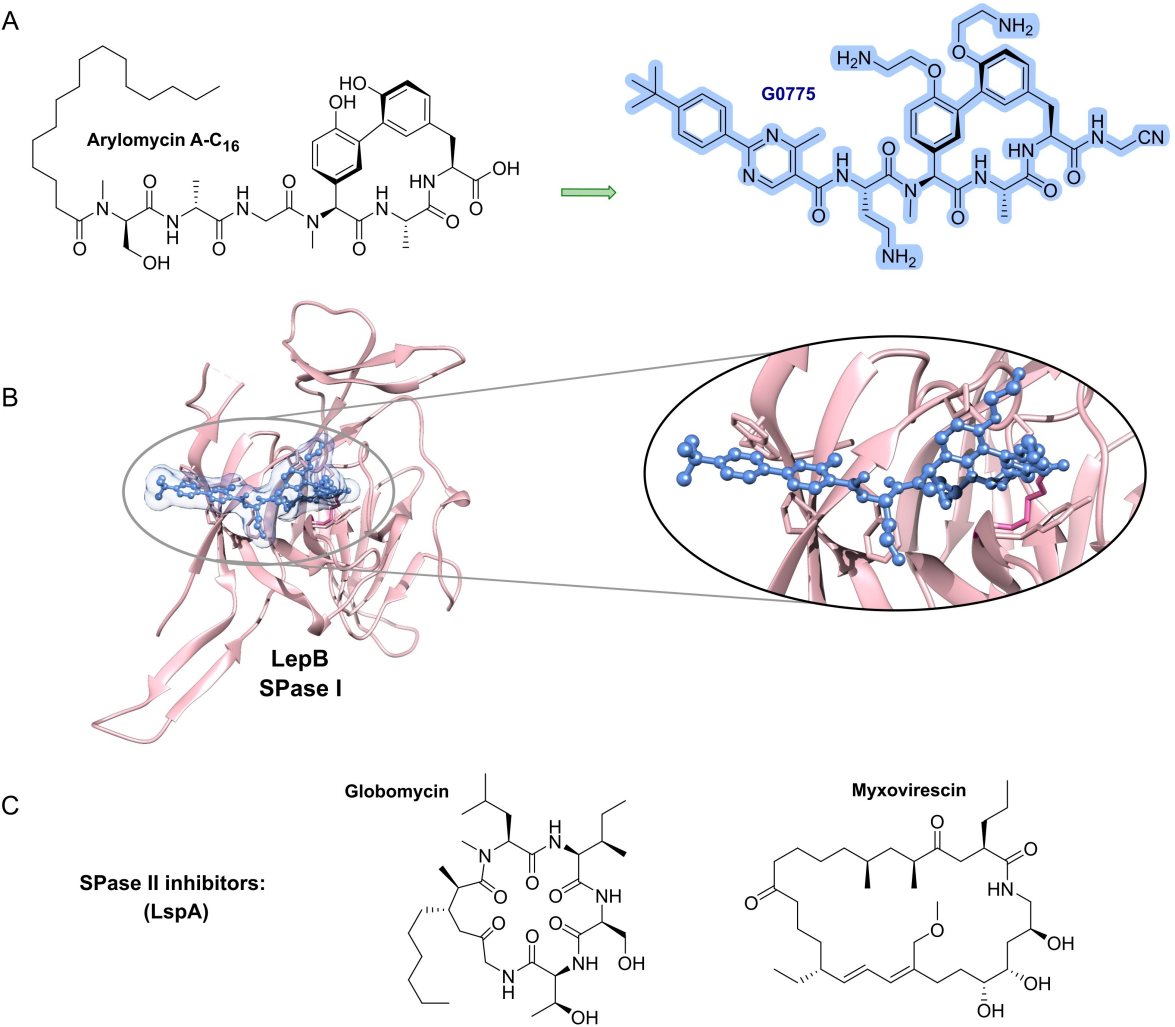
SPase I inhibitors A) arylomycin A‐C_16_ and G0775, B) *E. coli* LepB (salmon) in complex with G0775 (blue, PDB: 6B88). C) SPase II (LspA) inhibitors (globomycin, myxovirescin).

**Figure 23:**
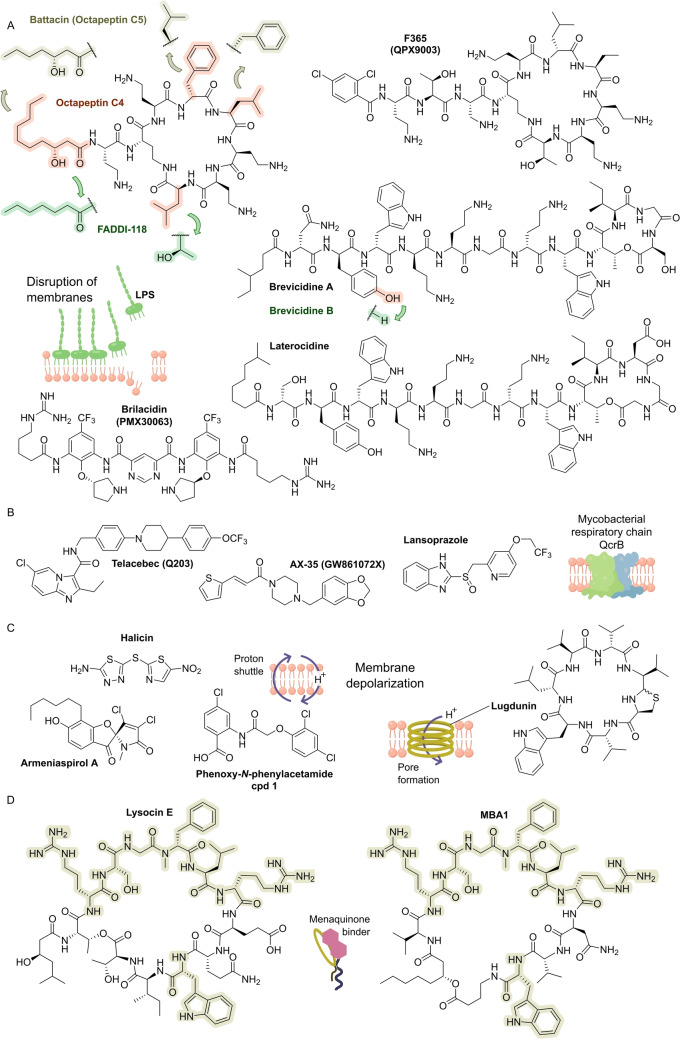
Compounds interacting with membranes and interfering with cellular bioenergetics. Structures of compounds A) binding to membrane lipids, B) affecting factors of the mycobacterial respiratory chain, C) causing membrane depolarization and D) binding to menaquinone.

**Figure 24:**
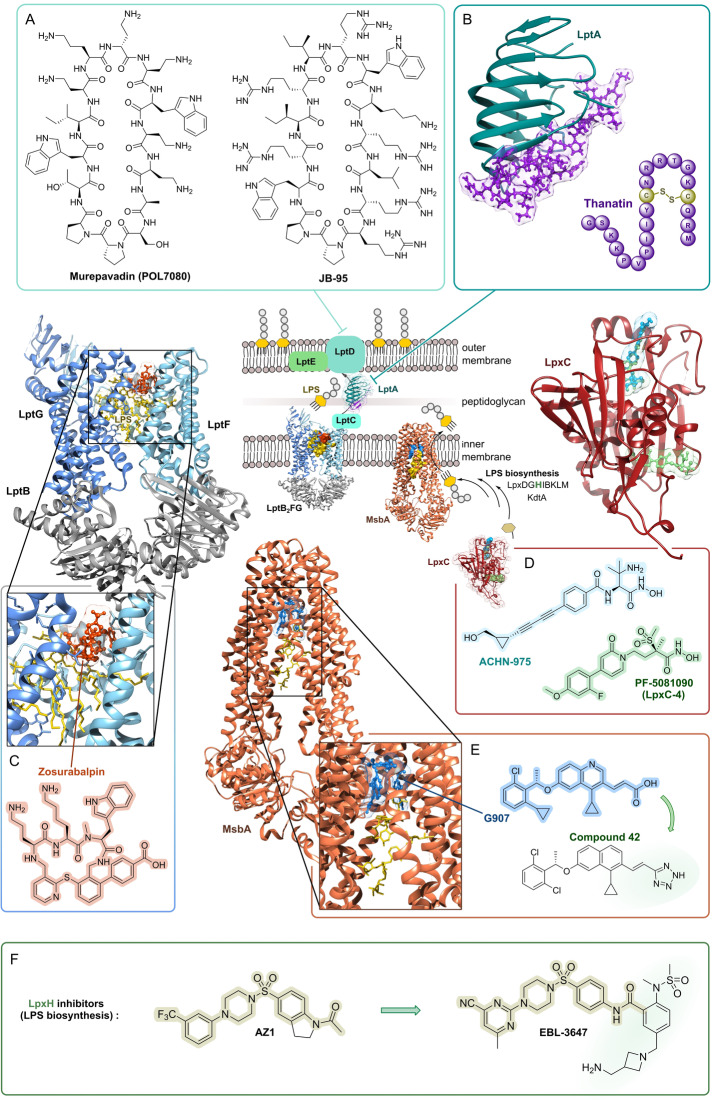
Compounds targeting the lipopolysaccharide (LPS) transport system and lipid A biosynthesis. Structures of A) the LptD inhibitors murepavadin and JB‐95, B) the peptide thanatin (purple, PDB: 6GD5) in complex with *E. coli* LptA (dark cyan), C) the ternary complex of LptB_2_FG (grey, light blue, blue) with LPS (yellow) and zosurabalpin (orange, PDB: 8FRN), D) *P. aeruginosa* UDP‐3‐O‐acyl‐N‐acetylglucosamine deacetylase LpxC (dark red) with ACHN‐975 (light blue, PDB: 6MOO) and PF‐5081090 (light green, PDB: 5UPG), E) the ATP‐dependent lipid A‐core flippase MsbA (red) with the antagonist G907 (blue, PDB: 6BPL) and trapped lipid A fragments (yellow), G) structures of LpxH inhibitors AZ1 and EBL‐3647.

**Figure 25:**
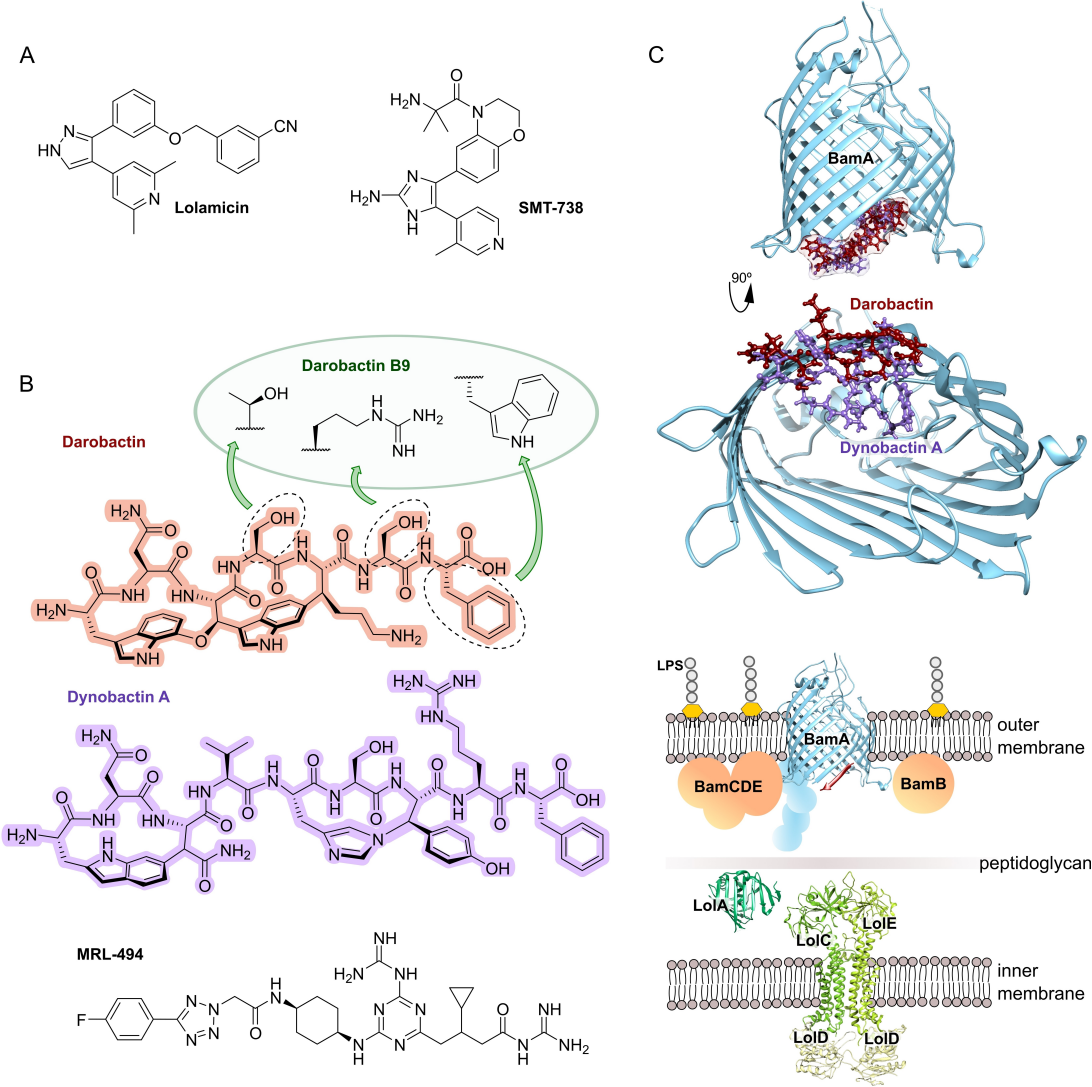
A) Structures of LolC inhibitors lolamicin and SMT‐738 and B) of inhibitors of *E. coli* outer membrane assembly factor BamA darobactin, darobactin B9, dynobactin A and MRL‐494. C) Structures of BamA in complex with superimposed darobactin (dark red) and dynobactin A (purple, PDB: 7R1V) and schematic overview of the Bam complex located in the outer membrane with lipoproteins BamBCDE (orange), BamA (light blue) and darobactin shown in ribbon representation (dark red, PDB: 7NRF). Directly below are structures of the LolCDE complex (PDB: 7MDX) located in the inner membrane and of the periplasmic protein LolA (PDB: 1IWL).

**Figure 26:**
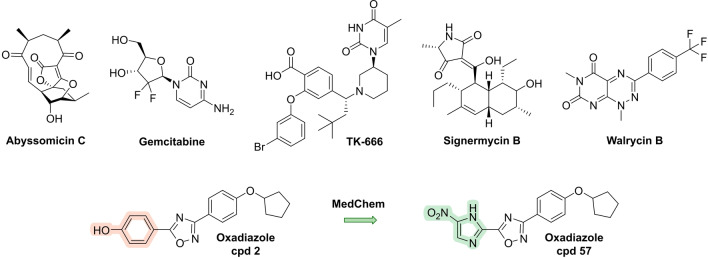
Structures of antibacterials from different target classes: Inhibitors of the folate biosynthesis (atrop‐abyssomicin C) and of the nucleotide biosynthesis (gemcitabine, TK‐666). Inhibitors of two‐component systems (walrycin B, signermycin) and of spore germination (oxadiazole cpd 2 and cpd 57).

The subsequent synopsis categorizes antibacterial target classes according to physiological and biochemical aspects, and highlights selected targets in the context of the drugs addressing them. This overview begins by recalling the chemical structures (Figure 10) and targets of currently available antibiotics used in human healthcare. The following sections include compounds which have reached different stages in profiling and development, including clinical phases (Figures 11 ‐ 26 and Tables 1, 2, 3, 4, 5, 6, 7, 8, 9, 10). An emphasis is laid on aspects of the availability of target‐inhibitor structure models, the antibacterial spectrum (without specifying (multidrug‐)resistant strains), resistance and on compound classes that have shown proof‐of‐concept in animal studies (in vivo).

### 3.1 The Current Medical Defense Lines

Even though it may appear trivial, it is worth considering the chemotype arsenal of drugs currently available for antibacterial therapy (Figure 10).^[26]^ While for large antibacterial classes only exemplary structures are shown, all classes provide highly important life‐saving medications. Some antibacterials are basically standalone structures, e.g., vancomycin/teicoplanin, and some represent extensively explored compound families, e.g., β‐lactams, fluoroquinolones and sulfonamides. Key conclusions of this compilation are that cell‐wall biosynthesis and ribosomal protein synthesis are the main target classes. A particular field is represented by anti‐mycobacterial drugs, which have properties pursuant to the requirements of the pathogen, e.g., inhibition of mycolic acid biosynthesis (e.g. pretomanid).^[196]^ A considerable number of antibacterials are orally bioavailable e.g. penicillins/cephalosporins, tetracyclines, erythromycins and linezolid, which significantly eases applicability over parenteral applications, e.g., vancomycin, daptomycin and others. With regard to their origin, natural product structures dominate over exclusively synthesis‐based drugs (e.g. sulfonamides, quinolones, oxazolidinones). Related to their biosynthetic origins, the natural product‐type compounds are derived from polyketide (PK, type I and II), non‐ribosomal peptide (NRP) and to a lesser extent terpenoid (pleuromutilins) biosynthesis. No RIPP or other ribosomally synthesized peptide is represented. The overall number of different core structures (molecular entities) may astonish and appear surprisingly small.

### 3.2 Peptidoglycan Biosynthesis and Cell Division

Peptidoglycan (PG) biosynthesis (Figure 1)^[197]^ is a broadly established antibacterial target which occurs in three stages: a) cytoplasmic assembly of a lipid I precursor (proteins MurA‐F), b) membrane‐based processing (MraY, MurG) and flipping (MurJ) of lipid II to the peptidoglycan side, and c) subsequent transglycosylation and transpeptidation steps by **transglycosidases** and **penicillin binding proteins** (PBPs). A main requirement of peptidoglycan emerges during cell division, performed by an estimated 30 protein components, termed the divisome.^[198]^ In this process, the protein **FtsZ** plays an essential role as a part of the **cytoskeleton** initiating cell division and septation by assembling the Z‐ring (Figure 1 and Figure 13).^[199]^ Inhibition of PG synthesis leads to destabilization of the cell wall and ultimately to cell lysis.^[200]^ The cell wall of mycobacteria has some peculiarities, e.g., **arabinogalactan** (AG), **lipoarabinomannan** (LAM) and **mycolic acids** (MA) as constituents (Figure 1), for which their biosyntheses are also targets for antimycobacterial drugs.^[27]^

In recent years, only a few contributions report on new impactful inhibitors of early peptidoglycan biosynthesis (MurA‐F).^[201]^ Similarly, with the exception of the MraY inhibitor **caprazamycin B**, which was assessed for anti‐mycobacterial therapy (Figure 11D, Table 1),[^202^, ^203^] uridyl (lipo)nucleoside antibiotics, e.g., the natural products **tunicamycin** (Figure 11D), mureidomycin or pacidamycin,^[204]^ as inhibitors of early peptidoglycan biosynthesis have played only a subordinate role for drug discovery efforts, partially due to a rapid resistance development^[205]^ or toxicity issues.^[204]^ The β‐lactams targeting PBPs (transpeptidases), are not discussed here as they are an established antibacterial class. Examples of non‐β‐lactam‐type inhibitors of transpeptidases are the above mentioned **ETX0462** ^[65]^ (BLI, Figures 3 and 11C, Table 1) and the oxadiazole‐antibiotics like **ND‐421** ^[143]^ (Figures 6 and 11C, Table 1). With regard to transglycosylation inhibition, a wealth of experimental semi‐synthetic modified vancomycin exist, which however, only address multi‐resistant G+ bacteria.^[186]^ An example for a phenotypic screening synergizing β‐lactams (imipenem) is the steroid‐type **murgocil** (Figure 11D),^[206]^ which targets the staphylococcal transglycosylase MurG.

A big portion of the research on inhibitors of peptidoglycan assembly has been performed following the increase of Methicillin‐resistant *Staphylococcus aureus* (MRSA) infections in the 1980ies and 1990ies.^[1]^ It is worthwhile to review a selection of natural product peptide antibiotics (Figure 12D and Table 1) that have been investigated for their mostly anti‐G+ antibacterial performance in vitro and in vivo. The bactericidal lipoglycodepsipeptide **ramoplanin** (discovered by Gruppo Lepetit Gerenzano, Figure 12D and Table 1)[^207^, ^208^] even reached clinical phases.^[209]^ The **mannopeptimycins** are glycocyclopeptides isolated from *Streptomyces hygroscopicus* by a team from Wyeth Research (Figure 12D and Table 1).^[210]^ Due to their activity against clinically relevant G+ bacteria, for a while they were intermediately reconsidered as antibacterials. The MOA is primarily lipid II binding and inhibition of transglycosylation.^[211]^ **Plusbacins** (Figure 11A and Table 1) are lipocyclo‐octadepsipeptides isolated from *Pseudomonas sp*.[^212^, ^213^] with strong activity against G+ bacteria (*S. aureus*, *Enterococci*).^[214]^ Studies on the MOA^[215]^ suggest a dual mode^[216]^ by inhibition of transglycosylation and probably by membrane disruption. The structurally identical cyclopeptides **lysobactin/katanosin B** (Figure 12D and Table 1) have been independently isolated from *Lysobacter* ^[217]^ and from *Cytophaga*.[^218^, ^219^] The MOA of the bactericidal lysobactin is a 1 : 1 complex formation with lipid II.^[220]^ While the total synthesis is known,[^221^, ^222^] detailed SAR studies are not available.

Unlike the above mentioned non‐ribosomally synthesized peptides (NRPs),^[101]^ **actagardin** (Figure 12D and Table 1) is a ribosomally synthesized (RiPP) lantibiotic. The 19mer peptide[^223^, ^224^, ^225^] is an inhibitor of transglycosylation by binding to lipid II.^[226]^ It has moderate activity against *Clostridium* and mostly low activity against other G+ bacteria, whereas C‐terminal amide derivatives^[227]^ showed enhanced antibacterial activity. A considerable amount of structural engineering was performed in the producer strain *A. garbadinensis (*Novacta Biosystems Ltd, UK).[^187^, ^228^] As a result, 228 variants were generated resulting in one mutant with improved activity (G+ bacteria including *C. difficile*). A semi‐synthetic C‐terminal amide modified **NVB302** (7‐aminoheptylamido‐deoxyactagardine, Figure 12D and Table 1)[^187^, ^229^] is efficacious in a hamster infection model (*C. difficile*), and showed no toxicity in rat and dog upon oral uptake. It entered phase I clinical trials in 2011.^[187]^ Another C‐terminally modified deoxy‐actagardin analog is **NVB333** (Figure 12D and Table 1)^[188]^ which has a broader anti‐G+ profile. The case of actagardin stands representative for other lantibiotics, e.g., the distantly related **mersacidin** ^[230]^ or **microbisporicin** (**NAI‐107**, Figure 12D and Table 1),^[231]^ which also underwent assessment programs.

At the time of its discovery, the fungal defensin **plectasin** (Figure 12D and Table 1) was an interesting new compound identified from the ascomycete (fungus) *Pseudoplectania nigrella*.^[232]^ This ribosomally synthesized 40mer peptide with three disulfide bonds was identified from cDNA screening in *E. coli*. The antibacterial activity was strong against various *S. pneumoniae* strains but only moderate against *S. aureus*. Investigations by NMR spectroscopy revealed a 1 : 1 complex of plectasin with lipid II.^[233]^ The lack of cytotoxicity or haemolysis spurred an assessment by an MTD (maximum tolerated dose) study and in murine infection models. The improved variant of plectasin NZ2114^[234]^ showed activity against S*. aureus* and was also characterized in murine infection models.

The report on the 11mer cyclodepsipeptide **teixobactin** (Figure 12D and Table 1) in 2015 gained enormous attention,^[235]^ as the authors could describe the entire research process from the molecule's discovery from *Eleftheria terrae* (G− β‐proteobacterium) to animal studies in mice. Crucial was the use of a multichannel device, the so‐called iChip10,[^117^, ^235^] that enabled simultaneously the isolation and growth of previous uncultured bacteria. Teixobactin's antibacterial activity is directed against various G+ bacteria but it is basically ineffective against most G− bacteria.^[235]^ Teixobactin was found to bind to lipid II, including the wall‐teichoic acid precursor lipid III.^[236]^ The latter does contribute to the liberation of autolysins supporting the antibacterial action. Solution and solid‐state NMR spectroscopic studies imply that the MOA is more complex than anticipated.[^237^, ^238^] The chemical synthesis[^239^, ^240^] is relatively uncomplicated and the considerable number of several hundred derivatives have been synthesized providing a good understanding of SAR.^[241]^

More recent screening of bacterial strain collections and genome mining efforts have unveiled new chemotypes such as **corbomycin** (*Streptomyces sp*., Figure 12B and Table 1), a type V family member of glycopeptide antibiotics (GPAs). It was discovered from mining BGCs of glycopeptide antibiotics lacking known self‐resistant genes.^[242]^ It primarily shows activity against G+ bacteria, e.g., MRSA, drug‐resistant *S. aureus* (DRSA), VRE, and was found to be effective in a topical MRSA mouse model.^[242]^ Corbomycin, like the structurally related complestatin, binds peptidoglycan, blocking its access to autolysins thus inhibiting their action.^[242]^ Autolysins are a highly diverse group of essential peptidoglycan hydrolases involved in remodeling of the cell wall during cell growth.^[243]^ The lanthipeptide **microvionin** ^[244]^ (*Microbacterium arborescens*, Figure 11E and Table 1) has shown a pronounced activity against G+ bacteria. Characteristic structural features are the N‐terminal, bismethylated guanidine fatty acid and the unusual quaternary amino acid avionin. The mechanism of action is currently under investigation, but it is likely that cell wall synthesis is the target.

Among the factors involved in cell division, the **GTPase FtsZ** (Figures 1 and 13) has received the biggest attention in the search for inhibitors. The small molecules **PC190723**, **TXA‐707** (prodrug **TXA‐709**) (Figure 13 and Table 1), which all share a similar molecular architecture, have anti‐staphylococcal activity. Inhibition of an efflux pump by PAβN (Figure 3B) could sensitize *E. coli*, *A. baumannii* and *K. pneumoniae* to **TXY436** (prodrug of PC190723) (Figure 13 and Table 1).^[245]^ Benzimidazole analog **SB‐P17G‐A38** (Figure 13 and Table 1)^[246]^ is a mycobacterial FtsZ inhibitor. However, general challenges for development of effective FtsZ inhibitors are the lack of broad‐spectrum activity, the requirement for membrane permeation, and rapid development of resistance.^[247]^

Further cell wall characteristics of many G+ bacteria are **wall teichoic acids** (**WTAs**),[^248^, ^249^, ^250^] tethered to peptidoglycan and **lipoteichoic acids** (**LTAs**),^[251]^ which are inserted into the membrane (Figures 1 and 11). WTAs and LTAs are important for cell physiology but some pathway inhibitors only attenuate virulence.[^252^, ^253^] WTAs are assembled mostly by Tar (teichoic acid ribitol) and Dlt proteins.[^249^, ^250^] WTA synthesis inhibitors are **targocil**[^254^, ^255^] and **targocil‐II** (both translocase TarGH, Figure 11B),^[256]^ as well as synergistically acting **tarocin A/B** (TarO) (Figure 11B).^[257]^ The last two were also reported as β‐lactam sensitizers for MRSA therapy.^[257]^ For LTA biosynthesis impacting cell division (FtsZ, autolysins), attempts were made to identify small molecule inhibitors of LTA synthase (LtaS),^[258]^ which also act as β‐lactam potentiators,^[259]^ but these studies, which included mouse infection models, were only of a preliminary character.

Essential factors for the biosynthesis of **mycobacterial arabinogalactan** are the membrane‐bound enzymes decaprenylphosphoryl‐β‐d‐ribose‐2’‐oxidase (DprE1) and ‐epimerase (DprE2; Figure 1). These proteins are targeted by various small molecule drugs, such as **BTZ043**, **PBTZ169** (Macozinone), **TBA‐7371**, **OPC‐167832** (all DprE1, Figure 12A and Table 1), and **pretomanid** (prodrug, DprE2, approved 2019, Figure 10) which are currently in various clinical phases.^[260]^

**Conclusions**. Apart from intense research efforts on β‐lactams and non‐β‐lactams as inhibitors of transpeptidases (PBPs) and lactamases, which still continue to make high impacting contributions, research in the 1990ies to early 2000s had a strong emphasis on PG biosynthesis inhibitors with lipid II as a target, however, which led to molecules with predominantly anti‐G+ properties. Cyclic non‐ribosomal peptides are probably the biggest group of inhibitors, but significant efforts were also undertaken to profile lantibiotics and defensins. For the latter two compound types site‐directed mutagenesis in heterologous host strains and semi‐synthesis were the predominant methods to obtain structural variants. The focus on peptides mostly acting as lipid II binders, required parenteral administrations and thus, with the exception of *C. difficile* infections, oral administrations are excluded. Antimycobacterial compounds can also target lipid II and FtsZ, with an additional focus on arabinogalactane synthesis (DprE1/E2). The general lack of potent inhibitors of early peptidoglycan synthesis enzymes (MurA‐F) can be basically explained by the restriction to small molecules, which additionally need to penetrate membranes and the risk of rapid development of resistance. This similarly also applies to inhibitors of the cell division protein FtsZ. Interestingly, with the exception of above mentioned BLIs and the antimycobacterial pretomanid, despite all these efforts, no new compound has yet been approved as a drug.

### 3.3 Ribosomal Protein Synthesis

The **ribosome** is highly complex biosynthetic machinery consisting of rRNA and proteins, translating mRNA into the synthesized protein. The underlying processes include amino acid activation and charging of tRNA by **aminoacyl‐tRNA synthetases** (**aaRSs**), delivery to the ribosome (**elongation factors, EFs**) as well as ribosomal protein synthesis (initiation, elongation and termination phase) (Figure 1).[^261^, ^262^, ^263^] The evolutionary difference between bacterial and eukaryotic protein synthesis is sufficiently distinct to provide a therapeutic advantage, although this has to be assessed individually, since some ribosomal inhibitors, e.g. blasticidin S, show cytotoxicity.^[264]^ To date, a vast number of molecules exist that interfere either directly with the ribosome or with its adjacent factors.^[262]^

It has been suggested that the cyclic heptapeptide **bottromycin** (Figure 14A and Table 2) binds to the acceptor‐site of the 50S ribosome.[^265^, ^266^] Although already described decades ago,[^267^, ^268^, ^269^] the compound has re‐gained interest and this has been accompanied by assessment of semi‐synthetic and synthetic analogs.[^270^, ^271^, ^272^] Issues of stability for potential in vivo applications could be addressed,^[272]^ but antibacterial activities are restricted to G+ bacteria. Similarly, the pseudodipeptide **negamycin** (Figure 14C and Table 2) is a long‐known ribosome inhibitor. It has anti‐G− activity and the availability of synthetic analogs has allowed SAR studies to be reinvestigated,[^273^, ^274^, ^275^] e.g., **negamycin cpd 31 f** (Figure 14C) emerged as an even more potent derivative. The **odilorhabdin NOSO‐95179** (Figure 14C and Table 2)^[276]^ is an analog of a linear polycationic peptide isolated from a phenotypic screening of *Xenorhabdus* strains. It induces amino acid misincorporation into the ribosome. NOSO‐95179 and the synthetic analog **NOSO‐502** (Figure 14C and Table 2) display activity against some G+ and G− bacteria, and, in animal studies, showed efficacy against G− bacteria (*Ec*/*Kp*). With their polycationic structures they resemble the **paenilamicins** (Figure 14C and Table 2),[^277^, ^278^] metabolites from the bee pathogen *Paenibacillus larvae* as well as the myxobacterial **myxovalargin** (Figure 14A and Table 2)^[279]^ which are all targeting the ribosome of G+ bacteria.^[277]^

The natural product **mupirocin** (Figure 15) is the oldest used **amino acyl‐tRNA synthetase** inhibitor, which is only used for topical infections with G+ *S. aureus* and *S. pyogenes*.^[280]^ Two more recent examples from target‐based HTS are the methionyl‐tRNA synthetase (MetS) inhibitors **REP8839** (Figure 15 and Table 2)[^281^, ^282^] for topical applications (anti‐G+; discontinued after clinical phase I), and its analog **CRS3123** (Figure 15 and Table 2), which reached clinical phases for treatment of *C. difficile* infections.[^283^, ^284^]

The compound **LFF571** (Novartis) (Figure 16A and Table 2)^[285]^ is a semi‐synthetic analog of the thiopeptide antibiotic **GE2270A** (Figure 16A and Table 2) which targets **elongation factor EF‐Tu**. Chemical modifications by structure‐guided design were aimed at improving aqueous solubility. LFF571 displays very good activity against G+ bacteria and reached clinical phase II for treatment of *C. difficile* infections, but further development has been discontinued.^[286]^ Other natural product inhibitors targeting EF‐Tu have been described, e.g., long‐known **pulvomycin**, **enacyloxin** and **kirromycin** (Figure 16A),^[287]^ which are difficult to optimise by medicinal chemistry approaches.

Enzymes performing the co‐translational removal of N‐terminal formylation of Met are classified as **peptide deformylases** (**PDFs**). They are Fe^2+^‐dependent metallohydrolases which occur in bacteria but not in eukaryotes, which makes them attractive antibacterial targets. The natural product **actinonin** (Figure 16B and Table 2)^[288]^ has been identified as the first PDF inhibitor. Its synthetic analog, **BB‐3497** (Figure 16B and Table 2), originates from a metalloenzyme inhibitor library and showed improved MICs and efficacy in a murine infection model (oral, *Sa*).^[289]^ The characteristic hydroxamate moiety similarly occurs in follow‐up inhibitors (Figure 16B and Table 2) developed by structure‐based design and two compounds even reached clinical phase I, e.g., **BB‐83698** ^[290]^ (intravenous) and **LBM‐415** ^[291]^ (oral).

**Conclusions**. Besides continued work on existing antibacterial classes, e.g., tetracyclines,[^183^, ^184^] erythromycins^[185]^ and clindamycin,^[182]^ there are few new, often peptidic natural product ribosome inhibitors, which mostly show anti‐G+ activity (Table 2). Interestingly, aminoacyl‐tRNA synthetases[^292^, ^293^] and peptide deformylases^[294]^ did not succeed as targets for small molecule compounds due to resistance problems and research efforts seem to have decreased in recent years.

### 3.4 DNA‐Synthesis

The synthesis of DNA involves various factors which are known as the replisome.^[295]^ Important associated factors are **DNA polymerases** Pol I‐V (replication & repair), topoisomerases as well as ligases and helicases (Figure 1). Pol III is a complex of several subunits and probably can be considered as the primary enzyme of the replisome. However, with Pol lII the situation is complicated due to lower sequential homologies between polymerases of G− bacteria (DnaE1), low GC G+ bacteria (PolC/DnaE3), high GC G+ bacteria (PolC/DnaE1) and further variations.[^296^, ^297^] Additional replicational processes comprise initiation by DnaA, formation of the primosome (DNA helicase DnaB/C, primase DnaG), as well as single strand binding (SSBs) proteins, ligase (LigA) and topoisomerases (gyrase/topoisomerase IV).^[298]^

The polyketide‐type **nargenicins**,[^299^, ^300^] (Figure 17A and Table 3), isolated from *Nocardia argentinensis*, have been known since the 1980ies. They target DNA polymerase subunit α (DnaE (*Sa*)^[301]^/DnaE1(*Mt*)).^[302]^ Structural data show that nargenicin A1 interacts at the site of the incoming nucleotide as well as with the templating base (data not shown). The activity spectrum, which has been explored by a comprehensive semi‐synthetic SAR program, however, is limited to *S. aureus* and *M. tuberculosis*.^[303]^ This is also similarly true for the structurally related **branimycins** (Figure 17C) which show activity against *S. aureus*.^[304]^

A whole series of nucleoside analogs from rational and target‐based design act as inhibitors of DNA polymerase PolC. The development dates back to work from the 1960ies.^[305]^ Representatives from the group of 6‐anilinouracils,[^306^, ^307^, ^308^] e.g., **HB‐EMAU**[^309^, ^310^] and **6‐anilinouracil cpd 7 k** (Figure 17C and Table 3),^[308]^ commonly showed good activity against G+ bacteria. The guanosine‐type inhibitor **ibezapolstat** (ACX‐362E; Acurxpharma; Figure 17C and Table 3) is in clinical development for treatment of *C. difficile* infections.[^311^, ^312^, ^313^]

The lipopeptide **griselimycin** (Figure 17A/B and Table 3) was discovered in the 1960ies from *Streptomyces caelicus*.^[314]^ Griselimycin and its synthetic analog **cyclohexyl‐griselimycin** (Figure 17A and Table 3) bind to the sliding clamp DnaN of DNA polymerase,^[315]^ and show strong bactericidal activity against *M. tuberculosis*. After a period of dormancy, griselimycin was reconsidered for drug development by Sanofi. A chemical synthesis route was developed and >200 derivatives were synthesized to establish SAR. Interestingly, cyclohexyl‐griselimycin is orally bioavailable (mouse), a rare case considering its peptidic nature and size.

An important argument for choosing **DNA ligase** LigA as a target, is the high conservation among bacteria and low sequence homology with eukaryotic ligases. From a target‐based HTS at Bayer AG the **pyridochromanones** (**cpd 3**, Figure 17D and Table 3) were identified as inhibitors of NAD^+^‐dependent DNA ligase *(E. coli*/*S. pneumoniae*).^[316]^ The compounds were found to be competitive with the NADH cofactor, but activity against *E. coli* could only be achieved in presence of the membranolytic polymyxin. Another target‐based screening by AstraZeneca revealed **adenosine‐type analogs** (**cpd 4**; Figure 17D and Table 3) with predominant activity against G+ bacteria and efficacy in murine infection models.^[317]^ **Helicases** have been addressed by two target‐based screening campaigns: 186,000 compounds were tested against helicases of *B. anthracis* and *S. aureus* yielding coumarin derivatives with activity restricted to *B. anthracis*.^[318]^ Further medicinal chemistry efforts led to **coumarin cpd 22** (Figure 17E and Table 3) which had potent activity against MRSA and moderate activity against VRE.^[319]^ **Triaminotriazine cpd 1** (Figure 17E and Table 3) was identified from 230,000 tested compounds against *P. aeruginosa* helicases, but synthetic analogs showed only activity against *S. aureus* as well as undesired cytotoxicity.^[320]^

The enzymes **gyrase** and **topoisomerase IV** remove topological strain from unwinding double stranded DNA (Figure 1).^[321]^ While gyrase (GyrA/B) impacts the work of DNA polymerase (replication), topoisomerase IV (ParC/E) is involved in decatenating DNA daughter strands following DNA replication. Both enzymes are ATP‐dependent heterotetramers (dimers of two heterodimers). These enzymes count amongst the most established antibacterial targets and compounds which target these enzymes have a high likelihood of efficacy, for example the quinolones, such as **moxifloxacin** (Figure 10), which are a big class of important clinically used antibacterials.

A comparatively old example of a natural product is **cyclothialidine** (Figure 18C) synthesized by *Streptomyces filipinensis* NR0484.[^322^, ^323^, ^324^, ^325^] The compound targets ATPase activity of GyrB. Despite an enormous synthetic program in past decades aiming for structural enhancements,^[326]^ analogs only showed a limited in vitro and in vivo profile, mostly against G+ bacteria. Another natural product is **amycolamicin** ^[327]^ found to be identical with **kibdelomycin**[^328^, ^329^, ^330^] (Figure 18C and Table 4), the latter was identified from a *Staphylococcus aureus* Fitness Test (SaFT) assay consisting of 245 inducible antisense RNA strains. It is an inhibitor of GyrB and ParE^[329]^ and shows strong activity against G+ bacteria (*Sa*, *Ef*), as well as some G− bacteria (*Ab*).^[331]^ Although various total syntheses are known,^[330]^ the complex structure hampers the establishment of SAR and subsequent development of candidate drugs. A noteworthy series of molecules from structure‐based drug design (SBDD) are the so called tricyclic GyrB/ParE inhibitors (**TriBEs**, **cpd C4**; Trius Therapeutics; Figure 18C and Table 4)[^332^, ^333^] with high in vitro activities against a broad panel of ESKAPE strains, which recently experienced revived interest for application in *N. gonorrhoeae* infection models.^[334]^

The **novel bacterial topoisomerase inhibitors** (**NBTIs**) have mechanisms of action against gyrase that is different to that of fluoroquinolones. Prominent members of this family of synthetic compounds are **NXL‐101** (**viquidacin**), **AZD9742**, **BWC0977** and **gepotidacin** (Figures 18A and D). Most advanced is gepotidacin (GSK2140944, has positive phase III data, Table 4), which originates from an antibacterial screening^[335]^ and targets GyrA/ParC.^[336]^ It has very good antibacterial activity against G+ and some G− bacteria, is orally bioavailable, and is currently being assessed in clinical studies against urinary tract infections (UTIs, *E. coli*) and infections with *Neisseria gonorrhoeae*. The piperidyl‐quinoline‐type NXL‐101 displays anti‐*Sa*/*Sp* activity.^[337]^ Development was discontinued due to cardiac QT interval prolongation.^[338]^ BWC0977 (Table 4) is a more recent molecule with activity also against G− and is currently in clinical phase I.[^339^, ^340^]

The spiropyrimidinetrione (SPT) **Zoliflodacin** (AZD0914/ETX0914; Figure 18A and Table 4) is derived from an antibacterial phenotypic screening (250,000 compounds) and subsequent synthetic optimizations.[^341^, ^342^] It targets GyrB with activity against G+ and some G− bacteria, is also orally available and is being clinically investigated for infections with *Neisseria gonorrhoeae*.^[343]^

**Albicidin** (Figure 18A and Table 4) was found in the early 1980s as a natural product from the plant pathogenic bacterium *Xanthomonas albilineans* which causes chlorosis of sugar cane (leaf scald disease).^[119]^ Its target is bacterial gyrase and antibacterial activity against G+ and G− bacteria was reported.^[55]^ Remarkably, even before knowing the chemical structure, a number of resistance factors were described: binding proteins AlbA and AlbB, the protease AlbD, and Tsx as the entrance channel of the outer membrane.^[344]^ The challenging structure elucidation was achieved more than three decades after its discovery.[^345^, ^346^] The total synthesis of albicidin^[176]^ represents the conceptual roadmap for variations and related structures. Since then, albicidin has been subject of extensive structure–activity‐relationship (SAR) studies.[^347^, ^348^, ^349^, ^350^] Structural optimizations have led to derivatives, e.g. **AF‐CD‐007** (Figure 18A and Table 4) with excellent activity against various G+ and G− strains and proof of concept was achieved with a murine septicemia infection model (FQR‐resistant *E. coli*).^[177]^ The **cystobactamids** (Figure 18C and Table 4) are structurally very similar to albicidin. They were isolated from the predatory myxobacterium *Cystobacter* sp. and derivatives also showed very good antimicrobial properties, as well as in vivo activity in a murine neutropenic thigh infection model (*E. coli*).[^351^, ^352^] Recently, the structure of the ternary gyrase‐DNA‐albicidin complex was determined by cryo‐EM (Figure 18B).^[353]^ Another gyrase inhibitor is the cyclodepsipeptide **evybactin** (Figure 18C and Table 4)^[354]^ found in an anti‐MT screening of bacterial nematode symbionts and isolated from *Photorhabdus noenieputensis*. Cellular uptake of this sizeable peptide has been attributed to the BacA transporter expressed in mycobacteria. The small molecule **LEI‐800** (Figure 18C) is the most recent GyrA inhibitor which binds in a new binding site, and has shown promising activity against G− bacteria (*Ec*, *Kp*).^[355]^ **DNM‐2** is a deoxynybomycin (Figure 18C) derivative originally isolated from *Streptomyces* that acts particularly on FQR gyrase.[^356^, ^357^] Interestingly, resistance development of MRSA harboring the S84 L *gyrA* mutation against DNM‐2 re‐sensitizes these strains against fluoroquinolones.

**Conclusions**. While DNA polymerase could be considered the most obvious target of the replisome, sequence differences between bacteria make a universal inhibitor of G+ and G− bacteria difficult (Table 3).^[297]^ Unlike for antiviral therapies, nucleobase‐type drugs as inhibitors of DNA‐polymerases seem less represented and less successful in research seeking novel antibacterials, possibly due to limited water solubility, penetration issues and a steep SAR, but also due to their restriction to G+ bacteria. An exception for a nucleobase‐type drug is ibezapolstat (Figure 17) which is in clinical phases for treatment of *C. difficile* infections. Interestingly, thus far the most effective target of the replisome is gyrase/topoisomerase IV. Inhibitors, which many times show broad spectrum activity (Table 4), are small molecules and natural products of varying structural complexity. The MOA of the NBTIs commonly involves interaction with topoisomerase‐bound DNA. Apart from the small molecule NBTIs, albicidins/cystobactamides constitute a new chemotype which can be synthetically modified. Other targets, e.g., further DNA polymerase subunits, helicases, SSBs, and ligases, seem under‐explored and have not yet delivered convincing antibacterial compounds.

### 3.5 RNA‐Synthesis

The essentiality, high conservation and sufficient evolutionary distance from eukaryotic RNA polymerases turn the bacterial **RNA polymerase** (**RNAP**; Figure 1) into an attractive target.^[358]^ To date, the polyketide‐ansamycins of the **rifamycin** group (Figure 19A) (**rifampicin**: Figure 10) are successfully used in anti‐TB therapy and lipiarmycins (**fidaxomicin**, Figure 10) are used for treatment of *C. difficile* infections. The structurally complex tetramic‐acid antibiotic **streptolydigin** (anti‐G+)[^359^, ^360^, ^361^] (Figure 19A) was among the first discovered RNAP inhibitors. Remarkably, a whole series of myxobacterial RNA polymerase inhibitors with anti‐G+ activity exist: **sorangicin A**,[^362^, ^363^] **myxopyronin B**,[^364^, ^365^] and **ripostatin B** (cytotoxicity)^[366]^ (Figure 19A and B, Table 5), which date back in their discovery to the 1980ies and 1990ies by researchers from GBF (Braunschweig, Germany). **Corallopyronin A** (Figure 19B and Table 5)^[367]^ is structurally related to myxopyronin. Its activity against G− *Wolbachia* species, which are obligate endosymbiont (intracellular) bacteria of nematodes, makes it of high interest for the treatment of filarial diseases for which it is currently being investigated for potential clinical applications.^[368]^ Peptide‐type inhibitors of RNAP are **GE23077** (Figure 19A)[^369^, ^370^] and the depsipeptide **salinamide A** (Figure 19B and Table 5).^[371]^ The latter shows only narrow spectrum antimicrobial activity. **CBR703** (Figure 19B) is a representative of a small molecule series described as allosteric inhibitors.[^372^, ^373^] However, moderate activity against G+ bacteria accompanied by cytotoxicity makes this compound type less useful.^[374]^ Remarkably, **pseudouridimycin** (Figure 19B and Table 5) from a target‐based extract screening is a rare example of a natural product‐type nucleoside RNAP inhibitor, which displays mostly anti‐G+ activity in vitro and in vivo.^[375]^ A particular example is the small molecule **ridinilazole** (SMT19969) (Figure 19C and Table 5) which reached clinical phase III recently for treatment of *C. difficile* infections.^[376]^ It is a bis‐benzimidazole, which originates from rational and target‐based design of DNA‐binders of the **Hoechst‐dye** or **netropsin/distamycin** class (Figure 19C). Some precursor analogs show good anti‐G+ activity (*Sa*).[^377^, ^378^] Ridinilazole itself has narrow activity against *C. difficile*, is basically not orally bioavailable and has a poor systemic absorption. For a while the MOA seemed unclear, but most recent reports indeed suggest minor groove binding of DNA with effects on transcriptional processes.^[379]^

**Conclusions**. Interference with RNA synthesis is basically restricted to RNA polymerase (RNAP) and activity is mostly against G+ bacteria (Table 5). Most RNAP inhibitors are complex polyketides, which makes SAR and optimization studies challenging. Inhibitors of factors adjacent to transcription, e.g., **bicyclomycin** (Figure 19D) which binds to the ATPase Rho factor which is involved in termination, are rare.^[380]^ In view of the high potential of the RNAP target, e.g., broad‐spectrum activity, it is remarkable that only a comparatively low number of inhibitors is known. However, addressing RNAP as a single target bears the risk of high level resistance development.^[1]^

### 3.6 Fatty Acid Biosynthesis

Fatty acid synthesis provides building blocks for glycerophospholipids as constituents of the cell membrane (Figure 1).^[29]^ Inhibition of bacterial **fatty acid synthase** (FAS II) and other enzymes involved in fatty acid synthesis provides some targets, which cannot be circumvented by addition of exogenous fatty acids. Unlike the human FAS I (multidomain protein), the bacterial FAS II system is dissociated into single domains. Target enzymes catalyze rate‐limiting reactions (Figure 20B). These are: ACP synthase which performs phosphopantetheine transfer to apo‐ACP (AcpS); acetyl‐CoA‐carboxylase complex (AccABCD) for the synthesis of malonyl‐CoA, β‐ketoacyl‐ACP synthase III (FabH), β‐ketoacyl synthases I (FabB – unsaturated FA) and II (FabF) which perform chain extensions.[^29^, ^381^] FabA/Z are dehydratases which have been hardly explored as targets. The enoylreductases exist in various isoforms (FabI/K/L/V) which execute the final step in each fatty acid elongation cycle. However, their occurrence is strain‐dependent. This and other aspects make fatty acid synthesis inhibition pathogen‐dependent: For G− bacteria, the free hydroxy fatty acids required for synthesis of lipid A/lipopolysaccharides (LPS) cannot be activated from exogenous sources and must be supplied by FASII biosynthesis in an ACP‐bound form.^[29]^ Similarly, the G+ bacterium *S. aureus* requires branched fatty acids that cannot be provided by exogenous sources. In contrast, *S. pneumoniae* can supply its needs from extracellular lipids, so that FAS is not essential. In mycobacteria, the essential long‐alkyl chain **mycolic acids** (Figure 1) are a major part of the mycomembrane and are synthesized by mycobacterial FAS I and FAS II.^[382]^

**Afabicin** (formerly Debio 1450, AFN‐1720) (Figure 20A and Table 6) is a small‐molecule phosphate ester prodrug of **Debio 1452** (formerly AFN‐1252).[^383^, ^384^, ^385^] Initially, a hit structure from a target‐based HTS program (~2002, GSK) (Figure 8D) showed weak FabI inhibition and no activity against *S. aureus*.[^386^, ^387^] This hit was further optimized into a compound structurally already very similar to afabicin, with high anti‐staphylococcal activity. Debio 1452 (Figure 20A) inhibits the NAD(P)‐dependant enoyl‐reductase FabI[^388^, ^389^] of *S. aureus* (IC_50_=49 nM) and *E. coli* (IC_50_=0.7 nM). From various forms of other bacterial enoyl ACP‐reductases (FabK, FabL and FabV), FabI is the only enoyl reductase present in *S. aureus* and *S. epidermidis*. Inefficacy of Debio 1452 against *E. coli* is seemingly linked to efflux.^[383]^ Debio 1452 is selective for *S. aureus* and *S. epidermidis* ^[383]^ and showed very good in vivo performance in infection models.^[385]^ The more soluble prodrug afabicin can be administered intravenously and orally, and is in clinical trials for treatment of acute bacterial skin and skin structure infections.^[390]^ Afabicin was also investigated for penetration of bone tissue for a therapy to prevent bone and joint infections during surgery.^[391]^ Recently, by introducing an amino group (**Debio‐1452‐NH3**; Figure 20A and Table 6), researchers could endow afabicin with anti‐G− activity^[171]^ and then apply the same approach to an expanded ring‐analog called **fabimycin** (Figure 20A and Table 6).^[195]^ Other small molecule inhibitors of staphylococcal FabI are **MUT056399** ^[392]^ and **nilofabicin** (CG400549) (Figure 20A and Table 6),^[393]^ the latter has entered into clinical trials.

**Platensimycin** (Figure 20C and Table 6), synthesized by *Streptomyces platensis*, was discovered at Merck from a screening campaign (83,000 strains) employing a cell‐based target screening with a specialized silencing technology.[^394^, ^395^] The compound selectively targets FabF and showed good anti‐G+ activity as well as low cellular toxicity. Efficacy was shown in a *S. aureus* mouse infection model. The pharmacokinetic properties were however not favorable, which was explained by a high clearance. Apart from total syntheses,[^396^, ^397^] several analogs were isolated from *Streptomyces platensis* or generated by semi‐synthesis[^398^, ^399^, ^400^, ^401^] but these lacked enhanced properties. The structurally related **platencin** (Figure 20C and Table 6),^[402]^ with a similar antibacterial profile, is a dual acting inhibitor of FabF and the initiation condensing enzyme FabH.^[403]^ The natural product **andrimide** (Figure 20D and Table 6) targets acetyl‐CoA carboxylase (AccAD), which is highly conserved among bacteria.^[404]^ However, derivatives show an unfavorable SAR and the compound's performance was mostly further investigated only for *S. aureus*.

In anti‐mycobacterial therapy, the enoyl‐ACP reductase (InhA‐FAS II) involved in mycolic acid synthesis is targeted by isoniazid (Figure 10). This is also the target of **pyridomycin** (Figure 20D and Table 6), a long known cyclodepsipeptide for which SAR^[405]^ and target ID in mycobacteria^[406]^ have been elucidated. **SQ109** (Figure 20D and Table 6), which reached clinical phases, comes from an anti‐mycobacterial screening of a synthetic library of etambutol‐analogs. It prevents the translocation of the mycobacterial lipid intermediate trehalose monomycolate (TMM) across the membrane.[^407^, ^408^] Subsequently it was found that SQ109 acts on multiple targets including menaquinone biosynthesis and has uncoupler activity (see section 3.8.).^[409]^

**Conclusions**. FabF/I are the main targets from the fatty acid synthesis pathway. Concerning medicinal chemistry aspects, often‐mentioned FAS inhibitors like cerulenin (epoxide), amycomicin (epoxide, isonitrile) or fasamycin (aromatic polyphenol) are difficult scaffolds, partially considering their lipid‐like or hydrophobic nature, cytotoxicity, synthetic access and ease of structural variation. Afabicin and platensimycin stand out because of their in‐depth profiling as drugs. However, most inhibitors address only G+ bacteria including mycobacteria, implying narrow spectrum applications (Table 6). Considering its chemical structure and despite its relationship to ethambutol, it may be surprising that SQ109 addresses a specific mycobacterial target involved in lipid transport.

### 3.7 Cytoplasmic and Membrane‐Bound Proteases and Cellular Degradation Machineries

Bacterial proteases comprise cytoplasmic oligomeric and **ATP‐dependent proteases** (AAA+ proteases),^[410]^ and amongst others, caseinolytic protease (ClpP, serine protease),^[411]^ membrane bound metalloprotease FtsH,^[412]^ Thr‐dependent protease HslUV (ClpQ/V),^[413]^ and the serine protease Lon.^[414]^ They are involved in various processes of proteostasis, protein quality control, and virulence. The most researched proteases for finding antibacterial drugs are those from the Clp family. The activity of the protease ClpP (Figure 1) is tightly controlled by activation of accessory AAA+ ATPases ClpC/X (G+ bacteria) or ClpA/X (G− bacteria). Molecules targeting Clp proteases can be categorized into inhibitors, activators and ATPase uncouplers. Furthermore, Clp‐essentiality is pathogen dependent, e.g., for mycobacteria (ClpP1)^[415]^ but not for *S. aureus*.

The natural products of the **A54556A** ^[416]^ and **enopeptin** series^[417]^ belong to the **acyldepsipeptides** or **cyclic acyldepsipeptides** (**ADEPs**) (Figure 21A and Table 7) and are activators of ClpP (Figure 21B/C),[^418^, ^419^] a target originally identified by a team at Bayer AG.^[420]^ For these compounds, dysregulation of ClpP leads to a long‐filamented phenotype (*B. subtilis*/*S. aureus*) which inhibits septum formation at the stage of Z‐ring assembly, degrading the cell division protein FtsZ.^[421]^ FtsZ has been shown to be particularly sensitive to ADEP‐activated degradation by ClpP.^[422]^ Lack of activity against G− bacteria has been thought to be due to efflux mechanisms. A straightforward total synthesis of enopeptin B^[423]^ permitted the establishment of an extended SAR,^[424]^ from which a significant enhancement in activity against G+ bacteria and in vivo efficacy was achieved, e.g. compound **ADEP4** (Figure 21A and Table 7). Further compound design rendered urea‐type compounds, termed **UDEPs** (Figure 21A) which maintained bioactivity and improved metabolic stability.^[425]^ More recently, the anti‐G+ **armeniaspiroles** (Figure 23C and Table 8), initially classified as depolarizers,^[426]^ were characterized as dual inhibitors of ClpX/P and ClpY/Q, impacting cell division.[^427^, ^428^] Among the Clp inhibitors, β‐lactones stand out as irreversible binders,^[429]^ but they rather reduce virulence (*S. aureus*).^[430]^

A particularity of *M. tuberculosis* is the essentiality of the ATPases ClpC1 and ClpX, which are targeted by various natural product peptides (Figure 21D and Table 7): **cyclomarin A** (ClpP1/2, ClpX),[^431^, ^432^] **rufomycin** (ilamycin) (ClpC1),^[433]^ **ecumicin** (ClpC1),^[434]^ and **lassomycin** (ClpC1).[^435^, ^436^] Inhibitor‐protease complexes that characterize the binding site on ClpC1 are known for cyclomarin^[437]^ and rufomycin.^[438]^

Another important group of proteases are **membrane‐bound signal peptidases (SPs or SPases)** of G+ and G− bacteria involved in (lipo)protein secretion (Figure 1). **SPs type‐I** (SP‐I) are serine proteases of the cytoplasmic membrane that process non‐lipoproteins; **SP type II** (SP‐II) or Asp proteases that process lipoproteins.^[439]^ Both protease‐types cleave signal sequences from pre‐proteins following their translocation (Sec/Tat) across the cytoplasmic membrane. Due to their unique features, SPs have in earlier times been suggested as antibiotic targets.^[440]^

The **arylomycins** (Figure 22A and Table 7) are a class of macrocyclic lipopeptides first described by Fiedler and Jung.[^441^, ^442^] Subsequently, other groups reported SP‐I as the molecular target.^[443]^ The paper on the total synthesis of arylomycin A2 by the Romesberg group reported low activity against few G+ bacteria (e.g. *S. epidermidis*).^[178]^ This contribution provided a template for analog synthesis,[^444^, ^445^] enabling subsequent MOA studies and establishment of an SAR.[^446^, ^447^, ^448^, ^449^] The idea of a C‐terminal aldehyde extension as a warhead possibly reacting with the catalytic Ser‐Lys dyad of SPase or an aminoethoxy modification of the phenols led in part to interesting MICs and IC_50_ies for SPase inhibitors of the arylomycin‐type.^[447]^

A game‐changing contribution came from a team at Genentech in 2018 on new arylomycin analogs.^[179]^ The study commenced with the rationale for synthetic design. A significant advancement of activity against *E. coli* was attained when the N‐terminal acyl‐tripeptide of arylomycin was replaced by a substituted pyrimidine‐diaminobutyryl residue. The attachment of an aminoacetonitrile warhead at the C‐terminus, forming an anticipated covalent bond with the catalytic Ser residue from the active site, further improved the antibacterial activity. Ultimately, the phenols were transformed into aminoethoxy ethers, bearing positive charges. The best performing candidate **G0775** (Figure 8B, Figure 22A and Table 7), turned out to be highly potent against a set of ESKAPE strains including MDR pathogens. The crystal structure of G0775 with SP‐I LepB (*E. coli*, Figure 22B) revealed covalent bond formation with the catalytic Lys via an amidine, and not with Ser as initially anticipated. The uptake was shown to be porin‐independent, possibly by a self‐promoted mechanism mediated by the cationic charges of the molecule.^[450]^ Subsequent in vivo experiments with different pathogenic G− bacteria revealed a high efficacy in preclinical models of infection. As the authors state in their article, >1000 arylomycins were synthesized, which outlined the requirements for the number of optimized analogs necessary to obtain a reliable SAR.

Examples for inhibitors of lipoprotein signal peptidase II (LspA) which is essential for G− bacteria but not for G+ bacteria, are the moderately anti‐G− peptide **globomycin** and the polyketide **myxovirescin** (Figure 22C), for which co‐crystal structures exist.[^451^, ^452^]

**Conclusions**. Among compounds targeting proteases ClpP and SP‐I, natural product‐derived peptides have shown the greatest impact thus far. Small molecule hits from HTS screenings, e.g., as Clp activators assessed in vitro,^[453]^ still have to be successfully optimized to yield viable drug‐like compounds. With the exception of the optimized arylomycin analog G0775, the antibacterial spectrum is restricted to G+ bacteria. Targeting cytoplasmic proteases presents further challenges as the drug must be able to permeate the cell membrane(s). Nevertheless, this approach appears to be remarkably effective for mycobacterial targets and peptide‐type drugs, an aspect which deserves further consideration.

### 3.8 Lipid Membranes and Bioenergetics – Disruptors, Depolarizers and Menaquinone Binders

The **cytoplasmic membrane** (CM, G+/G− bacteria)[^454^, ^455^] and the **outer membrane** (OM, G− bacteria)^[456]^ constitute permeability barriers for diffusion and transport of large molecules such as proteins but also of some compounds with lower molecular mass, e.g. nutrients (Figure 1). Particularly, the OM of G− bacteria is a major barrier for antibacterial drugs. Diffusion of certain molecules into the periplasm is typically facilitated by porins, which are β‐barrel proteins embedded in the outer membrane. Furthermore, the CM of G+ and G− bacteria contains the components of the **respiratory chain** (dehydrogenases→quinone carrier→cytochrome bc 1 complex→oxidases, ATP synthase; Figure 1), which under aerobic conditions convert chemical energy into a proton‐motive‐force (PMF) used to synthesize ATP.^[457]^ However, pathways of the individual respiratory chains can be complex and strain‐specific. Inhibitory molecules can either directly impact the integrity of the membrane and/or interfere with components of the respiratory chain to disturb electrochemical gradients.

The main mechanism of action of the last‐resort antibiotic **colistin** (**polymyxin E**, pmx‐E) (Figure 10) for treatment of various infections with G− bacteria (*Ec*/*Kp*/*Pa*) is binding to the lipopolysaccharides (LPS) and phospholipids with subsequent **disruption of cell membranes**.^[458]^ Although issues of nephrotoxicity and neurotoxicity cast general suspicion on this cyclopeptide‐type compound class, structure optimizations and further research aim at improving the safety profile: The octapeptins are closely related to polymyxins (pmx) but show activity against pmx‐resistant strains (*Pa*), which points to differences in the mode of action.^[459]^ However, **octapeptin C4** (Figure 23A) and also **battacin** (**octapeptin C5**, Figure 23A) suffer from cytotoxicity in a similar way to members of the pmx group.[^460^, ^461^] The synthetic analog **FADDI‐118** (Figure 23A and Table 8) was superior in mouse models against pmx‐resistant *Pa* compared to pmx‐B and octapeptin C4, but retained nephrotoxicity.^[462]^ Compound **F365** (QPX9003; Figure 23A and Table 8),^[175]^ which is in clinical phases, is a new‐generation polymyxin with potent activity against G− bacteria (*Ec*/*Kp*/*Pa*) and a significantly improved safety profile. The recently described lipopeptides **brevicidines** and **laterocidines** (Figure 23A and Table 8)^[463]^ with anti‐G− activity have been discovered by a genome mining approach for cationic NRPs from 5585 bacterial genomes. Solid‐phase peptide synthesis provided rapid access to SAR.^[464]^ An example for a compound mimicking the amphiphilic nature of antimicrobial peptides (AMPs) is **brilacidin** (PMX‐30063; Polymedix, Inc.; Figure 23A). Its design is based on synthesis and calculations derived from studies on foldamers.[^465^, ^466^] Investigations with *E. coli* show perturbations of the outer membrane and, to a lesser extent of the inner membrane.^[466]^ Brilacidin, which acts on G− and G+ bacteria (Table 8) has advanced to clinical phases for treatment of infections with *S. aureus*.^[467]^

The anti‐G+ **calcium‐binding antibiotics** constitute another compound class with the prominent representatives **daptomycin** (Figure 10) and **friulimicin B** (Figure 12C). Despite structural similarities, the cyclopeptides have distinct modes‐of‐action. The clinically used daptomycin has initially been suggested as pore‐forming and membrane depolarizing,[^468^, ^469^] but recent investigations suggest that it affects membrane fluidity by binding to phosphatidylglycerol and bactoprenol‐coupled lipid II intermediates, leading to a detachment of lipid II synthase MurG and phospholipid II synthase PlsX from membrane microdomains.[^470^, ^471^] The activity spectrum includes *S. aureus* (MSSA/MRSA) and vancomycin‐resistant *Enterococci* (VRE). Various analogs have been synthesized by combinatorial biosynthesis^[190]^ or by chemical synthesis.^[472]^ Friulimicin B (Figure 12C and Table 8) from *Actinoplanes friuliensis* ^[473]^ forms a Ca^2+^‐dependent complex with the bactoprenol phosphate carrier C_55_‐P and may be therefore rather classified as a cell wall synthesis inhibitor.^[474]^ Previously, it was considered for clinical development with a G+ activity spectrum (Combinature Biopharm AG now MerLion Pharmaceuticals GmbH).^[475]^

Interference with components of the **electron transport chain** provides a different concept of antibacterial therapy, particularly for mycobacteria. A prominent example is the medically used **bedaquiline** (Figure 10), which inhibits F_1_F_o_‐ATP synthase.^[476]^ Other inhibitors acting on the cytochrome bc_1_ complex (QcrB) are **telacebec** (Q203; clinical phase II) (Figure 23B and Table 8), which originates from a phenotypic high‐content screening.[^92^, ^477^] Further inhibitors include **lansoprazole** (Figure 23B and Table 8) derived from a drug repurposing approach,^[133]^ and the arylvinylpiperazine amides (**AX‐35,** GW861072X; Figure 23B), which originate from a phenotypic screening.^[478]^ As an example, inhibitors^[479]^ and activators^[480]^ have been identified for the mycobacterial type II NADH‐dehydrogenase (NDH‐2). However, in order to achieve therapeutic impact, strategies are still under discussion.^[481]^

**Depolarization** of the cell membrane by rapid proton translocation and thus indirect interference with the bacterial respiratory chain is a second MOA of the anti‐mycobacterial F_1_F_O_‐ATP synthase inhibitor bedaquiline^[482]^ and of the previously mentioned mycolic acid transport inhibitor (MmpL3) **SQ109** (anti‐TB; clinical phase II; Figure 20D).[^407^, ^408^] SQ109 was also found to be a menaquinone biosynthesis inhibitor.^[409]^ The **armeniaspirols** ^[426]^ (Figure 23C and Table 8) (also recently described as Clp inhibitors[^427^, ^428^]), the previously mentioned **halicin** and **phenoxy‐*N*‐phenylacetamide cpd 1** (Figure 23C and Table 8), and peptides like **lugdunin** (nanochannel formation; Figure 23C),[^483^, ^484^] which act against G+ bacteria, are further compounds targeting the proton motive force (PMF). Their MOA is partially reminiscent of uncouplers of oxidative phosphorylation in eukaryotic mitochondria, e.g., dinitrophenol, which are known for toxic side‐effects.^[485]^

An interesting mode of action is binding to menaquinone (Figure 1), a membrane‐associated key electron carrier in the respiratory chain of G+ bacteria. This has been described for **lysocin E** (anti‐*Sa* activity) (Figure 23D and Table 8)^[486]^ and diverse **menaquinone‐binding antibiotics** (**MBAs**; anti‐*Mt* activity),[^487^, ^488^, ^489^] which are all cyclopeptides. They facilitate Ca^2+^‐leakage and membrane damage. In G− bacteria, ubiquinone, which is also essential for humans (vitamin K), has an analogous function to menaquinone. However, ubiquinone binders have not been reported so far and do not appear suited for consideration as drugs.

**Conclusions**. Albeit having sometimes similar structures, the individual MOAs of mostly polycationic antimicrobial peptides (AMPs)^[490]^ of the pmx‐family that display membrane‐disruptive effects, may ultimately be different and complex. Development of these types of drugs as therapeutics potentially bear a risk of undesired side‐effects, e.g., interferences with eukaryotic membranes. On the other hand, many AMPs of the pmx‐type display highly desirable anti‐G− activity. Design approaches, as seen for foldamers like brilacidin can lead to similar results as AMPs. Interference with the electron transport chain by specific inhibitors or by uncoupling has turned out to be a viable antibacterial concept, particularly for mycobacteria, which require a fully energized membrane.^[491]^ Unfortunately, the other compound classes that interfere with bacterial membranes are restricted to anti‐G+ activity.

### 3.9 Membrane Proteins and Biogenesis of Membrane Components

The bacterial membrane contains various protein components involved in the assembly and maintenance of its stability and integrity. **Lipopolysaccharides** (LPS) and the sub‐component **lipid A** are characteristic for most G− bacteria (Figure 1), whereas the wall teichoic acids (WTAs) and lipoteichoic acids (LTAs) are characteristic for most G+ bacteria (Figure 1).^[492]^ LPSs consist of a lipidated and phosphorylated glucosamine disaccharide (lipid A) inserted into the membrane, which is connected to a mostly disaccharide‐type core region and a length‐variable O‐polysaccharide chain.^[493]^ The LPS core is assembled in the inner membrane by **Lpx proteins** and flipped via the ABC‐transporter MsbA to the periplasm and further guided by a chain of **Lpt proteins** (lipopolysaccharide transport, LptB_2_FG, LptC/A and LptD/E) to the outer membrane (Figure 24).[^493^, ^494^] It was found that LPS are essential for most G− bacteria (exception, e.g., *Acinetobacter baumannii* ^[495]^), and that a balance between LPS and phospholipid synthesis needs to be maintained.^[496]^

In **lipid A biosynthesis** of G− bacteria, the Zn^2+^/Fe^2+^‐dependent deacetylase LpxC is the first committed step (Figure 24).^[493]^ LpxC is the target of characteristic chelating hydroxamic acid‐based small molecules developed by rational and target‐based design.^[497]^ Due to extensive activities in industry and academia, various compounds with excellent activities against bacteria were identified, e.g., indazole and indole‐type inhibitors.^[498]^ Non‐hydroxamate type compounds are rare.^[499]^ The most advanced hydroxamate‐containing compounds are **LpxC‐4** (PF‐5081090, Pfizer) and **ACHN‐975** (Achaogen) (Figure 24D and Table 9). The latter was the first one to reach clinical phases, but further development was halted due to unwanted side effects,^[500]^ and recent attempts were not successful in advancing this compound.^[501]^ Additionally, small molecule‐based screenings for other Lpx enzymes have been conducted, e.g., LpxA,^[502]^ or LpxH,[^503^, ^504^, ^505^] but with moderate MICs until the most recent report on small molecule LpxH inhibitors. The compounds, e.g. **EBL‐3647** (Figure 24F), show a pronounced activity against *K. pneumoniae* and *E. coli* including evaluation in animal infection models.^[506]^ Small molecule inhibitors of the transporter MsbA, e.g., **G907 analogs** (**compound 42**; Figure 24E and Table 9) with activity against some G− bacteria,[^507^, ^508^] are noteworthy but rather remain in an exploratory stage.

In the multifaceted discovery of the polycationic β‐hairpin peptide **murepavadin** (POL7080) (Figures 8C, 24A and Table 9),^[33]^ the lipopolysaccharide transport protein LptD was identified as the target being based on a non‐lytic antibacterial mechanism of action.[^33^, ^509^] Initial work comes from the Robinson group, which was continued by Polyphor Ltd (now Spexis, Allschwil, Switzerland). The structure of the fully synthetic 14mer peptide was obtained in several optimization rounds^[510]^ from the polycationic host defence peptide **protegrin I** (PG‐1) (Figure 8C).^[511]^ PG‐1 has broad antibacterial[^512^, ^513^] but also haemolytic activity. Early derivatives generated by solid‐phase peptide synthesis showed moderate antibacterial activity and reduced haemolytic activity. Ultimately, the optimization attempts led to **POL7001** (Figure 8C) and murepavadin (Figure 8C),^[33]^ which specifically exhibited activity against various *P. aeruginosa* strains. Subsequent studies^[514]^ confirmed the essential role of LptD for viability of *Pa*. Efficacy in murine *Pa* infection models and further development led to evaluation in clinical phases for the treatment of patients with nosocomial pneumonia.^[515]^ The development as an intravenous formulation was stopped due to unexpected kidney injury findings, but the compound is still in development for inhaled applications.^[516]^ With the 14mer peptide **JB‐95** (Figure 24A and Table 9), the concept of β‐hairpin peptides has been further extended.^[509]^ JB‐95 showed activity against various ESKAPE(E) strains, including pronounced activity against *E. coli*. Investigations of the MOA revealed, similarly to murepavadin, outer membrane proteins LptD but also BamA, as well as the disruption of the outer membrane as molecular targets. A further example is the 21mer insect‐derived peptide **thanatin** (Figure 24B and Table 9),^[517]^ which shows broad antibacterial activity targeting LptD and LptA proteins of *E. coli*.^[518]^ Subsequent work by others suggests an additional mode of action on certain New Delhi metallo‐β‐lactamase (NDM‐1) producing *E. coli* and *Klebsiella* strains by OM disruption.^[519]^ Thanatin was used as a starting scaffold to derive N‐terminally truncated peptides, foremost **thanatin‐cpd 7** (Table 9) was the best analog with activity against *E. coli* and *Klebsiella* in vitro and in vivo.^[520]^ Mechanistically, these peptides have been found to disrupt LptA‐LptA and LptA‐LptD interactions, as a part of the periplasmic bridge of the Lpt‐protein complex. Only recently, the peptidomimetic **zosurabalpin** (RG6006) (Figure 24C and Table 9) which originates from a screening of a 44,985‐membered macrocyclic peptide library, has been described as an inhibitor of the LptB_2_FG complex, which is located in the cytoplasmic membrane.[^521^, ^522^] Interestingly, this compound is already in clinical phase I, and thus the unmet medical need of *A. baumannii* infections may apparently be being addressed.

Another important biosynthetic process is the transport, folding and placement of proteins in membranes of G+ and G− bacteria. The Sec pathway is a main player for co‐translational protein transport and insertion of proteins of G+ bacteria (Sec/SRP/FtsY), as well as the translocation of proteins through the plasma membrane of G+ and G− bacteria.[^523^, ^524^] A pathway for the **lipoprotein transport** of G− bacteria proceeds via the LolA‐E proteins located mainly in the periplasm, which have been found to be essential for *E. coli*.^[525]^ Here, small molecule inhibitors have been described (*E. coli*, *P. aeruginosa*)^[526]^ but in vivo studies remain elusive. Recent work on Lol‐inhibitors has been reviewed and pyridineimidazoles and ‐pyrazoles^[503]^ have been used as a basis for the development of **SMT‐738** (Figure 25A and Table 9)^[527]^ and **lolamicin** ^[528]^ (Figure 25A and Table 9). The compounds show inhibition of *E. coli*, *Klebsiella*, *Enterobacter* species and as shown for lolamicin spare bacteria from the gut microbiome.^[86]^

In G− bacteria, the proteins are further transported to the outer membrane **β‐barrel assembly machine** (**BAM**) complex which consists of four proteins (BamABCD) (Figure 1, Figure 25C).[^529^, ^530^, ^531^, ^532^] This complex, of which BamA, and conditionally BamD^[533]^ are essential components, performs the folding of proteins into transmembrane β‐barrels followed by insertion into the outer membrane.

The synthetic compound **MRL‐494** (Figure 25B and Table 9),^[534]^ an inhibitor of BamA was identified from a phenotypic screening. MRL‐494 showed interesting activity against several G− bacteria and disruption of the cytoplasmic membrane of G+ bacteria as a second mechanism. First SAR data were provided by synthesis and testing of analogs.^[535]^ **Darobactin** (Figure 25B and Table 9) is a bicyclic heptapeptide of the RiPP class, which also targets BamA.^[34]^ It was discovered in a screening of bacterial symbionts of the genus *Photorhabdus* and *Xenorhabdus*. Darobactin is a β‐strand mimic that seals the open lateral gate of BamA by binding in an antiparallel β‐sheet conformation.^[35]^ Darobactin displayed reasonable antibiotic activity against G− bacteria and was tested in vivo in several mice infection models where it showed promising results.^[34]^ Heterologous production of darobactin variants[^536^, ^537^] led to slightly improved antibacterial activity. Meanwhile, chemical syntheses[^538^, ^539^] of the challenging structure have been published, which are valuable, allowing synthesis of analogs which contribute to the understanding of SAR and possibly for the identification of optimized darobactin variants. The compound **dynobactin A** (Figure 25B and Table 9) is a more recently discovered darobactin‐related RiPP^[540]^ also targeting BamA. It displays interesting anti‐G− activity and proof‐of‐concept was shown in an in vivo infection model.

**Conclusions**. In recent years, lipopolysaccharide synthesis (Lpx/Lpt pathways)[^493^, ^531^] and the BAM complex[^530^, ^531^, ^532^, ^541^] have come into focus as antibacterial targets, which has been accompanied by advancements in the understanding of their molecular architecture and function. The discovery of broadly acting LPS synthesis inhibitors is hampered by the existence of orthologous genes, e.g., LpxH/I/G, as well as strain‐dependent differences, e.g., LptD (*Pseudomonas* vs. *E. coli*), requiring tailored drugs. In contrast, BamA is highly conserved in G− bacteria.^[542]^ Thus far, for the above pathways, few but potent inhibitors have been identified. These include the family of small molecule hydroxamate‐type chelators of cytoplasmic deacetylase LpxC,^[500]^ as well as peptides/peptidomimetics targeting LptD,[^33^, ^509^] LptA,^[520]^ BamA,^[34]^ and, most recently, LptB_2_FG.^[521]^ Since several target proteins of these pathways are located on the cell surface, a significant advantage of these targets is, that inhibitors do not have to enter the cytoplasm. In summary, the LPS, OMP and potentially Lol biosynthetic machineries are all highly promising targets in the search for new anti‐G− inhibitors.

### 3.10 Cofactor Synthesis, Nucleotide Synthesis and Other Targets

In addition to the above targets, research activities have also been performed on other pathways. The de novo **folate biosynthesis** is a **cofactor synthesis pathway** present in bacteria but not in humans with the sulfonamides (dihydropteroate synthase) and pyrimidine antibacterial agents such as trimethoprim (dihydrofolate reductase) as established drugs (Figure 10).^[543]^ Thus, inhibitors of this pathway have a lowered risk of target‐related toxicity. The trimethoprim‐analog iclaprim (anti‐G+) is one of the most recent developments, which reached clinical phase III trials.^[544]^ **Abyssomicin C** (Figure 26) is an example of a polyketide natural product that interferes with the amino‐deoxychorismate synthase (ADCS).[^545^, ^546^] However, the antibacterial activity of the abyssomicin‐type compounds is limited to G+ bacteria (*S. aureus*, mycobacteria)^[547]^ and the structure is difficult to modify chemically.

**Pantothenate** is essential for some bacteria lacking the genes for the de novo synthesis (e.g. *S. pneumoniae*) and corresponding uptake systems exist. Likewise, **coenzyme A** synthesis has been found essential for *E. coli* and also for mycobacteria.^[548]^ Hence, biosynthesis of pantothenate and coenzyme A has been addressed by various inhibitors ranging from isosteres and other small molecules to natural products.[^548^, ^549^] In this context, **biotin** is a cofactor, e.g., of bacterial acetyl‐CoA carboxylase (AccAD, see section 3.6. on fatty acid biosynthesis), and biotin protein ligase has been considered in some approaches as a target for combatting *S. aureus*.[^550^, ^551^] However, most compounds are biotin analogs for which resistance mechanisms have been reported,^[552]^ or suffer from being single enzyme inhibitors.[^1^, ^25^]

Another pathway is the **biosynthesis of nucleotides** ^[553]^ which consists of a de novo biosynthesis and a salvage pathway. De novo biosynthesis is required, particularly in the intracellular environment of a host. This pathway is linked to energy metabolism (ATP) and cofactor synthesis (e.g., NAD(P)H), but also to DNA and RNA synthesis and virulence. A natural product targeting the biosynthesis of nucleotides is the immunosuppressant mycophenolic acid, an inhibitor of inosine‐5′‐monophosphate dehydrogenase (IMPDH), which converts IMP to GMP. Several other inhibitors are starting point analogs^[554]^ which are derived from anticancer or antiviral drugs, providing further examples of the “repurposing approach”. For instance, the anticancer drug **gemcitabine** (Figure 26 and Table 10) has been described as a terminator of DNA synthesis (replication) by misintegration, but was also found to inhibit ribonucleotide reductase (RNR) in the synthesis of deoxynucleotides.^[555]^ It shows good anti‐G+ activity^[556]^ and was even assessed in animal experiments.^[557]^ Some inhibitors of the protozoan inosine 5′‐monophosphate dehydrogenase,^[558]^ an enzyme of the guanine nucleotide biosynthesis, show anti‐G+ activity, which can be partly reversed by the presence of guanine. Using structure based‐design, a team at AstraZeneca developed **TK‐666** (Figure 26 and Table 10), a nucleobase‐type inhibitor of thymidylate kinase which showed anti‐G+ activity.^[559]^ At least for nucleoside analogs, a mechanistic assignment to nucleotide biosynthesis or DNA/RNA metabolism (compare HB‐EMAU, section 3.4.^[306]^) is complicated and partly unresolved, yet this multitarget effect on complex pathways may be an advantage.[^1^, ^25^] General problems of nucleobase‐type compounds are toxicity issues and rapid resistance development.^[554]^

**Two component systems** (TCS)^[560]^ consist of a sensing histidine kinase and a response regulator (RR) which induces gene expression in order to react to environmental stimuli. Numerous and diverse environmental stimuli activate TCSs including exposure to antibiotics. Antibiotic resistance can be induced by the resulting regulatory actions which often involve changes to cell physiology. Some of these TCS have been found to be essential, particularly for G+ bacteria, e.g., WalK/WalR of *S. aureus*. Others are rather involved in virulence, e.g. AgrC/A of *S. aureus* or QseC/QseB of *E. coli*.^[560]^ The natural product **signermycin B** (Figure 26 and Table 10)^[561]^ is a WalK inhibitor of the two‐component signal‐transduction system WalK/WalR of low GC G+ bacteria (e.g. *S. aureus*/*E. faecalis*) which regulates factors of cell wall metabolism like some autolysins. The small molecule **walrycin B** (Figure 26 and Table 10), a toxoflavin analog, inhibits WalR and shows anti‐*Sa* activity^[562]^ but the compound also activates the human pregnane X receptor.^[563]^

The class of the **oxadiazole‐type antibiotics** (similar to **ND‐421**, Figures 6, 11C and Table 1) was recently found to be active against *C. difficile*. A related **oxadiazole cpd 2** (Figure 26) derivative proved to be bactericidal to vegetative cells and inhibit **spore germination** of *C. difficile*.^[564]^ Additionally to the known PBP inhibition, this compound targets the lytic transglycosylase SleC and the pseudoprotease CspC both of which are involved in spore germination. The optimized derivative **oxadiazole cpd 57** (Figure 26) has increased activities against *C. difficile*, but more investigations regarding the MOA are required. A most recent chemical proteomics study tested a library of 252 compounds in a phenotypic screening against *S. aureus*. Oxadiazolones known to bind to reactive Ser and Cys, were identified and structurally optimized to target various different pathways: FabH (β‐ketoacyl‐acyl carrier protein synthase III), FphC (membrane‐bound serine hydrolase), AdhE (dehydrogenase, virulence factor in *E. coli*),^[565]^ and possibly FphE (serine hydrolase).^[566]^ While further studies are required to more deeply understand the MOA and to give proof‐of‐concept in animal experiments, this study also mentions the risk for non‐specific toxicology.

## 4 Conclusions and Outlook

The past decades have seen a great multitude of attempts to find new targets and new antibacterial molecules beyond the established classes. To date, the discovery process has made significant advances with improved tools and methods. Technological advancements particularly in industry enable **assay campaigns** for phenotypic, target‐based and high‐content screenings, which allow processing of enormous compound numbers. Target identification and investigation of the mode of action by molecular biology and biochemical methods follow established and comparatively straightforward workflows. The application of state‐of‐the‐art **structural biology** methods such as X‐ray crystallography, NMR spectroscopy, and more recently cryo‐electron microscopy contribute to a better understanding of molecular targets, which is the basis for structure‐based design strategies. The developments in **computer‐ and AI‐based methods** provide additional tools for hit finding and optimization. In the H2L process, **organic synthesis methods** are crucial to elucidate structure–activity‐relationships and provide hundreds if not thousands of derivatives, even of synthetically challenging structures.

The above categorization into **target classes** (Figures 11 ‐ 26 and Tables 1, 2, 3, 4, 5, 6, 7, 8, 9, 10) highlights more than 100 molecules, for which the molecular target is known for most of them, and of which most have undergone a deeper profiling of antibacterial properties, and which have given proof‐of‐concept in animal experiments. Compounds with an activity against G+ and G− key pathogens (ESKAPE(E) strains) as well as *C. difficile* and mycobacteria have been emphasized. In this context, it is worth recapitulating some **requirements for new antibacterial drugs**: Ideally, they should have broad spectrum activity, particularly against G− bacteria. However, a demonstrated unmet medical need justifies development of a compound with a narrow spectrum of activity. Hence, for cases such as *A. baumannii*, *P. aeruginosa* and *H. pylori* infections, an appropriate standby diagnostic is required, which however poses a problem for low‐income countries. An additional aspect of complexity is that the treatment regimen rather depends on medical indications (e.g. urinary tract infections (UTIs), hospital‐acquired pneumonia (HAP), etc.). Symptoms assigned to a medical indication can be caused by very different pathogenic bacteria, e.g. HAP by G+ *S. pneumoniae* but also by G− *P. aeruginosa*, which require coverage and hence treatment by an antibiotic or combinations thereof. In view of the high unmet medical need, antibacterial activities against *C. difficile* (oral administration, non‐systemic) or mycobacteria (specialized metabolism requiring long treatment periods) justify the development of a drug for these special cases.

Looking at the **discovery** process, antibacterial **phenotypic screenings** have clearly contributed the majority of the compounds reviewed, followed by **target‐based screenings**. Some **rational design approaches** stand out, such as nucleoside analogs targeting DNA polymerase, hydroxamate/non‐hydroxamate‐type LpxC inhibitors and the β‐hairpin peptidomimics binding LptD. **Structure‐based design** specifically helped to design penicillin‐binding protein inhibitor ETX0462, and in general is involved in the design of inhibitors wherever a target structure is available. Other design approaches, like the **trojan‐horse** strategy,^[153]^ the **eNTRy** rules (Debio‐1452‐NH3, fabimycin),^[171]^ **bacterial PROTACS**[^164^, ^165^] and applying computer/**AI‐based** methodology resulted in successful proof of concept and, in several cases, led to compounds showing efficacy in vivo. Besides these, future significant contributions can be expected from DNA‐encoded libraries, or from peptide‐based library compounds, particularly those employing display technologies,^[135]^ if appropriate targets are carefully selected.

A closer look at the type and origin of molecules (Tables 1, 2, 3, 4, 5, 6, 7, 8, 9, 10) reveals that among **natural products** the previously very successful **polyketides** have declined in importance as sources for new structures. Corallopyronin is an exception but it is rather more aimed at use in antiparasitic therapy. **Non‐ribosomal peptides** and also **RiPPs** are highly represented and also provide anti‐G− scaffold structures, e.g. albicidin/cystobactamid and darobactin. It goes without saying, that for natural products, chemical synthesis is crucial to further optimize the activity spectrum or pharmacokinetic properties. In this context, polyketide structures commonly are more difficult to optimize compared to peptide‐derived structures. An impressive example for a synthetic optimization of a peptide is the weakly to moderately anti‐G+ natural product arylomycin into the anti‐G− compound G0775. Previously, particularly actinobacteria have been a reliable source of natural products. More recently the genera *Photorhabdus* and *Xenorhabdus* contributed interesting new chemotypes. **Genome mining**, the **iChip** technology and **observed biological effects** yielded few, but admittedly noteworthy, antibacterials, indicating that these technologies and approaches are expected to become increasingly important in the future. Many natural products provide sophisticated MOAs, which is unlikely to be the case for small molecule approaches, and the future discovery of new scaffolds will strongly depend on research groups still continuing with natural product research.

Most **small molecules** originate from screenings by pharmaceutical companies, although nowadays, similar screening campaigns can also be set up in academic institutions using commercially available libraries or be provided by collaborations with companies. As frontrunners today are still in clinical phases, the success of small molecules as therapeutics remains to be seen, with the exception of antimycobacterial compounds which achieved noteworthy recent successes. Due to synthetic access and the feasible generation of libraries, small molecules are generally highly competitive to natural product scaffolds. A recently growing compound class are **peptides** and **peptidomimetics**, respectively. Although they share features with NRPs and RiPPs (e.g. lantibiotics) or have similarities with classic small molecules in the design and/or library screening approaches, they deserve an own classification. Molecules we would include into this classification are murepavadin, JB‐95, zosurabalpin and plectasin (defensin‐type). Even if the starting structure sometimes is a natural product, the design principles of these mostly macrocyclic peptides are based on conserved secondary‐structure elements (e.g. β‐hairpins, disulfide‐stabilized α‐β motif) and provide options for the rapid generation of big libraries for the H2L process but also lead optimization. Future impactful contributions are likely to come from phage display or RNA display technologies.[^135^, ^567^]

A glance at the anti‐G+/G− profiles of the above compiled drugs and drug candidates (Tables 1, 2, 3, 4, 5, 6, 7, 8, 9, 10) highlights the sizeable number of known anti‐G+ drugs with only a few anti‐G− compounds. This is partially due to redirection of research efforts following pressure in the 1980ies to 1990ies to find new treatments of MRSA.^[1]^ This focus is meanwhile increasingly shifting towards anti‐G− compounds. Considering target classes, novel inhibitors of **peptidoglycan biosynthesis** and **cell division** (FtsZ) with very few exceptions, e.g. ETX0462, are restricted to G+ bacteria. The same is the case for novel inhibitors of **ribosomal synthesis** (exceptions: negamycin, odilorhabdins), **DNA polymerase**, **RNA polymerase**, **fatty acid biosynthesis** (exceptions: Debio‐1452‐NH3, fabimycin) and the **proteolytic degradation** or **proteolytic processing of secretory proteins** (exception: arylomycin analog G0775). Compounds addressing **intracellular targets** also come with impediments: For instance, differences between DNA polymerases of various pathogens probably exclude the concept of a universal inhibitor. Similarly, biosynthetic pathways of fatty acids and lipids differ between pathogens and therefore inhibitors will specifically address certain strains, e.g. afabicin with anti‐*Sa* activity. Remarkably, in recent years, only one new inhibitor (pseudouridimycin) has been identified for the highly conserved RNA polymerase. Projects on helicases and ligases, which are also conserved and essential drug targets of the replisome, have been abandoned. This is in contrast to the long running target gyrase/topoisomerase IV, for which various inhibitors show a remarkable broad‐spectrum activity. Other pathways, e.g. cofactor syntheses (section 3.10.) or the mevalonate/non‐mevalonate pathway (not mentioned here) currently play a subordinate role.

**Table 1:**
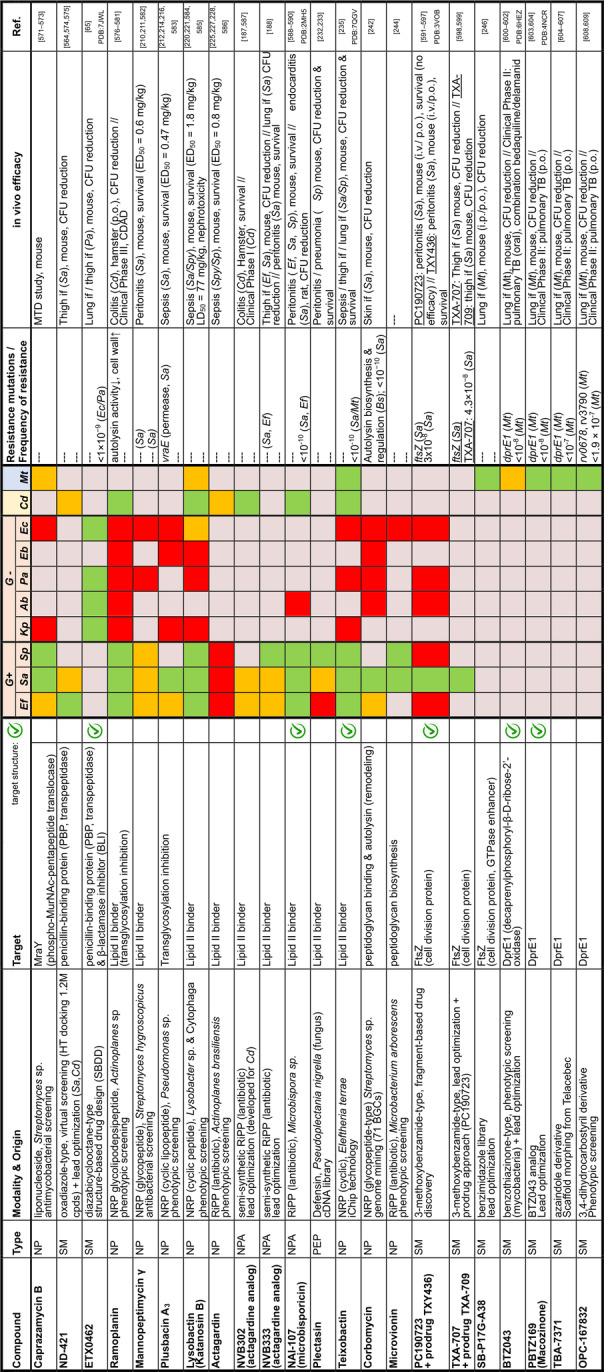
cell wall synthesis and cell division (divisome).

**Type**: NP=natural product, NPA=natural product analog, SM=small molecule, PEP=peptide; **Modality & Origin**: NRP=non‐ribosomal peptide, PK=polyketide, RiPP=ribosomally synthesized and posttranslationally modified peptide; **Target**: The availability of a structure of a target‐inhibitor complex is indicated by a green tick. If the target‐inhibitor complex was resolved only for a related compound, an asterisk was added. **Antibacterial activity**: Averaged values of antibacterial activities against ESKAPEE*/*C. difficile* (*Cd*)/*Mycobacterium tuberculosis* (*Mt*) according to published data (green≤2 μg/mL, orange 4–16 μg/mL, red≥32 μg/mL). *Enterococcus faecalis*/*faecium* (*Ef*), *S. aureus* (*Sa*), *Streptococcus pneumoniae* (*Sp*), *Klebsiella pneumoniae* (Kp), *Acinetobacter baumannii* (*Ab*), *Pseudomonas aeruginosa* (*Pa*), *Enterobacter* sp. (*Eb*), E. coli* (*Ec*), *Clostriodoides difficile* (*Cd*), *Mycobacterium tuberculosis* (*Mt*), *Helicobacter pylori* (*Hp*, G−), *Streptococcus epidermidis* (*Se*, G+), *Streptococcus pyogenes* (*Spy*, G+), *Neisseria gonorrhoeae* (*Ng*, G−) *Bacillus subtilis* (*Bs*, G+). Data on multi‐resistant strains/MIC_90_ etc. not particularly considered. **Resistance**: **Gene** (**organism**), **Frequency of resistance** (**FoR, organism**). For comparison, a resistance rate of 10^−6^ is high, 10^−8^ moderate and <10^−9^ rather low. FoR@MIC may vary. **In vivo efficacy**: infection model (bacterial strain) animal, readout (survival or reduction of colony‐forming units (CFU)). Route of administration only particularly considered for oral (p.o.) application. Thigh infection model mostly uses neutropenic animals. If applicable: most recent status on Clinical Phases I/II/III. ABSSSI=acute bacterial skin and skin structure infections; if=infection; n.d.=not disclosed; CDAD=*C. difficile*‐associated diarrhea, CDI=*C. difficile* infection.

**Table 2:**
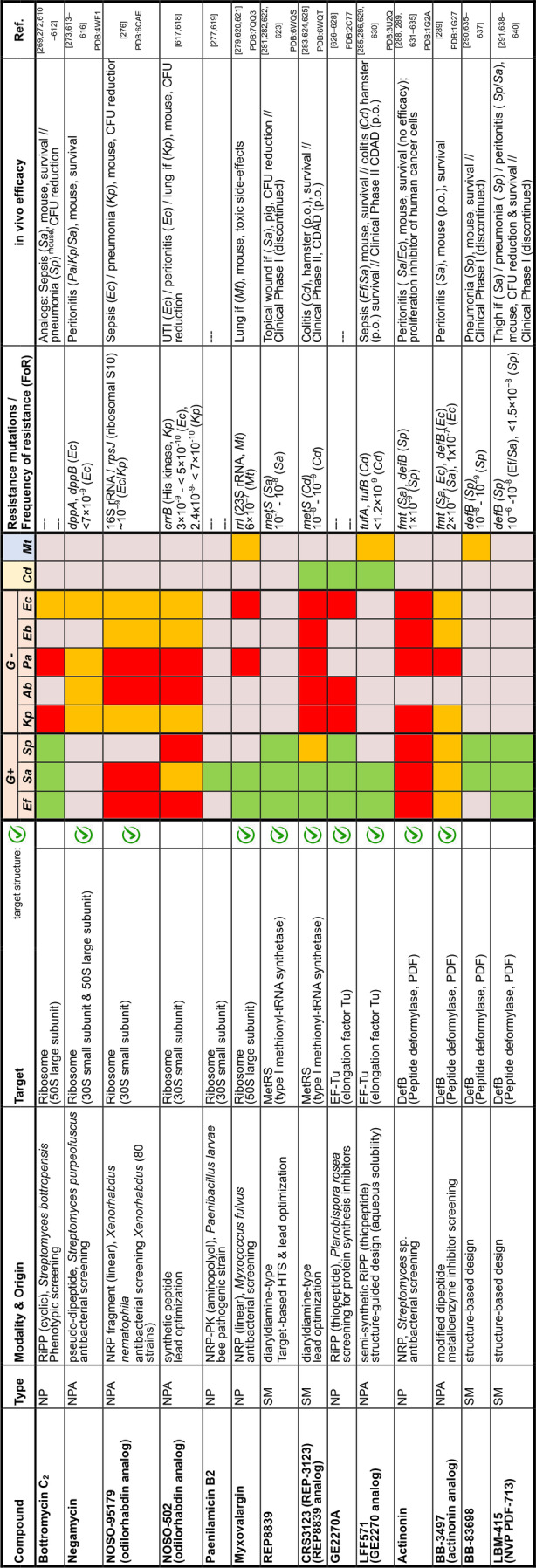
Ribosomal protein synthesis.

**Table 3:**
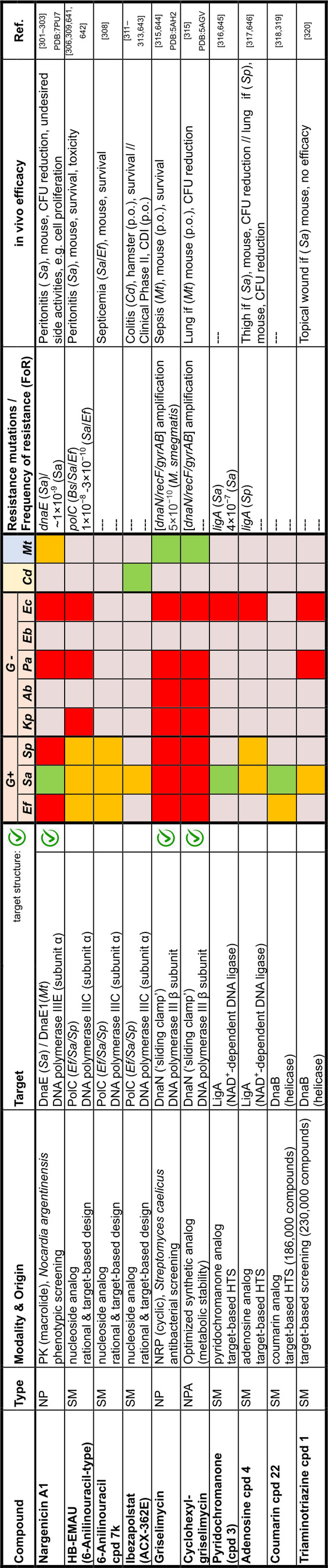
DNA polymerase.

**Table 4:**
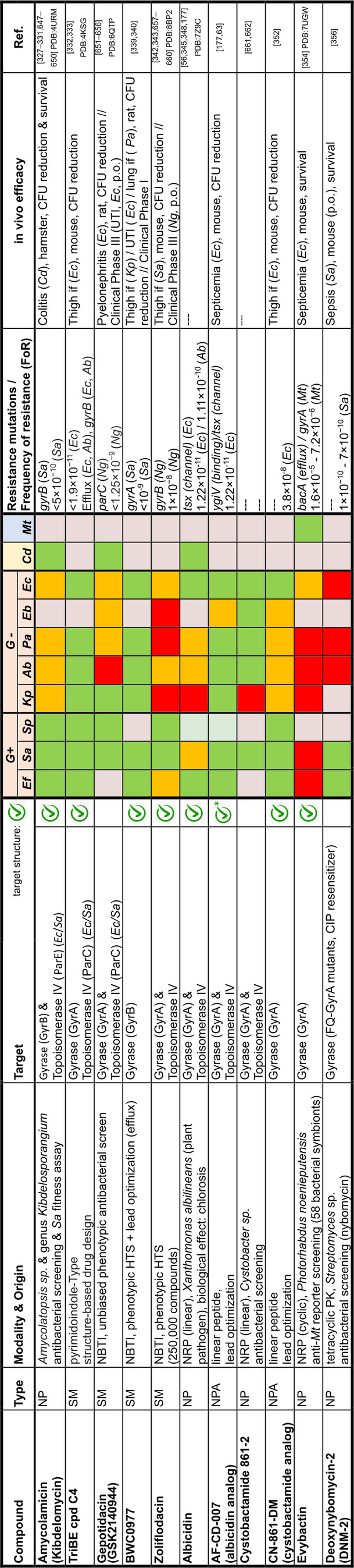
Gyrase and topoisomerase IV.

**Table 5:**
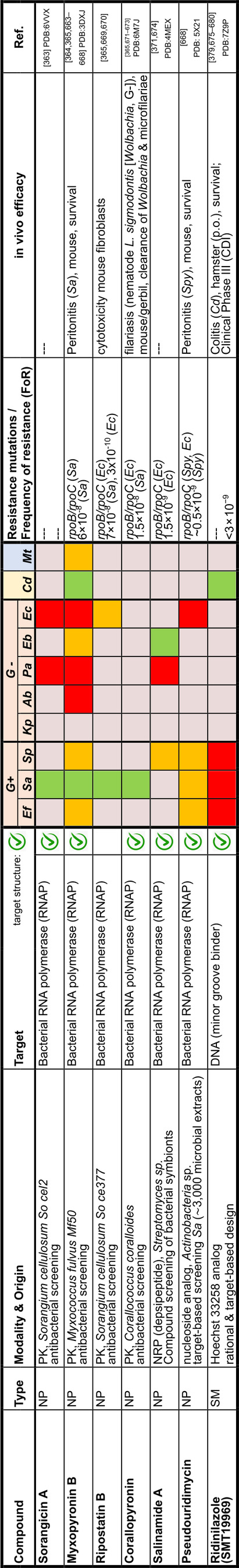
RNA synthesis.

**Table 6:**
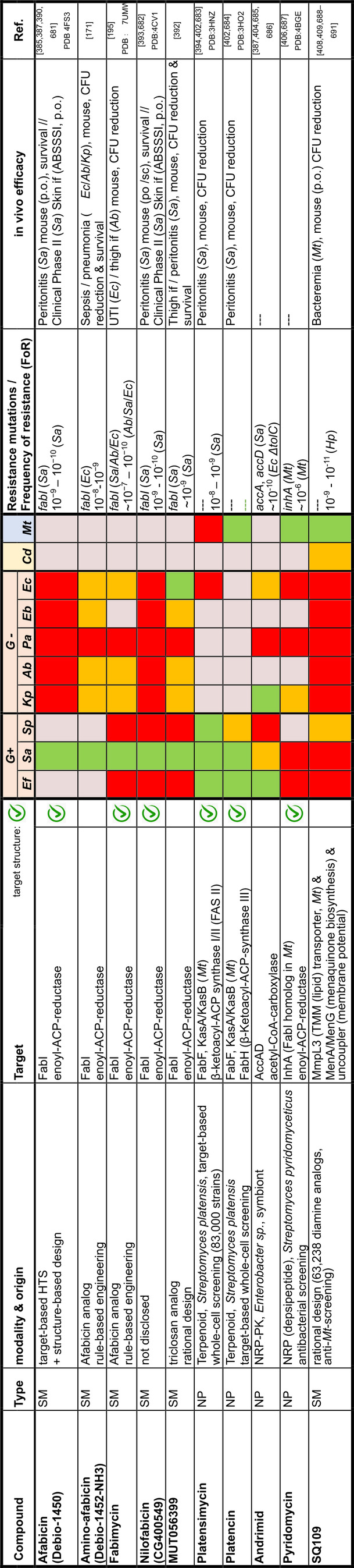
Fatty acid biosynthesis.

**Table 7:**
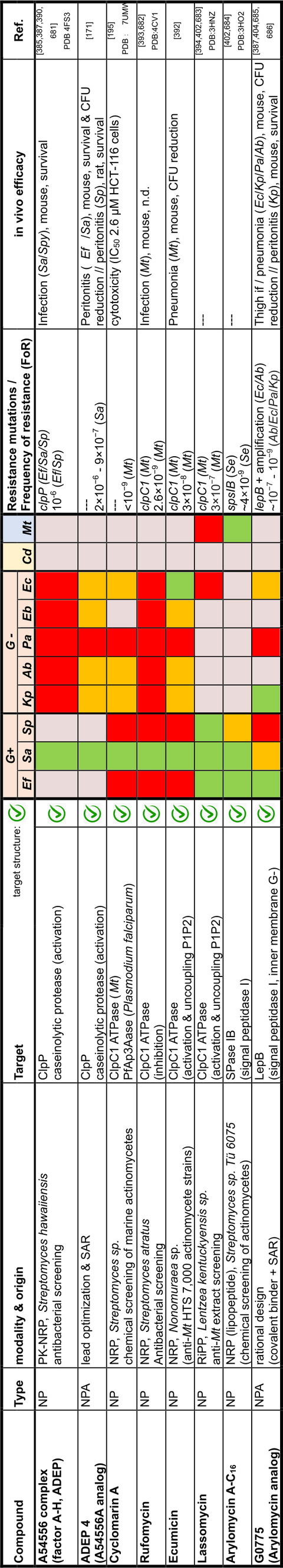
Cytoplasmic & membrane‐bound proteases and cellular degradation machineries.

**Table 8:**
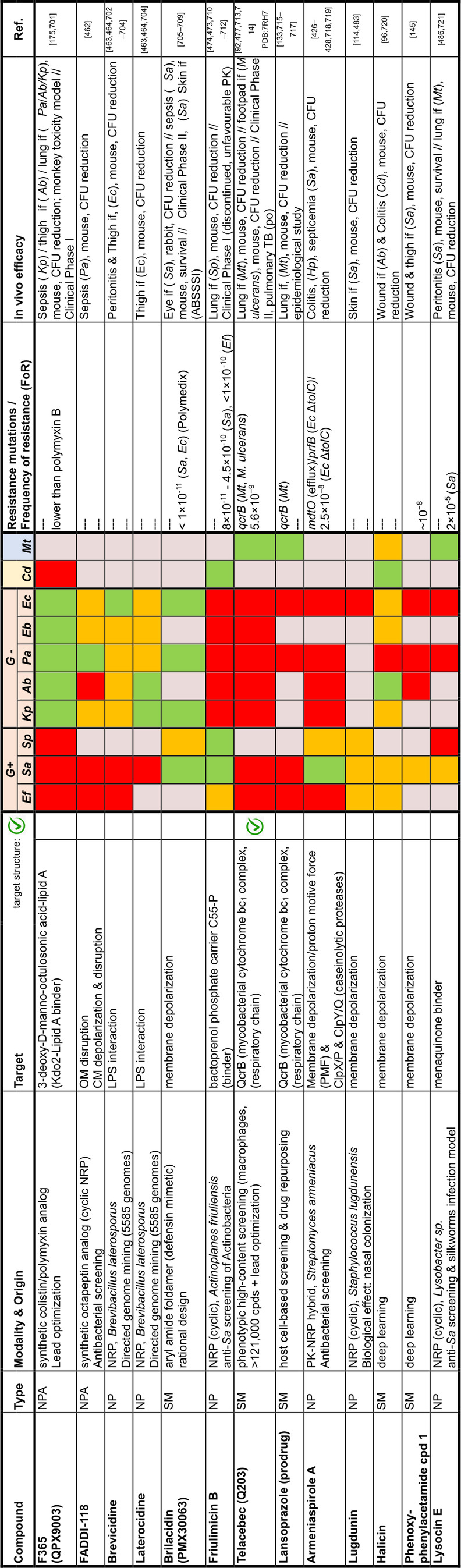
Lipid membranes and bioenergetics.

**Table 9:**
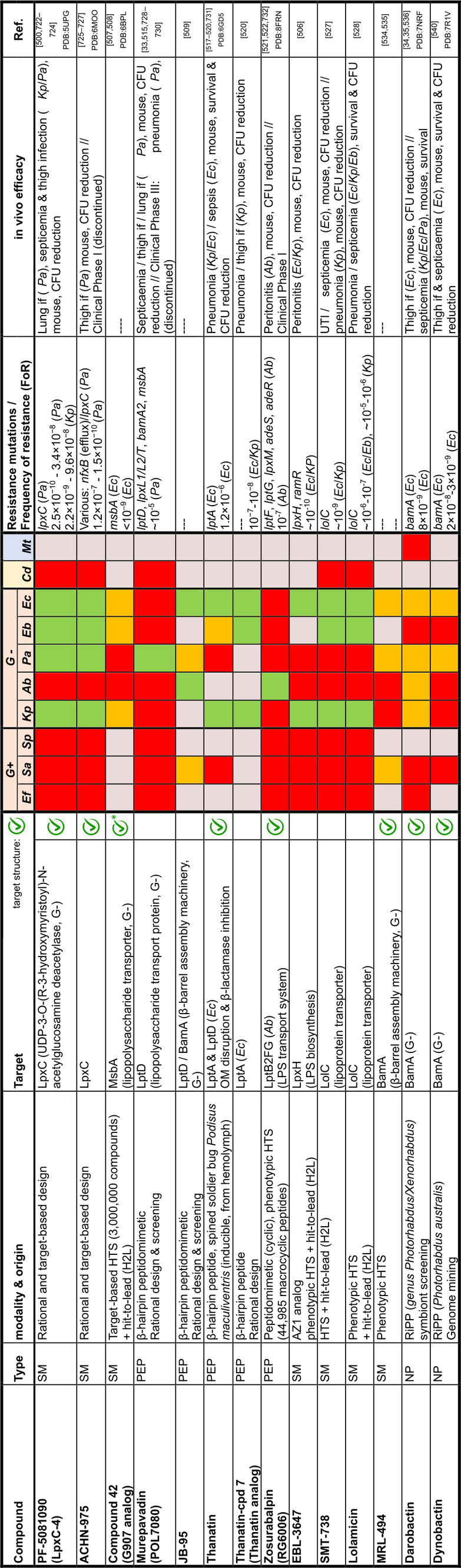
Membrane proteins and biogenesis of membrane components.

**Table 10:**
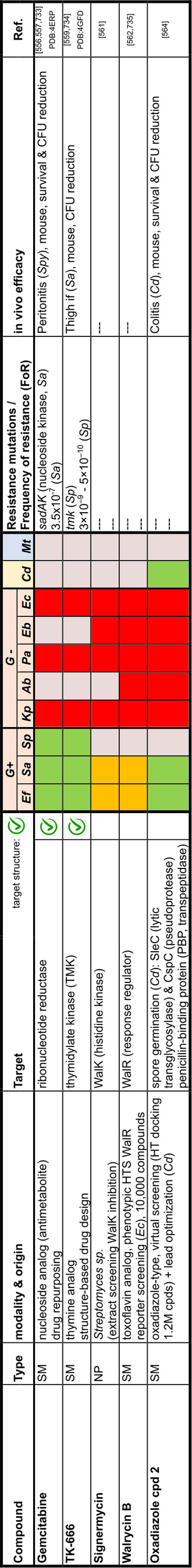
Cofactor synthesis, nucleotide synthesis and other targets.

A liability of many intracellularly acting inhibitors is membrane penetration/uptake and emerging resistance mechanisms, particularly efflux.^[6]^ This has been realized and the shift of research activities towards **extracellular or membrane‐proximal targets** has delivered new lead structures with anti‐G− activity. In this context however, compounds disrupting **lipid membranes** or interfering with **bioenergetic processes** (membrane potential & proton motive force) may be considered risky because of a narrow therapeutic window and side‐effects. Hence, this research area requires a better understanding of the modes‐of‐action of these compounds. Nevertheless, the lipopeptide‐derived compound F365, a candidate molecule sharing several characteristics with polymyxin/colistin, progressed to clinical phase I. Additionally targeted pathways unique to G− bacteria are the **lipopolysaccharide synthesis** and **export** (Lpx/MsbA/Lpt), the **β‐barrel assembly machine** (**BAM**) complex and the lipoprotein transport system between inner and outer membrane (Lol). Thus far, the initially very promising small molecule‐type LpxC inhibitors (LpxC‐4 and ACHN‐975) have failed in clinical phases. The front running peptide‐type compounds murepavadin (anti‐*Pa*) and zosurabalpin (anti‐*Ab*) targeting Lpt proteins show strain‐specificity but are basically restricted to parenteral applications, which however appears justified considering the medical need for the targeted bacteria. Lolamicin and SMT‐738 were suggested to bind to the lipoprotein‐binding site of the LolCDE‐complex in the cytoplasmic membrane and showed promising activity against some G− bacteria, particularly anti‐*Kp* activity.[^527^, ^528^] Interestingly, with arylomycin (membrane‐bound SPase) and darobactin/dynobactin (BamA) only few natural products target the membrane‐protein synthesis and the processing machinery of extracytoplasmic proteins. It is, however, too early to predict whether there are still natural compounds targeting these pathways remaining to be discovered and which small molecule and peptide classes will appear in the future.

For antibacterials against ***C. difficile*** **infections**, a considerable number of compounds have been identified, for which in vivo proof of concept has been obtained (commonly colitis hamster model). These drugs, of which many have previously been considered as antibacterials for more general use mostly against G+ pathogens, are commonly orally applied, are not systemic and act in the gut. Remarkably, in recent years various **anti‐mycobacterial drugs** have been discovered which mostly originate from research on small molecules. Besides already established targets, they specifically act on pathways of the mycobacterial membrane (e.g. KasA/B, InhA, DprE1) or affect the respiratory chain (e.g. QcrB).

The future focus on target proteins located in the membrane may be the path forward to discover new antibacterials. Interestingly, synthetic macrocycles of the peptide/peptidomimetics class have proven very successful and by exploiting library concepts may deliver further lead structures. However, in regard of the general scarcity of new and potent inhibitory scaffold structures particularly against G− bacteria, it is not wise to dispense with “old and established” targets, which may be located in the cytoplasm.

Aspects which deserve some considerations, are the reasons for **dropouts of clinical candidates** in the past. A recent article highlighting failed antibacterial compounds from established and novel drug classes spotted the transition from clinical phase I to phase II as critical.^[568]^ Hence, compounds mostly failed due to safety issues and only one due to resistance development. However, for several compounds the reasons for failure were not specified. The overall number of above examples is indeed too small to delineate further trends, e.g. whether novel compounds addressing new targets are more failure‐prone than addressing established targets.

Apart from some disengagement from antibacterial research, the advancements in the field over the past decades are still strongly impacted by contributions from industry. This is not only true for the H2L process and compound optimizations (SAR and preclinical studies), but also for the identification and characterization of molecular targets. Unfortunately, however depending on the company's philosophy and strategy, valuable knowledge and research data may remain unpublished and thus unrevealed to the scientific community. In this context donor‐funded initiatives need to be mentioned, which incentivize and support antibacterial research by academic institutions, clinics and industry in order to ensure smooth progression to clinical candidates. Examples are the Combating Antibiotic Resistant Bacteria Biopharmaceutical Accelerator (**CARB‐X**),^[569]^ or the Global Antibiotic Research & Development Partnership (**GARDP**).^[570]^ It remains to be seen, whether these efforts will result in an increased number of successes and ultimately antibacterials, which will be used in human healthcare. A main driver for R&D activities of biotech and pharma companies is the chance to earn money with their products in a reasonable timescale, but unfortunately this opportunity, compared to other therapeutic areas, still appears to be poorly attractive.

## Conflict of Interests

R.S. is a consultant to Selmod (Basel, Switzerland) and Corden Pharma International GmbH (Plankstadt, Germany).

5

## Biographical Information

*Roderich D. Süssmuth studied chemistry at the University of Tübingen and received his PhD with Professor Günther Jung. After a post‐doctoral stay with Carlos Barbas III and Richard Lerner at The Scripps Research Institute (La Jolla, CA, USA), he habilitated in Organic Chemistry and Biochemistry at Tübingen University. Since 2004, he has been the Rudolf‐Wiechert‐Professor of Biological Chemistry at the Technical University of Berlin. His research interests are the discovery and biosynthetic investigations of secondary metabolites, biological and chemical peptide synthesis, and medicinal chemistry*.

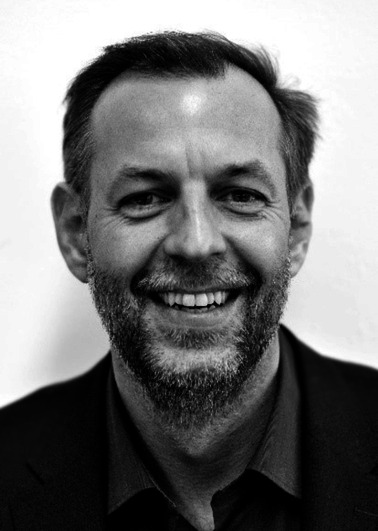

## Biographical Information

*Marcel Kulike‐Koczula was born and raised in Berlin. He studied chemistry at the Technische Universität Berlin, where he completed his master's degree. In 2020, he joined the group of Professor Roderich D. Süssmuth as doctoral student. His dissertation focuses on the synthesis and characterization of natural product analogs in the context of medicinal chemistry. His research interests include organic synthesis, antibacterials, medicinal chemistry, and molecular biology*.

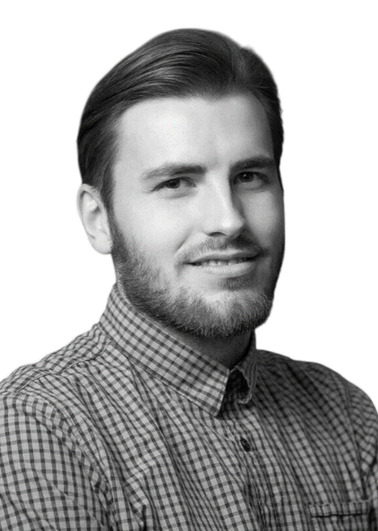

## Biographical Information

*Simone Kosol studied biology and genetics at the University of Salzburg and received her PhD in biochemistry with a focus on biomolecular NMR spectroscopy under the guidance of Prof. Klaus Zangger at the University of Graz in 2011. Before joining the group of Professor Roderich D. Süssmuth, she worked as a post‐doctoral associate at VIB Brussels with Professor Peter Tompa and at the University of Warwick with Professor Józef Lewandowski and Professor Greg Challis, where she investigated molecular interactions in natural product biosynthesis pathways. She has been a lecturer at the Medical School Berlin since 2023, teaching biochemistry and molecular biology*.

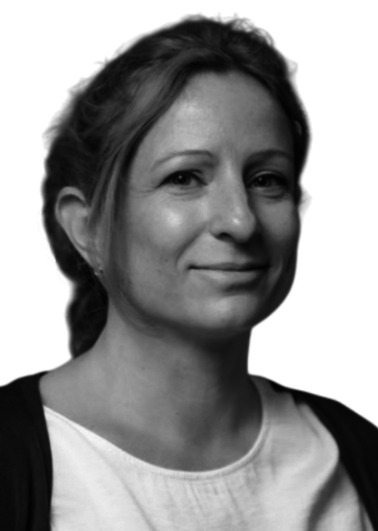

## Biographical Information

*Gao Peng studied pharmaceutical engineering in Ireland and obtained a master′s degree in organic chemistry from Kobe University in Japan in 2018. Over the next four years, he worked at several pharmaceutical companies in China, focusing on the laboratory synthesis of drugs and early‐stage process development. During this time, he completed the research and synthesis of multiple pharmaceutical projects. In 2022, he joined the group of Professor Roderich D. Süssmuth to pursue a PhD and conduct research on the development of antimicrobial compounds*.

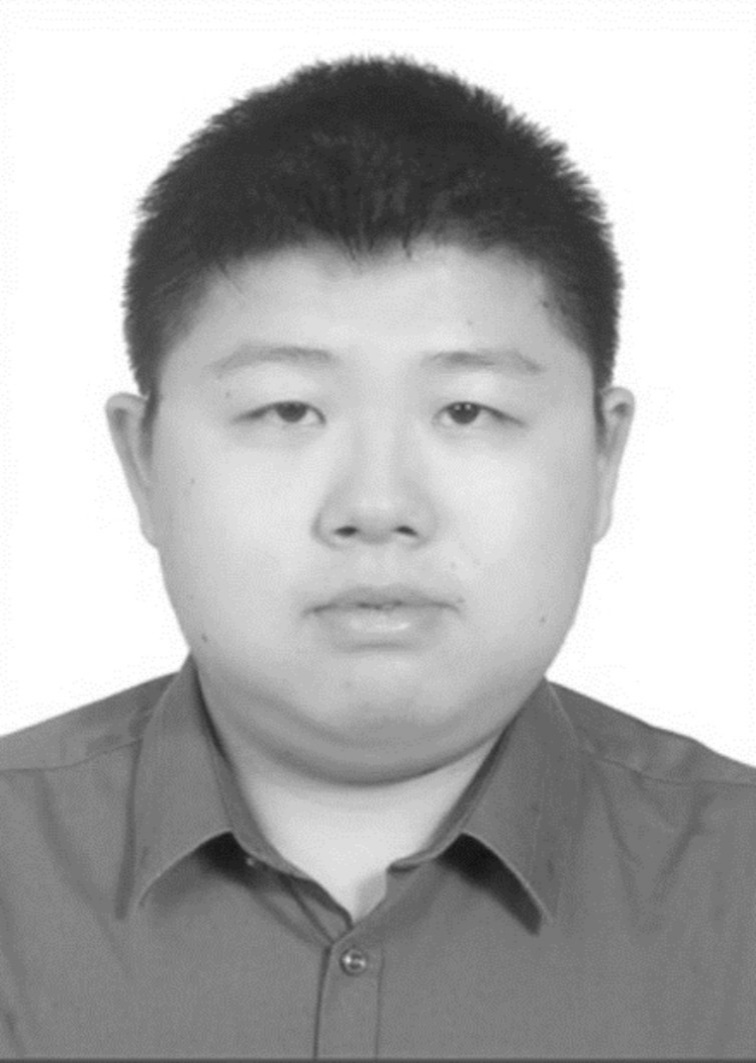

## Data Availability

Research data are not shared.

## References

[1] L. L. Silver , Cold Spring Harbor Perspect. Med. 2016, 6, a030239. 10.1101/cshperspect.a030239 PMC5131749 27599531

[2] Centers for Disease Control and Prevention (U.S.), *Antibiotic Resistance Threats in the United States, 2019*, Department of Health and Human Services, CDC, **2019**.

[3] World Health Organization, *WHO Bacterial Priority Pathogens List, 2024: Bacterial Pathogens of Public Health Importance to Guide Research, Development and Strategies to Prevent and Control Antimicrobial Resistance*, World Health Organization, **2024**.

[4] H. W. Boucher , G. H. Talbot , J. S. Bradley , J. E. Edwards , D. Gilbert , L. B. Rice , M. Scheld , B. Spellberg , J. Bartlett , Clin. Infect. Dis. 2009, 48, 1–12. 19035777 10.1086/595011

[5] L. B. Rice , J. Infect. Dis. 2008, 197, 1079–1081. 18419525 10.1086/533452

[6] H. Y. Mahmood , S. Jamshidi , J. M. Sutton , K. M. Rahman , Curr. Med. Chem. 2016, 23, 1062–1081. 26947776 10.2174/0929867323666160304150522 PMC5425656

[7] B. Młynarczyk-Bonikowska , A. Majewska , M. Malejczyk , G. Młynarczyk , S. Majewski , Med. Microbiol. Immunol. 2020, 209, 95–108. 31802195 10.1007/s00430-019-00651-4 PMC7125254

[8] J. van Prehn , E. Reigadas , E. H. Vogelzang , E. Bouza , A. Hristea , B. Guery , M. Krutova , T. Norén , F. Allerberger , J. E. Coia , A. Goorhuis , T. M. van Rossen , R. E. Ooijevaar , K. Burns , B. R. S. Olesen , S. Tschudin-Sutter , M. H. Wilcox , M. J. G. T. Vehreschild , F. Fitzpatrick , E. J. Kuijper , Clin. Microbiol. Infect. 2021, 27, S1–S21. 10.1016/j.cmi.2021.09.038 34678515

[9] O. Fleitas Martínez , M. H. Cardoso , S. M. Ribeiro , O. L. Franco , Front. Cell. Infect. Microbiol. 2019, 9. 10.3389/fcimb.2019.00074 PMC6454102 31001485

[10] S. Ruer , N. Pinotsis , D. Steadman , G. Waksman , H. Remaut , Chem. Biol. Drug Des. 2015, 86, 379–399. 25589217 10.1111/cbdd.12517

[11] S. T. Rutherford , B. L. Bassler , Cold Spring Harbor Perspect. Med. 2012, 2, a012427. 10.1101/cshperspect.a012427 PMC3543102 23125205

[12] N. Blasey , D. Rehrmann , A. K. Riebisch , S. Mühlen , Front. Cell. Infect. Microbiol. 2022, 12, 1065561. 36704108 10.3389/fcimb.2022.1065561 PMC9872159

[13] M. Iwatsuki , R. Uchida , H. Yoshijima , H. Ui , K. Shiomi , Y.-P. Kim , T. Hirose , T. Sunazuka , A. Abe , H. Tomoda , S. Omura , J. Antibiot. 2008, 61, 230–236. 10.1038/ja.2008.33 18503202

[14] N. Rabin , Y. Zheng , C. Opoku-Temeng , Y. Du , E. Bonsu , H. O. Sintim , Future Med. Chem. 2015, 7, 647–671. 25921403 10.4155/fmc.15.7

[15] S. Nadar , T. Khan , S. G. Patching , A. Omri , Microorganisms 2022, 10, 303. 35208758 10.3390/microorganisms10020303 PMC8879831

[16] R. Srinivasan , S. Santhakumari , P. Poonguzhali , M. Geetha , M. Dyavaiah , L. Xiangmin , Front. Microbiol. 2021, 12, 676458. 34054785 10.3389/fmicb.2021.676458 PMC8149761

[17] T. Maeda , R. García-Contreras , M. Pu , L. Sheng , L. R. Garcia , M. Tomás , T. K. Wood , ISME J. 2012, 6, 493–501. 21918575 10.1038/ismej.2011.122 PMC3280137

[18] R. García-Contreras , M. Martínez-Vázquez , N. Velázquez Guadarrama , A. G. Villegas Pañeda , T. Hashimoto , T. Maeda , H. Quezada , T. K. Wood , Pathog. Dis. 2013, 68, 8–11. 23620228 10.1111/2049-632X.12039

[19] R. García-Contreras , T. Maeda , T. K. Wood , ISME J. 2016, 10, 4–10. 26023871 10.1038/ismej.2015.84 PMC4681860

[20] T. Defoirdt , Trends Microbiol. 2018, 26, 313–328. 29132819 10.1016/j.tim.2017.10.005

[21] P. Krzyżek , Front. Microbiol. 2019, 10. 10.3389/fmicb.2019.02473 PMC6834643 31736912

[22] G. A. Pankey , L. D. Sabath , Clin. Infect. Dis. 2004, 38, 864–870. 14999632 10.1086/381972

[23] J. L. Martinez , F. Baquero , Antimicrob. Agents Chemother. 2000, 44, 1771–1777. 10858329 10.1128/aac.44.7.1771-1777.2000 PMC89960

[24] J. Krajewska , S. Tyski , A. E. Laudy , Antimicrob. Agents Chemother. 2023, 67, e0137322. 37022162 10.1128/aac.01373-22 PMC10190592

[25] H. Brötz-Oesterhelt , N. A. Brunner , Curr. Opin. Pharmacol. 2008, 8, 564–573. 18621146 10.1016/j.coph.2008.06.008

[26] F. von Nussbaum , M. Brands , B. Hinzen , S. Weigand , D. Häbich , Angew. Chem. Int. Ed. 2006, 45, 5072–5129; 10.1002/anie.200600350 16881035

[27] C. L. Dulberger , E. J. Rubin , C. C. Boutte , Nat. Rev. Microbiol. 2020, 18, 47–59. 31728063 10.1038/s41579-019-0273-7

[28] D. A. Gray , M. Wenzel , ACS Infect. Dis. 2020, 6, 1346–1365. 32156116 10.1021/acsinfecdis.0c00001 PMC7307902

[29] C. D. Radka , C. O. Rock , Annu. Rev. Microbiol. 2022, 76, 281–304. 35650664 10.1146/annurev-micro-041320-110408 PMC9463108

[30] R. J. Heath , C. O. Rock , Curr. Opin. Invest. Drugs 2004, 5, 146–153. PMC1618763 15043388

[31] A. Casiraghi , L. Suigo , E. Valoti , V. Straniero , Antibiotics 2020, 9, 69. 32046082 10.3390/antibiotics9020069 PMC7167804

[32] E. Culp , G. D. Wright , J. Antibiot. 2017, 70, 366–377. 10.1038/ja.2016.138 27899793

[33] N. Srinivas , P. Jetter , B. J. Ueberbacher , M. Werneburg , K. Zerbe , J. Steinmann , B. Van der Meijden , F. Bernardini , A. Lederer , R. L. A. Dias , P. E. Misson , H. Henze , J. Zumbrunn , F. O. Gombert , D. Obrecht , P. Hunziker , S. Schauer , U. Ziegler , A. Käch , L. Eberl , K. Riedel , S. J. DeMarco , J. A. Robinson , Science 2010, 327, 1010–1013. 20167788 10.1126/science.1182749

[34] Y. Imai , K. J. Meyer , A. Iinishi , Q. Favre-Godal , R. Green , S. Manuse , M. Caboni , M. Mori , S. Niles , M. Ghiglieri , C. Honrao , X. Ma , J. J. Guo , A. Makriyannis , L. Linares-Otoya , N. Böhringer , Z. G. Wuisan , H. Kaur , R. Wu , A. Mateus , A. Typas , M. M. Savitski , J. L. Espinoza , A. O'Rourke , K. E. Nelson , S. Hiller , N. Noinaj , T. F. Schäberle , A. D'Onofrio , K. Lewis , Nature 2019, 576, 459–464. 31747680 10.1038/s41586-019-1791-1 PMC7188312

[35] H. Kaur , R. P. Jakob , J. K. Marzinek , R. Green , Y. Imai , J. R. Bolla , E. Agustoni , C. V. Robinson , P. J. Bond , K. Lewis , T. Maier , S. Hiller , Nature 2021, 593, 125–129. 33854236 10.1038/s41586-021-03455-w

[36] D. A. Rasko , V. Sperandio , Nat. Rev. Drug Discovery 2010, 9, 117–128. 20081869 10.1038/nrd3013

[37] S. Mühlen , P. Dersch , Front. Cell. Infect. Microbiol. 2020, 10, 169. 32435624 10.3389/fcimb.2020.00169 PMC7218068

[38] C. K. Schmitt , K. C. Meysick , A. D. O'Brien , Emerging Infect. Dis. 1999, 5, 224–234. 10.3201/eid0502.990206 PMC2640701 10221874

[39] I. Frost , H. Sati , P. Garcia-Vello , M. Hasso-Agopsowicz , C. Lienhardt , V. Gigante , P. Beyer , Lancet Microbe 2023, 4, e113–e125. 36528040 10.1016/S2666-5247(22)00303-2 PMC9892012

[40] S. X. Wang-Lin , J. P. Balthasar , Antibodies 2018, 7, 5. 31544858 10.3390/antib7010005 PMC6698815

[41] M. P. Motley , K. Banerjee , B. C. Fries , Curr. Opin. Infect. Dis. 2019, 32, 210–216. 30950853 10.1097/QCO.0000000000000539 PMC7050834

[42] P. Orth , L. Xiao , L. D. Hernandez , P. Reichert , P. R. Sheth , M. Beaumont , X. Yang , N. Murgolo , G. Ermakov , E. DiNunzio , F. Racine , J. Karczewski , S. Secore , R. N. Ingram , T. Mayhood , C. Strickland , A. G. Therien , J. Biol. Chem. 2014, 289, 18008–18021. 24821719 10.1074/jbc.M114.560748 PMC4140266

[43] R. Dehbanipour , Z. Ghalavand , Germs 2022, 12, 262–275. 36504617 10.18683/germs.2022.1328 PMC9719373

[44] J. O'Neill, Tackling Drug-Resistant Infections Globally: Final Report and Recommendations. Government of the United Kingdom, can be found under https://amr-review.org/, **2016** (accessed 28 November 2024).

[45] C. J. L. Murray , K. S. Ikuta , F. Sharara , L. Swetschinski , G. R. Aguilar , A. Gray , C. Han , C. Bisignano , P. Rao , E. Wool , S. C. Johnson , A. J. Browne , M. G. Chipeta , F. Fell , S. Hackett , G. Haines-Woodhouse , B. H. K. Hamadani , E. A. P. Kumaran , B. McManigal , S. Achalapong , R. Agarwal , S. Akech , S. Albertson , J. Amuasi , J. Andrews , A. Aravkin , E. Ashley , F.-X. Babin , F. Bailey , S. Baker , B. Basnyat , A. Bekker , R. Bender , J. A. Berkley , A. Bethou , J. Bielicki , S. Boonkasidecha , J. Bukosia , C. Carvalheiro , C. Castañeda-Orjuela , V. Chansamouth , S. Chaurasia , S. Chiurchiù , F. Chowdhury , R. C. Donatien , A. J. Cook , B. Cooper , T. R. Cressey , E. Criollo-Mora , M. Cunningham , S. Darboe , N. P. J. Day , M. D. Luca , K. Dokova , A. Dramowski , S. J. Dunachie , T. D. Bich , T. Eckmanns , D. Eibach , A. Emami , N. Feasey , N. Fisher-Pearson , K. Forrest , C. Garcia , D. Garrett , P. Gastmeier , A. Z. Giref , R. C. Greer , V. Gupta , S. Haller , A. Haselbeck , S. I. Hay , M. Holm , S. Hopkins , Y. Hsia , K. C. Iregbu , J. Jacobs , D. Jarovsky , F. Javanmardi , A. W. J. Jenney , M. Khorana , S. Khusuwan , N. Kissoon , E. Kobeissi , T. Kostyanev , F. Krapp , R. Krumkamp , A. Kumar , H. H. Kyu , C. Lim , K. Lim , D. Limmathurotsakul , M. J. Loftus , M. Lunn , J. Ma , A. Manoharan , F. Marks , J. May , M. Mayxay , N. Mturi , T. Munera-Huertas , P. Musicha , L. A. Musila , M. M. Mussi-Pinhata , R. N. Naidu , T. Nakamura , R. Nanavati , S. Nangia , P. Newton , C. Ngoun , A. Novotney , D. Nwakanma , C. W. Obiero , T. J. Ochoa , A. Olivas-Martinez , P. Olliaro , E. Ooko , E. Ortiz-Brizuela , P. Ounchanum , G. D. Pak , J. L. Paredes , A. Y. Peleg , C. Perrone , T. Phe , K. Phommasone , N. Plakkal , A. Ponce-de-Leon , M. Raad , T. Ramdin , S. Rattanavong , A. Riddell , T. Roberts , J. V. Robotham , A. Roca , V. D. Rosenthal , K. E. Rudd , N. Russell , H. S. Sader , W. Saengchan , J. Schnall , J. A. G. Scott , S. Seekaew , M. Sharland , M. Shivamallappa , J. Sifuentes-Osornio , A. J. Simpson , N. Steenkeste , A. J. Stewardson , T. Stoeva , N. Tasak , A. Thaiprakong , G. Thwaites , C. Tigoi , C. Turner , P. Turner , H. R. van Doorn , S. Velaphi , A. Vongpradith , M. Vongsouvath , H. Vu , T. Walsh , J. L. Walson , S. Waner , T. Wangrangsimakul , P. Wannapinij , T. Wozniak , T. E. M. W. Y. Sharma , K. C. Yu , P. Zheng , B. Sartorius , A. D. Lopez , A. Stergachis , C. Moore , C. Dolecek , M. Naghavi , The Lancet 2022, 399, 629–655.

[46] K. Bush , P. A. Bradford , Clin. Microbiol. Rev. 2020, 33, e00047–19. 32102899 10.1128/CMR.00047-19 PMC7048014

[47] J. Vila , J. Ruiz , P. Goñi , M. T. De Anta , Antimicrob. Agents Chemother. 1996, 40, 491–493. 8834907 10.1128/aac.40.2.491 PMC163143

[48] A.-P. Magiorakos , A. Srinivasan , R. B. Carey , Y. Carmeli , M. E. Falagas , C. G. Giske , S. Harbarth , J. F. Hindler , G. Kahlmeter , B. Olsson-Liljequist , D. L. Paterson , L. B. Rice , J. Stelling , M. J. Struelens , A. Vatopoulos , J. T. Weber , D. L. Monnet , Clin. Microbiol. Infect. 2012, 18, 268–281. 21793988 10.1111/j.1469-0691.2011.03570.x

[49] B. Van den Bergh , M. Fauvart , J. Michiels , FEMS Microbiol. Rev. 2017, 41, 219–251. 28333307 10.1093/femsre/fux001

[50] A. Baran , A. Kwiatkowska , L. Potocki , Int. J. Mol. Sci. 2023, 24, 5777. 36982857 10.3390/ijms24065777 PMC10056106

[51] E. M. Darby , E. Trampari , P. Siasat , M. S. Gaya , I. Alav , M. A. Webber , J. M. A. Blair , Nat. Rev. Microbiol. 2023, 21, 280–295. 36411397 10.1038/s41579-022-00820-y

[52] W. C. Reygaert , AIMS Microbiol. 2018, 4, 482–501. 31294229 10.3934/microbiol.2018.3.482 PMC6604941

[53] S. M. Hashimi , G. Huang , A. Maxwell , R. G. Birch , Antimicrob. Agents Chemother. 2008, 52, 1382–1390. 18268084 10.1128/AAC.01551-07 PMC2292561

[54] J. M. Bostock , G. Huang , S. M. Hashimi , L. Zhang , R. G. Birch , J. Appl. Microbiol. 2006, 101, 151–160. 16834602 10.1111/j.1365-2672.2006.02899.x

[55] S. M. Hashimi , M. K. Wall , A. B. Smith , A. Maxwell , R. G. Birch , Antimicrob. Agents Chemother. 2007, 51, 181–187. 17074789 10.1128/AAC.00918-06 PMC1797663

[56] R. G. Birch , J. M. Pemberton , W. V. S. Basnayake , Microbiology 1990, 136, 51–58. 10.1099/00221287-136-1-51 2191080

[57] L. Zhang , R. G. Birch , Proc. Natl. Acad. Sci. USA 1997, 94, 9984–9989. 9275238 10.1073/pnas.94.18.9984 PMC23319

[58] L. Vieweg , J. Kretz , A. Pesic , D. Kerwat , S. Grätz , M. Royer , S. Cociancich , A. Mainz , R. D. Süssmuth , J. Am. Chem. Soc. 2015, 137, 7608–7611. 26057615 10.1021/jacs.5b04099

[59] M. J. Walker , R. G. Birch , J. M. Pemberton , Mol. Microbiol. 1988, 2, 443–454. 2845223 10.1111/j.1365-2958.1988.tb00050.x

[60] W. V. Shiromi Basnayake , R. G. Birch , Microbiology 1995, 141, 551–560. 7711894 10.1099/13500872-141-3-551

[61] L. Rostock , R. Driller , S. Grätz , D. Kerwat , L. von Eckardstein , D. Petras , M. Kunert , C. Alings , F.-J. Schmitt , T. Friedrich , M. C. Wahl , B. Loll , A. Mainz , R. D. Süssmuth , Nat. Commun. 2018, 9, 3095. 30082794 10.1038/s41467-018-05551-4 PMC6078987

[62] S. Kosol , L. Rostock , J. Barsig , T. Tabarelli , K. Hommernick , M. Kulike , T. Eulberg , M. Seidel , I. Behroz , L. Kleebauer , S. Grätz , A. Mainz , R. D. Süssmuth , Chem. Sci. 2023, 14, 5069–5078. 37206387 10.1039/d3sc00955f PMC10189885

[63] M. Saathoff , S. Kosol , T. Semmler , K. Tedin , N. Dimos , J. Kupke , M. Seidel , F. Ghazisaeedi , M. C. Jonske , S. A. Wolf , B. Kuropka , W. Czyszczoń , D. Ghilarov , S. Grätz , J. G. Heddle , B. Loll , R. D. Süssmuth , M. Fulde , PLoS Biol. 2023, 21, e3002186. 37561817 10.1371/journal.pbio.3002186 PMC10414762

[64] M. F. Mojica , M.-A. Rossi , A. J. Vila , R. A. Bonomo , Lancet Infect. Dis. 2022, 22, e28–e34. 34246322 10.1016/S1473-3099(20)30868-9 PMC8266270

[65] T. F. Durand-Reville , A. A. Miller , J. P. O'Donnell , X. Wu , M. A. Sylvester , S. Guler , R. Iyer , A. B. Shapiro , N. M. Carter , C. Velez-Vega , S. H. Moussa , S. M. McLeod , A. Chen , A. M. Tanudra , J. Zhang , J. Comita-Prevoir , J. A. Romero , H. Huynh , A. D. Ferguson , P. S. Horanyi , S. J. Mayclin , H. S. Heine , G. L. Drusano , J. E. Cummings , R. A. Slayden , R. A. Tommasi , Nature 2021, 597, 698–702. 34526714 10.1038/s41586-021-03899-0

[66] G. Luci , F. Mattioli , M. Falcone , A. Di Paolo , Antibiotics 2021, 10, 769. 34202609 10.3390/antibiotics10070769 PMC8300739

[67] B. Liu , R. E. L. Trout , G.-H. Chu , D. McGarry , R. W. Jackson , J. C. Hamrick , D. M. Daigle , S. M. Cusick , C. Pozzi , F. De Luca , M. Benvenuti , S. Mangani , J.-D. Docquier , W. J. Weiss , D. C. Pevear , L. Xerri , C. J. Burns , J. Med. Chem. 2020, 63, 2789–2801. 31765155 10.1021/acs.jmedchem.9b01518 PMC7104248

[68] J. Brem , T. Panduwawala , J. U. Hansen , J. Hewitt , E. Liepins , P. Donets , L. Espina , A. J. M. Farley , K. Shubin , G. G. Campillos , P. Kiuru , S. Shishodia , D. Krahn , R. K. Leśniak , J. Schmidt (Adrian) , K. Calvopiña , M.-C. Turrientes , M. E. Kavanagh , D. Lubriks , P. Hinchliffe , G. W. Langley , A. F. Aboklaish , A. Eneroth , M. Backlund , A. G. Baran , E. I. Nielsen , M. Speake , J. Kuka , J. Robinson , S. Grinberga , L. Robinson , M. A. McDonough , A. M. Rydzik , T. M. Leissing , J. C. Jimenez-Castellanos , M. B. Avison , S. Da Silva Pinto , A. D. Pannifer , M. Martjuga , E. Widlake , M. Priede , I. Hopkins Navratilova , M. Gniadkowski , A. K. Belfrage , P. Brandt , J. Yli-Kauhaluoma , E. Bacque , M. G. P. Page , F. Björkling , J. M. Tyrrell , J. Spencer , P. A. Lang , P. Baranczewski , R. Cantón , S. P. McElroy , P. S. Jones , F. Baquero , E. Suna , A. Morrison , T. R. Walsh , C. J. Schofield , Nat. Chem. 2022, 14, 15–24. 34903857 10.1038/s41557-021-00831-x

[69] O. Lomovskaya , M. S. Warren , A. Lee , J. Galazzo , R. Fronko , M. Lee , J. Blais , D. Cho , S. Chamberland , T. Renau , R. Leger , S. Hecker , W. Watkins , K. Hoshino , H. Ishida , V. J. Lee , Antimicrob. Agents Chemother. 2001, 45, 105–116. 11120952 10.1128/AAC.45.1.105-116.2001 PMC90247

[70] S. Tran , L. Lebreuilly , D. Cormontagne , S. Samson , T. Tô , R. Dervyn , A. Grießhammer , J. de la Cuesta-Zuluaga , L. Maier , T. Naas , S. Mura , J. Nicolas , D. Rognan , G. André , N. Ramarao , bioRxiv preprint 2024, DOI: 10.1101/2024.01.22.576688.

[71] T. A. Le , T. Hiba , D. Chaudhari , A. N. Preston , Z. R. Palowsky , S. Ahmadzadeh , S. Shekoohi , E. M. Cornett , A. D. Kaye , Adv. Ther. 2023, 40, 1357–1365. 36738370 10.1007/s12325-023-02436-x

[72] Y. Khaliq , G. G. Zhanel , Clin. Infect. Dis. 2003, 36, 1404–1410. 12766835 10.1086/375078

[73] E. C. Böttger , B. Springer , T. Prammananan , Y. Kidan , P. Sander , EMBO Rep. 2001, 2, 318–323. 11306553 10.1093/embo-reports/kve062 PMC1083859

[74] G. B. Stefano , J. Samuel , R. M. Kream , Med. Sci. Monit. 2017, 23, 101–106. 28063266 10.12659/MSM.899478 PMC5240889

[75] M. A. Kohanski , D. J. Dwyer , B. Hayete , C. A. Lawrence , J. J. Collins , Cell 2007, 130, 797–810. 17803904 10.1016/j.cell.2007.06.049

[76] S. Kalghatgi , C. S. Spina , J. C. Costello , M. Liesa , J. R. Morones-Ramirez , S. Slomovic , A. Molina , O. S. Shirihai , J. J. Collins , Sci. Transl. Med. 2013, 5, 192ra85–192ra85. 10.1126/scitranslmed.3006055 PMC3760005 23825301

[77] A. M. Tomé , A. Filipe , Drug Saf. 2011, 34, 465–488. 21585220 10.2165/11587280-000000000-00000

[78] S. Povea-Cabello , M. Álvarez-Córdoba , I. Villalón-García , M. Talaverón-Rey , A. Suárez-Carrillo , M. Munuera-Cabeza , Biomol. Eng. 2021, 11, 1050. 10.3390/biom11071050 PMC8301944 34356674

[79] A. Palleja , K. H. Mikkelsen , S. K. Forslund , A. Kashani , K. H. Allin , T. Nielsen , T. H. Hansen , S. Liang , Q. Feng , C. Zhang , P. T. Pyl , L. P. Coelho , H. Yang , J. Wang , A. Typas , M. F. Nielsen , H. B. Nielsen , P. Bork , J. Wang , T. Vilsbøll , T. Hansen , F. K. Knop , M. Arumugam , O. Pedersen , Nat. Microbiol. 2018, 3, 1255–1265. 30349083 10.1038/s41564-018-0257-9

[80] M. Willmann , M. J. G. T. Vehreschild , L. M. Biehl , W. Vogel , D. Dörfel , A. Hamprecht , H. Seifert , I. B. Autenrieth , S. Peter , BMC Biol. 2019, 17, 76. 31533707 10.1186/s12915-019-0692-y PMC6749691

[81] B. W. Haak , J. M. Lankelma , F. Hugenholtz , C. Belzer , W. M. de Vos , W. J. Wiersinga , J. Antimicrob. Chemother. 2019, 74, 782–786. 30418539 10.1093/jac/dky471

[82] M. J. Blaser , Science 2016, 352, 544–545. 27126037 10.1126/science.aad9358 PMC4939477

[83] K. Korpela , A. Salonen , L. J. Virta , R. A. Kekkonen , K. Forslund , P. Bork , W. M. de Vos , Nat. Commun. 2016, 7, 10410. 26811868 10.1038/ncomms10410 PMC4737757

[84] A. E. Clatworthy , E. Pierson , D. T. Hung , Nat. Chem. Biol. 2007, 3, 541–548. 17710100 10.1038/nchembio.2007.24

[85] S. W. Dickey , G. Y. C. Cheung , M. Otto , Nat. Rev. Drug Discovery 2017, 16, 457–471. 28337021 10.1038/nrd.2017.23 PMC11849574

[86] T. Avis , F. X. Wilson , N. Khan , C. S. Mason , D. J. Powell , Drug Discovery Today 2021, 26, 2198–2203. 34329771 10.1016/j.drudis.2021.07.016

[87] S. B. Singh , K. Young , L. Miesel , Expert Rev. Anti-Infect. Ther. 2011, 9, 589–613. 21819327 10.1586/eri.11.81

[88] N. Aulner , A. Danckaert , J. Ihm , D. Shum , S. L. Shorte , Trends Parasitol. 2019, 35, 559–570. 31176583 10.1016/j.pt.2019.05.004

[89] D. J. Payne , M. N. Gwynn , D. J. Holmes , D. L. Pompliano , Nat. Rev. Drug Discovery 2007, 6, 29–40. 17159923 10.1038/nrd2201

[90] R. Tommasi , D. G. Brown , G. K. Walkup , J. I. Manchester , A. A. Miller , Nat. Rev. Drug Discovery 2015, 14, 529–542. 26139286 10.1038/nrd4572

[91] B. L. Staker , G. W. Buchko , P. J. Myler , Curr. Opin. Microbiol. 2015, 27, 133–138. 26458180 10.1016/j.mib.2015.09.003 PMC4659754

[92] K. Pethe , P. Bifani , J. Jang , S. Kang , S. Park , S. Ahn , J. Jiricek , J. Jung , H. K. Jeon , J. Cechetto , T. Christophe , H. Lee , M. Kempf , M. Jackson , A. J. Lenaerts , H. Pham , V. Jones , M. J. Seo , Y. M. Kim , M. Seo , J. J. Seo , D. Park , Y. Ko , I. Choi , R. Kim , S. Y. Kim , S. Lim , S.-A. Yim , J. Nam , H. Kang , H. Kwon , C.-T. Oh , Y. Cho , Y. Jang , J. Kim , A. Chua , B. H. Tan , M. B. Nanjundappa , S. P. S. Rao , W. S. Barnes , R. Wintjens , J. R. Walker , S. Alonso , S. Lee , J. Kim , S. Oh , T. Oh , U. Nehrbass , S.-J. Han , Z. No , J. Lee , P. Brodin , S.-N. Cho , K. Nam , J. Kim , Nat. Med. 2013, 19, 1157–1160. 23913123 10.1038/nm.3262

[93] V. Blay , B. Tolani , S. P. Ho , M. R. Arkin , Drug Discovery Today 2020, 25, 1807–1821. 32801051 10.1016/j.drudis.2020.07.024

[94] P. I. O'Daniel , Z. Peng , H. Pi , S. A. Testero , D. Ding , E. Spink , E. Leemans , M. A. Boudreau , T. Yamaguchi , V. A. Schroeder , W. R. Wolter , L. I. Llarrull , W. Song , E. Lastochkin , M. Kumarasiri , N. T. Antunes , M. Espahbodi , K. Lichtenwalter , M. A. Suckow , S. Vakulenko , S. Mobashery , M. Chang , J. Am. Chem. Soc. 2014, 136, 3664–3672. 24517363 10.1021/ja500053x PMC3985699

[95] J. Janardhanan , M. Chang , S. Mobashery , Curr. Opin. Microbiol. 2016, 33, 13–17. 27239942 10.1016/j.mib.2016.05.009 PMC5069118

[96] J. M. Stokes , K. Yang , K. Swanson , W. Jin , A. Cubillos-Ruiz , N. M. Donghia , C. R. MacNair , S. French , L. A. Carfrae , Z. Bloom-Ackermann , V. M. Tran , A. Chiappino-Pepe , A. H. Badran , I. W. Andrews , E. J. Chory , G. M. Church , E. D. Brown , T. S. Jaakkola , R. Barzilay , J. J. Collins , Cell 2020, 180, 688–702.e13. 32084340 10.1016/j.cell.2020.01.021 PMC8349178

[97] M. A. Farha , E. D. Brown , Nat. Prod. Rep. 2016, 33, 668–680. 26806527 10.1039/c5np00127g

[98] R. G. K. Donald , S. Skwish , R. A. Forsyth , J. W. Anderson , T. Zhong , C. Burns , S. Lee , X. Meng , L. LoCastro , L. W. Jarantow , J. Martin , S. H. Lee , I. Taylor , D. Robbins , C. Malone , L. Wang , C. S. Zamudio , P. J. Youngman , J. W. Phillips , Chem. Biol. 2009, 16, 826–836. 19716473 10.1016/j.chembiol.2009.07.004

[99] G. D. Amoutzias , M. Nikolaidis , A. Hesketh , Microorganisms 2022, 10, 1040. 35630482 10.3390/microorganisms10051040 PMC9148168

[100] J. Herrmann , A. A. Fayad , R. Müller , Nat. Prod. Rep. 2017, 34, 135–160. 27907217 10.1039/c6np00106h

[101] R. D. Süssmuth , A. Mainz , Angew. Chem. Int. Ed. 2017, 56, 3770–3821; 10.1002/anie.201609079 28323366

[102] A. Nivina , K. P. Yuet , J. Hsu , C. Khosla , Chem. Rev. 2019, 119, 12524–12547. 31838842 10.1021/acs.chemrev.9b00525 PMC6935866

[103] M. Montalbán-López , T. A. Scott , S. Ramesh , I. R. Rahman , A. J. van Heel , J. H. Viel , V. Bandarian , E. Dittmann , O. Genilloud , Y. Goto , M. J. G. Burgos , C. Hill , S. Kim , J. Koehnke , J. A. Latham , A. J. Link , B. Martínez , S. K. Nair , Y. Nicolet , S. Rebuffat , H.-G. Sahl , D. Sareen , E. W. Schmidt , L. Schmitt , K. Severinov , R. D. Süssmuth , A. W. Truman , H. Wang , J.-K. Weng , G. P. van Wezel , Q. Zhang , J. Zhong , J. Piel , D. A. Mitchell , O. P. Kuipers , W. A. van der Donk , Nat. Prod. Rep. 2021, 38, 130–239. 32935693 10.1039/d0np00027b PMC7864896

[104] A. G. Atanasov , S. B. Zotchev , V. M. Dirsch , C. T. Supuran , Nat. Rev. Drug Discovery 2021, 20, 200–216. 33510482 10.1038/s41573-020-00114-z PMC7841765

[105] B. M. Hover , S.-H. Kim , M. Katz , Z. Charlop-Powers , J. G. Owen , M. A. Ternei , J. Maniko , A. B. Estrela , H. Molina , S. Park , D. S. Perlin , S. F. Brady , Nat. Microbiol. 2018, 3, 415–422. 29434326 10.1038/s41564-018-0110-1 PMC5874163

[106] M. T. Henke , N. L. Kelleher , Nat. Prod. Rep. 2016, 33, 942–950. 27376415 10.1039/c6np00024j PMC4981503

[107] O. Genilloud , I. González , O. Salazar , J. Martín , J. R. Tormo , F. Vicente , J. Ind. Microbiol. Biotechnol. 2011, 38, 375–389. 20931260 10.1007/s10295-010-0882-7

[108] J. Rajkumari , M. Dyavaiah , A. Syed , B. Siddhardha , in Model Organisms for Microbial Pathogenesis, Biofilm Formation and Antimicrobial Drug Discovery (Eds.: B. Siddhardha , M. Dyavaiah , A. Syed ), Springer, Singapore, 2020, pp. 527–543.

[109] O. Genilloud , Curr. Opin. Microbiol. 2019, 51, 81–87. 31739283 10.1016/j.mib.2019.10.012

[110] H. Cimen , M. Touray , S. H. Gulsen , S. Hazir , Appl. Microbiol. Biotechnol. 2022, 106, 4387–4399. 35723692 10.1007/s00253-022-12023-9

[111] N. Boemare , R. Akhurst , in The Prokaryotes: A Handbook on the Biology of Bacteria Volume 6: Proteobacteria: Gamma Subclass (Eds.: M. Dworkin , S. Falkow , E. Rosenberg , K.-H. Schleifer , E. Stackebrandt ), Springer, New York, NY, 2006, pp. 451–494.

[112] E. Tortorella , P. Tedesco , F. Palma Esposito , G. G. January , R. Fani , M. Jaspars , D. de Pascale , Mar. Drugs 2018, 16, 355. 30274274 10.3390/md16100355 PMC6213577

[113] C. K. Okoro , R. Brown , A. L. Jones , B. A. Andrews , J. A. Asenjo , M. Goodfellow , A. T. Bull , Antonie van Leeuwenhoek 2009, 95, 121–133. 19052913 10.1007/s10482-008-9295-2

[114] A. Zipperer , M. C. Konnerth , C. Laux , A. Berscheid , D. Janek , C. Weidenmaier , M. Burian , N. A. Schilling , C. Slavetinsky , M. Marschal , M. Willmann , H. Kalbacher , B. Schittek , H. Brötz-Oesterhelt , S. Grond , A. Peschel , B. Krismer , Nature 2016, 535, 511–516. 27466123 10.1038/nature18634

[115] B. C. Covington , F. Xu , M. R. Seyedsayamdost , Annu. Rev. Biochem. 2021, 90, 763–788. 33848426 10.1146/annurev-biochem-081420-102432 PMC9148385

[116] J. T. Staley , A. Konopka , Annu. Rev. Microbiol. 1985, 39, 321–346. 3904603 10.1146/annurev.mi.39.100185.001541

[117] D. Nichols , N. Cahoon , E. M. Trakhtenberg , L. Pham , A. Mehta , A. Belanger , T. Kanigan , K. Lewis , S. S. Epstein , Appl. Environ. Microbiol. 2010, 76, 2445–2450. 20173072 10.1128/AEM.01754-09 PMC2849220

[118] F. Maglangit , Y. Yu , H. Deng , Nat. Prod. Rep. 2021, 38, 782–821. 33119013 10.1039/d0np00061b

[119] R. G. Birch , S. S. Patil , J. Gen. Microbiol. 1985, 131, 1069–1075. 2410547 10.1099/00221287-131-5-1069

[120] M. Sorokina , C. Steinbeck , J. Cheminf. 2020, 12, 20. 10.1186/s13321-020-00424-9 PMC7118820 33431011

[121] O. N. Sekurova , O. Schneider , S. B. Zotchev , Microb. Biotechnol. 2019, 12, 828–844. 30834674 10.1111/1751-7915.13398 PMC6680616

[122] S. F. Brady , C. J. Chao , J. Clardy , J. Am. Chem. Soc. 2002, 124, 9968–9969. 12188643 10.1021/ja0268985

[123] V. Libis , N. Antonovsky , M. Zhang , Z. Shang , D. Montiel , J. Maniko , M. A. Ternei , P. Y. Calle , C. Lemetre , J. G. Owen , S. F. Brady , Nat. Commun. 2019, 10, 3848. 31451725 10.1038/s41467-019-11658-z PMC6710260

[124] K. D. Bauman , K. S. Butler , B. S. Moore , J. R. Chekan , Nat. Prod. Rep. 2021, 38, 2100–2129. 34734626 10.1039/d1np00032b PMC8597713

[125] M. H. Medema , K. Blin , P. Cimermancic , V. de Jager , P. Zakrzewski , M. A. Fischbach , T. Weber , E. Takano , R. Breitling , Nucleic Acids Res. 2011, 39, W339–346. 21672958 10.1093/nar/gkr466 PMC3125804

[126] M. A. Skinnider , C. W. Johnston , M. Gunabalasingam , N. J. Merwin , A. M. Kieliszek , R. J. MacLellan , H. Li , M. R. M. Ranieri , A. L. H. Webster , M. P. T. Cao , A. Pfeifle , N. Spencer , Q. H. To , D. P. Wallace , C. A. Dejong , N. A. Magarvey , Nat. Commun. 2020, 11, 6058. 33247171 10.1038/s41467-020-19986-1 PMC7699628

[127] M. Alanjary , B. Kronmiller , M. Adamek , K. Blin , T. Weber , D. Huson , B. Philmus , N. Ziemert , Nucleic Acids Res. 2017, 45, W42–W48. 28472505 10.1093/nar/gkx360 PMC5570205

[128] K. Blin , S. Shaw , M. H. Medema , T. Weber , Nucleic Acids Res. 2024, 52, D586–D589. 37904617 10.1093/nar/gkad984 PMC10767862

[129] K. Andries , P. Verhasselt , J. Guillemont , H. W. H. Göhlmann , J.-M. Neefs , H. Winkler , J. Van Gestel , P. Timmerman , M. Zhu , E. Lee , P. Williams , D. de Chaffoy , E. Huitric , S. Hoffner , E. Cambau , C. Truffot-Pernot , N. Lounis , V. Jarlier , Science 2005, 307, 223–227. 15591164 10.1126/science.1106753

[130] A. Miró-Canturri , R. Ayerbe-Algaba , Y. Smani , Front. Microbiol. 2019, 10 : 41. 10.3389/fmicb.2019.00041 PMC6360151 30745898

[131] P. Le , E. Kunold , R. Macsics , K. Rox , M. C. Jennings , I. Ugur , M. Reinecke , D. Chaves-Moreno , M. W. Hackl , C. Fetzer , F. A. M. Mandl , J. Lehmann , V. S. Korotkov , S. M. Hacker , B. Kuster , I. Antes , D. H. Pieper , M. Rohde , W. M. Wuest , E. Medina , S. A. Sieber , Nat. Chem. 2020, 12, 145–158. 31844194 10.1038/s41557-019-0378-7 PMC6994260

[132] S. Jacques , A. M. van der Sloot , C. C. Huard , J. Coulombe-Huntington , S. Tsao , S. Tollis , T. Bertomeu , E. J. Culp , D. Pallant , M. A. Cook , E. Bonneil , P. Thibault , G. D. Wright , M. Tyers , Genetics 2020, 214, 1103–1120. 32094149 10.1534/genetics.119.302851 PMC7153937

[133] J. Rybniker , A. Vocat , C. Sala , P. Busso , F. Pojer , A. Benjak , S. T. Cole , Nat. Commun. 2015, 6, 7659. 26158909 10.1038/ncomms8659 PMC4510652

[134] W. G. Cochrane , P. R. Fitzgerald , B. M. Paegel , ACS Chem. Biol. 2021, 16, 2752–2756. 34806373 10.1021/acschembio.1c00714 PMC8688339

[135] C. Sohrabi , A. Foster , A. Tavassoli , Nat. Chem. Rev. 2020, 4, 90–101. 10.1038/s41570-019-0159-2 37128052

[136] Y. Fan , R. Feng , X. Zhang , Z.-L. Wang , F. Xiong , S. Zhang , Z.-F. Zhong , H. Yu , Q.-W. Zhang , Z. Zhang , Y. Wang , G. Li , Acta Pharm. Sin. B 2024, 14, 3362–3384. 39220863 10.1016/j.apsb.2024.04.006 PMC11365444

[137] P. 't Hart , T. M. Wood , K. H. M. Ebrahim Tehrani , R. M. van Harten , M. Śleszyńska , I. R. Rebollo , A. P. A. Hendrickx , R. J. L. Willems , E. Breukink , N. I. Martin , Chem. Sci. 2017, 8, 7991–7997. 29568446 10.1039/c7sc03413j PMC5853558

[138] M. S. Newton , Y. Cabezas , B. Seelig , ACS Synth. Biol. 2020, 9, 181–190. 31891492 10.1021/acssynbio.9b00419 PMC8203280

[139] E. Stefan , R. Obexer , S. Hofmann , K. Vu Huu , Y. Huang , N. Morgner , H. Suga , R. Tampé , eLife 2021, 10, e67732. 33929325 10.7554/eLife.67732 PMC8116058

[140] J. M. Rogers , H. Suga , Org. Biomol. Chem. 2015, 13, 9353–9363. 26280393 10.1039/c5ob01336d

[141] S. R. Fleming , T. E. Bartges , A. A. Vinogradov , C. L. Kirkpatrick , Y. Goto , H. Suga , L. M. Hicks , A. A. Bowers , J. Am. Chem. Soc. 2019, 141, 758–762. 30602112 10.1021/jacs.8b11521 PMC6642631

[142] A. Frei , J. Zuegg , A. G. Elliott , M. Baker , S. Braese , C. Brown , F. Chen , C. G. Dowson , G. Dujardin , N. Jung , A. P. King , A. M. Mansour , M. Massi , J. Moat , H. A. Mohamed , A. K. Renfrew , P. J. Rutledge , P. J. Sadler , M. H. Todd , C. E. Willans , J. J. Wilson , M. A. Cooper , M. A. T. Blaskovich , Chem. Sci. 2020, 11, 2627–2639. 32206266 10.1039/c9sc06460e PMC7069370

[143] E. Spink , D. Ding , Z. Peng , M. A. Boudreau , E. Leemans , E. Lastochkin , W. Song , K. Lichtenwalter , P. I. O'Daniel , S. A. Testero , H. Pi , V. A. Schroeder , W. R. Wolter , N. T. Antunes , M. A. Suckow , S. Vakulenko , M. Chang , S. Mobashery , J. Med. Chem. 2015, 58, 1380–1389. 25590813 10.1021/jm501661f PMC6863074

[144] G. Liu , D. B. Catacutan , K. Rathod , K. Swanson , W. Jin , J. C. Mohammed , A. Chiappino-Pepe , S. A. Syed , M. Fragis , K. Rachwalski , J. Magolan , M. G. Surette , B. K. Coombes , T. Jaakkola , R. Barzilay , J. J. Collins , J. M. Stokes , Nat. Chem. Biol. 2023, 19, 1342–1350. 37231267 10.1038/s41589-023-01349-8

[145] F. Wong , E. J. Zheng , J. A. Valeri , N. M. Donghia , M. N. Anahtar , S. Omori , A. Li , A. Cubillos-Ruiz , A. Krishnan , W. Jin , A. L. Manson , J. Friedrichs , R. Helbig , B. Hajian , D. K. Fiejtek , F. F. Wagner , H. H. Soutter , A. M. Earl , J. M. Stokes , L. D. Renner , J. J. Collins , Nature 2024, 626, 177–185. 38123686 10.1038/s41586-023-06887-8 PMC10866013

[146] J. Nüesch , F. Knüsel , in Mechanism of Action (Eds.: D. Gottlieb , P. D. Shaw ), Springer, Berlin, Heidelberg, 1967, pp. 499–541.

[147] A. Hartmann , H.-P. Fiedler , V. Braun , Eur. J. Biochem. 1979, 99, 517–524. 387415 10.1111/j.1432-1033.1979.tb13283.x

[148] B. K. Bhuyan , in Antibiotics: Volume I Mechanism of Action (Eds.: D. Gottlieb , P. D. Shaw ), Springer, Berlin, Heidelberg, 1967, pp. 153–155.

[149] G. F. Gause , Br. Med. J. 1955, 2, 1177–1179. 13269824 10.1136/bmj.2.4949.1177 PMC1981213

[150] H. Maehr , R. G. Pitcher , J. Antibiot. 1971, 24, 830–834. 10.7164/antibiotics.24.830 5140530

[151] G. Benz , T. Schröder , J. Kurz , C. Wünsche , W. Karl , G. Steffens , J. Pfitzner , D. Schmidt , Angew. Chem. Int. Ed. 1982, 21, 527–528;

[152] H. Zähner , H. Diddens , W. Keller-Schierlein , H. U. Nägeli , Jpn. J. Antibiot. 1977, 30 Suppl, 201–206. 612705

[153] M. J. Miller , R. Liu , Acc. Chem. Res. 2021, 54, 1646–1661. 33684288 10.1021/acs.accounts.1c00004 PMC9262095

[154] M. G. P. Page , Ann. N. Y. Acad. Sci. 2013, 1277, 115–126. 23346861 10.1111/nyas.12024

[155] M. G. P. Page , C. Dantier , E. Desarbre , Antimicrob. Agents Chemother. 2010, 54, 2291–2302. 20308379 10.1128/AAC.01525-09 PMC2876421

[156] B. Hofer , C. Dantier , K. Gebhardt , E. Desarbre , A. Schmitt-Hoffmann , M. G. P. Page , J. Antimicrob. Chemother. 2013, 68, 1120–1129. 23344577 10.1093/jac/dks527

[157] M. Straubinger , H. Blenk , K. G. Naber , F. M. E. Wagenlehner , Antimicrob. Agents Chemother. 2016, 60, 3309–3315. 26976871 10.1128/AAC.02425-15 PMC4879366

[158] F. Paech , S. Messner , J. Spickermann , M. Wind , A.-H. Schmitt-Hoffmann , A. T. Witschi , B. A. Howell , R. J. Church , J. Woodhead , M. Engelhardt , S. Krähenbühl , M. Maurer , Arch. Toxicol. 2017, 91, 3647–3662. 28536862 10.1007/s00204-017-1994-x

[159] M. W. McCarthy , Drugs Today 2020, 56, 177–184. 10.1358/dot.2020.56.3.3118466 32282864

[160] A. Ito , T. Sato , M. Ota , M. Takemura , T. Nishikawa , S. Toba , N. Kohira , S. Miyagawa , N. Ishibashi , S. Matsumoto , R. Nakamura , M. Tsuji , Y. Yamano , Antimicrob. Agents Chemother. 2017, 62, 1–11. 10.1128/AAC.01454-17 PMC5740388 29061741

[161] Y. R. Lee , S. Yeo , Clin. Drug Invest. 2020, 40, 901–913. 10.1007/s40261-020-00955-x PMC7374078 32700154

[162] D. Greenwood , F. O'Grady , Antimicrob. Agents Chemother. 1976, 10, 249–252. 791095 10.1128/aac.10.2.249 PMC429730

[163] R. Domalaon , T. Idowu , G. G. Zhanel , F. Schweizer , Clin. Microbiol. Rev. 2018, 31, 1–45. 10.1128/CMR.00077-17 PMC5967690 29540434

[164] F. E. Morreale , S. Kleine , J. Leodolter , S. Junker , D. M. Hoi , S. Ovchinnikov , A. Okun , J. Kley , R. Kurzbauer , L. Junk , S. Guha , D. Podlesainski , U. Kazmaier , G. Boehmelt , H. Weinstabl , K. Rumpel , V. M. Schmiedel , M. Hartl , D. Haselbach , A. Meinhart , M. Kaiser , T. Clausen , Cell 2022, 185, 2338–2353.e18. 35662409 10.1016/j.cell.2022.05.009 PMC9240326

[165] D. M. Hoi , S. Junker , L. Junk , K. Schwechel , K. Fischel , D. Podlesainski , P. M. E. Hawkins , L. van Geelen , F. Kaschani , J. Leodolter , F. E. Morreale , S. Kleine , S. Guha , K. Rumpel , V. M. Schmiedel , H. Weinstabl , A. Meinhart , R. J. Payne , M. Kaiser , M. Hartl , G. Boehmelt , U. Kazmaier , R. Kalscheuer , T. Clausen , Cell 2023, 186, 2176–2192.e22. 37137307 10.1016/j.cell.2023.04.009

[166] L. Junk , V. M. Schmiedel , S. Guha , K. Fischel , P. Greb , K. Vill , V. Krisilia , L. van Geelen , K. Rumpel , P. Kaur , R. V. Krishnamurthy , S. Narayanan , R. K. Shandil , M. Singh , C. Kofink , A. Mantoulidis , P. Biber , G. Gmaschitz , U. Kazmaier , A. Meinhart , J. Leodolter , D. Hoi , S. Junker , F. E. Morreale , T. Clausen , R. Kalscheuer , H. Weinstabl , G. Boehmelt , Nat. Commun. 2024, 15, 2005. 38443338 10.1038/s41467-024-46218-7 PMC10914731

[167] A. Lavecchia , Drug Discovery Today 2015, 20, 318–331. 25448759 10.1016/j.drudis.2014.10.012

[168] R. O'Shea , H. E. Moser , J. Med. Chem. 2008, 51, 2871–2878. 18260614 10.1021/jm700967e

[169] L. L. Silver , Bioorg. Med. Chem. 2016, 24, 6379–6389. 27381365 10.1016/j.bmc.2016.06.044

[170] M. F. Richter , P. J. Hergenrother , Ann. N. Y. Acad. Sci. 2019, 1435, 18–38. 29446459 10.1111/nyas.13598 PMC6093809

[171] E. N. Parker , B. S. Drown , E. J. Geddes , H. Y. Lee , N. Ismail , G. W. Lau , P. J. Hergenrother , Nat. Microbiol. 2020, 5, 67–75. 31740764 10.1038/s41564-019-0604-5 PMC6953607

[172] M. F. Richter , B. S. Drown , A. P. Riley , A. Garcia , T. Shirai , R. L. Svec , P. J. Hergenrother , Nature 2017, 545, 299–304. 28489819 10.1038/nature22308 PMC5737020

[173] S. J. Perlmutter , E. J. Geddes , B. S. Drown , S. E. Motika , M. R. Lee , P. J. Hergenrother , ACS Infect. Dis. 2021, 7, 162–173. 33228356 10.1021/acsinfecdis.0c00715 PMC7796962

[174] N. Haloi , A. K. Vasan , E. J. Geddes , A. Prasanna , P.-C. Wen , W. W. Metcalf , P. J. Hergenrother , E. Tajkhorshid , Chem. Sci. 2021, 12, 15028–15044. 34909143 10.1039/d1sc04445a PMC8612397

[175] K. D. Roberts , Y. Zhu , M. A. K. Azad , M.-L. Han , J. Wang , L. Wang , H. H. Yu , A. S. Horne , J.-A. Pinson , D. Rudd , N. H. Voelcker , N. A. Patil , J. Zhao , X. Jiang , J. Lu , K. Chen , O. Lomovskaya , S. J. Hecker , P. E. Thompson , R. L. Nation , M. N. Dudley , D. C. Griffith , T. Velkov , J. Li , Nat. Commun. 2022, 13, 1625. 35338128 10.1038/s41467-022-29234-3 PMC8956739

[176] J. Kretz , D. Kerwat , V. Schubert , S. Grätz , A. Pesic , S. Semsary , S. Cociancich , M. Royer , R. D. Süssmuth , Angew. Chem. Int. Ed. 2015, 54, 1969–1973; 10.1002/anie.201409584 25504839

[177] L. Zborovsky , L. Kleebauer , M. Seidel , A. Kostenko , L. von Eckardstein , F. O. Gombert , J. Weston , R. D. Süssmuth , Chem. Sci. 2021, 12, 14606–14617. 34881013 10.1039/d1sc04019g PMC8580050

[178] T. C. Roberts , P. A. Smith , R. T. Cirz , F. E. Romesberg , J. Am. Chem. Soc. 2007, 129, 15830–15838. 18052061 10.1021/ja073340u

[179] P. A. Smith , M. F. T. Koehler , H. S. Girgis , D. Yan , Y. Chen , Y. Chen , J. J. Crawford , M. R. Durk , R. I. Higuchi , J. Kang , J. Murray , P. Paraselli , S. Park , W. Phung , J. G. Quinn , T. C. Roberts , L. Rougé , J. B. Schwarz , E. Skippington , J. Wai , M. Xu , Z. Yu , H. Zhang , M.-W. Tan , C. E. Heise , Nature 2018, 561, 189–194. 30209367 10.1038/s41586-018-0483-6

[180] Z.-C. Wu , D. L. Boger , Acc. Chem. Res. 2020, 53, 2587–2599. 33138354 10.1021/acs.accounts.0c00569 PMC7674238

[181] Q. Li , J. Pellegrino , D. J. Lee , A. A. Tran , H. A. Chaires , R. Wang , J. E. Park , K. Ji , D. Chow , N. Zhang , A. F. Brilot , J. T. Biel , G. van Zundert , K. Borrelli , D. Shinabarger , C. Wolfe , B. Murray , M. P. Jacobson , E. Mühle , O. Chesneau , J. S. Fraser , I. B. Seiple , Nature 2020, 586, 145–150. 32968273 10.1038/s41586-020-2761-3 PMC7546582

[182] M. J. Mitcheltree , A. Pisipati , E. A. Syroegin , K. J. Silvestre , D. Klepacki , J. D. Mason , D. W. Terwilliger , G. Testolin , A. R. Pote , K. J. Y. Wu , R. P. Ladley , K. Chatman , A. S. Mankin , Y. S. Polikanov , A. G. Myers , Nature 2021, 599, 507–512. 34707295 10.1038/s41586-021-04045-6 PMC8549432

[183] M. G. Charest , C. D. Lerner , J. D. Brubaker , D. R. Siegel , A. G. Myers , Science 2005, 308, 395–398. 15831754 10.1126/science.1109755

[184] C. Sun , Q. Wang , J. D. Brubaker , P. M. Wright , C. D. Lerner , K. Noson , M. Charest , D. R. Siegel , Y.-M. Wang , A. G. Myers , J. Am. Chem. Soc. 2008, 130, 17913–17927. 19053822 10.1021/ja806629e PMC2681267

[185] I. B. Seiple , Z. Zhang , P. Jakubec , A. Langlois-Mercier , P. M. Wright , D. T. Hog , K. Yabu , S. R. Allu , T. Fukuzaki , P. N. Carlsen , Y. Kitamura , X. Zhou , M. L. Condakes , F. T. Szczypiński , W. D. Green , A. G. Myers , Nature 2016, 533, 338–345. 27193679 10.1038/nature17967 PMC6526944

[186] E. van Groesen , P. Innocenti , N. I. Martin , ACS Infect. Dis. 2022, 8, 1381–1407. 35895325 10.1021/acsinfecdis.2c00253 PMC9379927

[187] S. Boakes , M. J. Dawson , in Natural Products, John Wiley & Sons, Ltd, 2014, pp. 455–468.

[188] S. Boakes , W. J. Weiss , M. Vinson , S. Wadman , M. J. Dawson , J. Antibiot. 2016, 69, 850–857. 10.1038/ja.2016.47 27189121

[189] J. J. Zhang , X. Tang , B. S. Moore , Nat. Prod. Rep. 2019, 36, 1313–1332. 31197291 10.1039/c9np00025a PMC6750982

[190] K. T. Nguyen , D. Ritz , J.-Q. Gu , D. Alexander , M. Chu , V. Miao , P. Brian , R. H. Baltz , Proc. Natl. Acad. Sci. USA 2006, 103, 17462–17467. 17090667 10.1073/pnas.0608589103 PMC1859951

[191] A. Rittner , M. Joppe , J. J. Schmidt , L. M. Mayer , S. Reiners , E. Heid , D. Herzberg , D. H. Sherman , M. Grininger , Nat. Chem. 2022, 14, 1000–1006. 35879443 10.1038/s41557-022-00996-z PMC9832397

[192] L. Foulston , Curr. Opin. Microbiol. 2019, 51, 1–8. 30776510 10.1016/j.mib.2019.01.001

[193] Z. Wang , B. Koirala , Y. Hernandez , M. Zimmerman , S. F. Brady , Science 2022, 376, 991–996. 35617397 10.1126/science.abn4213 PMC10904332

[194] Z. Wang , B. Koirala , Y. Hernandez , M. Zimmerman , S. Park , D. S. Perlin , S. F. Brady , Nature 2022, 601, 606–611. 34987225 10.1038/s41586-021-04264-x PMC10321319

[195] E. N. Parker , B. N. Cain , B. Hajian , R. J. Ulrich , E. J. Geddes , S. Barkho , H. Y. Lee , J. D. Williams , M. Raynor , D. Caridha , A. Zaino , M. Shekhar , K. A. Muñoz , K. M. Rzasa , E. R. Temple , D. Hunt , X. Jin , C. Vuong , K. Pannone , A. M. Kelly , M. P. Mulligan , K. K. Lee , G. W. Lau , D. T. Hung , P. J. Hergenrother , ACS Cent. Sci. 2022, 8, 1145–1158. 36032774 10.1021/acscentsci.2c00598 PMC9413440

[196] M. Matsumoto , H. Hashizume , T. Tomishige , M. Kawasaki , H. Tsubouchi , H. Sasaki , Y. Shimokawa , M. Komatsu , PLoS Med. 2006, 3, e466. 17132069 10.1371/journal.pmed.0030466 PMC1664607

[197] A. J. F. Egan , J. Errington , W. Vollmer , Nat. Rev. Microbiol. 2020, 18, 446–460. 32424210 10.1038/s41579-020-0366-3

[198] T. den Blaauwen , L. W. Hamoen , P. A. Levin , Curr. Opin. Microbiol. 2017, 36, 85–94. 28254403 10.1016/j.mib.2017.01.007 PMC6436919

[199] S. Du , J. Lutkenhaus , Trends Microbiol. 2019, 27, 781–791. 31171437 10.1016/j.tim.2019.04.011 PMC6831097

[200] M. A. Kohanski , D. J. Dwyer , J. J. Collins , Nat. Rev. Microbiol. 2010, 8, 423–435. 20440275 10.1038/nrmicro2333 PMC2896384

[201] H. Barreteau , A. Kovač , A. Boniface , M. Sova , S. Gobec , D. Blanot , FEMS Microbiol. Rev. 2008, 32, 168–207. 18266853 10.1111/j.1574-6976.2008.00104.x

[202] Y. Ishizaki , Y. Takahashi , T. Kimura , M. Inoue , C. Hayashi , M. Igarashi , J. Antibiot. 2019, 72, 970–980. 10.1038/s41429-019-0225-5 31471594

[203] R. A. Pitner , P. G. Durham , I. E. Stewart , S. G. Reed , G. H. Cassell , A. J. Hickey , D. Carter , J. Pharm. Sci. 2019, 108, 3302–3311. 31152746 10.1016/j.xphs.2019.05.024 PMC6759370

[204] K. Kimura , J. Antibiot. 2019, 72, 877–889. 10.1038/s41429-019-0241-5 31582803

[205] A. Mistry , M. S. Warren , J. K. Cusick , R. R. Karkhoff-Schweizer , O. Lomovskaya , H. P. Schweizer , Antimicrob. Agents Chemother. 2013, 57, 5565–5571. 23979749 10.1128/AAC.01198-13 PMC3811240

[206] P. A. Mann , A. Müller , L. Xiao , P. M. Pereira , C. Yang , S. Ho Lee , H. Wang , J. Trzeciak , J. Schneeweis , M. M. dos Santos , N. Murgolo , X. She , C. Gill , C. J. Balibar , M. Labroli , J. Su , A. Flattery , B. Sherborne , R. Maier , C. M. Tan , T. Black , K. Önder , S. Kargman , F. J. Jr. Monsma , M. G. Pinho , T. Schneider , T. Roemer , ACS Chem. Biol. 2013, 8, 2442–2451. 23957438 10.1021/cb400487f

[207] B. Cavalleri , H. Pagani , G. Volpe , E. Selva , F. Parenti , J. Antibiot. 1984, 37, 309–317. 10.7164/antibiotics.37.309 6547132

[208] R. Pallanza , M. Berti , R. Scotti , E. Randisi , V. Arioli , J. Antibiot. 1984, 37, 318–324. 10.7164/antibiotics.37.318 6547133

[209] X. Fang , K. Tiyanont , Y. Zhang , J. Wanner , D. Boger , S. Walker , Mol. BioSyst. 2006, 2, 69–76. 16880924 10.1039/b515328j

[210] H. He , R. T. Williamson , B. Shen , E. I. Graziani , H. Y. Yang , S. M. Sakya , P. J. Petersen , G. T. Carter , J. Am. Chem. Soc. 2002, 124, 9729–9736. 12175230 10.1021/ja020257s

[211] A. Ruzin , G. Singh , A. Severin , Y. Yang , R. G. Dushin , A. G. Sutherland , A. Minnick , M. Greenstein , M. K. May , D. M. Shlaes , P. A. Bradford , Antimicrob. Agents Chemother. 2004, 48, 728–738. 14982757 10.1128/AAC.48.3.728-738.2004 PMC353120

[212] J. Shoji , H. Hinoo , T. Katayama , K. Matsumoto , T. Tanimoto , T. Hattori , I. Higashiyama , H. Miwa , K. Motokawa , T. Yoshida , J. Antibiot. 1992, 45, 817–823. 10.7164/antibiotics.45.817 1500345

[213] J. Shoji , H. Hinoo , T. Katayama , Y. Nakagawa , Y. Ikenishi , K. Iwatani , T. Yoshida , J. Antibiot. 1992, 45, 824–831. 10.7164/antibiotics.45.824 1500346

[214] H. Maki , K. Miura , Y. Yamano , Antimicrob. Agents Chemother. 2001, 45, 1823–1827. 11353632 10.1128/AAC.45.6.1823-1827.2001 PMC90552

[215] S. J. Kim , M. Singh , A. Wohlrab , T.-Y. Yu , G. J. Patti , R. D. O'Connor , M. VanNieuwenhze , J. Schaefer , Biochemistry 2013, 52, 1973–1979. 23421534 10.1021/bi4000222 PMC3628776

[216] R. D. O'Connor , M. Singh , J. Chang , S. J. Kim , M. VanNieuwenhze , J. Schaefer , J. Phys. Chem. B 2017, 121, 1499–1505. 28135800 10.1021/acs.jpcb.6b11039 PMC5555578

[217] J. O'Sullivan , J. E. McCullough , A. A. Tymiak , D. R. Kirsch , W. H. Trejo , P. A. Principe , J. Antibiot. 1988, 41, 1740–1744. 10.7164/antibiotics.41.1740 3209465

[218] J. Shoji , H. Hinoo , K. Matsumoto , T. Hattori , T. Yoshida , S. Matsuura , E. Kondo , J. Antibiot. 1988, 41, 713–718. 10.7164/antibiotics.41.713 3403364

[219] T. Kato , H. Hinoo , Y. Terui , J. Kikuchi , J. Shoji , J. Antibiot. 1988, 41, 719–725. 10.7164/antibiotics.41.719 3403365

[220] W. Lee , K. Schaefer , Y. Qiao , V. Srisuknimit , H. Steinmetz , R. Müller , D. Kahne , S. Walker , J. Am. Chem. Soc. 2016, 138, 100–103. 26683668 10.1021/jacs.5b11807 PMC4817722

[221] F. von Nussbaum , S. Anlauf , J. Benet-Buchholz , D. Häbich , J. Köbberling , L. Musza , J. Telser , H. Rübsamen-Waigmann , N. A. Brunner , Angew. Chem. Int. Ed. 2007, 46, 2039–2042; 10.1002/anie.200604232 17211904

[222] A. Guzman-Martinez , R. Lamer , M. S. VanNieuwenhze , J. Am. Chem. Soc. 2007, 129, 6017–6021. 17432854 10.1021/ja067648h PMC2151959

[223] C. Coronelli , G. Tamoni , G. C. Lancini , J. Antibiot. 1976, 29, 507–510. 10.7164/antibiotics.29.507 956038

[224] V. Arioli , M. Berti , L. G. Silvestri , J. Antibiot. 1976, 29, 511–515. 10.7164/antibiotics.29.511 8414

[225] N. Zimmermann , J. W. Metzger , G. Jung , Eur. J. Biochem. 1995, 228, 786–797. 7737178

[226] H. Brötz , G. Bierbaum , P. E. Reynolds , H. G. Sahl , Eur. J. Biochem. 1997, 246, 193–199. 9210483 10.1111/j.1432-1033.1997.t01-1-00193.x

[227] A. Malabarba , R. Pallanza , M. Berti , B. Cavalleri , J. Antibiot. 1990, 43, 1089–1097. 10.7164/antibiotics.43.1089 2211372

[228] S. Boakes , T. Ayala , M. Herman , A. N. Appleyard , M. J. Dawson , J. Cortés , Appl. Microbiol. Biotechnol. 2012, 95, 1509–1517. 22526797 10.1007/s00253-012-4041-0

[229] M. J. Dawson, A. N. Appleyard, J. C. Bargallo, S. N. Wadman, (Novacta Biosystems Limited), WO2011095769A1 **2010**.

[230] S. Chatterjee , D. K. Chatterjee , R. H. Jani , J. Blumbach , B. N. Ganguli , N. Klesel , M. Limbert , G. Seibert , J. Antibiot. 1992, 45, 839–845. 10.7164/antibiotics.45.839 1500348

[231] K. M. Gomes , R. S. Duarte , M. do C. de Freire Bastos , Microbiology 2017, 163, 109–121. 28270262 10.1099/mic.0.000397

[232] P. H. Mygind , R. L. Fischer , K. M. Schnorr , M. T. Hansen , C. P. Sönksen , S. Ludvigsen , D. Raventós , S. Buskov , B. Christensen , L. De Maria , O. Taboureau , D. Yaver , S. G. Elvig-Jørgensen , M. V. Sørensen , B. E. Christensen , S. Kjaerulff , N. Frimodt-Moller , R. I. Lehrer , M. Zasloff , H.-H. Kristensen , Nature 2005, 437, 975–980. 16222292 10.1038/nature04051

[233] T. Schneider , T. Kruse , R. Wimmer , I. Wiedemann , V. Sass , U. Pag , A. Jansen , A. K. Nielsen , P. H. Mygind , D. S. Raventós , S. Neve , B. Ravn , A. M. J. J. Bonvin , L. De Maria , A. S. Andersen , L. K. Gammelgaard , H.-G. Sahl , H.-H. Kristensen , Science 2010, 328, 1168–1172. 20508130 10.1126/science.1185723

[234] D. Andes , W. Craig , L. A. Nielsen , H. H. Kristensen , Antimicrob. Agents Chemother. 2009, 53, 3003–3009. 19414576 10.1128/AAC.01584-08 PMC2704636

[235] L. L. Ling , T. Schneider , A. J. Peoples , A. L. Spoering , I. Engels , B. P. Conlon , A. Mueller , T. F. Schäberle , D. E. Hughes , S. Epstein , M. Jones , L. Lazarides , V. A. Steadman , D. R. Cohen , C. R. Felix , K. A. Fetterman , W. P. Millett , A. G. Nitti , A. M. Zullo , C. Chen , K. Lewis , Nature 2015, 517, 455–459. 25561178 10.1038/nature14098 PMC7414797

[236] T. Homma , A. Nuxoll , A. B. Gandt , P. Ebner , I. Engels , T. Schneider , F. Götz , K. Lewis , B. P. Conlon , Antimicrob. Agents Chemother. 2016, 60, 6510–6517. 27550357 10.1128/AAC.01050-16 PMC5075054

[237] C. Öster , G. P. Walkowiak , D. E. Hughes , A. L. Spoering , A. J. Peoples , A. C. Catherwood , J. A. Tod , A. J. Lloyd , T. Herrmann , K. Lewis , C. G. Dowson , J. R. Lewandowski , Chem. Sci. 2018, 9, 8850–8859. 30627403 10.1039/c8sc03655a PMC6296168

[238] R. Shukla , J. Medeiros-Silva , A. Parmar , B. J. A. Vermeulen , S. Das , A. L. Paioni , S. Jekhmane , J. Lorent , A. M. J. J. Bonvin , M. Baldus , M. Lelli , E. J. A. Veldhuizen , E. Breukink , I. Singh , M. Weingarth , Nat. Commun. 2020, 11, 2848. 32503964 10.1038/s41467-020-16600-2 PMC7275090

[239] A. M. Giltrap , L. J. Dowman , G. Nagalingam , J. L. Ochoa , R. G. Linington , W. J. Britton , R. J. Payne , Org. Lett. 2016, 18, 2788–2791. 27191730 10.1021/acs.orglett.6b01324

[240] L. Liu , S. Wu , Q. Wang , M. Zhang , B. Wang , G. He , G. Chen , Org. Chem. Front. 2018, 5, 1431–1435.

[241] V. B. Gunjal , R. Thakare , S. Chopra , D. S. Reddy , J. Med. Chem. 2020, 63, 12171–12195. 32520557 10.1021/acs.jmedchem.0c00173

[242] E. J. Culp , N. Waglechner , W. Wang , A. A. Fiebig-Comyn , Y.-P. Hsu , K. Koteva , D. Sychantha , B. K. Coombes , M. S. Van Nieuwenhze , Y. V. Brun , G. D. Wright , Nature 2020, 578, 582–587. 32051588 10.1038/s41586-020-1990-9

[243] S. A. Blackman , T. J. Smith , S. J. Foster , Microbiology 1998, 144, 73–82. 9537764 10.1099/00221287-144-1-73

[244] V. Wiebach , A. Mainz , M.-A. J. Siegert , N. A. Jungmann , G. Lesquame , S. Tirat , A. Dreux-Zigha , J. Aszodi , D. Le Beller , R. D. Süssmuth , Nat. Chem. Biol. 2018, 14, 652–654. 29915235 10.1038/s41589-018-0068-6

[245] M. Kaul , Y. Zhang , A. K. Parhi , E. J. LaVoie , D. S. Pilch , Biochem. Pharmacol. 2014, 89, 321–328. 24637241 10.1016/j.bcp.2014.03.002

[246] S. E. Knudson , D. Awasthi , K. Kumar , A. Carreau , L. Goullieux , S. Lagrange , H. Vermet , I. Ojima , R. A. Slayden , J. Antimicrob. Chemother. 2015, 70, 3070–3073. 26245639 10.1093/jac/dkv226 PMC4613742

[247] J. M. Andreu , S. Huecas , L. Araújo-Bazán , H. Vázquez-Villa , M. Martín-Fontecha , Biomedicine 2022, 10, 1825. 10.3390/biomedicines10081825 PMC9405007 36009372

[248] J. Campbell , A. K. Singh , J. G. Swoboda , M. S. Gilmore , B. J. Wilkinson , S. Walker , Antimicrob. Agents Chemother. 2012, 56, 1810–1820. 22290958 10.1128/AAC.05938-11 PMC3318382

[249] S. Brown , J. P. Santa Maria , S. Walker , Annu. Rev. Microbiol. 2013, 67, 313–336. 24024634 10.1146/annurev-micro-092412-155620 PMC3883102

[250] C. Weidenmaier , A. Peschel , Nat. Rev. Microbiol. 2008, 6, 276–287. 18327271 10.1038/nrmicro1861

[251] M. G. Percy , A. Gründling , Annu. Rev. Microbiol. 2014, 68, 81–100. 24819367 10.1146/annurev-micro-091213-112949

[252] J. G. Swoboda , J. Campbell , T. C. Meredith , S. Walker , ChemBioChem 2010, 11, 35–45. 19899094 10.1002/cbic.200900557 PMC2798926

[253] M. A. D'Elia , J. A. Henderson , T. J. Beveridge , D. E. Heinrichs , E. D. Brown , J. Bacteriol. 2009, 191, 4030–4034. 19376878 10.1128/JB.00611-08 PMC2698391

[254] J. G. Swoboda , T. C. Meredith , J. Campbell , S. Brown , T. Suzuki , T. Bollenbach , A. J. Malhowski , R. Kishony , M. S. Gilmore , S. Walker , ACS Chem. Biol. 2009, 4, 875–883. 19689117 10.1021/cb900151k PMC2787957

[255] K. Lee , J. Campbell , J. G. Swoboda , G. D. Cuny , S. Walker , Bioorg. Med. Chem. Lett.. 2010, 20, 1767–1770. 20138521 10.1016/j.bmcl.2010.01.036 PMC2844852

[256] L. M. Matano , H. G. Morris , A. R. Hesser , S. E. S. Martin , W. Lee , T. W. Owens , E. Laney , H. Nakaminami , D. Hooper , T. C. Meredith , S. Walker , J. Am. Chem. Soc. 2017, 139, 10597–10600. 28727445 10.1021/jacs.7b04726 PMC5699463

[257] S. H. Lee , H. Wang , M. Labroli , S. Koseoglu , P. Zuck , T. Mayhood , C. Gill , P. Mann , X. Sher , S. Ha , S.-W. Yang , M. Mandal , C. Yang , L. Liang , Z. Tan , P. Tawa , Y. Hou , R. Kuvelkar , K. DeVito , X. Wen , J. Xiao , M. Batchlett , C. J. Balibar , J. Liu , J. Xiao , N. Murgolo , C. G. Garlisi , P. R. Sheth , A. Flattery , J. Su , C. Tan , T. Roemer , Sci. Transl. Med. 2016, 8, 329ra32–329ra32. 10.1126/scitranslmed.aad7364 26962156

[258] S. G. Richter , D. Elli , H. K. Kim , A. P. A. Hendrickx , J. A. Sorg , O. Schneewind , D. Missiakas , Proc. Natl. Acad. Sci. USA 2013, 110, 3531–3536. 23401520 10.1073/pnas.1217337110 PMC3587227

[259] X. Chee Wezen , A. Chandran , R. S. Eapen , E. Waters , L. Bricio-Moreno , T. Tosi , S. Dolan , C. Millership , A. Kadioglu , A. Gründling , L. S. Itzhaki , M. Welch , T. Rahman , J. Chem. Inf. Model. 2022, 62, 2586–2599. 35533315 10.1021/acs.jcim.2c00300 PMC9131456

[260] S. Tiberi , N. du Plessis , G. Walzl , M. J. Vjecha , M. Rao , F. Ntoumi , S. Mfinanga , N. Kapata , P. Mwaba , T. D. McHugh , G. Ippolito , G. B. Migliori , M. J. Maeurer , A. Zumla , Lancet Infect. Dis. 2018, 18, e183–e198. 10.1016/S1473-3099(18)30110-5 29580819

[261] Y. S. Polikanov , N. A. Aleksashin , B. Beckert , D. N. Wilson , Front. Mol. Biosci. 2018, 5, 48. 29868608 10.3389/fmolb.2018.00048 PMC5960728

[262] J. Lin , D. Zhou , T. A. Steitz , Y. S. Polikanov , M. G. Gagnon , Annu. Rev. Biochem. 2018, 87, 451–478. 29570352 10.1146/annurev-biochem-062917-011942 PMC9176271

[263] S. Arenz , D. N. Wilson , Mol. Cell 2016, 61, 3–14. 26585390 10.1016/j.molcel.2015.10.019

[264] S. E. Dmitriev , D. O. Vladimirov , K. A. Lashkevich , Biochemistry 2020, 85, 1389–1421. 33280581 10.1134/S0006297920110097 PMC7689648

[265] T. Otaka , A. Kaji , FEBS Lett. 1981, 123, 173–176. 7014241 10.1016/0014-5793(81)80280-3

[266] T. Otaka , A. Kaji , FEBS Lett. 1983, 153, 53–59. 6337880 10.1016/0014-5793(83)80118-5

[267] J. M. Waisvisz , M. G. van der Hoeven , J. van Peppen , W. C. M. Zwennis , J. Am. Chem. Soc. 1957, 79, 4520–4521.

[268] S. Nakamura , T. Yajima , Y. Lin , H. Umezawa , J. Antibiot. 1967, 20, 1–5. 6072278

[269] S. Nakamura , T. Chikaike , K. Karasawa , N. Tanaka , H. Yonehara , H. Umezawa , J. Antibiot. Ser. A 1965, 18, 47–52. 14282373

[270] U. Kazmaier , Isr. J. Chem. 2021, 61, 308–321.

[271] T. Yamada , M. Yagita , Y. Kobayashi , G. Sennari , H. Shimamura , H. Matsui , Y. Horimatsu , H. Hanaki , T. Hirose , S. O̅mura , T. Sunazuka , J. Org. Chem. 2018, 83, 7135–7149. 29560726 10.1021/acs.joc.8b00045

[272] H.-G. Lerchen, G. Schiffer, H. Brötz-Oesterhelt, A. Mayer-Bartschmid, S. Eckermann, C. Freiberg, R. Endermann, J. Schuhmacher, H. Meier, N. Svenstrup, S. Seip, M. Gehling, D. Häbich, (Aicuris GmbH & Co. KG), WO2006103010A1, **2006**.

[273] D. C. McKinney , G. S. Basarab , A. I. Cocozaki , M. A. Foulk , M. D. Miller , A. M. Ruvinsky , C. W. Scott , K. Thakur , L. Zhao , E. T. Buurman , S. Narayan , ACS Med. Chem. Lett. 2015, 6, 930–935. 26288696 10.1021/acsmedchemlett.5b00205 PMC4538447

[274] B. Raju , S. Anandan , S. Gu , P. Herradura , H. O'Dowd , B. Kim , M. Gomez , C. Hackbarth , C. Wu , W. Wang , Z. Yuan , R. White , J. Trias , D. V. Patel , Bioorg. Med. Chem. Lett.. 2004, 14, 3103–3107. 15149653 10.1016/j.bmcl.2004.04.036

[275] B. Raju , K. Mortell , S. Anandan , H. O'Dowd , H. Gao , M. Gomez , C. Hackbarth , C. Wu , W. Wang , Z. Yuan , R. White , J. Trias , D. V. Patel , Bioorg. Med. Chem. Lett.. 2003, 13, 2413–2418. 12824046 10.1016/s0960-894x(03)00393-7

[276] L. Pantel , T. Florin , M. Dobosz-Bartoszek , E. Racine , M. Sarciaux , M. Serri , J. Houard , J.-M. Campagne , R. M. de Figueiredo , C. Midrier , S. Gaudriault , A. Givaudan , A. Lanois , S. Forst , A. Aumelas , C. Cotteaux-Lautard , J.-M. Bolla , C. Vingsbo Lundberg , D. L. Huseby , D. Hughes , P. Villain-Guillot , A. S. Mankin , Y. S. Polikanov , M. Gualtieri , Mol. Cell 2018, 70, 83–94.e7. 29625040 10.1016/j.molcel.2018.03.001

[277] T. Bulatov , S. Gensel , A. Mainz , T. Dang , T. O. Koller , K. Voigt , J. Ebeling , D. N. Wilson , E. Genersch , R. D. Süssmuth , J. Am. Chem. Soc. 2022, 144, 288–296. 34968060 10.1021/jacs.1c09616

[278] T. O. Koller , M. J. Berger , M. Morici , H. Paternoga , T. Bulatov , A. D. Stasi , T. Dang , A. Mainz , K. Raulf , C. Crowe-McAuliffe , M. Scocchi , M. Mardirossian , B. Beckert , N. Vázquez-Laslop , A. Mankin , R. D. Süssmuth , D. N. Wilson , bioRxiv preprint 2024, DOI: 10.1101/2024.05.21.595107. PMC11581978 39420228

[279] T. O. Koller , U. Scheid , T. Kösel , J. Herrmann , D. Krug , H. I. M. Boshoff , B. Beckert , J. C. Evans , J. Schlemmer , B. Sloan , D. M. Weiner , L. E. Via , A. Moosa , T. R. Ioerger , M. Graf , B. Zinshteyn , M. Abdelshahid , F. Nguyen , S. Arenz , F. Gille , M. Siebke , T. Seedorf , O. Plettenburg , R. Green , A.-L. Warnke , J. Ullrich , R. Warrass , C. E. 3rd Barry , D. F. Warner , V. Mizrahi , A. Kirschning , D. N. Wilson , R. Müller , J. Am. Chem. Soc. 2023, 145, 851–863. 36603206 10.1021/jacs.2c08816 PMC9853869

[280] R. Sutherland , R. J. Boon , K. E. Griffin , P. J. Masters , B. Slocombe , A. R. White , Antimicrob. Agents Chemother. 1985, 27, 495–498. 3923922 10.1128/aac.27.4.495 PMC180082

[281] I. A. Critchley , C. L. Young , K. C. Stone , U. A. Ochsner , J. Guiles , T. Tarasow , N. Janjic , Antimicrob. Agents Chemother. 2005, 49, 4247–4252. 16189105 10.1128/AAC.49.10.4247-4252.2005 PMC1251549

[282] U. A. Ochsner , C. L. Young , K. C. Stone , F. B. Dean , N. Janjic , I. A. Critchley , Antimicrob. Agents Chemother. 2005, 49, 4253–4262. 16189106 10.1128/AAC.49.10.4253-4262.2005 PMC1251548

[283] B. K. Lomeli , H. Galbraith , J. Schettler , G. A. Saviolakis , W. El-Amin , B. Osborn , J. Ravel , K. Hazleton , C. A. Lozupone , R. J. Evans , S. J. Bell , U. A. Ochsner , T. C. Jarvis , S. Baqar , N. Janjic , Antimicrob. Agents Chemother. 2019, 64, e01395–19. 31685472 10.1128/AAC.01395-19 PMC7187627

[284] S. U. Nayak , J. M. Griffiss , J. Blumer , M. A. O'Riordan , W. Gray , R. McKenzie , R. A. Jurao , A. T. An , M. Le , S. J. Bell , U. A. Ochsner , T. C. Jarvis , N. Janjic , J. M. Zenilman , Antimicrob. Agents Chemother. 2017, 61, e02760–16. 28584140 10.1128/AAC.02760-16 PMC5527627

[285] M. J. LaMarche , J. A. Leeds , A. Amaral , J. T. Brewer , S. M. Bushell , G. Deng , J. M. Dewhurst , J. Ding , J. Dzink-Fox , G. Gamber , A. Jain , K. Lee , L. Lee , T. Lister , D. McKenney , S. Mullin , C. Osborne , D. Palestrant , M. A. Patane , E. M. Rann , M. Sachdeva , J. Shao , S. Tiamfook , A. Trzasko , L. Whitehead , A. Yifru , D. Yu , W. Yan , Q. Zhu , J. Med. Chem. 2012, 55, 2376–2387. 22315981 10.1021/jm201685h

[286] S. G. Bhansali , K. Mullane , L. S. L. Ting , J. A. Leeds , K. Dabovic , J. Praestgaard , P. Pertel , Antimicrob. Agents Chemother. 2015, 59, 1441–1445. 25534724 10.1128/AAC.04252-14 PMC4325791

[287] A. Parmeggiani , P. Nissen , FEBS Lett. 2006, 580, 4576–4581. 16876786 10.1016/j.febslet.2006.07.039

[288] J. J. Gordon , B. K. Kelly , G. A. Miller , Nature 1962, 195, 701–702. 10.1038/195701b0 13900478

[289] J. M. Clements , R. P. Beckett , A. Brown , G. Catlin , M. Lobell , S. Palan , W. Thomas , M. Whittaker , S. Wood , S. Salama , P. J. Baker , H. F. Rodgers , V. Barynin , D. W. Rice , M. G. Hunter , Antimicrob. Agents Chemother. 2001, 45, 563–570. 11158755 10.1128/AAC.45.2.563-570.2001 PMC90327

[290] D. Lofland , S. Difuntorum , A. Waller , J. M. Clements , M. K. Weaver , J. A. Karlowsky , K. Johnson , J. Antimicrob. Chemother. 2004, 53, 664–668. 14973152 10.1093/jac/dkh129

[291] C. S. Osborne , G. Neckermann , E. Fischer , R. Pecanka , D. Yu , K. Manni , J. Goldovitz , K. Amaral , J. Dzink-Fox , N. S. Ryder , Antimicrob. Agents Chemother. 2009, 53, 3777–3781. 19596876 10.1128/AAC.00026-09 PMC2737836

[292] L. Pang , S. D. Weeks , A. Van Aerschot , Int. J. Mol. Sci. 2021, 22, 1750. 33578647 10.3390/ijms22041750 PMC7916415

[293] N. H. Kwon , P. L. Fox , S. Kim , Nat. Rev. Drug Discovery 2019, 18, 629–650. 31073243 10.1038/s41573-019-0026-3

[294] D. R. P. Guay , Ther. Clin. Risk Manage. 2007, 3, 513–525. PMC2374925 18472972

[295] Z.-Q. Xu , N. E. Dixon , Curr. Opin. Struct. Biol. 2018, 53, 159–168. 30292863 10.1016/j.sbi.2018.09.006

[296] C. S. McHenry , Curr. Opin. Chem. Biol. 2011, 15, 587–594. 21855395 10.1016/j.cbpa.2011.07.018 PMC3190588

[297] K. Timinskas , M. Balvočiūtė , A. Timinskas , Č. Venclovas , Nucleic Acids Res. 2014, 42, 1393–1413. 24106089 10.1093/nar/gkt900 PMC3919608

[298] A. Robinson , R. J. Causer , N. E. Dixon , Curr. Drug Targets 2012, 13, 352–372. 22206257 10.2174/138945012799424598 PMC3290774

[299] W. D. Celmer , G. N. Chmurny , C. E. Moppett , R. S. Ware , P. C. Watts , E. B. Whipple , J. Am. Chem. Soc. 1980, 102, 4203–4209.

[300] S. J. Pidot , M. A. Rizzacasa , Chem. Eur. J. 2020, 26, 2780–2792. 31667915 10.1002/chem.201904053

[301] R. E. Painter , G. C. Adam , M. Arocho , E. DiNunzio , R. G. K. Donald , K. Dorso , O. Genilloud , C. Gill , M. Goetz , N. N. Hairston , N. Murgolo , B. Nare , D. B. Olsen , M. Powles , F. Racine , J. Su , F. Vicente , D. Wisniewski , L. Xiao , M. Hammond , K. Young , Chem. Biol. 2015, 22, 1362–1373. 26456734 10.1016/j.chembiol.2015.08.015

[302] M. D. Chengalroyen , M. K. Mason , A. Borsellini , R. Tassoni , G. L. Abrahams , S. Lynch , Y.-M. Ahn , J. Ambler , K. Young , B. M. Crowley , D. B. Olsen , D. F. Warner , C. E. Barry III , H. I. M. Boshoff , M. H. Lamers , V. Mizrahi , ACS Infect. Dis. 2022, 8, 612–625. 35143160 10.1021/acsinfecdis.1c00643 PMC8922275

[303] K. Young, D. B. Olsen, S. B. Singh, J. Su, R. R. Wilkening, J. M. Apgar, D. Meng, D. Parker, M. Mandal, L. Yang, R. E. Painter, Q. Dang, T. Suzuki (Merck Sharp & Dohme LLC), WO2016061772A1, **2016**.

[304] P. J. Dudfield, J. Lowther, C. A. J. Delachaume, R. H. M. Lépine, A. P. M. Thys, J. G. P.-O. Doyon, M. P. Toumi, F. G. Hansske, (Galapagos Nv), WO2015028094A1, **2015**.

[305] N. C. Brown , R. E. Handschumacher , J. Biol. Chem. 1966, 241, 3083–3089. 4957971

[306] C. Zhi , Z.-Y. Long , J. Gambino , W.-C. Xu , N. C. Brown , M. Barnes , M. Butler , W. LaMarr , G. E. Wright , J. Med. Chem. 2003, 46, 2731–2739. 12801236 10.1021/jm020591z

[307] Y. Rose , S. Ciblat , R. Reddy , A. C. Belley , E. Dietrich , D. Lehoux , G. A. McKay , H. Poirier , A. R. Far , D. Delorme , Bioorg. Med. Chem. Lett.. 2006, 16, 891–896. 16298129 10.1016/j.bmcl.2005.11.009

[308] N. Svenstrup , K. Ehlert , C. Ladel , A. Kuhl , D. Häbich , ChemMedChem 2008, 3, 1604–1615. 18792034 10.1002/cmdc.200800117

[309] W.-C. Xu , G. E. Wright , N. C. Brown , Z.-Y. Long , C.-X. Zhi , S. Dvoskin , J. J. Gambino , M. H. Barnes , M. M. Butler , Bioorg. Med. Chem. Lett. 2011, 21, 4197–4202. 21684746 10.1016/j.bmcl.2011.05.093 PMC3128661

[310] G. E. Wright , N. C. Brown , W.-C. Xu , Z. Long , C. Zhi , J. J. Gambino , M. H. Barnes , M. M. Butler , Bioorg. Med. Chem. Lett.. 2005, 15, 729–732. 15664846 10.1016/j.bmcl.2004.11.016

[311] A. Torti , A. Lossani , L. Savi , F. Focher , G. E. Wright , N. C. Brown , W.-C. Xu , Curr. Enzyme Inhib. 2011, 7, 147–153. 10.2174/157340811798807597 PMC3404731 22844265

[312] B. Murray , C. Wolfe , A. Marra , C. Pillar , D. Shinabarger , J. Antimicrob. Chemother. 2020, 75, 2149–2155. 32285102 10.1093/jac/dkaa134

[313] S. Dvoskin , W.-C. Xu , N. C. Brown , I. B. Yanachkov , M. Yanachkova , G. E. Wright , Antimicrob. Agents Chemother. 2012, 56, 1624–1626. 22203600 10.1128/AAC.06097-11 PMC3294905

[314] H. Noufflard-Guy-Loé , S. Berteaux , Rev. Tuberc. Pneumol. 1965, 29, 301–326. 4953631

[315] A. Kling , P. Lukat , D. V. Almeida , A. Bauer , E. Fontaine , S. Sordello , N. Zaburannyi , J. Herrmann , S. C. Wenzel , C. König , N. C. Ammerman , M. B. Barrio , K. Borchers , F. Bordon-Pallier , M. Brönstrup , G. Courtemanche , M. Gerlitz , M. Geslin , P. Hammann , D. W. Heinz , H. Hoffmann , S. Klieber , M. Kohlmann , M. Kurz , C. Lair , H. Matter , E. Nuermberger , S. Tyagi , L. Fraisse , J. H. Grosset , S. Lagrange , R. Müller , Science 2015, 348, 1106–1112. 26045430 10.1126/science.aaa4690

[316] H. Brötz-Oesterhelt , I. Knezevic , S. Bartel , T. Lampe , U. Warnecke-Eberz , K. Ziegelbauer , D. Häbich , H. Labischinski , J. Biol. Chem. 2003, 278, 39435–39442. 12867414 10.1074/jbc.M306479200

[317] S. D. Mills , A. E. Eakin , E. T. Buurman , J. V. Newman , N. Gao , H. Huynh , K. D. Johnson , S. Lahiri , A. B. Shapiro , G. K. Walkup , W. Yang , S. S. Stokes , Antimicrob. Agents Chemother. 2011, 55, 1088–1096. 21189350 10.1128/AAC.01181-10 PMC3067096

[318] D. Aiello , M. H. Barnes , E. E. Biswas , S. B. Biswas , S. Gu , J. D. Williams , T. L. Bowlin , D. T. Moir , Bioorg. Med. Chem. 2009, 17, 4466–4476. 19477652 10.1016/j.bmc.2009.05.014 PMC2776654

[319] B. Li , R. Pai , M. Di , D. Aiello , M. H. Barnes , M. M. Butler , T. F. Tashjian , N. P. Peet , T. L. Bowlin , D. T. Moir , J. Med. Chem. 2012, 55, 10896–10908. 23231076 10.1021/jm300922h PMC3531573

[320] G. A. McKay , R. Reddy , F. Arhin , A. Belley , D. Lehoux , G. Moeck , I. Sarmiento , T. R. Parr , P. Gros , J. Pelletier , A. R. Far , Bioorg. Med. Chem. Lett. 2006, 16, 1286–1290. 16343901 10.1016/j.bmcl.2005.11.076

[321] S. J. McKie , K. C. Neuman , A. Maxwell , BioEssays 2021, 43, 2000286. 10.1002/bies.202000286 PMC7614492 33480441

[322] T. Kamiyama , N. Shimma , T. Ohtsuka , N. Nakayama , Y. Itezono , N. Nakada , J. Watanabe , K. Yokose , J. Antibiot. 1994, 47, 37–45. 10.7164/antibiotics.47.37 8119860

[323] E. Goetschi , P. Angehrn , H. Gmuender , P. Hebeisen , H. Link , R. Masciadri , J. Nielsen , Pharmacol. Ther. 1993, 60, 367–380. 8022866 10.1016/0163-7258(93)90017-8

[324] N. Nakada , H. Shimada , T. Hirata , Y. Aoki , T. Kamiyama , J. Watanabe , M. Arisawa , Antimicrob. Agents Chemother. 1993, 37, 2656–2661. 8109932 10.1128/aac.37.12.2656 PMC192769

[325] R. J. Lewis , O. M. Singh , C. V. Smith , A. Maxwell , T. Skarzynski , A. J. Wonacott , D. B. Wigley , J. Mol. Biol. 1994, 241, 128–130. 8051702 10.1006/jmbi.1994.1480

[326] P. Angehrn , E. Goetschi , H. Gmuender , P. Hebeisen , M. Hennig , B. Kuhn , T. Luebbers , P. Reindl , F. Ricklin , A. Schmitt-Hoffmann , J. Med. Chem. 2011, 54, 2207–2224. 21388139 10.1021/jm1014023

[327] S. Tohyama , Y. Takahashi , Y. Akamatsu , J. Antibiot. 2010, 63, 147–149. 10.1038/ja.2010.1 20111064

[328] J. W. Phillips , M. A. Goetz , S. K. Smith , D. L. Zink , J. Polishook , R. Onishi , S. Salowe , J. Wiltsie , J. Allocco , J. Sigmund , K. Dorso , S. Lee , S. Skwish , M. de la Cruz , J. Martín , F. Vicente , O. Genilloud , J. Lu , R. E. Painter , K. Young , K. Overbye , R. G. K. Donald , S. B. Singh , Chem. Biol. 2011, 18, 955–965. 21867911 10.1016/j.chembiol.2011.06.011

[329] J. Lu , S. Patel , N. Sharma , S. M. Soisson , R. Kishii , M. Takei , Y. Fukuda , K. J. Lumb , S. B. Singh , ACS Chem. Biol. 2014, 9, 2023–2031. 24992706 10.1021/cb5001197

[330] S. Yang , C. Chen , J. Chen , C. Li , J. Am. Chem. Soc. 2021, 143, 21258–21263. 34879199 10.1021/jacs.1c11477

[331] S. B. Singh , P. Dayananth , C. J. Balibar , C. G. Garlisi , J. Lu , R. Kishii , M. Takei , Y. Fukuda , S. Ha , K. Young , Antimicrob. Agents Chemother. 2015, 59, 3474–3481. 25845866 10.1128/AAC.00382-15 PMC4432116

[332] L. W. Tari , X. Li , M. Trzoss , D. C. Bensen , Z. Chen , T. Lam , J. Zhang , S. J. Lee , G. Hough , D. Phillipson , S. Akers-Rodriguez , M. L. Cunningham , B. P. Kwan , K. J. Nelson , A. Castellano , J. B. Locke , V. Brown-Driver , T. M. Murphy , V. S. Ong , C. M. Pillar , D. L. Shinabarger , J. Nix , F. C. Lightstone , S. E. Wong , T. B. Nguyen , K. J. Shaw , J. Finn , PLoS One 2013, 8, e84409. 24386374 10.1371/journal.pone.0084409 PMC3873466

[333] S. A. Leyn , J. E. Kent , J. E. Zlamal , M. L. Elane , M. Vercruysse , A. L. Osterman , bioRxiv preprint 2023, DOI: 10.1101/2023.06.26.546596.

[334] S. Park , R. Russo , L. Westfall , R. Shrestha , M. Zimmerman , V. Dartois , N. Kurepina , B. Kreiswirth , E. Singleton , S.-G. Li , N. Mittal , Y.-M. Ahn , J. Bilotta , K. L. Connolly , A. E. Jerse , J. S. Freundlich , D. S. Perlin , Antimicrob. Agents Chemother. 2022, 66, e0041422. 35972242 10.1128/aac.00414-22 PMC9487510

[335] W. J. Coates, M. N. Gwynn, I. K. Hatton, P. J. Masters, N. D. Pearson, S. S. Rahman, B. Slocombe, J. D. Warrack (Smithkline Beecham Plc), WO1999037635A1, **1999**.

[336] B. D. Bax , P. F. Chan , D. S. Eggleston , A. Fosberry , D. R. Gentry , F. Gorrec , I. Giordano , M. M. Hann , A. Hennessy , M. Hibbs , J. Huang , E. Jones , J. Jones , K. K. Brown , C. J. Lewis , E. W. May , M. R. Saunders , O. Singh , C. E. Spitzfaden , C. Shen , A. Shillings , A. J. Theobald , A. Wohlkonig , N. D. Pearson , M. N. Gwynn , Nature 2010, 466, 935–940. 20686482 10.1038/nature09197

[337] M. T. Black , T. Stachyra , D. Platel , A.-M. Girard , M. Claudon , J.-M. Bruneau , C. Miossec , Antimicrob. Agents Chemother. 2008, 52, 3339–3349. 18625781 10.1128/AAC.00496-08 PMC2533460

[338] M. Kokot , M. Anderluh , M. Hrast , N. Minovski , J. Med. Chem. 2022, 65, 6431–6440. 35503563 10.1021/acs.jmedchem.2c00039 PMC9109137

[339] Bugworks Research Inc. A First in Human Study of the Safety and Tolerability of Single and Multiple Doses of BWC0977 in Healthy Volunteers, ClinicalTrials.gov identifier: NCT05088421. Updated July 6 **2023**. Accessed November 28, 2024. https://clinicaltrials.gov/study/NCT05088421.

[340] S. H. P. Mohamed, N. Bharatham, N. Katagihallimath, S. Sharma, R. Nandishaiah, V. Ramachandran, B. Venkataraman, (Bugworks Research India Pvt Ltd), WO2018225097A1, **2018**.

[341] G. S. Basarab , P. Brassil , P. Doig , V. Galullo , H. B. Haimes , G. Kern , A. Kutschke , J. McNulty , V. J. A. Schuck , G. Stone , M. Gowravaram , J. Med. Chem. 2014, 57, 9078–9095. 25286019 10.1021/jm501174m

[342] A. A. Miller , G. L. Bundy , J. E. Mott , J. E. Skepner , T. P. Boyle , D. W. Harris , A. E. Hromockyj , K. R. Marotti , G. E. Zurenko , J. B. Munzner , M. T. Sweeney , G. F. Bammert , J. C. Hamel , C. W. Ford , W.-Z. Zhong , D. R. Graber , G. E. Martin , F. Han , L. A. Dolak , E. P. Seest , J. C. Ruble , G. M. Kamilar , J. R. Palmer , L. S. Banitt , A. R. Hurd , M. R. Barbachyn , Antimicrob. Agents Chemother. 2008, 52, 2806–2812. 18519725 10.1128/AAC.00247-08 PMC2493097

[343] P. A. Bradford , A. A. Miller , J. O'Donnell , J. P. Mueller , ACS Infect. Dis. 2020, 6, 1332–1345. 32329999 10.1021/acsinfecdis.0c00021

[344] S. M. Hashimi , J. Antibiot. 2019, 72, 785–792. 10.1038/s41429-019-0228-2 31451755

[345] S. Cociancich , A. Pesic , D. Petras , S. Uhlmann , J. Kretz , V. Schubert , L. Vieweg , S. Duplan , M. Marguerettaz , J. Noëll , I. Pieretti , M. Hügelland , S. Kemper , A. Mainz , P. Rott , M. Royer , R. D. Süssmuth , Nat. Chem. Biol. 2015, 11, 195–197. 25599532 10.1038/nchembio.1734

[346] L. von Eckardstein , D. Petras , T. Dang , S. Cociancich , S. Sabri , S. Grätz , D. Kerwat , M. Seidel , A. Pesic , P. C. Dorrestein , M. Royer , J. B. Weston , R. D. Süssmuth , Chemistry 2017, 23, 15316–15321. 28876492 10.1002/chem.201704074

[347] S. Grätz , D. Kerwat , J. Kretz , L. von Eckardstein , S. Semsary , M. Seidel , M. Kunert , J. B. Weston , R. D. Süssmuth , ChemMedChem 2016, 11, 1499–1502. 27245621 10.1002/cmdc.201600163

[348] I. Behroz , P. Durkin , S. Grätz , M. Seidel , L. Rostock , M. Spinczyk , J. B. Weston , R. D. Süssmuth , Chem. Eur. J. 2019, 25, 16538–16543. 31642561 10.1002/chem.201904752 PMC6972991

[349] D. Kerwat , S. Grätz , J. Kretz , M. Seidel , M. Kunert , J. B. Weston , R. D. Süssmuth , ChemMedChem 2016, 11, 1899–1903. 27439374 10.1002/cmdc.201600231

[350] L. Kleebauer , L. Zborovsky , K. Hommernick , M. Seidel , J. B. Weston , R. D. Süssmuth , Org. Lett. 2021, 23, 7023–7027. 34398605 10.1021/acs.orglett.1c02312

[351] S. Baumann , J. Herrmann , R. Raju , H. Steinmetz , K. I. Mohr , S. Hüttel , K. Harmrolfs , M. Stadler , R. Müller , Angew. Chem. Int. Ed. 2014, 53, 14605–14609; 10.1002/anie.201409964 25510965

[352] G. Testolin , K. Cirnski , K. Rox , H. Prochnow , V. Fetz , C. Grandclaudon , T. Mollner , A. Baiyoumy , A. Ritter , C. Leitner , J. Krull , J. van den Heuvel , A. Vassort , S. Sordello , M. M. Hamed , W. A. M. Elgaher , J. Herrmann , R. W. Hartmann , R. Müller , M. Brönstrup , Chem. Sci. 2020, 11, 1316–1334. 10.1039/c9sc04769g PMC8148378 34123255

[353] E. Michalczyk , K. Hommernick , I. Behroz , M. Kulike , Z. Pakosz-Stępień , L. Mazurek , M. Seidel , M. Kunert , K. Santos , H. von Moeller , B. Loll , J. B. Weston , A. Mainz , J. G. Heddle , R. D. Süssmuth , D. Ghilarov , Nat. Catal. 2023, 6, 52–67. 36741192 10.1038/s41929-022-00904-1 PMC9886550

[354] Y. Imai , G. Hauk , J. Quigley , L. Liang , S. Son , M. Ghiglieri , M. F. Gates , M. Morrissette , N. Shahsavari , S. Niles , D. Baldisseri , C. Honrao , X. Ma , J. J. Guo , J. M. Berger , K. Lewis , Nat. Chem. Biol. 2022, 18, 1236–1244. 35996001 10.1038/s41589-022-01102-7 PMC9844538

[355] A. T. Bakker , I. Kotsogianni , M. Avalos , J. M. Punt , B. Liu , D. Piermarini , B. Gagestein , C. J. Slingerland , L. Zhang , J. J. Willemse , L. B. Ghimire , R. J. H. B. N. van den Berg , A. P. A. Janssen , T. H. M. Ottenhoff , C. A. A. van Boeckel , G. P. van Wezel , D. Ghilarov , N. I. Martin , M. van der Stelt , Nat. Chem. 2024, 1–11. 38898213 10.1038/s41557-024-01516-x PMC11374673

[356] E. I. Parkinson , J. S. Bair , B. A. Nakamura , H. Y. Lee , H. I. Kuttab , E. H. Southgate , S. Lezmi , G. W. Lau , P. J. Hergenrother , Nat. Commun. 2015, 6, 6947. 25907309 10.1038/ncomms7947 PMC4421842

[357] K. Hiramatsu , M. Igarashi , Y. Morimoto , T. Baba , M. Umekita , Y. Akamatsu , Int. J. Antimicrob. Agents 2012, 39, 478–485. 22534508 10.1016/j.ijantimicag.2012.02.007

[358] A. Srivastava , M. Talaue , S. Liu , D. Degen , R. Y. Ebright , E. Sineva , A. Chakraborty , S. Y. Druzhinin , S. Chatterjee , J. Mukhopadhyay , Y. W. Ebright , A. Zozula , J. Shen , S. Sengupta , R. R. Niedfeldt , C. Xin , T. Kaneko , H. Irschik , R. Jansen , S. Donadio , N. Connell , R. H. Ebright , Curr. Opin. Microbiol. 2011, 14, 532–543. 21862392 10.1016/j.mib.2011.07.030 PMC3196380

[359] S. Tuske , S. G. Sarafianos , X. Wang , B. Hudson , E. Sineva , J. Mukhopadhyay , J. J. Birktoft , O. Leroy , S. Ismail , A. D. Clark , C. Dharia , A. Napoli , O. Laptenko , J. Lee , S. Borukhov , R. H. Ebright , E. Arnold , Cell 2005, 122, 541–552. 16122422 10.1016/j.cell.2005.07.017 PMC2754413

[360] G. F. Crum , W. H. Devries , T. E. Eble , C. M. Large , J. W. Shell , Antibiot. Annu. 1955, 3, 893–896. 13355382

[361] C. Deboer , A. Dietz , G. M. Savage , W. S. Silver , Antibiot. Annu. 1955, 3, 886–892. 13355381

[362] R. Jansen , V. Wray , H. Irschik , H. Reichenbach , G. Höfle , Tetrahedron Lett. 1985, 26, 6031–6034.

[363] H. Irschik , R. Jansen , K. Gerth , G. Höfle , H. Reichenbach , J. Antibiot. 1987, 40, 7–13. 10.7164/antibiotics.40.7 3104268

[364] H. Irschik , K. Gerth , G. Höfle , W. Kohl , H. Reichenbach , J. Antibiot. 1983, 36, 1651–1658. 10.7164/antibiotics.36.1651 6420386

[365] J. Mukhopadhyay , K. Das , S. Ismail , D. Koppstein , M. Jang , B. Hudson , S. Sarafianos , S. Tuske , J. Patel , R. Jansen , H. Irschik , E. Arnold , R. H. Ebright , Cell 2008, 135, 295–307. 18957204 10.1016/j.cell.2008.09.033 PMC2580802

[366] H. Irschik , H. Augustiniak , K. Gerth , G. Höfle , H. Reichenbach , J. Antibiot. 1995, 48, 787–792. 10.7164/antibiotics.48.787 7592022

[367] A. K. Krome , T. Becker , S. Kehraus , A. Schiefer , M. Gütschow , L. Chaverra-Muñoz , S. Hüttel , R. Jansen , M. Stadler , A. Ehrens , D. Pogorevc , R. Müller , M. P. Hübner , T. Hesterkamp , K. Pfarr , A. Hoerauf , K. G. Wagner , G. M. König , Nat. Prod. Rep. 2022, 39, 1705–1720. 35730490 10.1039/d2np00012a

[368] A. Schiefer , M. P. Hübner , A. Krome , C. Lämmer , A. Ehrens , T. Aden , M. Koschel , H. Neufeld , L. Chaverra-Muñoz , R. Jansen , S. Kehraus , G. M. König , D. Pogorevc , R. Müller , M. Stadler , S. Hüttel , T. Hesterkamp , K. Wagner , K. Pfarr , A. Hoerauf , PLoS Neglected Trop. Dis. 2020, 14, e0008930. 10.1371/journal.pntd.0008930 PMC7746275 33284808

[369] E. Sarubbi , F. Monti , E. Corti , A. Miele , E. Selva , Eur. J. Biochem. 2004, 271, 3146–3154. 15265034 10.1111/j.1432-1033.2004.04244.x

[370] I. Ciciliato , E. Corti , E. Sarubbi , S. Stefanelli , L. Gastaldo , N. Montanini , M. Kurz , D. Losi , F. Marinelli , E. Selva , J. Antibiot. 2004, 57, 210–217. 10.7164/antibiotics.57.210 15152807

[371] D. Degen , Y. Feng , Y. Zhang , K. Y. Ebright , Y. W. Ebright , M. Gigliotti , H. Vahedian-Movahed , S. Mandal , M. Talaue , N. Connell , E. Arnold , W. Fenical , R. H. Ebright , eLife 2014, 3, e02451. 24843001 10.7554/eLife.02451 PMC4029172

[372] I. Artsimovitch , C. Chu , A. S. Lynch , R. Landick , Science 2003, 302, 650–654. 14576436 10.1126/science.1087526

[373] A. M. Malinen , M. NandyMazumdar , M. Turtola , H. Malmi , T. Grocholski , I. Artsimovitch , G. A. Belogurov , Nat. Commun. 2014, 5, 3408. 24598909 10.1038/ncomms4408 PMC3959191

[374] W. Zhu , J. Haupenthal , M. Groh , M. Fountain , R. W. Hartmann , Antimicrob. Agents Chemother. 2014, 58, 4242–4245. 24820077 10.1128/AAC.02600-14 PMC4068539

[375] S. I. Maffioli , Y. Zhang , D. Degen , T. Carzaniga , G. D. Gatto , S. Serina , P. Monciardini , C. Mazzetti , P. Guglierame , G. Candiani , A. I. Chiriac , G. Facchetti , P. Kaltofen , H.-G. Sahl , G. Dehò , S. Donadio , R. H. Ebright , Cell 2017, 169, 1240–1248.e23. 28622509 10.1016/j.cell.2017.05.042 PMC5542026

[376] D. A. Collins , T. V. Riley , Lett. Appl. Microbiol. 2022, 75, 526–536. 35119124 10.1111/lam.13664 PMC9541751

[377] W. Hasler, Y.-H. Ji, W. Leupin, (Hoffmann La Roche Inc.), US5824698A, **1998**.

[378] J. Mann , P. W. Taylor , C. R. Dorgan , P. D. Johnson , F. X. Wilson , R. Vickers , A. G. Dale , S. Neidle , MedChemComm 2015, 6, 1420–1426. 26949507 10.1039/c5md00238a PMC4756575

[379] C. S. Mason , T. Avis , C. Hu , N. Nagalingam , M. Mudaliar , C. Coward , K. Begum , K. Gajewski , M. J. Alam , E. Bassères , S. Moss , S. Reich , E. Duperchy , K. R. Fox , K. W. Garey , D. J. Powell , Antimicrob. Agents Chemother. 2023, 67, e0156322. 37093023 10.1128/aac.01563-22 PMC10246881

[380] H. Kohn , W. Widger , Curr. Drug Targets Infect. Disord. 2005, 5, 273–295. 16181146 10.2174/1568005054880136

[381] J. Yao , C. O. Rock , Biochim. Biophys. Acta Mol. Cell Biol. Lipids 2017, 1862, 1300–1309. 27668701 10.1016/j.bbalip.2016.09.014 PMC5364071

[382] E. J. North , M. Jackson , R. E. Lee , Curr. Pharm. Des. 2014, 20, 4357–4378. 24245756 10.2174/1381612819666131118203641 PMC4568743

[383] J. A. Karlowsky , N. Kaplan , B. Hafkin , D. J. Hoban , G. G. Zhanel , Antimicrob. Agents Chemother. 2009, 53, 3544–3548. 19487444 10.1128/AAC.00400-09 PMC2715641

[384] J. A. Karlowsky , N. M. Laing , T. Baudry , N. Kaplan , D. Vaughan , D. J. Hoban , G. G. Zhanel , Antimicrob. Agents Chemother. 2007, 51, 1580–1581. 17220418 10.1128/AAC.01254-06 PMC1855524

[385] N. Kaplan , M. Albert , D. Awrey , E. Bardouniotis , J. Berman , T. Clarke , M. Dorsey , B. Hafkin , J. Ramnauth , V. Romanov , M. B. Schmid , R. Thalakada , J. Yethon , H. W. Pauls , Antimicrob. Agents Chemother. 2012, 56, 5865–5874. 22948878 10.1128/AAC.01411-12 PMC3486558

[386] D. J. Payne , W. H. Miller , V. Berry , J. Brosky , W. J. Burgess , E. Chen , W. E. DeWolf , A. P. Fosberry , R. Greenwood , M. S. Head , D. A. Heerding , C. A. Janson , D. D. Jaworski , P. M. Keller , P. J. Manley , T. D. Moore , K. A. Newlander , S. Pearson , B. J. Polizzi , X. Qiu , S. F. Rittenhouse , C. Slater-Radosti , K. L. Salyers , M. A. Seefeld , M. G. Smyth , D. T. Takata , I. N. Uzinskas , K. Vaidya , N. G. Wallis , S. B. Winram , C. C. K. Yuan , W. F. Huffman , Antimicrob. Agents Chemother. 2002, 46, 3118–3124. 12234833 10.1128/AAC.46.10.3118-3124.2002 PMC128775

[387] W. H. Miller , M. A. Seefeld , K. A. Newlander , I. N. Uzinskas , W. J. Burgess , D. A. Heerding , C. C. K. Yuan , M. S. Head , D. J. Payne , S. F. Rittenhouse , T. D. Moore , S. C. Pearson , V. Berry , W. E. DeWolf , P. M. Keller , B. J. Polizzi , X. Qiu , C. A. Janson , W. F. Huffman , J. Med. Chem. 2002, 45, 3246–3256. 12109908 10.1021/jm020050+

[388] R. Heath , S. White , C. Rock , Appl. Microbiol. Biotechnol. 2002, 58, 695–703. 12021787 10.1007/s00253-001-0918-z

[389] J. B. Parsons , M. W. Frank , C. Subramanian , P. Saenkham , C. O. Rock , Proc. Natl. Acad. Sci. USA 2011, 108, 15378–15383. 21876172 10.1073/pnas.1109208108 PMC3174620

[390] F. Wittke , C. Vincent , J. Chen , B. Heller , H. Kabler , J. S. Overcash , F. Leylavergne , G. Dieppois , Antimicrob. Agents Chemother. 2020, 64, e00250–20. 32747361 10.1128/AAC.00250-20 PMC7508579

[391] A. Menetrey , A. Janin , J. Pullman , J. S. Overcash , A. Haouala , F. Leylavergne , L. Turbe , F. Wittke , V. Nicolas-Métral , Antimicrob. Agents Chemother. 2019, 63, 1–11. 10.1128/AAC.01669-18 PMC6395911 30559136

[392] S. Escaich , L. Prouvensier , M. Saccomani , L. Durant , M. Oxoby , V. Gerusz , F. Moreau , V. Vongsouthi , K. Maher , I. Morrissey , C. Soulama-Mouze , Antimicrob. Agents Chemother. 2011, 55, 4692–4697. 21825292 10.1128/AAC.01248-10 PMC3186954

[393] H. S. Park , Y. M. Yoon , S. J. Jung , C. M. Kim , J. M. Kim , J.-H. Kwak , J. Antimicrob. Chemother. 2007, 60, 568–574. 17606482 10.1093/jac/dkm236

[394] J. Wang , S. M. Soisson , K. Young , W. Shoop , S. Kodali , A. Galgoci , R. Painter , G. Parthasarathy , Y. S. Tang , R. Cummings , S. Ha , K. Dorso , M. Motyl , H. Jayasuriya , J. Ondeyka , K. Herath , C. Zhang , L. Hernandez , J. Allocco , Á. Basilio , J. R. Tormo , O. Genilloud , F. Vicente , F. Pelaez , L. Colwell , S. H. Lee , B. Michael , T. Felcetto , C. Gill , L. L. Silver , J. D. Hermes , K. Bartizal , J. Barrett , D. Schmatz , J. W. Becker , D. Cully , S. B. Singh , Nature 2006, 441, 358–361. 16710421 10.1038/nature04784

[395] S. B. Singh , H. Jayasuriya , J. G. Ondeyka , K. B. Herath , C. Zhang , D. L. Zink , N. N. Tsou , R. G. Ball , A. Basilio , O. Genilloud , M. T. Diez , F. Vicente , F. Pelaez , K. Young , J. Wang , J. Am. Chem. Soc. 2006, 128, 11916–11920. 16953632 10.1021/ja062232p

[396] K. C. Nicolaou , A. Li , D. J. Edmonds , G. S. Tria , S. P. Ellery , J. Am. Chem. Soc. 2009, 131, 16905–16918. 19874023 10.1021/ja9068003 PMC2783699

[397] K. Tiefenbacher , J. Mulzer , J. Org. Chem. 2009, 74, 2937–2941. 19260660 10.1021/jo9001855

[398] H. C. Shen , F.-X. Ding , S. B. Singh , G. Parthasarathy , S. M. Soisson , S. N. Ha , X. Chen , S. Kodali , J. Wang , K. Dorso , J. R. Tata , M. L. Hammond , M. Maccoss , S. L. Colletti , Bioorg. Med. Chem. Lett. 2009, 19, 1623–1627. 19233644 10.1016/j.bmcl.2009.02.006

[399] S. B. Singh , K. B. Herath , J. Wang , N. Tsou , R. G. Ball , Tetrahedron Lett. 2007, 48, 5429–5433.

[400] C. Zhang , J. Ondeyka , K. Herath , H. Jayasuriya , Z. Guan , D. L. Zink , L. Dietrich , B. Burgess , S. N. Ha , J. Wang , S. B. Singh , J. Nat. Prod. 2011, 74, 329–340. 21214253 10.1021/np100635f

[401] H. Jayasuriya , K. B. Herath , J. G. Ondeyka , D. L. Zink , B. Burgess , J. Wang , S. B. Singh , Tetrahedron Lett. 2008, 49, 3648–3651.

[402] J. Wang , S. Kodali , S. H. Lee , A. Galgoci , R. Painter , K. Dorso , F. Racine , M. Motyl , L. Hernandez , E. Tinney , S. L. Colletti , K. Herath , R. Cummings , O. Salazar , I. González , A. Basilio , F. Vicente , O. Genilloud , F. Pelaez , H. Jayasuriya , K. Young , D. F. Cully , S. B. Singh , Proc. Natl. Acad. Sci. USA 2007, 104, 7612–7616. 17456595 10.1073/pnas.0700746104 PMC1863502

[403] C.-Y. Lai , J. E. Cronan , J. Biol. Chem. 2003, 278, 51494–51503. 14523010 10.1074/jbc.M308638200

[404] C. Freiberg , N. A. Brunner , G. Schiffer , T. Lampe , J. Pohlmann , M. Brands , M. Raabe , D. Häbich , K. Ziegelbauer , J. Biol. Chem. 2004, 279, 26066–26073. 15066985 10.1074/jbc.M402989200

[405] M. Kienle , P. Eisenring , B. Stoessel , O. P. Horlacher , S. Hasler , G. van Colen , R. C. Hartkoorn , A. Vocat , S. T. Cole , K.-H. Altmann , J. Med. Chem. 2020, 63, 1105–1131. 31904960 10.1021/acs.jmedchem.9b01457

[406] R. C. Hartkoorn , C. Sala , J. Neres , F. Pojer , S. Magnet , R. Mukherjee , S. Uplekar , S. Boy-Röttger , K.-H. Altmann , S. T. Cole , EMBO Mol. Med. 2012, 4, 1032–1042. 22987724 10.1002/emmm.201201689 PMC3491834

[407] K. A. Sacksteder , M. Protopopova , C. E. Barry , K. Andries , C. A. Nacy , Future Microbiol. 2012, 7, 823–837. 22827305 10.2217/fmb.12.56 PMC3480206

[408] M. Protopopova , C. Hanrahan , B. Nikonenko , R. Samala , P. Chen , J. Gearhart , L. Einck , C. A. Nacy , J. Antimicrob. Chemother. 2005, 56, 968–974. 16172107 10.1093/jac/dki319

[409] K. Li , L. A. Schurig-Briccio , X. Feng , A. Upadhyay , V. Pujari , B. Lechartier , F. L. Fontes , H. Yang , G. Rao , W. Zhu , A. Gulati , J. H. No , G. Cintra , S. Bogue , Y.-L. Liu , K. Molohon , P. Orlean , D. A. Mitchell , L. Freitas-Junior , F. Ren , H. Sun , T. Jiang , Y. Li , R.-T. Guo , S. T. Cole , R. B. Gennis , D. C. Crick , E. Oldfield , J. Med. Chem. 2014, 57, 3126–3139. 24568559 10.1021/jm500131s PMC4084622

[410] R. T. Sauer , T. A. Baker , Annu. Rev. Biochem. 2011, 80, 587–612. 21469952 10.1146/annurev-biochem-060408-172623

[411] C. Moreno-Cinos , K. Goossens , I. G. Salado , P. Van Der Veken , H. De Winter , K. Augustyns , Int. J. Mol. Sci. 2019, 20, 2232. 31067645 10.3390/ijms20092232 PMC6540193

[412] K. Ito , Y. Akiyama , Annu. Rev. Microbiol. 2005, 59, 211–231. 15910274 10.1146/annurev.micro.59.030804.121316

[413] M. Bogyo , J. S. McMaster , M. Gaczynska , D. Tortorella , A. L. Goldberg , H. Ploegh , Proc. Natl. Acad. Sci. USA 1997, 94, 6629–6634. 9192616 10.1073/pnas.94.13.6629 PMC21209

[414] V. Tsilibaris , G. Maenhaut-Michel , L. Van Melderen , Res. Microbiol. 2006, 157, 701–713. 16854568 10.1016/j.resmic.2006.05.004

[415] J. Ollinger , T. O'Malley , E. A. Kesicki , J. Odingo , T. Parish , J. Bacteriol. 2012, 194, 663–668. 22123255 10.1128/JB.06142-11 PMC3264079

[416] K. H. Michel, R. E. Kastner, (Eli Lilly & Co), US4492650 A, **1985**.

[417] H. Osada , T. Yano , H. Koshino , K. Isono , J. Antibiot. 1991, 44, 1463–1466. 10.7164/antibiotics.44.1463 1778798

[418] J. Kirstein , A. Hoffmann , H. Lilie , R. Schmidt , H. Rübsamen-Waigmann , H. Brötz-Oesterhelt , A. Mogk , K. Turgay , EMBO Mol. Med. 2009, 1, 37–49. 20049702 10.1002/emmm.200900002 PMC3378108

[419] B.-G. Lee , E. Y. Park , K.-E. Lee , H. Jeon , K. H. Sung , H. Paulsen , H. Rübsamen-Schaeff , H. Brötz-Oesterhelt , H. K. Song , Nat. Struct. Mol. Biol. 2010, 17, 471–478. 20305655 10.1038/nsmb.1787

[420] H. Brötz-Oesterhelt , D. Beyer , H.-P. Kroll , R. Endermann , C. Ladel , W. Schroeder , B. Hinzen , S. Raddatz , H. Paulsen , K. Henninger , J. E. Bandow , H.-G. Sahl , H. Labischinski , Nat. Med. 2005, 11, 1082–1087. 16200071 10.1038/nm1306

[421] P. Sass , M. Josten , K. Famulla , G. Schiffer , H.-G. Sahl , L. Hamoen , H. Brötz-Oesterhelt , Proc. Natl. Acad. Sci. USA 2011, 108, 17474–17479. 21969594 10.1073/pnas.1110385108 PMC3198362

[422] N. Silber , S. Pan , S. Schäkermann , C. Mayer , H. Brötz-Oesterhelt , P. Sass , mBio 2020, 11, e01006–20. 32605984 10.1128/mBio.01006-20 PMC7327170

[423] U. Schmidt , K. Neumann , A. Schumacher , S. Weinbrenner , Angew. Chem. Int. Ed. 1997, 36, 1110–1112;

[424] B. Hinzen , S. Raddatz , H. Paulsen , T. Lampe , A. Schumacher , D. Häbich , V. Hellwig , J. Benet-Buchholz , R. Endermann , H. Labischinski , H. Brötz-Oesterhelt , ChemMedChem 2006, 1, 689–693. 16902918 10.1002/cmdc.200600055

[425] E. C. Griffith , Y. Zhao , A. P. Singh , B. P. Conlon , R. Tangallapally , W. R. Shadrick , J. Liu , M. J. Wallace , L. Yang , J. M. Elmore , Y. Li , Z. Zheng , D. J. Miller , M. N. Cheramie , R. B. Lee , M. D. LaFleur , K. Lewis , R. E. Lee , ACS Infect. Dis. 2019, 5, 1915–1925. 31588734 10.1021/acsinfecdis.9b00245 PMC6916429

[426] N. Arisetti , H. L. S. Fuchs , J. Coetzee , M. Orozco , D. Ruppelt , A. Bauer , D. Heimann , E. Kuhnert , S. P. Bhamidimarri , J. A. Bafna , B. Hinkelmann , K. Eckel , S. A. Sieber , P. P. Müller , J. Herrmann , R. Müller , M. Winterhalter , C. Steinem , M. Brönstrup , Chem. Sci. 2021, 12, 16023–16034. 35024125 10.1039/d1sc04290d PMC8672772

[427] P. Labana , M. H. Dornan , M. Lafrenière , T. L. Czarny , E. D. Brown , J. P. Pezacki , C. N. Boddy , Cell Chem. Biol. 2021, 28, 1703–1715.e11. 34293284 10.1016/j.chembiol.2021.07.001

[428] M. G. Darnowski , T. D. Lanosky , P. Labana , J. T. Brazeau-Henrie , N. D. Calvert , M. H. Dornan , C. Natola , A. R. Paquette , A. J. Shuhendler , C. N. Boddy , RSC Med. Chem. 2022, 13, 436–444. 35647545 10.1039/d1md00355k PMC9020616

[429] T. Böttcher , S. A. Sieber , Angew. Chem. Int. Ed. 2008, 47, 4600–4603; 10.1002/anie.200705768 18383487

[430] T. Böttcher , S. A. Sieber , J. Am. Chem. Soc. 2008, 130, 14400–14401. 18847196 10.1021/ja8051365

[431] M. K. Renner , Y.-C. Shen , X.-C. Cheng , P. R. Jensen , W. Frankmoelle , C. A. Kauffman , W. Fenical , E. Lobkovsky , J. Clardy , J. Am. Chem. Soc. 1999, 121, 11273–11276.

[432] E. K. Schmitt , M. Riwanto , V. Sambandamurthy , S. Roggo , C. Miault , C. Zwingelstein , P. Krastel , C. Noble , D. Beer , S. P. S. Rao , M. Au , P. Niyomrattanakit , V. Lim , J. Zheng , D. Jeffery , K. Pethe , L. R. Camacho , Angew. Chem. Int. Ed. 2011, 50, 5889–5891; 10.1002/anie.201101740 21563281

[433] M. P. Choules , N. M. Wolf , H. Lee , J. R. Anderson , E. M. Grzelak , Y. Wang , R. Ma , W. Gao , J. B. McAlpine , Y.-Y. Jin , J. Cheng , H. Lee , J.-W. Suh , N. M. Duc , S. Paik , J. H. Choe , E.-K. Jo , C. L. Chang , J. S. Lee , B. U. Jaki , G. F. Pauli , S. G. Franzblau , S. Cho , Antimicrob. Agents Chemother. 2019, 63, 1–17. 10.1128/AAC.02204-18 PMC6395927 30602512

[434] W. Gao , J.-Y. Kim , J. R. Anderson , T. Akopian , S. Hong , Y.-Y. Jin , O. Kandror , J.-W. Kim , I.-A. Lee , S.-Y. Lee , J. B. McAlpine , S. Mulugeta , S. Sunoqrot , Y. Wang , S.-H. Yang , T.-M. Yoon , A. L. Goldberg , G. F. Pauli , J.-W. Suh , S. G. Franzblau , S. Cho , Antimicrob. Agents Chemother. 2015, 59, 880–889. 25421483 10.1128/AAC.04054-14 PMC4335914

[435] S. Lear , T. Munshi , A. S. Hudson , C. Hatton , J. Clardy , J. A. Mosely , T. J. Bull , C. S. Sit , S. L. Cobb , Org. Biomol. Chem. 2016, 14, 4534–4541. 27101411 10.1039/c6ob00631k

[436] E. Gavrish , C. S. Sit , S. Cao , O. Kandror , A. Spoering , A. Peoples , L. Ling , A. Fetterman , D. Hughes , A. Bissell , H. Torrey , T. Akopian , A. Mueller , S. Epstein , A. Goldberg , J. Clardy , K. Lewis , Chem. Biol. 2014, 21, 509–518. 24684906 10.1016/j.chembiol.2014.01.014 PMC4060151

[437] D. Vasudevan , S. P. S. Rao , C. G. Noble , J. Biol. Chem. 2013, 288, 30883–30891. 24022489 10.1074/jbc.M113.493767 PMC3829403

[438] N. M. Wolf , H. Lee , M. P. Choules , G. F. Pauli , R. Phansalkar , J. R. Anderson , W. Gao , J. Ren , B. D. Santarsiero , H. Lee , J. Cheng , Y.-Y. Jin , N. A. Ho , N. M. Duc , J.-W. Suh , C. Abad-Zapatero , S. Cho , ACS Infect. Dis. 2019, 5, 829–840. 30990022 10.1021/acsinfecdis.8b00276 PMC6657506

[439] M. Paetzel , in Bacterial Cell Walls and Membranes (Ed.: A. Kuhn ), Springer International Publishing, Cham, 2019, pp. 187–219.

[440] M. Paetzel , R. E. Dalbey , N. C. Strynadka , Pharmacol. Ther. 2000, 87, 27–49. 10924740 10.1016/s0163-7258(00)00064-4

[441] A. Höltzel , D. G. Schmid , G. J. Nicholson , S. Stevanovic , J. Schimana , K. Gebhardt , H.-P. Fiedler , G. Jung , J. Antibiot. 2002, 55, 571–577. 10.7164/antibiotics.55.571 12195963

[442] J. Schimana , K. Gebhardt , A. Höltzel , D. G. Schmid , R. Süssmuth , J. Müller , R. Pukall , H.-P. Fiedler , J. Antibiot. 2002, 55, 565–570. 10.7164/antibiotics.55.565 12195962

[443] M. Paetzel , J. J. Goodall , M. Kania , R. E. Dalbey , M. G. P. Page , J. Biol. Chem. 2004, 279, 30781–30790. 15136583 10.1074/jbc.M401686200

[444] J. Dufour , L. Neuville , J. Zhu , Chem. Eur. J. 2010, 16, 10523–10534. 20658499 10.1002/chem.201000924

[445] J. Liu , C. Luo , P. A. Smith , J. K. Chin , M. G. P. Page , M. Paetzel , F. E. Romesberg , J. Am. Chem. Soc. 2011, 133, 17869–17877. 21999324 10.1021/ja207318n PMC3277211

[446] T. C. Roberts , M. A. Schallenberger , J. Liu , P. A. Smith , F. E. Romesberg , J. Med. Chem. 2011, 54, 4954–4963. 21630667 10.1021/jm1016126 PMC3151006

[447] J. Liu , P. A. Smith , D. Barrios Steed , F. Romesberg , Bioorg. Med. Chem. Lett. 2013, 23, 5654–5659. 24012184 10.1016/j.bmcl.2013.08.026 PMC3981466

[448] P. A. Smith , M. E. Powers , T. C. Roberts , F. E. Romesberg , Antimicrob. Agents Chemother. 2011, 55, 1130–1134. 21189343 10.1128/AAC.01459-10 PMC3067118

[449] P. A. Smith , T. C. Roberts , F. E. Romesberg , Chem. Biol. 2010, 17, 1223–1231. 21095572 10.1016/j.chembiol.2010.09.009 PMC3003444

[450] R. E. W. Hancock , A. Bell , Eur. J. Clin. Microbiol. Infect. Dis. 1988, 7, 713–720. 2850910 10.1007/BF01975036

[451] L. Vogeley , T. El Arnaout , J. Bailey , P. J. Stansfeld , C. Boland , M. Caffrey , Science 2016, 351, 876–880. 26912896 10.1126/science.aad3747

[452] S. Olatunji , X. Yu , J. Bailey , C.-Y. Huang , M. Zapotoczna , K. Bowen , M. Remškar , R. Müller , E. M. Scanlan , J. A. Geoghegan , V. Olieric , M. Caffrey , Nat. Commun. 2020, 11, 140. 31919415 10.1038/s41467-019-13724-y PMC6952399

[453] E. Leung , A. Datti , M. Cossette , J. Goodreid , S. E. McCaw , M. Mah , A. Nakhamchik , K. Ogata , M. El Bakkouri , Y.-Q. Cheng , S. J. Wodak , B. T. Eger , E. F. Pai , J. Liu , S. Gray-Owen , R. A. Batey , W. A. Houry , Chem. Biol. 2011, 18, 1167–1178. 21944755 10.1016/j.chembiol.2011.07.023

[454] H. Strahl , J. Errington , Annu. Rev. Microbiol. 2017, 71, 519–538. 28697671 10.1146/annurev-micro-102215-095630

[455] J. R. Willdigg , J. D. Helmann , Front. Mol. Biosci. 2021, 8 : 634438. 10.3389/fmolb.2021.634438 PMC8144471 34046426

[456] J. Sun , S. T. Rutherford , T. J. Silhavy , K. C. Huang , Nat. Rev. Microbiol. 2022, 20, 236–248. 34732874 10.1038/s41579-021-00638-0 PMC8934262

[457] V. R. I. Kaila , M. Wikström , Nat. Rev. Microbiol. 2021, 19, 319–330. 33437024 10.1038/s41579-020-00486-4

[458] M. J. Trimble , P. Mlynárčik , M. Kolář , R. E. W. Hancock , Cold Spring Harbor Perspect. Med. 2016, 6, a025288. 10.1101/cshperspect.a025288 PMC5046685 27503996

[459] M. A. T. Blaskovich , M. E. Pitt , A. G. Elliott , M. A. Cooper , Expert Rev. Anti-Infect. Ther. 2018, 16, 485–499. 29848132 10.1080/14787210.2018.1483240 PMC6335237

[460] C.-D. Qian , X.-C. Wu , Y. Teng , W.-P. Zhao , O. Li , S.-G. Fang , Z.-H. Huang , H.-C. Gao , Antimicrob. Agents Chemother. 2012, 56, 1458–1465. 22183171 10.1128/AAC.05580-11 PMC3294921

[461] B. Becker , M. S. Butler , K. A. Hansford , A. Gallardo-Godoy , A. G. Elliott , J. X. Huang , D. J. Edwards , M. A. T. Blaskovich , M. A. Cooper , Bioorg. Med. Chem. Lett. 2017, 27, 2407–2409. 28454673 10.1016/j.bmcl.2017.04.027 PMC5515593

[462] T. Velkov , A. Gallardo-Godoy , J. D. Swarbrick , Mark A. T. Blaskovich , A. G. Elliott , M. Han , P. E. Thompson , K. D. Roberts , J. X. Huang , B. Becker , M. S. Butler , L. H. Lash , S. T. Henriques , R. L. Nation , S. Sivanesan , M.-A. Sani , F. Separovic , H. Mertens , D. Bulach , T. Seemann , J. Owen , J. Li , M. A. Cooper , Cell Chem. Biol. 2018, 25, 380–391.e5. 29396290 10.1016/j.chembiol.2018.01.005 PMC6560181

[463] Y.-X. Li , Z. Zhong , W.-P. Zhang , P.-Y. Qian , Nat. Commun. 2018, 9, 3273. 30115920 10.1038/s41467-018-05781-6 PMC6095874

[464] K. A. Ayed , R. D. Ballantine , M. Hoekstra , S. J. Bann , C. M. J. Wesseling , A. T. Bakker , Z. Zhong , Y.-X. Li , N. C. Brüchle , M. van der Stelt , S. A. Cochrane , N. I. Martin , Chem. Sci. 2022, 13, 3563–3570. 35432860 10.1039/d2sc00143h PMC8943889

[465] S. Choi , A. Isaacs , D. Clements , D. Liu , H. Kim , R. W. Scott , J. D. Winkler , W. F. DeGrado , Proc. Natl. Acad. Sci. USA 2009, 106, 6968–6973. 19359494 10.1073/pnas.0811818106 PMC2667368

[466] B. Mensa , Y. H. Kim , S. Choi , R. Scott , G. A. Caputo , W. F. DeGrado , Antimicrob. Agents Chemother. 2011, 55, 5043–5053. 21844313 10.1128/AAC.05009-11 PMC3195038

[467] R. W. Scott , G. N. Tew , Curr. Top. Med. Chem. 2017, 17, 576–589. 27411325 10.2174/1568026616666160713130452

[468] J. A. Silverman , N. G. Perlmutter , H. M. Shapiro , Antimicrob. Agents Chemother. 2003, 47, 2538–2544. 12878516 10.1128/AAC.47.8.2538-2544.2003 PMC166110

[469] T. Zhang , J. K. Muraih , B. MacCormick , J. Silverman , M. Palmer , Biochim. Biophys. Acta 2014, 1838, 2425–2430. 24857935 10.1016/j.bbamem.2014.05.014

[470] F. Grein , A. Müller , K. M. Scherer , X. Liu , K. C. Ludwig , A. Klöckner , M. Strach , H.-G. Sahl , U. Kubitscheck , T. Schneider , Nat. Commun. 2020, 11, 1455. 32193379 10.1038/s41467-020-15257-1 PMC7081307

[471] A. Müller , M. Wenzel , H. Strahl , F. Grein , T. N. V. Saaki , B. Kohl , T. Siersma , J. E. Bandow , H.-G. Sahl , T. Schneider , L. W. Hamoen , Proc. Natl. Acad. Sci. USA 2016, 113, E7077–E7086. 27791134 10.1073/pnas.1611173113 PMC5111643

[472] J. A. Karas , G. P. Carter , B. P. Howden , A. M. Turner , O. K. A. Paulin , J. D. Swarbrick , Mark A. Baker , J. Li , T. Velkov , J. Med. Chem. 2020, 63, 13266–13290. 32687352 10.1021/acs.jmedchem.0c00780

[473] L. Vértesy , E. Ehlers , H. Kogler , M. Kurz , J. Meiwes , G. Seibert , M. Vogel , P. Hammann , J. Antibiot. 2000, 53, 816–827. 10.7164/antibiotics.53.816 11079804

[474] T. Schneider , K. Gries , M. Josten , I. Wiedemann , S. Pelzer , H. Labischinski , H.-G. Sahl , Antimicrob. Agents Chemother. 2009, 53, 1610–1618. 19164139 10.1128/AAC.01040-08 PMC2663061

[475] T. Wecke , D. Zühlke , U. Mäder , S. Jordan , B. Voigt , S. Pelzer , H. Labischinski , G. Homuth , M. Hecker , T. Mascher , Antimicrob. Agents Chemother. 2009, 53, 1619–1623. 19164157 10.1128/AAC.01046-08 PMC2663105

[476] A. Koul , N. Dendouga , K. Vergauwen , B. Molenberghs , L. Vranckx , R. Willebrords , Z. Ristic , H. Lill , I. Dorange , J. Guillemont , D. Bald , K. Andries , Nat. Chem. Biol. 2007, 3, 323–324. 17496888 10.1038/nchembio884

[477] S. Kang , R. Y. Kim , M. J. Seo , S. Lee , Y. M. Kim , M. Seo , J. J. Seo , Y. Ko , I. Choi , J. Jang , J. Nam , S. Park , H. Kang , H. J. Kim , J. Kim , S. Ahn , K. Pethe , K. Nam , Z. No , J. Kim , J. Med. Chem. 2014, 57, 5293–5305. 24870926 10.1021/jm5003606

[478] C. S. Foo , A. Lupien , M. Kienle , A. Vocat , A. Benjak , R. Sommer , D. A. Lamprecht , A. J. C. Steyn , K. Pethe , J. Piton , K.-H. Altmann , S. T. Cole , mBio 2018, 9, 10.1128/mbio.01276-18. PMC6178619 30301850

[479] P. S. Shirude , B. Paul , N. Roy Choudhury , C. Kedari , B. Bandodkar , B. G. Ugarkar , ACS Med. Chem. Lett. 2012, 3, 736–740. 24900541 10.1021/ml300134b PMC4025736

[480] A. Heikal , K. Hards , C.-Y. Cheung , A. Menorca , M. S. M. Timmer , B. L. Stocker , G. M. Cook , J. Antimicrob. Chemother. 2016, 71, 2840–2847. 27365187 10.1093/jac/dkw244

[481] T. Beites , K. O'Brien , D. Tiwari , C. A. Engelhart , S. Walters , J. Andrews , H.-J. Yang , M. L. Sutphen , D. M. Weiner , E. K. Dayao , M. Zimmerman , B. Prideaux , P. V. Desai , T. Masquelin , L. E. Via , V. Dartois , H. I. Boshoff , C. E. Barry , S. Ehrt , D. Schnappinger , Nat. Commun. 2019, 10, 4970. 31672993 10.1038/s41467-019-12956-2 PMC6823465

[482] K. Hards , D. G. G. McMillan , L. A. Schurig-Briccio , R. B. Gennis , H. Lill , D. Bald , G. M. Cook , Proc. Natl. Acad. Sci. USA 2018, 115, 7326–7331. 29941569 10.1073/pnas.1803723115 PMC6048524

[483] N. A. Schilling , A. Berscheid , J. Schumacher , J. S. Saur , M. C. Konnerth , S. N. Wirtz , J. M. Beltrán-Beleña , A. Zipperer , B. Krismer , A. Peschel , H. Kalbacher , H. Brötz-Oesterhelt , C. Steinem , S. Grond , Angew. Chem. Int. Ed. 2019, 58, 9234–9238; 10.1002/anie.201901589 PMC6618241 31059155

[484] D. Ruppelt , M. F. W. Trollmann , T. Dema , S. N. Wirtz , H. Flegel , S. Mönnikes , S. Grond , R. A. Böckmann , C. Steinem , Nat. Commun. 2024, 15, 3521. 38664456 10.1038/s41467-024-47803-6 PMC11045845

[485] H. Terada , Environ. Health Perspect. 1990, 87, 213–218. 2176586 10.1289/ehp.9087213 PMC1567840

[486] H. Hamamoto , M. Urai , K. Ishii , J. Yasukawa , A. Paudel , M. Murai , T. Kaji , T. Kuranaga , K. Hamase , T. Katsu , J. Su , T. Adachi , R. Uchida , H. Tomoda , M. Yamada , M. Souma , H. Kurihara , M. Inoue , K. Sekimizu , Nat. Chem. Biol. 2015, 11, 127–133. 25485686 10.1038/nchembio.1710

[487] H. Itoh , K. Tokumoto , T. Kaji , A. Paudel , S. Panthee , H. Hamamoto , K. Sekimizu , M. Inoue , J. Org. Chem. 2018, 83, 6924–6935. 29019678 10.1021/acs.joc.7b02318

[488] M. Sang , H. Wang , Y. Shen , N. Rodrigues de Almeida , M. Conda-Sheridan , S. Li , Y. Li , L. Du , Org. Lett. 2019, 21, 6432–6436. 31386380 10.1021/acs.orglett.9b02333

[489] L. Li , B. Koirala , Y. Hernandez , L. W. MacIntyre , M. A. Ternei , R. Russo , S. F. Brady , Nat. Microbiol. 2022, 7, 120–131. 34949828 10.1038/s41564-021-01013-8 PMC8732328

[490] Y. Huan , Q. Kong , H. Mou , H. Yi , Front. Microbiol. 2020, 11, 582779. 33178164 10.3389/fmicb.2020.582779 PMC7596191

[491] S. P. S. Rao , S. Alonso , L. Rand , T. Dick , K. Pethe , Proc. Natl. Acad. Sci. USA 2008, 105, 11945–11950. 18697942 10.1073/pnas.0711697105 PMC2575262

[492] I. R. Poxton , in Molecular Medical Microbiology (Second Edition) (Eds.: Y.-W. Tang , M. Sussman , D. Liu , I. Poxton , J. Schwartzman ), Academic Press, Boston, 2015, pp. 91–103.

[493] B. Bertani , N. Ruiz , EcoSal Plus 2018, 8, 1–33. 10.1128/ecosalplus.esp-0001-2018 PMC6091223 30066669

[494] P. Zhou , J. Zhao , Biochim. Biophys. Acta 2017, 1862, 1424–1438. 10.1016/j.bbalip.2016.11.014 PMC5466501 27940308

[495] J. H. Moffatt , M. Harper , P. Harrison , J. D. F. Hale , E. Vinogradov , T. Seemann , R. Henry , B. Crane , F. St. Michael , A. D. Cox , B. Adler , R. L. Nation , J. Li , J. D. Boyce , Antimicrob. Agents Chemother. 2010, 54, 4971–4977. 20855724 10.1128/AAC.00834-10 PMC2981238

[496] T. Ogura , K. Inoue , T. Tatsuta , T. Suzaki , K. Karata , K. Young , L. H. Su , C. A. Fierke , J. E. Jackman , C. R. Raetz , J. Coleman , T. Tomoyasu , H. Matsuzawa , Mol. Microbiol. 1999, 31, 833–844. 10048027 10.1046/j.1365-2958.1999.01221.x

[497] H. R. Onishi , B. A. Pelak , L. S. Gerckens , L. L. Silver , F. M. Kahan , M. H. Chen , A. A. Patchett , S. M. Galloway , S. A. Hyland , M. S. Anderson , C. R. Raetz , Science 1996, 274, 980–982. 8875939 10.1126/science.274.5289.980

[498] G. Chapoux, J.-C. Gauvin, C. Hubschwerlen, A. Mirre, E. Ochala, J.-L. Specklin, J.-P. Surivet (Actelion Pharmaceuticals Ltd), US9624206B2, **2017**.

[499] S. M. Cohen, D. T. Puerta, C. Perez (The Regents of the University of California, USA), WO2015085238, **2015**.

[500] A. L. Erwin , Cold Spring Harbor Perspect. Med. 2016, 6, a025304. 10.1101/cshperspect.a025304 PMC4930914 27235477

[501] F. Cohen , J. B. Aggen , L. D. Andrews , Z. Assar , J. Boggs , T. Choi , P. Dozzo , A. N. Easterday , C. M. Haglund , D. J. Hildebrandt , M. C. Holt , K. Joly , A. Jubb , Z. Kamal , T. R. Kane , A. W. Konradi , K. M. Krause , M. S. Linsell , T. D. Machajewski , O. Miroshnikova , H. E. Moser , V. Nieto , T. Phan , C. Plato , A. W. Serio , J. Seroogy , A. Shakhmin , A. J. Stein , A. D. Sun , S. Sviridov , Z. Wang , K. Wlasichuk , W. Yang , X. Zhou , H. Zhu , R. T. Cirz , ChemMedChem 2019, 14, 1560–1572. 31283109 10.1002/cmdc.201900287

[502] W. Han , X. Ma , C. J. Balibar , C. M. Baxter Rath , B. Benton , A. Bermingham , F. Casey , B. Chie-Leon , M.-K. Cho , A. O. Frank , A. Frommlet , C.-M. Ho , P. S. Lee , M. Li , A. Lingel , S. Ma , H. Merritt , E. Ornelas , G. De Pascale , R. Prathapam , K. R. Prosen , D. Rasper , A. Ruzin , W. S. Sawyer , J. Shaul , X. Shen , S. Shia , M. Steffek , S. Subramanian , J. Vo , F. Wang , C. Wartchow , T. Uehara , J. Am. Chem. Soc. 2020, 142, 4445–4455. 32064871 10.1021/jacs.9b13530

[503] A. S. Nayar , T. J. Dougherty , K. E. Ferguson , B. A. Granger , L. McWilliams , C. Stacey , L. J. Leach , S.-I. Narita , H. Tokuda , A. A. Miller , D. G. Brown , S. M. McLeod , J. Bacteriol. 2015, 197, 1726–1734. 25733621 10.1128/JB.02552-14 PMC4402386

[504] M. Lee , J. Zhao , S.-H. Kwak , J. Cho , M. Lee , R. A. Gillespie , D.-Y. Kwon , H. Lee , H.-J. Park , Q. Wu , P. Zhou , J. Hong , ACS Infect. Dis. 2019, 5, 641–651. 30721024 10.1021/acsinfecdis.8b00364 PMC6730544

[505] S.-H. Kwak , C. S. Cochrane , A. F. Ennis , W. Y. Lim , C. G. Webster , J. Cho , B. A. Fenton , P. Zhou , J. Hong , Bioorg. Chem. 2020, 102, 104055. 32663666 10.1016/j.bioorg.2020.104055 PMC7484203

[506] D. L. Huseby , S. Cao , E. Zamaratski , S. Sooriyaarachchi , S. Ahmad , T. Bergfors , L. Krasnova , J. Pelss , M. Ikaunieks , E. Loza , M. Katkevics , O. Bobileva , H. Cirule , B. Gukalova , S. Grinberga , M. Backlund , I. Simoff , A. T. Leber , T. Berruga-Fernández , D. Antonov , V. R. Konda , S. Lindström , G. Olanders , P. Brandt , P. Baranczewski , C. Vingsbo Lundberg , E. Liepinsh , E. Suna , T. A. Jones , S. L. Mowbray , D. Hughes , A. Karlén , Proc. Natl. Acad. Sci. USA 2024, 121, e2317274121. 38579010 10.1073/pnas.2317274121 PMC11009625

[507] H. Ho , A. Miu , M. K. Alexander , N. K. Garcia , A. Oh , I. Zilberleyb , M. Reichelt , C. D. Austin , C. Tam , S. Shriver , H. Hu , S. S. Labadie , J. Liang , L. Wang , J. Wang , Y. Lu , H. E. Purkey , J. Quinn , Y. Franke , K. Clark , M. H. Beresini , M.-W. Tan , B. D. Sellers , T. Maurer , M. F. T. Koehler , A. T. Wecksler , J. R. Kiefer , V. Verma , Y. Xu , M. Nishiyama , J. Payandeh , C. M. Koth , Nature 2018, 557, 196–201. 29720648 10.1038/s41586-018-0083-5

[508] V. A. Verma , L. Wang , S. S. Labadie , J. Liang , B. D. Sellers , J. Wang , L. Dong , Q. Wang , S. Zhang , Z. Xu , Y. Zhang , Y. Niu , X. Wang , J. Wai , M. F. T. Koehler , H. Hu , M. K. Alexander , M. Nishiyama , A. Miu , Y. Xu , J. Pang , A. K. Katakam , M. Reichelt , C. D. Austin , H. Ho , J. Payandeh , C. M. Koth , J. Med. Chem. 2022, 65, 4085–4120. 35184554 10.1021/acs.jmedchem.1c01909

[509] M. Urfer , J. Bogdanovic , F. Lo Monte , K. Moehle , K. Zerbe , U. Omasits , C. H. Ahrens , G. Pessi , L. Eberl , J. A. Robinson , J. Biol. Chem. 2016, 291, 1921–1932. 26627837 10.1074/jbc.M115.691725 PMC4722468

[510] J. A. Robinson , S. C. Shankaramma , P. Jetter , U. Kienzl , R. A. Schwendener , J. W. Vrijbloed , D. Obrecht , Bioorg. Med. Chem. 2005, 13, 2055–2064. 15727859 10.1016/j.bmc.2005.01.009

[511] V. N. Kokryakov , S. S. Harwig , E. A. Panyutich , A. A. Shevchenko , G. M. Aleshina , O. V. Shamova , H. A. Korneva , R. I. Lehrer , FEBS Lett. 1993, 327, 231–236. 8335113 10.1016/0014-5793(93)80175-t

[512] B. Yasin , R. I. Lehrer , S. S. Harwig , E. A. Wagar , Infect. Immun. 1996, 64, 4863–4866. 8890254 10.1128/iai.64.11.4863-4866.1996 PMC174460

[513] D. A. Steinberg , M. A. Hurst , C. A. Fujii , A. H. Kung , J. F. Ho , F. C. Cheng , D. J. Loury , J. C. Fiddes , Antimicrob. Agents Chemother. 1997, 41, 1738–1742. 9257752 10.1128/aac.41.8.1738 PMC163996

[514] M. Werneburg , K. Zerbe , M. Juhas , L. Bigler , U. Stalder , A. Kaech , U. Ziegler , D. Obrecht , L. Eberl , J. A. Robinson , ChemBioChem 2012, 13, 1767–1775. 22807320 10.1002/cbic.201200276

[515] I. Martin-Loeches , G. E. Dale , A. Torres , Expert Rev. Anti-Infect. Ther. 2018, 16, 259–268. 29451043 10.1080/14787210.2018.1441024

[516] Spexis, “The Spexis Proposition: Life-changing macrocycle therapeutics for rare disease and oncology patients,” can be found under https://spexisbio.com/wp-content/uploads/2022/12/2022–11-Spexis-NC-Prez.pdf, **2022** (accessed 28 November 2024).

[517] P. Fehlbaum , P. Bulet , S. Chernysh , J. P. Briand , J. P. Roussel , L. Letellier , C. Hetru , J. A. Hoffmann , Proc. Natl. Acad. Sci. USA 1996, 93, 1221–1225. 8577744 10.1073/pnas.93.3.1221 PMC40060

[518] S. U. Vetterli , K. Zerbe , M. Müller , M. Urfer , M. Mondal , S.-Y. Wang , K. Moehle , O. Zerbe , A. Vitale , G. Pessi , L. Eberl , B. Wollscheid , J. A. Robinson , Sci. Adv. 2018, 4, eaau2634. 30443594 10.1126/sciadv.aau2634 PMC6235536

[519] B. Ma , C. Fang , L. Lu , M. Wang , X. Xue , Y. Zhou , M. Li , Y. Hu , X. Luo , Z. Hou , Nat. Commun. 2019, 10, 3517. 31388008 10.1038/s41467-019-11503-3 PMC6684654

[520] M. Schuster , E. Brabet , K. K. Oi , N. Desjonquères , K. Moehle , K. Le Poupon , S. Hell , S. Gable , V. Rithié , S. Dillinger , P. Zbinden , A. Luther , C. Li , S. Stiegeler , C. D'Arco , H. Locher , T. Remus , S. DiMaio , P. Motta , A. Wach , F. Jung , G. Upert , D. Obrecht , M. Benghezal , O. Zerbe , Sci. Adv. 2023, 9, eadg3683. 37224246 10.1126/sciadv.adg3683 PMC10208570

[521] C. Zampaloni , P. Mattei , K. Bleicher , L. Winther , C. Thäte , C. Bucher , J.-M. Adam , A. Alanine , K. E. Amrein , V. Baidin , C. Bieniossek , C. Bissantz , F. Boess , C. Cantrill , T. Clairfeuille , F. Dey , P. Di Giorgio , P. du Castel , D. Dylus , P. Dzygiel , A. Felici , F. García-Alcalde , A. Haldimann , M. Leipner , S. Leyn , S. Louvel , P. Misson , A. Osterman , K. Pahil , S. Rigo , A. Schäublin , S. Scharf , P. Schmitz , T. Stoll , A. Trauner , S. Zoffmann , D. Kahne , J. A. T. Young , M. A. Lobritz , K. A. Bradley , Nature 2024, 566–571. 10.1038/s41586-023-06873-0 PMC10794144 38172634

[522] K. S. Pahil , M. S. A. Gilman , V. Baidin , T. Clairfeuille , P. Mattei , C. Bieniossek , F. Dey , D. Muri , R. Baettig , M. Lobritz , K. Bradley , A. C. Kruse , D. Kahne , Nature 2024, 572–577. 10.1038/s41586-023-06799-7 PMC10794137 38172635

[523] P. Natale , T. Brüser , A. J. M. Driessen , Biochim. Biophys. Acta Biomembr. 2008, 1778, 1735–1756. 10.1016/j.bbamem.2007.07.015 17935691

[524] E. R. Green , J. Mecsas , Microbiol. Spectrum 2016, 4, 1–19. 10.1128/microbiolspec.VMBF-0012-2015 PMC4804464 26999395

[525] T. Yakushi , K. Masuda , S. Narita , S. Matsuyama , H. Tokuda , Nat. Cell Biol. 2000, 2, 212–218. 10783239 10.1038/35008635

[526] R. Pathania , S. Zlitni , C. Barker , R. Das , D. A. Gerritsma , J. Lebert , E. Awuah , G. Melacini , F. A. Capretta , E. D. Brown , Nat. Chem. Biol. 2009, 5, 849–856. 19783991 10.1038/nchembio.221

[527] E. B. M. Breidenstein , N. Khan , T. Duffy , C. Coward , T. Avis , O. Abdulle , C.-M. Li , C. S. Mason , Antimicrob. Agents Chemother. 2023, 68, e00695–23. 38084954 10.1128/aac.00695-23 PMC10777851

[528] K. A. Muñoz , R. J. Ulrich , A. K. Vasan , M. Sinclair , P.-C. Wen , J. R. Holmes , H. Y. Lee , C.-C. Hung , C. J. Fields , E. Tajkhorshid , G. W. Lau , P. J. Hergenrother , Nature 2024, 630, 429–436. 38811738 10.1038/s41586-024-07502-0 PMC12135874

[529] Y. Gu , H. Li , H. Dong , Y. Zeng , Z. Zhang , N. G. Paterson , P. J. Stansfeld , Z. Wang , Y. Zhang , W. Wang , C. Dong , Nature 2016, 531, 64–69. 26901871 10.1038/nature17199

[530] D. Tomasek , S. Rawson , J. Lee , J. S. Wzorek , S. C. Harrison , Z. Li , D. Kahne , Nature 2020, 583, 473–478. 32528179 10.1038/s41586-020-2370-1 PMC7367713

[531] L. Han , J. Zheng , Y. Wang , X. Yang , Y. Liu , C. Sun , B. Cao , H. Zhou , D. Ni , J. Lou , Y. Zhao , Y. Huang , Nat. Struct. Mol. Biol. 2016, 23, 192–196. 26900875 10.1038/nsmb.3181

[532] N. Noinaj , A. J. Kuszak , J. C. Gumbart , P. Lukacik , H. Chang , N. C. Easley , T. Lithgow , S. K. Buchanan , Nature 2013, 501, 385–390. 23995689 10.1038/nature12521 PMC3779476

[533] E. M. Hart , M. Gupta , M. Wühr , T. J. Silhavy , Proc. Natl. Acad. Sci. USA 2020, 117, 18737–18743. 32675245 10.1073/pnas.2007696117 PMC7414184

[534] E. M. Hart , A. M. Mitchell , A. Konovalova , M. Grabowicz , J. Sheng , X. Han , F. P. Rodriguez-Rivera , A. G. Schwaid , J. C. Malinverni , C. J. Balibar , S. Bodea , Q. Si , H. Wang , M. F. Homsher , R. E. Painter , A. K. Ogawa , H. Sutterlin , T. Roemer , T. A. Black , D. M. Rothman , S. S. Walker , T. J. Silhavy , Proc. Natl. Acad. Sci. USA 2019, 116, 21748–21757. 31591200 10.1073/pnas.1912345116 PMC6815139

[535] N. Wade , C. M. J. Wesseling , P. Innocenti , C. J. Slingerland , G. M. Koningstein , J. Luirink , N. I. Martin , ACS Infect. Dis. 2022, 8, 2242–2252. 36318734 10.1021/acsinfecdis.2c00459 PMC9673140

[536] N. Böhringer , R. Green , Y. Liu , U. Mettal , M. Marner , S. M. Modaresi , R. P. Jakob , Z. G. Wuisan , T. Maier , A. Iinishi , S. Hiller , K. Lewis , T. F. Schäberle , Microbiol. Spectrum 2021, 9, e01535–21. 10.1128/spectrum.01535-21 PMC8694152 34937193

[537] S. Groß , F. Panter , D. Pogorevc , C. E. Seyfert , S. Deckarm , C. D. Bader , J. Herrmann , R. Müller , Chem. Sci. 2021, 12, 11882–11893. 34659729 10.1039/d1sc02725e PMC8442675

[538] M. Nesic , D. B. Ryffel , J. Maturano , M. Shevlin , S. R. Pollack , D. R. Jr. Gauthier , P. Trigo-Mouriño , L.-K. Zhang , D. M. Schultz , J. M. McCabe Dunn , L.-C. Campeau , N. R. Patel , D. A. Petrone , D. Sarlah , J. Am. Chem. Soc. 2022, 144, 14026–14030. 35900216 10.1021/jacs.2c05891

[539] Y.-C. Lin , F. Schneider , K. J. Eberle , D. Chiodi , H. Nakamura , S. H. Reisberg , J. Chen , M. Saito , P. S. Baran , J. Am. Chem. Soc. 2022, 144, 14458–14462. 35926121 10.1021/jacs.2c05892 PMC9829381

[540] R. D. Miller , A. Iinishi , S. M. Modaresi , B.-K. Yoo , T. D. Curtis , P. J. Lariviere , L. Liang , S. Son , S. Nicolau , R. Bargabos , M. Morrissette , M. F. Gates , N. Pitt , R. P. Jakob , P. Rath , T. Maier , A. G. Malyutin , J. T. Kaiser , S. Niles , B. Karavas , M. Ghiglieri , S. E. J. Bowman , D. C. Rees , S. Hiller , K. Lewis , Nat. Microbiol. 2022, 7, 1661–1672. 36163500 10.1038/s41564-022-01227-4 PMC10155127

[541] B. K. Gorityala , G. Guchhait , S. Goswami , D. M. Fernando , A. Kumar , G. G. Zhanel , F. Schweizer , J. Med. Chem. 2016, 59, 8441–8455. 27524179 10.1021/acs.jmedchem.6b00867

[542] J. C. Malinverni , T. J. Silhavy , EcoSal Plus 2011, 4, 1–22. 10.1128/ecosalplus.4.3.8 PMC4231818 26442509

[543] M. V. Bertacine Dias , J. C. Santos , G. A. Libreros-Zúñiga , J. A. Ribeiro , S. M. Chavez-Pacheco , Future Med. Chem. 2018, 10, 935–959. 29629843 10.4155/fmc-2017-0168

[544] S. Noviello , D. B. Huang , G. R. Corey , Expert Rev. Anti-Infect. Ther. 2018, 16, 793–803. 30317894 10.1080/14787210.2018.1536545

[545] B. Bister , D. Bischoff , M. Ströbele , J. Riedlinger , A. Reicke , F. Wolter , A. T. Bull , H. Zähner , H. Fiedler , R. D. Süssmuth , Angew. Chem. Int. Ed. 2004, 43, 2574–2576. 10.1002/anie.200353160 15127456

[546] S. Keller , H. S. Schadt , I. Ortel , R. D. Süssmuth , Angew. Chem. Int. Ed. 2007, 46, 8284–8286. 10.1002/anie.200701836 17886307

[547] C. Sadaka , E. Ellsworth , P. R. Hansen , R. Ewin , P. Damborg , J. L. Watts , Molecules 2018, 23, 1371. 29882815 10.3390/molecules23061371 PMC6100094

[548] H. S. Butman , T. J. Kotzé , C. S. Dowd , E. Strauss , Front. Cell. Infect. Microbiol. 2020, 10, 605662. 33384970 10.3389/fcimb.2020.605662 PMC7770189

[549] C. Spry , K. Kirk , K. J. Saliba , FEMS Microbiol. Rev. 2008, 32, 56–106. 18173393 10.1111/j.1574-6976.2007.00093.x

[550] T. P. Soares da Costa , W. Tieu , M. Y. Yap , O. Zvarec , J. M. Bell , J. D. Turnidge , J. C. Wallace , G. W. Booker , M. C. J. Wilce , A. D. Abell , S. W. Polyak , ACS Med. Chem. Lett. 2012, 3, 509–514. 24900501 10.1021/ml300106p PMC4025796

[551] J. Feng , A. S. Paparella , G. W. Booker , S. W. Polyak , A. D. Abell , Antibiotics 2016, 5, 26. 27463729 10.3390/antibiotics5030026 PMC5039522

[552] A. J. Hayes , J. Satiaputra , L. M. Sternicki , A. S. Paparella , Z. Feng , K. J. Lee , B. Blanco-Rodriguez , W. Tieu , B. A. Eijkelkamp , K. E. Shearwin , T. L. Pukala , A. D. Abell , G. W. Booker , S. W. Polyak , Antibiotics 2020, 9, 165. 32268615 10.3390/antibiotics9040165 PMC7235819

[553] M. I. Goncheva , D. Chin , D. E. Heinrichs , Trends Microbiol. 2022, 30, 793–804. 35074276 10.1016/j.tim.2021.12.007

[554] J. M. Thomson , I. L. Lamont , Front. Microbiol. 2019, 10 : 952. 10.3389/fmicb.2019.00952 PMC6540614 31191461

[555] V. Heinemann , Y. Z. Xu , S. Chubb , A. Sen , L. W. Hertel , G. B. Grindey , W. Plunkett , Mol. Pharmacol. 1990, 38, 567–572. 2233693

[556] M. P. B. Sandrini , A. R. Clausen , S. L. W. On , F. M. Aarestrup , B. Munch-Petersen , J. Piskur , J. Antimicrob. Chemother. 2007, 60, 510–520. 17615154 10.1093/jac/dkm240

[557] M. P. B. Sandrini , O. Shannon , A. R. Clausen , L. Björck , J. Piskur , Antimicrob. Agents Chemother. 2007, 51, 2726–2732. 17526755 10.1128/AAC.00081-07 PMC1932510

[558] K. Mandapati , S. K. Gorla , A. L. House , E. S. McKenney , M. Zhang , S. N. Rao , D. R. Gollapalli , B. J. Mann , J. B. Goldberg , G. D. Cuny , I. J. Glomski , L. Hedstrom , ACS Med. Chem. Lett. 2014, 5, 846–850. 25147601 10.1021/ml500203p PMC4137380

[559] G. Martínez-Botella , J. N. Breen , J. E. S. Duffy , J. Dumas , B. Geng , I. K. Gowers , O. M. Green , S. Guler , M. F. Hentemann , F. A. Hernandez-Juan , D. Joseph-McCarthy , S. Kawatkar , N. A. Larsen , O. Lazari , J. T. Loch , J. A. Macritchie , A. R. McKenzie , J. V. Newman , N. B. Olivier , L. G. Otterson , A. P. Owens , J. Read , D. W. Sheppard , T. A. Keating , J. Med. Chem. 2012, 55, 10010–10021. 23043329 10.1021/jm3011806

[560] Y. Gotoh , Y. Eguchi , T. Watanabe , S. Okamoto , A. Doi , R. Utsumi , Curr. Opin. Microbiol. 2010, 13, 232–239. 20138000 10.1016/j.mib.2010.01.008

[561] T. Watanabe , M. Igarashi , T. Okajima , E. Ishii , H. Kino , M. Hatano , R. Sawa , M. Umekita , T. Kimura , S. Okamoto , Y. Eguchi , Y. Akamatsu , R. Utsumi , Antimicrob. Agents Chemother. 2012, 56, 3657–3663. 22526318 10.1128/AAC.06467-11 PMC3393415

[562] Y. Gotoh , A. Doi , E. Furuta , S. Dubrac , Y. Ishizaki , M. Okada , M. Igarashi , N. Misawa , H. Yoshikawa , T. Okajima , T. Msadek , R. Utsumi , J. Antibiot. 2010, 63, 127–134. 10.1038/ja.2010.4 20111065

[563] A. Berthier , F. Oger , C. Gheeraert , A. Boulahtouf , R. Le Guével , P. Balaguer , B. Staels , G. Salbert , P. Lefebvre , Toxicol. Sci. 2012, 127, 225–235. 22314385 10.1093/toxsci/kfs073 PMC3435511

[564] J. Janardhanan , C. Kim , Y. Qian , J. Yang , J. E. Meisel , D. Ding , E. Speri , V. A. Schroeder , W. R. Wolter , A. G. Oliver , S. Mobashery , M. Chang , Proc. Natl. Acad. Sci. USA 2023, 120, e2304110120. 37155891 10.1073/pnas.2304110120 PMC10193928

[565] K. S. H. Beckham , J. P. R. Connolly , J. M. Ritchie , D. Wang , J. A. Gawthorne , A. Tahoun , D. L. Gally , K. Burgess , R. J. Burchmore , B. O. Smith , S. A. Beatson , O. Byron , A. J. Wolfe , G. R. Douce , A. J. Roe , Mol. Microbiol. 2014, 93, 199–211. 24846743 10.1111/mmi.12651 PMC4249723

[566] A. T. Bakker , I. Kotsogianni , L. Mirenda , V. M. Straub , M. Avalos , R. J. B. H. N. van den Berg , B. I. Florea , G. P. van Wezel , A. P. A. Janssen , N. I. Martin , M. van der Stelt , J. Am. Chem. Soc. 2023, 145, 1136–1143. 36584241 10.1021/jacs.2c10819 PMC9853856

[567] C. E. Rowland , H. Newman , T. T. Martin , R. Dods , N. Bournakas , J. M. Wagstaff , N. Lewis , S. J. Stanway , M. Balmforth , C. Kessler , K. van Rietschoten , D. Bellini , D. I. Roper , A. J. Lloyd , C. G. Dowson , M. J. Skynner , P. Beswick , M. J. Dawson , bioRxiv preprint 2024, DOI: 10.1101/2024.03.20.581580.

[568] N. K. Prasad , I. B. Seiple , R. T. Cirz , O. S. Rosenberg , Antimicrob. Agents Chemother. 2022, 66, e00054–22. 35471042 10.1128/aac.00054-22 PMC9112940

[569] “CARB-X,” can be found under https://carb-x.org/, **2024** (accessed 28 November 2024).

[570] “GARDP Global Antibiotic Research and Development Partnership,” can be found under https://gardp.org/, **2024** (accessed28 November 2024).

[571] Y. Ishizaki , C. Hayashi , K. Inoue , M. Igarashi , Y. Takahashi , V. Pujari , D. C. Crick , P. J. Brennan , A. Nomoto , J. Biol. Chem. 2013, 288, 30309–30319. 23986448 10.1074/jbc.M113.492173 PMC3798496

[572] M. Igarashi , N. Nakagawa , N. Doi , S. Hattori , H. Naganawa , M. Hamada , J. Antibiot. 2003, 56, 580–583. 10.7164/antibiotics.56.580 12931868

[573] Y. Takahashi , M. Igarashi , T. Miyake , H. Soutome , K. Ishikawa , Y. Komatsuki , Y. Koyama , N. Nakagawa , S. Hattori , K. Inoue , N. Doi , Y. Akamatsu , J. Antibiot. 2013, 66, 171–178. 10.1038/ja.2013.9 23532021

[574] J. Janardhanan , J. E. Meisel , D. Ding , V. A. Schroeder , W. R. Wolter , S. Mobashery , M. Chang , Antimicrob. Agents Chemother. 2016, 60, 5581–5588. 27401567 10.1128/AAC.00787-16 PMC4997879

[575] S. Ceballos , C. Kim , D. Ding , S. Mobashery , M. Chang , C. Torres , Antimicrob. Agents Chemother. 2018, 62:e00453–18. 29866865 10.1128/AAC.00453-18 PMC6105842

[576] J. W. Schmidt , A. Greenough , M. Burns , A. E. Luteran , D. G. McCafferty , FEMS Microbiol. Lett. 2010, 310, 104–111. 20659164 10.1111/j.1574-6968.2010.02051.x PMC3596103

[577] M. T. Wong , C. A. Kauffman , H. C. Standiford , P. Linden , G. Fort , H. J. Fuchs , S. B. Porter , R. P. Wenzel , T. R. V. C. S. Group , Clin. Infect. Dis. 2001, 33, 1476–1482. 11588692 10.1086/322687

[578] R. N. Jones , A. L. Barry , Diagn. Microbiol. Infect. Dis. 1989, 12, 279–282. 2507218 10.1016/0732-8893(89)90029-1

[579] D. K. Farver , D. D. Hedge , S. C. Lee , Ann. Pharmacother. 2005, 39, 863–868. 15784805 10.1345/aph.1E397

[580] Y. Hu , J. S. Helm , L. Chen , X.-Y. Ye , S. Walker , J. Am. Chem. Soc. 2003, 125, 8736–8737. 12862463 10.1021/ja035217i

[581] J. Freeman , S. D. Baines , D. Jabes , M. H. Wilcox , J. Antimicrob. Chemother. 2005, 56, 717–725. 16143709 10.1093/jac/dki321

[582] M. P. Singh , P. J. Petersen , W. J. Weiss , J. E. Janso , S. W. Luckman , E. B. Lenoy , P. A. Bradford , R. T. Testa , M. Greenstein , Antimicrob. Agents Chemother. 2003, 47, 62–69. 12499170 10.1128/AAC.47.1.62-69.2003 PMC148986

[583] A. Katsuyama , A. Paudel , S. Panthee , H. Hamamoto , T. Kawakami , H. Hojo , F. Yakushiji , S. Ichikawa , Org. Lett. 2017, 19, 3771–3774. 28696114 10.1021/acs.orglett.7b01629

[584] F. V. Nussbaum, N. Brunner, R. Endermann, C. Fürstner, E. Hartmann, J. Ragot, G. Schiffer, J. Schuhmacher, N. Svenstrup, J. Telser, S. Anlauf, M.-A. Brüning, (Aicuris GmbH), EP1809654B1, **2008**.

[585] D. P. Bonner , J. O'Sullivan , S. K. Tanaka , J. M. Clark , R. R. Whitney , J. Antibiot. 1988, 41, 1745–1751. 10.7164/antibiotics.41.1745 3209466

[586] H. Mathur , P. M. O'Connor , C. Hill , P. D. Cotter , R. P. Ross , Antimicrob. Agents Chemother. 2013, 57, 2882–2886. 23571539 10.1128/AAC.00261-13 PMC3716125

[587] Novacta Biosystems Limited. Assessment of the Safety and Distribution of NVB302 in Healthy Volunteers. Identifyer ISRCTN40071144. Updated April 27 **2016**. Accessed November 28, 2024. https://www.isrctn.com/ISRCTN40071144.

[588] D. Jabés , C. Brunati , G. Candiani , S. Riva , G. Romanó , S. Donadio , Antimicrob. Agents Chemother. 2011, 55, 1671–1676. 21220527 10.1128/AAC.01288-10 PMC3067139

[589] D. Münch , A. Müller , T. Schneider , B. Kohl , M. Wenzel , J. E. Bandow , S. Maffioli , M. Sosio , S. Donadio , R. Wimmer , H.-G. Sahl , J. Biol. Chem. 2014, 289, 12063–12076. 24627484 10.1074/jbc.M113.537449 PMC4002112

[590] F. Castiglione , A. Lazzarini , L. Carrano , E. Corti , I. Ciciliato , L. Gastaldo , P. Candiani , D. Losi , F. Marinelli , E. Selva , F. Parenti , Chem. Biol. 2008, 15, 22–31. 18215770 10.1016/j.chembiol.2007.11.009

[591] N. L. Elsen , J. Lu , G. Parthasarathy , J. C. Reid , S. Sharma , S. M. Soisson , K. J. Lumb , J. Am. Chem. Soc. 2012, 134, 12342–12345. 22793495 10.1021/ja303564a

[592] J. M. Andreu , C. Schaffner-Barbero , S. Huecas , D. Alonso , M. L. Lopez-Rodriguez , L. B. Ruiz-Avila , R. Núñez-Ramírez , O. Llorca , A. J. Martín-Galiano , J. Biol. Chem. 2010, 285, 14239–14246. 20212044 10.1074/jbc.M109.094722 PMC2863232

[593] M. Kaul , L. Mark , Y. Zhang , A. K. Parhi , E. J. LaVoie , D. S. Pilch , Antimicrob. Agents Chemother. 2013, 57, 5860–5869. 24041882 10.1128/AAC.01016-13 PMC3837898

[594] D. J. Haydon , N. R. Stokes , R. Ure , G. Galbraith , J. M. Bennett , D. R. Brown , P. J. Baker , V. V. Barynin , D. W. Rice , S. E. Sedelnikova , J. R. Heal , J. M. Sheridan , S. T. Aiwale , P. K. Chauhan , A. Srivastava , A. Taneja , I. Collins , J. Errington , L. G. Czaplewski , Science 2008, 321, 1673–1675. 18801997 10.1126/science.1159961

[595] C. M. Tan , A. G. Therien , J. Lu , S. H. Lee , A. Caron , C. J. Gill , C. Lebeau-Jacob , L. Benton-Perdomo , J. M. Monteiro , P. M. Pereira , N. L. Elsen , J. Wu , K. Deschamps , M. Petcu , S. Wong , E. Daigneault , S. Kramer , L. Liang , E. Maxwell , D. Claveau , J. Vaillancourt , K. Skorey , J. Tam , H. Wang , T. C. Meredith , S. Sillaots , L. Wang-Jarantow , Y. Ramtohul , E. Langlois , F. Landry , J. C. Reid , G. Parthasarathy , S. Sharma , A. Baryshnikova , K. J. Lumb , M. G. Pinho , S. M. Soisson , T. Roemer , Sci. Transl. Med. 2012, 4, 126ra35–126ra35. 10.1126/scitranslmed.3003592 22440737

[596] D. Song , F. Bi , N. Zhang , Y. Qin , X. Liu , Y. Teng , S. Ma , Bioorg. Med. Chem. 2020, 28, 115729. 33065440 10.1016/j.bmc.2020.115729

[597] M. Kaul , L. Mark , Y. Zhang , A. K. Parhi , E. J. LaVoie , D. S. Pilch , Biochem. Pharmacol. 2013, 86, 1699–1707. 24148278 10.1016/j.bcp.2013.10.010

[598] A. J. Lepak , A. Parhi , M. Madison , K. Marchillo , J. VanHecker , D. R. Andes , Antimicrob. Agents Chemother. 2015, 59, 6568–6574. 26259789 10.1128/AAC.01464-15 PMC4576109

[599] J. Fujita , Y. Maeda , E. Mizohata , T. Inoue , M. Kaul , A. K. Parhi , E. J. LaVoie , D. S. Pilch , H. Matsumura , ACS Chem. Biol. 2017, 12, 1947–1955. 28621933 10.1021/acschembio.7b00323 PMC5705026

[600] V. Makarov , G. Manina , K. Mikusova , U. Möllmann , O. Ryabova , B. Saint-Joanis , N. Dhar , M. R. Pasca , S. Buroni , A. P. Lucarelli , A. Milano , E. De Rossi , M. Belanova , A. Bobovska , P. Dianiskova , J. Kordulakova , C. Sala , E. Fullam , P. Schneider , J. D. McKinney , P. Brodin , T. Christophe , S. Waddell , P. Butcher , J. Albrethsen , I. Rosenkrands , R. Brosch , V. Nandi , S. Bharath , S. Gaonkar , R. K. Shandil , V. Balasubramanian , T. Balganesh , S. Tyagi , J. Grosset , G. Riccardi , S. T. Cole , Science 2009, 324, 801–804. 19299584 10.1126/science.1171583 PMC3128490

[601] R. Tiwari , G. C. Moraski , V. Krchňák , P. A. Miller , M. Colon-Martinez , E. Herrero , A. G. Oliver , M. J. Miller , J. Am. Chem. Soc. 2013, 135, 3539–3549. 23402278 10.1021/ja311058q PMC3677520

[602] M. Hoelscher. A Phase IIb, Open-Label, Randomized Controlled Dose Ranging Multi-Center Trial to Evaluate the Safety, Tolerability, Pharmacokinetics and Exposure-Response Relationship of Different Doses of BTZ-043 in Combination With Bedaquiline and Delamanid in Adult Subjects With Newly Diagnosed, Uncomplicated, Drug-Sensitive Pulmonary Tuberculosis, ClinicalTrials.gov identifyer: NCT05926466. Updated December 22, **2023**. Accessed November 28, 2024. https://clinicaltrials.gov/study/NCT05926466. 10.1186/s13063-023-07354-5 PMC10243693 37280643

[603] Nearmedic Plus LLC. Multicenter, Open, Randomized Study With Active Control to Evaluate the Early Bactericidal Activity, Safety and Pharmacokinetics of the Drug PBTZ169 When Used in Patients With First-Diagnosed Tuberculosis of the Respiratory System With Bacterial Excretion and Saved Bacterial Susceptibility to Isoniazid and Rifampicin. ClinicalTrials.gov identifyer: NCT03334734. Updated: March 9, **2020**. Accessed November 28, 2024. https://clinicaltrials.gov/study/NCT03334734.

[604] G. T. Robertson , M. E. Ramey , L. M. Massoudi , C. L. Carter , M. Zimmerman , F. Kaya , B. G. Graham , V. Gruppo , C. Hastings , L. K. Woolhiser , D. W. L. Scott , B. C. Asay , F. Eshun-Wilson , E. Maidj , B. K. Podell , J. J. Vásquez , M. A. Lyons , V. Dartois , A. J. Lenaerts , Antimicrob. Agents Chemother. 2021, 65, 1–21. 10.1128/AAC.00583-21 PMC8522729 34370580

[605] M. Chatterji , R. Shandil , M. R. Manjunatha , S. Solapure , V. Ramachandran , N. Kumar , R. Saralaya , V. Panduga , J. Reddy , K.R. Prabhakar , S. Sharma , C. Sadler , C. B. Cooper , K. Mdluli , P. S. Iyer , S. Narayanan , P. S. Shirude , Antimicrob. Agents Chemother. 2014, 58, 5325–5331. 24957839 10.1128/AAC.03233-14 PMC4135869

[606] P. S. Shirude , R. K. Shandil , M. R. Manjunatha , C. Sadler , M. Panda , V. Panduga , J. Reddy , R. Saralaya , R. Nanduri , A. Ambady , S. Ravishankar , V. K. Sambandamurthy , V. Humnabadkar , L. K. Jena , R. S. Suresh , A. Srivastava , K. R. Prabhakar , J. Whiteaker , R. E. McLaughlin , S. Sharma , C. B. Cooper , K. Mdluli , S. Butler , P. S. Iyer , S. Narayanan , M. Chatterji , J. Med. Chem. 2014, 57, 5728–5737. 24874895 10.1021/jm500571f

[607] Bill & Melinda Gates Medical Research Institute. A Phase 2a, Dose Escalation, Controlled, Randomized Study to Evaluate Safety, Early Bactericidal Activity (EBA) and Pharmacokinetics of TBA-7371 in Adult Patients With Rifampicin-Sensitive Pulmonary Tuberculosis. ClinicalTrials.gov identifyer: NCT04176250. Updated April 11 **2024**. Accessed November 28, 2024. https://clinicaltrials.gov/study/NCT04176250.

[608] N. Hariguchi , X. Chen , Y. Hayashi , Y. Kawano , M. Fujiwara , M. Matsuba , H. Shimizu , Y. Ohba , I. Nakamura , R. Kitamoto , T. Shinohara , Y. Uematsu , S. Ishikawa , M. Itotani , Y. Haraguchi , I. Takemura , M. Matsumoto , Antimicrob. Agents Chemother. 2020, 64, 1–13. 10.1128/AAC.02020-19 PMC7269503 32229496

[609] Otsuka Pharmaceutical Development & Commercialization, Inc. Safety and Efficacy Evaluation of 4-month Regimen of OPC-167832, Delamanid and Bedaquiline in Participants With Drug-Susceptible Pulmonary TB. ClinicalTrials.gov identifyer: NCT05221502. Updated June 27, **2024**. Accessed November 28, 2024. https://clinicaltrials.gov/study/NCT05221502.

[610] S. Nakamura , N. Tanaka , H. Umezawa , J. Antibiot. 1966, 19, 10–12. 5952015

[611] T. Kinoshita , N. Tanaka , J. Antibiot. 1970, 23, 311–312. 10.7164/antibiotics.23.311 5458308

[612] Y. Kobayashi , M. Ichioka , T. Hirose , K. Nagai , A. Matsumoto , H. Matsui , H. Hanaki , R. Masuma , Y. Takahashi , S. Ōmura , T. Sunazuka , Bioorg. Med. Chem. Lett.. 2010, 20, 6116–6120. 20833545 10.1016/j.bmcl.2010.08.037

[613] M. Hamada , T. Takeuchi , S. Kondo , Y. Ikeda , H. Naganawa , J. Antibiot. 1970, 23, 170–171. 10.7164/antibiotics.23.170 5453311

[614] N. B. Olivier , R. B. Altman , J. Noeske , G. S. Basarab , E. Code , A. D. Ferguson , N. Gao , J. Huang , M. F. Juette , S. Livchak , M. D. Miller , D. B. Prince , J. H. D. Cate , E. T. Buurman , S. C. Blanchard , Proc. Natl. Acad. Sci. USA 2014, 111, 16274–16279. 25368144 10.1073/pnas.1414401111 PMC4246262

[615] D. Hörömpöli , C. Ciglia , K.-H. Glüsenkamp , L. O. Haustedt , H. Falkenstein-Paul , G. Bendas , A. Berscheid , H. Brötz-Oesterhelt , Antimicrob. Agents Chemother. 2021, 65, e00986–20. 10.1128/AAC.00986-20 PMC8097410 33468467

[616] S. J. Schroeder , G. Blaha , P. B. Moore , Antimicrob. Agents Chemother. 2007, 51, 4462–4465. 17664317 10.1128/AAC.00455-07 PMC2167971

[617] L. Pantel , P. Juarez , M. Serri , L. Boucinha , E. Lessoud , A. Lanois , A. Givaudan , E. Racine , M. Gualtieri , Antimicrob. Agents Chemother. 2021, 65, 1–11. 10.1128/AAC.00139-21 PMC8092918 33685902

[618] E. Racine , P. Nordmann , L. Pantel , M. Sarciaux , M. Serri , J. Houard , P. Villain-Guillot , A. Demords , C. Vingsbo Lundberg , M. Gualtieri , Antimicrob. Agents Chemother. 2018, 62, 1–16. 10.1128/AAC.01016-18 PMC6125496 29987155

[619] T. Dang , B. Loll , S. Müller , R. Skobalj , J. Ebeling , T. Bulatov , S. Gensel , J. Göbel , M. C. Wahl , E. Genersch , A. Mainz , R. D. Süssmuth , Nat. Commun. 2022, 13, 2349. 35487884 10.1038/s41467-022-29829-w PMC9054821

[620] H. Irschik , H. Reichenbach , J. Antibiot. 1985, 38, 1237–1245. 10.7164/antibiotics.38.1237 2415501

[621] H. Irschik , K. Gerth , T. Kemmer , H. Steinmetz , H. Reichenbach , J. Antibiot. 1983, 36, 6–12. 10.7164/antibiotics.36.6 6432761

[622] I. A. Critchley , U. A. Ochsner , Curr. Opin. Chem. Biol. 2008, 12, 409–417. 18620074 10.1016/j.cbpa.2008.06.011

[623] R. L. Jarvest , J. M. Berge , V. Berry , H. F. Boyd , M. J. Brown , J. S. Elder , A. K. Forrest , A. P. Fosberry , D. R. Gentry , M. J. Hibbs , D. D. Jaworski , P. J. O'Hanlon , A. J. Pope , S. Rittenhouse , R. J. Sheppard , C. Slater-Radosti , A. Worby , J. Med. Chem. 2002, 45, 1959–1962. 11985462 10.1021/jm025502x

[624] U. A. Ochsner , S. J. Bell , A. L. O'Leary , T. Hoang , K. C. Stone , C. L. Young , I. A. Critchley , N. Janjic , J. Antimicrob. Chemother. 2009, 63, 964–971. 19251726 10.1093/jac/dkp042

[625] Crestone, Inc. A Phase 2, Randomized, Double-Blind, Comparator-Controlled, Multicenter Study to Evaluate the Safety and Efficacy of CRS3123 Compared With Oral Vancomycin in Adults With Clostridioides Difficile Infection. ClinicalTrials.gov identifyer: NCT04781387. Updated February 22, **2024**. Accessed November 28, 2024. https://clinicaltrials.gov/study/NCT04781387.

[626] A. Parmeggiani , I. M. Krab , S. Okamura , R. C. Nielsen , J. Nyborg , P. Nissen , Biochemistry 2006, 45, 6846–6857. 16734421 10.1021/bi0525122

[627] E. Selva , G. Beretta , N. Montanini , G. S. Saddler , L. Gastaldo , P. Ferrari , R. Lorenzetti , P. Landini , F. Ripamonti , B. P. Goldstein , M. Berti , L. Montanaro , M. Denaro , J. Antibiot. 1991, 44, 693–701. 10.7164/antibiotics.44.693 1908853

[628] J. A. Leeds , M. J. LaMarche , J. T. Brewer , S. M. Bushell , G. Deng , J. M. Dewhurst , J. Dzink-Fox , E. Gangl , A. Jain , L. Lee , M. Lilly , K. Manni , S. Mullin , G. Neckermann , C. Osborne , D. Palestrant , M. A. Patane , A. Raimondi , S. Ranjitkar , E. M. Rann , M. Sachdeva , J. Shao , S. Tiamfook , L. Whitehead , D. Yu , Antimicrob. Agents Chemother. 2011, 55, 5277–5283. 21825297 10.1128/AAC.00582-11 PMC3195004

[629] J. A. Leeds , M. Sachdeva , S. Mullin , J. Dzink-Fox , M. J. LaMarche , Antimicrob. Agents Chemother. 2012, 56, 4463–4465. 22644023 10.1128/AAC.06354-11 PMC3421628

[630] Novartis Pharmaceuticals. Multi-Center, Randomized, Evaluator-Blind, Active-Controlled, Parallel-Group Design to Determine Safety, Tolerability, and Efficacy of Multiple Daily Administration of LFF571 for 10 Days in Patients With Moderate Clostridium Difficile Infections. ClinicalTrials.gov (Identifier NCT01232595), December 19, **2020**. Accessed November 28, 2024. https://clinicaltrials.gov/study/NCT01232595.

[631] D. Z. Chen , D. V. Patel , C. J. Hackbarth , W. Wang , G. Dreyer , D. C. Young , P. S. Margolis , C. Wu , Z.-J. Ni , J. Trias , R. J. White , Z. Yuan , Biochemistry 2000, 39, 1256–1262. 10684604 10.1021/bi992245y

[632] F. Wolf , F. Leipoldt , A. Kulik , D. Wibberg , J. Kalinowski , L. Kaysser , ChemBioChem 2018, 19, 1189–1195. 10.1002/cbic.201800116 29600569

[633] B. J. Broughton , P. Chaplen , W. A. Freeman , P. J. Warren , K. R. H. Wooldridge , D. E. Wright , J. Chem. Soc. Perkin Trans. 1 1975, 857–860. 1095607

[634] P. S. Margolis , C. J. Hackbarth , D. C. Young , W. Wang , D. Chen , Z. Yuan , R. White , J. Trias , Antimicrob. Agents Chemother. 2000, 44, 1825–1831. 10858337 10.1128/aac.44.7.1825-1831.2000 PMC89968

[635] P. Margolis , C. Hackbarth , S. Lopez , M. Maniar , W. Wang , Z. Yuan , R. White , J. Trias , Antimicrob. Agents Chemother. 2001, 45, 2432–2435. 11502510 10.1128/AAC.45.9.2432-2435.2001 PMC90673

[636] E. Azoulay-Dupuis , J. Mohler , J. P. Bédos , Antimicrob. Agents Chemother. 2004, 48, 80–85. 14693522 10.1128/AAC.48.1.80-85.2004 PMC310171

[637] S. Ramanathan-Girish , J. McColm , J. M. Clements , P. Taupin , S. Barrowcliffe , J. Hevizi , S. Safrin , C. Moore , G. Patou , H. Moser , A. Gadd , U. Hoch , V. Jiang , D. Lofland , K. W. Johnson , Antimicrob. Agents Chemother. 2004, 48, 4835–4842. 15561864 10.1128/AAC.48.12.4835-4842.2004 PMC529202

[638] T. R. Fritsche , H. S. Sader , R. Cleeland , R. N. Jones , Antimicrob. Agents Chemother. 2005, 49, 1468–1476. 15793128 10.1128/AAC.49.4.1468-1476.2005 PMC1068652

[639] C. J. Hackbarth , D. Z. Chen , J. G. Lewis , K. Clark , J. B. Mangold , J. A. Cramer , P. S. Margolis , W. Wang , J. Koehn , C. Wu , S. Lopez , G. Withers , H. Gu , E. Dunn , R. Kulathila , S.-H. Pan , W. L. Porter , J. Jacobs , J. Trias , D. V. Patel , B. Weidmann , R. J. White , Z. Yuan , Antimicrob. Agents Chemother. 2002, 46, 2752–2764. 12183225 10.1128/AAC.46.9.2752-2764.2002 PMC127453

[640] K. Kosowska-Shick , K. L. Credito , G. A. Pankuch , B. DeWasse , P. McGhee , P. C. Appelbaum , Antimicrob. Agents Chemother. 2007, 51, 770–773. 17116666 10.1128/AAC.01150-06 PMC1797776

[641] M. M. Butler , W. A. LaMarr , K. A. Foster , M. H. Barnes , D. J. Skow , P. T. Lyden , L. M. Kustigian , C. Zhi , N. C. Brown , G. E. Wright , T. L. Bowlin , Antimicrob. Agents Chemother. 2007, 51, 119–127. 17074800 10.1128/AAC.01311-05 PMC1797695

[642] M. M. Butler , D. J. Skow , R. O. Stephenson , P. T. Lyden , W. A. LaMarr , K. A. Foster , Antimicrob. Agents Chemother. 2002, 46, 3770–3775. 12435675 10.1128/AAC.46.12.3770-3775.2002 PMC132772

[643] Acurx Pharmaceuticals Inc. ACX-362E [Ibezapolstat] for Oral Treatment of Clostridioides Difficile Infection: A Phase 2A Open-Label Segment Followed by a Phase 2B Double-Blind Vancomycin-Controlled Segment. ClinicalTrials.gov identifyer: NCT04247542. Updated May 14, **2024**. Accessed November 28, 2024. https://clinicaltrials.gov/study/NCT04247542.

[644] G. Jolles (Rhonepoulenc S. A.), GB1252553A, **1971**.

[645] S. D. Podos , J. A. Thanassi , M. J. Pucci , Antimicrob. Agents Chemother. 2012, 56, 4095–4102. 22585221 10.1128/AAC.00215-12 PMC3421614

[646] M. Cavero-Tomas, M. Gowravaram, H. Huynh, H. Ni, S. Stokes (AstraZeneca Ab, AstraZeneca UK Limited), WO2006040558A1, **2006**.

[647] L. Miesel , D. W. Hecht , J. R. Osmolski , D. Gerding , A. Flattery , F. Li , J. Lan , P. Lipari , J. D. Polishook , L. Liang , J. Liu , D. B. Olsen , S. B. Singh , Antimicrob. Agents Chemother. 2014, 58, 2387–2392. 24514098 10.1128/AAC.00021-14 PMC4023737

[648] R. Sawa , Y. Takahashi , H. Hashizume , K. Sasaki , Y. Ishizaki , M. Umekita , M. Hatano , H. Abe , T. Watanabe , N. Kinoshita , Y. Homma , C. Hayashi , K. Inoue , S. Ohba , T. Masuda , M. Arakawa , Y. Kobayashi , M. Hamada , M. Igarashi , H. Adachi , Y. Nishimura , Y. Akamatsu , Chem. Eur. J. 2012, 18, 15772–15781. 23129443 10.1002/chem.201202645

[649] M. Igarashi, R. Sawa, Y. Homma, (Microbial Chemistry Research Foundation), EP2423319B1, **2014**.

[650] S. B. Singh , Bioorg. Med. Chem. 2016, 24, 6291–6297. 27143131 10.1016/j.bmc.2016.04.043

[651] P. Szili , G. Draskovits , T. Révész , F. Bogár , D. Balogh , T. Martinek , L. Daruka , R. Spohn , B. M. Vásárhelyi , M. Czikkely , B. Kintses , G. Grézal , G. Ferenc , C. Pál , Á. Nyerges , Antimicrob. Agents Chemother. 2019, 63, e00207–19. 31235632 10.1128/AAC.00207-19 PMC6709476

[652] D. J. Payne, S. Rittenhouse, N. Scangarella-Oman, (GlaxoSmithKline Intellectual Property Development Limited), US20220184071A1, **2022**.

[653] D. J. Farrell , H. S. Sader , P. R. Rhomberg , N. E. Scangarella-Oman , R. K. Flamm , Antimicrob. Agents Chemother. 2017, 61, e02047–16. 28483959 10.1128/AAC.00468-17 PMC5487655

[654] N. E. Scangarella-Oman , M. Hossain , J. L. Hoover , C. R. Perry , C. Tiffany , A. Barth , E. F. Dumont , Antimicrob. Agents Chemother. 2022, 66, e01492–21. 34978887 10.1128/aac.01492-21 PMC8923173

[655] D. J. Biedenbach , S. K. Bouchillon , M. Hackel , L. A. Miller , N. E. Scangarella-Oman , C. Jakielaszek , D. F. Sahm , Antimicrob. Agents Chemother. 2016, 60, 1918–1923. 26729499 10.1128/AAC.02820-15 PMC4776004

[656] E. G. Gibson , B. Bax , P. F. Chan , N. Osheroff , ACS Infect. Dis. 2019, 5, 570–581. 30757898 10.1021/acsinfecdis.8b00315 PMC6461504

[657] “NIH Statement on Preliminary Efficacy Results of First-in-Class Gonorrhea Antibiotic Developed Through Public-Private Partnership. NIH: National Institute of Allergy and Infectious Diseases,” can be found under https://www.niaid.nih.gov/news-events/nih-statement-preliminary-efficacy-results-first-class-gonorrhea-antibiotic-developed, **2023**.

[658] G. S. Basarab , G. H. Kern , J. McNulty , J. P. Mueller , K. Lawrence , K. Vishwanathan , R. A. Alm , K. Barvian , P. Doig , V. Galullo , H. Gardner , M. Gowravaram , M. Huband , A. Kimzey , M. Morningstar , A. Kutschke , S. D. Lahiri , M. Perros , R. Singh , V. J. A. Schuck , R. Tommasi , G. Walkup , J. V. Newman , Sci. Rep. 2015, 5, 11827. 26168713 10.1038/srep11827 PMC4501059

[659] S. N. Taylor , J. Marrazzo , B. E. Batteiger , E. W. Hook , A. C. Seña , J. Long , M. R. Wierzbicki , H. Kwak , S. M. Johnson , K. Lawrence , J. Mueller , N. Engl. J. Med. 2018, 379, 1835–1845. 30403954 10.1056/NEJMoa1706988

[660] G. S. Basarab , P. Doig , C. J. Eyermann , V. Galullo , G. Kern , A. Kimzey , A. Kutschke , M. Morningstar , V. Schuck , K. Vishwanathan , F. Zhou , M. Gowravaram , S. Hauck , J. Med. Chem. 2020, 63, 11882–11901. 32914979 10.1021/acs.jmedchem.0c01100

[661] M. Moeller , M. D. Norris , T. Planke , K. Cirnski , J. Herrmann , R. Müller , A. Kirschning , Org. Lett. 2019, 21, 8369–8372. 31599597 10.1021/acs.orglett.9b03143

[662] S. Hüttel , G. Testolin , J. Herrmann , T. Planke , F. Gille , M. Moreno , M. Stadler , M. Brönstrup , A. Kirschning , R. Müller , Angew. Chem. Int. Ed. 2017, 56, 12760–12764; 10.1002/anie.201705913 28730677

[663] A. Srivastava , M. Talaue , S. Liu , D. Degen , R. Y. Ebright , E. Sineva , A. Chakraborty , S. Y. Druzhinin , S. Chatterjee , J. Mukhopadhyay , Y. W. Ebright , A. Zozula , J. Shen , S. Sengupta , R. R. Niedfeldt , C. Xin , T. Kaneko , H. Irschik , R. Jansen , S. Donadio , N. Connell , R. H. Ebright , Curr. Opin. Microbiol. 2011, 14, 532–543. 21862392 10.1016/j.mib.2011.07.030 PMC3196380

[664] R. H. Ebright, Y. W. Ebright, J. Shen, J. Bacci, A.-C. Hiebel, W. Solvibile, C. Self, G. Olson (Rutgers, the State University of New Jersey; Provid Pharmaceuticals Inc.), WO2013192352A1, **2013**.

[665] T. Hu , J. V. Schaus , K. Lam , M. G. Palfreyman , M. Wuonola , G. Gustafson , J. S. Panek , J. Org. Chem. 1998, 63, 2401–2406.

[666] M. Brauer , J. Herrmann , D. Zühlke , R. Müller , K. Riedel , S. Sievers , Gut Pathog. 2022, 14, 4. 34991700 10.1186/s13099-021-00475-9 PMC8739712

[667] A. Srivastava , D. Degen , Y. W. Ebright , R. H. Ebright , Antimicrob. Agents Chemother. 2012, 56, 6250–6255. 23006749 10.1128/AAC.01060-12 PMC3497154

[668] S. I. Maffioli , Y. Zhang , D. Degen , T. Carzaniga , G. Del Gatto , S. Serina , P. Monciardini , C. Mazzetti , P. Guglierame , G. Candiani , A. I. Chiriac , G. Facchetti , P. Kaltofen , H.-G. Sahl , G. Dehò , S. Donadio , R. H. Ebright , Cell 2017, 169, 1240–1248.e23. 28622509 10.1016/j.cell.2017.05.042 PMC5542026

[669] F. Glaus , D. Dedić , P. Tare , V. Nagaraja , L. Rodrigues , J. A. Aínsa , J. Kunze , G. Schneider , R. C. Hartkoorn , S. T. Cole , K.-H. Altmann , J. Org. Chem. 2018, 83, 7150–7172. 29542926 10.1021/acs.joc.8b00193

[670] R. H. Ebright, (Rutgers, the State University of New Jersey), WO2013119564A1, **2013**.

[671] R. Jansen , G. Höfle , H. Irschik , H. Reichenbach , Liebigs Ann. Chem. 1985, 1985, 822–836.

[672] J. Balansky , K. Pfarr , C. Szekat , S. Kehraus , T. Aden , M. Grosse , R. Jansen , T. Hesterkamp , A. Schiefer , G. M. König , M. Stadler , A. Hoerauf , G. Bierbaum , Antibiotics 2022, 11, 920. 35884174 10.3390/antibiotics11070920 PMC9311656

[673] A. Schiefer , A. Schmitz , T. F. Schäberle , S. Specht , C. Lämmer , K. L. Johnston , D. G. Vassylyev , G. M. König , A. Hoerauf , K. Pfarr , J. Infect. Dis. 2012, 206, 249–257. 22586066 10.1093/infdis/jis341 PMC3490692

[674] J. A. Trischman , D. M. Tapiolas , P. R. Jensen , R. Dwight , W. Fenical , T. C. MCKEE , C. M. Ireland , T. J. Stout , J. Clardy , J. Am. Chem. Soc. 1994, 116, 757–758.

[675] A. Baron , C. L. Sann , J. Mann , Bioorg. Med. Chem. 2022, 58, 116656. 35183028 10.1016/j.bmc.2022.116656

[676] E. J. C. Goldstein , D. M. Citron , K. L. Tyrrell , C. V. Merriam , Antimicrob. Agents Chemother. 2013, 57, 4872–4876. 23877700 10.1128/AAC.01136-13 PMC3811411

[677] D. Corbett , A. Wise , S. Birchall , P. Warn , S. D. Baines , G. Crowther , J. Freeman , C. H. Chilton , J. Vernon , M. H. Wilcox , R. J. Vickers , J. Antimicrob. Chemother. 2015, 70, 1751–1756. 25652750 10.1093/jac/dkv006 PMC4498293

[678] S. D. Baines , G. S. Crowther , J. Freeman , S. Todhunter , R. Vickers , M. H. Wilcox , J. Antimicrob. Chemother. 2015, 70, 182–189. 25190720 10.1093/jac/dku324 PMC4267497

[679] D. A. Collins , T. V. Riley , Lett. Appl. Microbiol. 2022, 75, 526–536. 35119124 10.1111/lam.13664 PMC9541751

[680] Summit Therapeutics. Safety, Tolerability and the Pharmacokinetics of Ridinilazole in Adolescent Subjects (Ri-CoDIFy 3), ClinicalTrials.gov NCT04802837. Updated August 21, **2023**. Accessed November 28, 2024. https://clinicaltrials.gov/study/NCT04802837.

[681] J. Yao , J. B. Maxwell , C. O. Rock , J. Biol. Chem. 2013, 288, 36261–36271. 24189061 10.1074/jbc.M113.512905 PMC3868742

[682] J. H. Yum , C. K. Kim , D. Yong , K. Lee , Y. Chong , C. M. Kim , J. M. Kim , S. Ro , J. M. Cho , Antimicrob. Agents Chemother. 2007, 51, 2591–2593. 17420210 10.1128/AAC.01562-06 PMC1913239

[683] A. K. Brown , R. C. Taylor , A. Bhatt , K. Fütterer , G. S. Besra , PLoS One 2009, 4, e6306. 19609444 10.1371/journal.pone.0006306 PMC2707616

[684] G. A. I. Moustafa , S. Nojima , Y. Yamano , A. Aono , M. Arai , S. Mitarai , T. Tanaka , T. Yoshimitsu , MedChemComm 2013, 4, 720–723.

[685] M. P. Singh , M. J. Mroczenski-Wildey , D. A. Steinberg , R. J. Andersen , W. M. Maiese , M. Greenstein , J. Antibiot. 1997, 50, 270–273. 9439702

[686] M. A. Silvers , G. T. Robertson , C. M. Taylor , G. L. Waldrop , J. Med. Chem. 2014, 57, 8947–8959. 25280369 10.1021/jm501082n

[687] K. Maeda , J. Antibiot. Ser. A 1957, 10, 94–106. 13449017

[688] O. K. Onajole , X. V. Belewa , Y. Coovadia , T. Govender , H. G. Kruger , G. E. M. Maguire , D. Naidu , B. Somai , N. Singh , P. Govender , Med. Chem. Res. 2011, 20, 1394–1401.

[689] B. V. Nikonenko , R. Samala , L. Einck , C. A. Nacy , Antimicrob. Agents Chemother. 2004, 48, 4550–4555. 15561824 10.1128/AAC.48.12.4550-4555.2004 PMC529200

[690] M. N. Protopopova, L. Einck, B. Nikonenko, P. Chen, (Sequella, Inc.), WO2007005896A2, **2007**.

[691] R. E. Lee , M. Protopopova , E. Crooks , R. A. Slayden , M. Terrot , C. E. Barry , J. Comb. Chem. 2003, 5, 172–187. 12625709 10.1021/cc020071p

[692] I. T. Malik , R. Pereira , M.-T. Vielberg , C. Mayer , J. Straetener , D. Thomy , K. Famulla , H. Castro , P. Sass , M. Groll , H. Brötz-Oesterhelt , ChemBioChem 2020, 21, 1997–2012. 32181548 10.1002/cbic.201900787 PMC7496096

[693] B. Hinzen, H. Brötz-Oesterhelt, R. Endermann, K. Henninger, H. Paulsen, S. Raddatz, T. Lampe, V. Hellwig, A. Schumacher (Bayer Healthcare AG), WO2003024996A2, **2003**.

[694] K. Weinhäupl , M. Brennich , U. Kazmaier , J. Lelievre , L. Ballell , A. Goldberg , P. Schanda , H. Fraga , J. Biol. Chem. 2018, 293, 8379–8393. 29632076 10.1074/jbc.RA118.002251 PMC5986217

[695] N. Bürstner , S. Roggo , N. Ostermann , J. Blank , C. Delmas , F. Freuler , B. Gerhartz , A. Hinniger , D. Hoepfner , B. Liechty , M. Mihalic , J. Murphy , D. Pistorius , M. Rottmann , J. R. Thomas , M. Schirle , E. K. Schmitt , ChemBioChem 2015, 16, 2433–2436. 26472355 10.1002/cbic.201500472

[696] B. Zhou , P. S. Achanta , G. Shetye , S.-N. Chen , H. Lee , Y.-Y. Jin , J. Cheng , M.-J. Lee , J.-W. Suh , S. Cho , S. G. Franzblau , G. F. Pauli , J. B. McAlpine , J. Nat. Prod. 2021, 84, 2644–2663. 34628863 10.1021/acs.jnatprod.1c00198 PMC8865217

[697] E. Higashide , M. Shibata , H. Yamamoto , K. Nakazawa , H. Iwasaki , J. Ueyanagi , A. Miyake , Agric. Biol. Chem. 1962, 26, 234–237.

[698] W. Gao , J.-Y. Kim , S.-N. Chen , S.-H. Cho , J. Choi , B. U. Jaki , Y.-Y. Jin , D. C. Lankin , J.-E. Lee , S.-Y. Lee , J. B. McAlpine , J. G. Napolitano , S. G. Franzblau , J.-W. Suh , G. F. Pauli , Org. Lett. 2014, 16, 6044–6047. 25409285 10.1021/ol5026603 PMC5450905

[699] M. C. Stone , A. Mychack , K. A. Coe , S. Walker , Antimicrob. Agents Chemother. 2023, 67, e00115–23. 37097175 10.1128/aac.00115-23 PMC10190671

[700] H. S. Girgis , C. D. DuPai , J. Lund , J. Reeder , J. Guillory , S. Durinck , Y. Liang , J. Kaminker , P. A. Smith , E. Skippington , Proc. Natl. Acad. Sci. USA 2021, 118, e2021958118. 33443214 10.1073/pnas.2021958118 PMC7817135

[701] D. Griffith , Y. Carmeli , S. Gehrke , E. Morgan , M. Dudley , J. Loutit , Open Forum Infect. Dis. 2022, 9, ofac492.295.

[702] X. Zhao , O. P. Kuipers , Front. Microbiol. 2021, 12, 693117. 34177875 10.3389/fmicb.2021.693117 PMC8219939

[703] X. Zhao , X. Zhong , S. Yang , K. Deng , L. Liu , X. Song , Y. Zou , L. Li , X. Zhou , R. Jia , J. Lin , H. Tang , G. Ye , J. Yang , S. Zhao , Y. Lang , H. Wan , Z. Yin , O. P. Kuipers , Antimicrob. Agents Chemother. 2023, 67, e00010–23. 36912655 10.1128/aac.00010-23 PMC10190627

[704] S. Cochrane, R. Ballantine, N. Martin, K. A. Ayed, M. Hoekstra, S. D. Z. Losada, (The Queen's University Of Belfast & Universiteit Leiden), WO2022162332A1, **2022**.

[705] R. P. Kowalski , E. G. Romanowski , K. A. Yates , F. S. Mah , J. Ocul. Pharmacol. Ther. 2016, 32, 23–27. 26501484 10.1089/jop.2015.0098 PMC4742993

[706] I. Nicolas , V. Bordeau , A. Bondon , M. Baudy-Floc'h , B. Felden , PLoS Biol. 2019, 17, e3000337. 31287812 10.1371/journal.pbio.3000337 PMC6615598

[707] Cellceutix Corporation. A Randomized, Double-Blind Study Comparing Three Dosing Regimens of Brilacidin to Daptomycin in the Treatment of Acute Bacterial Skin and Skin Structure Infections (ABSSSI). ClinicalTrials.gov identifyer: NCT02052388. Updated September 26, **2018**. Accessed November 28, 2024. https://clinicaltrials.gov/study/NCT02052388.

[708] B. Mensa , G. L. Howell , R. Scott , W. F. DeGrado , Antimicrob. Agents Chemother. 2014, 58, 5136–5145. 24936592 10.1128/AAC.02955-14 PMC4135847

[709] Cellceutix Corporation. “*Synthetic Novel Host Defense Protein Mimetics for the Treatment of Gram-Negative Bacterial Infections”*, ECMIID 2015 Copenhagen, **2015**.

[710] K. Reder-Christ , H. Falkenstein-Paul , G. Klocek , S. Al-Kaddah , U. Bakowsky , G. Bendas , Int. J. Antimicrob. Agents 2011, 37, 256–260. 21306875 10.1016/j.ijantimicag.2010.11.024

[711] H. Labischinski, S. Pelzer, H. Priefert, A. Vente, S.-E. Wohlert, (Merlion Pharmaceuticals GmbH), WO2007057005A1, **2007**.

[712] T. Schneider, K. Gries, I. Wiedemann, S. Pelzer, H. Labischinski, H. G. Sahl, 47^th^ ICAAC Chicago, **2007**. can be found under https://www.merlionpharma.com/fileadmin/user upload/04-news-presentations/04–03-presentations-poster/2007/ICAAC2007PosterAll.pdf, uploaded 28 November 2024.

[713] N. Scherr , R. Bieri , S. S. Thomas , A. Chauffour , N. P. Kalia , P. Schneide , M.-T. Ruf , A. Lamelas , M. S. S. Manimekalai , G. Grüber , N. Ishii , K. Suzuki , M. Tanner , G. C. Moraski , M. J. Miller , M. Witschel , V. Jarlier , G. Pluschke , K. Pethe , Nat. Commun. 2018, 9, 5370. 30560872 10.1038/s41467-018-07804-8 PMC6299076

[714] N. P. Kalia , B. Shi Lee , N. B. Ab Rahman , G. C. Moraski , M. J. Miller , K. Pethe , Sci. Rep. 2019, 9, 8608. 31197236 10.1038/s41598-019-44887-9 PMC6565617

[715] K. Arora , B. Ochoa-Montaño , P. S. Tsang , T. L. Blundell , S. S. Dawes , V. Mizrahi , T. Bayliss , C. J. Mackenzie , L. A. T. Cleghorn , P. C. Ray , P. G. Wyatt , E. Uh , J. Lee , C. E. Barry , H. I. Boshoff , Antimicrob. Agents Chemother. 2014, 58, 6962–6965. 25155596 10.1128/AAC.03486-14 PMC4249445

[716] A. Lupien , C. S.-Y. Foo , S. Savina , A. Vocat , J. Piton , N. Monakhova , A. Benjak , D. A. Lamprecht , A. J. C. Steyn , K. Pethe , V. A. Makarov , S. T. Cole , PLoS Pathog. 2020, 16, e1008270. 31971990 10.1371/journal.ppat.1008270 PMC6999911

[717] T. A. Yates , L. A. Tomlinson , K. Bhaskaran , S. Langan , S. Thomas , L. Smeeth , I. J. Douglas , PLoS Med. 2017, 14, e1002457. 29161254 10.1371/journal.pmed.1002457 PMC5697821

[718] J. Jia , C. Zhang , Y. Liu , Y. Huang , Y. Bai , X. Hang , L. Zeng , D. Zhu , H. Bi , Microb. Biotechnol. 2022, 15, 442–454. 33780131 10.1111/1751-7915.13807 PMC8867979

[719] C. Dufour , J. Wink , M. Kurz , H. Kogler , H. Olivan , S. Sablé , W. Heyse , M. Gerlitz , L. Toti , A. Nußer , A. Rey , C. Couturier , A. Bauer , M. Brönstrup , Chem. Eur. J. 2012, 18, 16123–16128. 23143837 10.1002/chem.201201635

[720] Z. Hussain , S. Pengfei , L. Yimin , L. Shasha , L. Zehao , Y. Yifan , L. Linhui , Z. Linying , W. Yong , Pathog. Dis. 2022, 80, ftac037. 36152595 10.1093/femspd/ftac037

[721] G. Geberetsadik , A. Inaizumi , A. Nishiyama , T. Yamaguchi , H. Hamamoto , S. Panthee , A. Tamaru , M. Hayatsu , Y. Mizutani , S. A. Kaboso , M. Hakamata , A. Ilinov , Y. Ozeki , Y. Tateishi , K. Sekimizu , S. Matsumoto , Antimicrob. Agents Chemother. 2022, 66, e00171–22. 35969044 10.1128/aac.00171-22 PMC9487456

[722] A. P. Tomaras , C. J. McPherson , M. Kuhn , A. Carifa , L. Mullins , D. George , C. Desbonnet , T. M. Eidem , J. I. Montgomery , M. F. Brown , U. Reilly , A. A. Miller , J. P. O'Donnell , mBio 2014, 5, e01551–14. 25271285 10.1128/mBio.01551-14 PMC4196226

[723] M. F. Brown, Y. Che, A. Marfat, M. J. Melnick, J. I. Montgomery, U. Reilly (Pfizer Inc.), WO2011073845A1, **2011**.

[724] J. I. Montgomery , M. F. Brown , U. Reilly , L. M. Price , J. A. Abramite , J. Arcari , R. Barham , Y. Che , J. M. Chen , S. W. Chung , E. M. Collantes , C. Desbonnet , M. Doroski , J. Doty , J. J. Engtrakul , T. M. Harris , M. Huband , J. D. Knafels , K. L. Leach , S. Liu , A. Marfat , L. McAllister , E. McElroy , C. A. Menard , M. Mitton-Fry , L. Mullins , M. C. Noe , J. O'Donnell , R. Oliver , J. Penzien , M. Plummer , V. Shanmugasundaram , C. Thoma , A. P. Tomaras , D. P. Uccello , A. Vaz , D. G. Wishka , J. Med. Chem. 2012, 55, 1662–1670. 22257165 10.1021/jm2014875

[725] R. Kasar, M. S. Linsell, J. B. Aggen, Q. Lu (Jane), D. Wang, T. Church, H. E. Moser, P. A. Patten (Achaogen, Inc.), WO2012154204A1, **2012**.

[726] K. M. Krause , C. M. Haglund , C. Hebner , A. W. Serio , G. Lee , V. Nieto , F. Cohen , T. R. Kane , T. D. Machajewski , D. Hildebrandt , C. Pillar , M. Thwaites , D. Hall , L. Miesel , M. Hackel , A. Burek , L. D. Andrews , E. Armstrong , L. Swem , A. Jubb , R. T. Cirz , Antimicrob. Agents Chemother. 2019, 63, e00977–19. 31451507 10.1128/AAC.00977-19 PMC6811409

[727] Achaogen, Inc. A Study to Assess the Safety, Tolerability, and Pharmacokinetics of ACHN-975 in Healthy Volunteers, Clinicaltrials.Gov identifyer: NCT01597947. Udated on January 21, **2013**. Accessed November 28, 2024. https://clinicaltrials.gov/study/NCT01597947.

[728] M. Díez-Aguilar , M. Hernández-García , M.-I. Morosini , A. Fluit , M. M. Tunney , N. Huertas , R. del Campo , D. Obrecht , F. Bernardini , M. Ekkelenkamp , R. Cantón , J. Antimicrob. Chemother. 2021, 76, 984–992. 33367642 10.1093/jac/dkaa529

[729] M. J. Melchers , J. Teague , P. Warn , J. Hansen , F. Bernardini , A. Wach , D. Obrecht , G. E. Dale , J. W. Mouton , Antimicrob. Agents Chemother. 2019, 63, e01699–18. 30642931 10.1128/AAC.01699-18 PMC6395900

[730] J. Schmidt , K. Patora-Komisarska , K. Moehle , D. Obrecht , J. A. Robinson , Bioorg. Med. Chem. 2013, 21, 5806–5810. 23932450 10.1016/j.bmc.2013.07.013

[731] B. Ma , C. Niu , Y. Zhou , X. Xue , J. Meng , X. Luo , Z. Hou , Antimicrob. Agents Chemother. 2016, 60, 4283–4289. 27161645 10.1128/AAC.00041-16 PMC4914623

[732] A. Guenther , L. Millar , A. Messer , M. Giraudon , K. Patel , E. J. Deurloo , M. Lobritz , A. Gloge , Open Forum Infect. Dis. 2023, 10, ofad500.1749.

[733] L. P. Jordheim , S. Ben Larbi , O. Fendrich , C. Ducrot , E. Bergeron , C. Dumontet , J. Freney , A. Doléans-Jordheim , Int. J. Antimicrob. Agents 2012, 39, 444–447. 22445492 10.1016/j.ijantimicag.2012.01.019

[734] T. A. Keating , J. V. Newman , N. B. Olivier , L. G. Otterson , B. Andrews , P. A. Boriack-Sjodin , J. N. Breen , P. Doig , J. Dumas , E. Gangl , O. M. Green , S. Y. Guler , M. F. Hentemann , D. Joseph-McCarthy , S. Kawatkar , A. Kutschke , J. T. Loch , A. R. McKenzie , S. Pradeepan , S. Prasad , G. Martínez-Botella , ACS Chem. Biol. 2012, 7, 1866–1872. 22908966 10.1021/cb300316n

[735] W. Kim , N. Fricke , A. L. Conery , B. B. Fuchs , R. Rajamuthiah , E. Jayamani , P. M. Vlahovska , F. M. Ausubel , E. Mylonakis , Future Med. Chem. 2016, 8, 257–269. 26910612 10.4155/fmc.15.189 PMC4976882

